# Integrating Transcriptomic and Proteomic Data Using Predictive Regulatory Network Models of Host Response to Pathogens

**DOI:** 10.1371/journal.pcbi.1005013

**Published:** 2016-07-12

**Authors:** Deborah Chasman, Kevin B. Walters, Tiago J. S. Lopes, Amie J. Eisfeld, Yoshihiro Kawaoka, Sushmita Roy

**Affiliations:** 1 Wisconsin Institute for Discovery, University of Wisconsin—Madison, Madison, Wisconsin, United States of America; 2 Influenza Research Institute, Department of Pathobiological Sciences, School of Veterinary Medicine, University of Wisconsin—Madison, Madison, Wisconsin, United States of America; 3 Division of Virology, Department of Microbiology and Immunology, Institute of Medical Science, University of Tokyo, Tokyo, Japan; 4 Department of Special Pathogens, International Research Center for Infectious Diseases, Institute of Medical Science, University of Tokyo, Tokyo, Japan; 5 Laboratory of Bioresponses Regulation, Department of Biological Responses, Institute for Virus Research, Kyoto University, Kyoto, Japan; 6 Department of Computer Sciences, University of Wisconsin—Madison, Madison, Wisconsin, United States of America; 7 Department of Biostatistics and Medical Informatics, University of Wisconsin—Madison, Madison, Wisconsin, United States of America; Memorial Sloan-Kettering Cancer Center, UNITED STATES

## Abstract

Mammalian host response to pathogenic infections is controlled by a complex regulatory network connecting regulatory proteins such as transcription factors and signaling proteins to target genes. An important challenge in infectious disease research is to understand molecular similarities and differences in mammalian host response to diverse sets of pathogens. Recently, systems biology studies have produced rich collections of omic profiles measuring host response to infectious agents such as influenza viruses at multiple levels. To gain a comprehensive understanding of the regulatory network driving host response to multiple infectious agents, we integrated host transcriptomes and proteomes using a network-based approach. Our approach combines expression-based regulatory network inference, structured-sparsity based regression, and network information flow to infer putative physical regulatory programs for expression modules. We applied our approach to identify regulatory networks, modules and subnetworks that drive host response to multiple influenza infections. The inferred regulatory network and modules are significantly enriched for known pathways of immune response and implicate apoptosis, splicing, and interferon signaling processes in the differential response of viral infections of different pathogenicities. We used the learned network to prioritize regulators and study virus and time-point specific networks. RNAi-based knockdown of predicted regulators had significant impact on viral replication and include several previously unknown regulators. Taken together, our integrated analysis identified novel module level patterns that capture strain and pathogenicity-specific patterns of expression and helped identify important regulators of host response to influenza infection.

## Author Summary

An important challenge in infectious disease research is to understand how the human immune system responds to different types of pathogenic infections. An important component of mounting proper response is the transcriptional regulatory network that specifies the context-specific gene expression program in the host cell. However, our understanding of this regulatory network and how it drives context-specific transcriptional programs is incomplete. To address this gap, we performed a network-based analysis of host response to influenza viruses that integrated high-throughput mRNA- and protein measurements and protein-protein interaction networks to identify virus and pathogenicity-specific modules and their upstream physical regulatory programs. We inferred regulatory networks for human cell line and mouse host systems, which recapitulated several known regulators and pathways of the immune response and viral life cycle. We used the networks to study time point and strain-specific subnetworks and to prioritize important regulators of host response. We predicted several novel regulators, both at the mRNA and protein levels, and experimentally verified their role in the virus life cycle based on their ability to significantly impact viral replication.

## Introduction

To combat infections from diverse pathogens, mammalian immune systems must be able to mount appropriate and specific responses to pathogenic infections. A key challenge in current infectious disease research is to understand the molecular mechanisms that make the host immune system more or less susceptible to a particular strain of a pathogen, for example, different influenza A virus strains, than another. Transcriptional regulatory networks that connect regulatory proteins to target genes are central players in how mammalian cells mount appropriate responses to different pathogenic infections. Because the components and connectivity of these networks are largely not known, a significant amount of effort has been invested to collect high-throughput datasets that provide a comprehensive molecular characterization of host response to multiple viruses at multiple levels, including the transcriptome and proteome [1–4]. These genomic datasets provide unique opportunities to identify molecular network components of host response that are conserved across multiple viruses or specific to a virus of a particular pathogenicity. Such networks can be used to prioritize regulators for follow up validation studies that provide greater insight into the mechanisms by which the host cell perceives and responds to different pathogenic infections.

Network- and module-based approaches for analyzing omic datasets have been powerful for dissecting mammalian cellular response to different environmental perturbations, including response to various pathogenic infections [5,6]. The majority of these approaches have used genome-wide transcriptomic data and can be grouped into those that infer correlational networks [3,7–12], or Module Networks, in which regulators are inferred for groups of co-expressed genes called modules [9,13,14]. A few approaches have integrated additional data types, e.g. physical protein-protein or signaling networks with transcriptional data [15–20], that vary in the number of samples needed to infer networks. While these approaches have provided important insights into the host response, they have not integrated multiple types of omic measurements (e.g. mRNA and protein levels), which can differ in quality and sample size. A second challenge is that experimental validation of large-scale predictions is expensive and most of the generated predictions have not been validated experimentally. Although several network-based prioritization methods have been proposed, the results of a prioritization scheme have been followed up with experimental validation in a handful of studies [21].

To gain a more complete understanding of the molecular networks driving host response, we integrated transcriptomic and proteomic measurements of mammalian host response with existing protein-protein interactions. These omic measurements capture human and mouse cellular response to multiple Influenza A viruses exhibiting different levels of pathogenicities. Our starting point is an expression-based regulatory network connecting transcription factors and signaling proteins to target genes and gene modules. We then use proteomic measurements to predict additional regulators of gene modules by applying a structured sparsity-inducing regression approach, Multi-Task Group LASSO, to find proteins whose levels are predictive of mRNA levels of entire modules. By decoupling the mRNA and protein-based regression into two steps, our approach is less sensitive to varying sample size for each type of omic data. Finally, we predict physical regulatory programs connecting mRNA and protein-based regulators through a small number intermediate nodes using Integer Linear Programming (ILP)-based network information flow.

We used our integrated regulatory networks to study the host response across the different viruses. We tested prioritized regulators using small interfering RNAs and found several regulators that significantly impact viral replication, five of which have not been previously associated with influenza related response. We examined host response dynamics at the network level, identifying regulatory network components that are active or missing under different viral treatments. Our inferred gene modules capture strain- and pathogenicity-specific patterns of mammalian immune response to influenza infections that recapitulate and expand upon known immune response pathways. In particular, we identified one module that was enriched for interferon signaling and exhibited repressed expression in the high-pathogenicity wild-type H5N1, but was induced in low pathogenicity H1N1 strains. Another module suggests that apoptosis-related pathways might be down-regulated in low-pathogenicity viruses. Our findings of host regulatory modules together with their upstream regulatory programs suggest that our network-based approach is a powerful way to systematically characterize immune response to diverse pathogenic infections.

## Results

### Inferred regulatory modules and network interactions captured immune response processes and identified conservation between host systems

**Fig 1:**
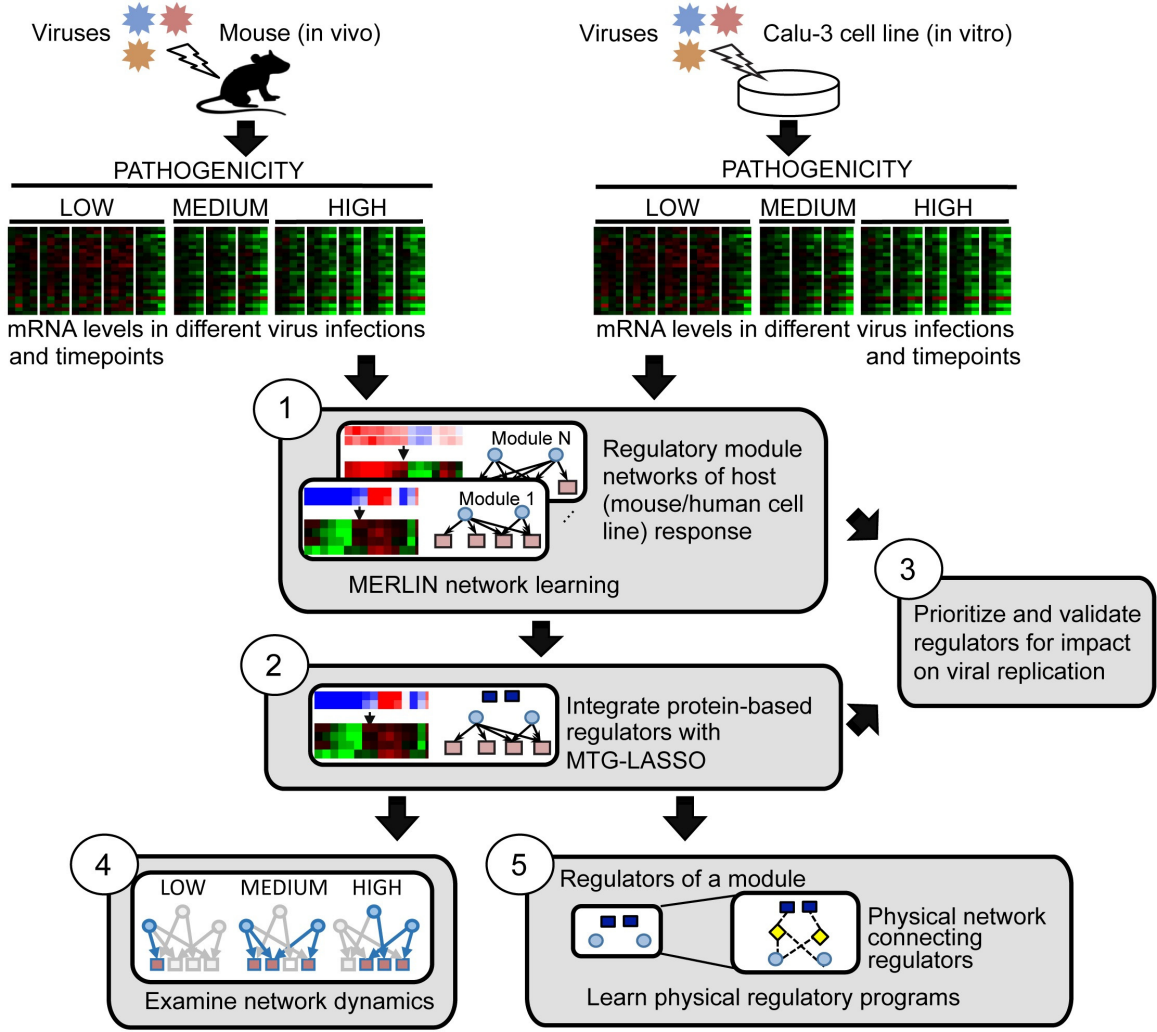
Integrative inference of regulatory networks controlling host response to influenza infection. The main components of our approach are: (1) MERLIN to learn regulatory networks and modules from genome-wide host mRNA profiles from multiple independent virus infections, (2) Multi-Task Group LASSO (MTG-LASSO) approach to predict protein-level regulators (dark blue squares) of mRNA-based modules, (3) construction of active network components for different virus strains and time points, (4) siRNA-based validation of predicted regulators, (5) predicting upstream signaling networks for each module by identifying minimal physical subnetworks that connect module regulators (dark blue squares and light blue ellipses) with a small number of intermediate nodes (yellow diamonds).

To perform a systematic, integrative analysis of transcriptome and proteome measurements of host response to influenza virus infections, we began by inferring a regulatory module network in two stages, followed by three major downstream analyses (**Fig 1**). We (1) infer a regulatory module network based on changes in mRNA abundance under viral infection and (2) predict protein regulators whose abundances are predictive of gene expression in the modules. This integrated regulatory module network enabled (3) prioritization of regulators for validation of their ability to modulate viral replication, (4) an examination of network dynamics across virus treatments, and (5) a further integration with external protein-protein interactions to predict directed physical connections between the mRNA, protein-based regulators and known influenza host response genes.

**Fig 2:**
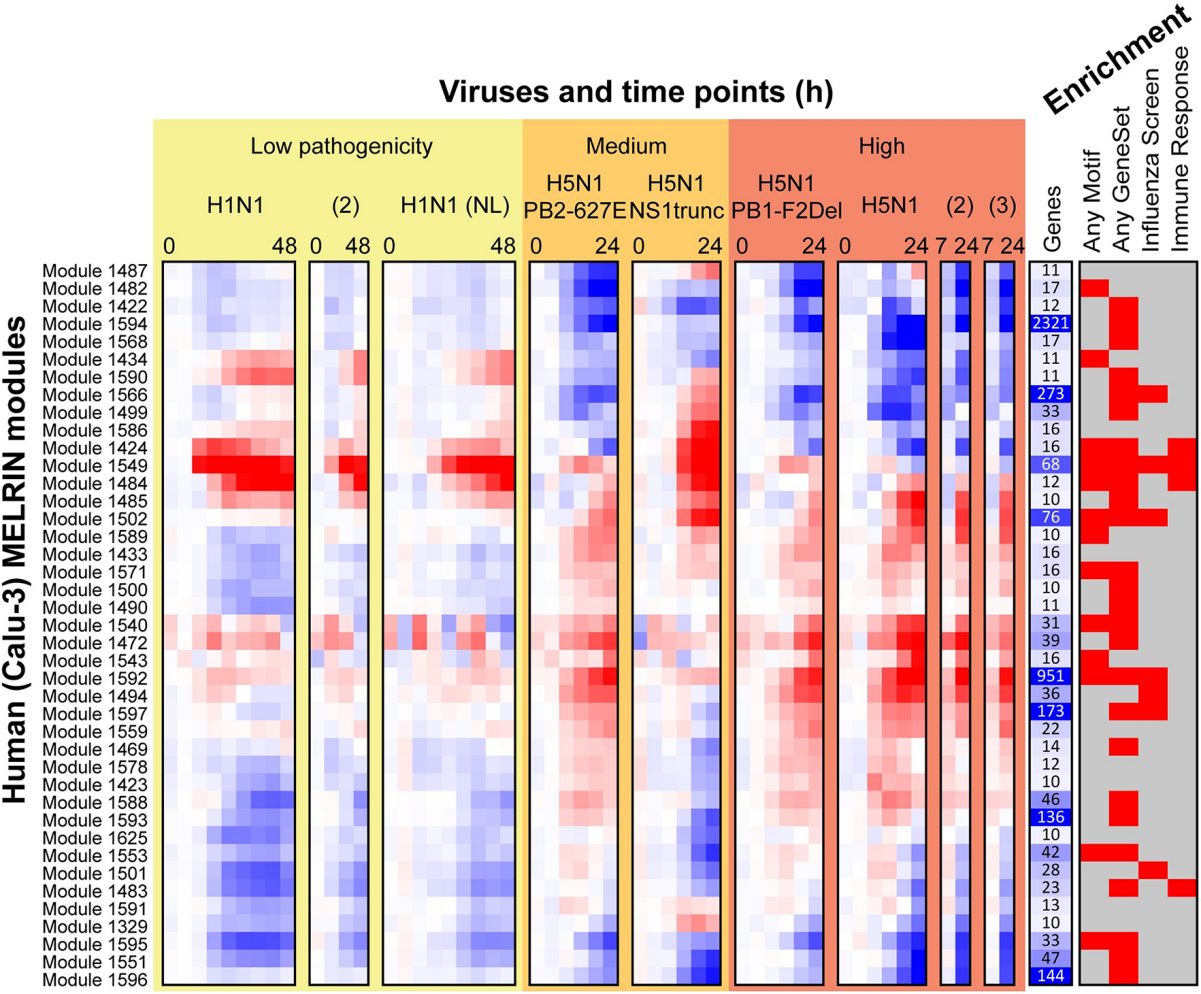
Overview of human influenza response module expression patterns. Shown are 41 human Calu-3 modules with at least 10 genes. The red-blue heat map shows mean expression of all genes in each module. The more red an entry the higher the expression in infected versus mock, while the more blue the entry, the higher the expression in mock compared to the infected sample. Under “Viruses and time points (h)”, each virus treatment's time series is shown separately, and viruses are ordered from low to high pathogenicity. Low-pathogenicity samples labeled H1N1 used A/CA/04/09 H1N1 unless labeled '(NL)' for A/Netherlands/602/09. Samples labeled H5N1 used A/VN/1203/04 H5N1 and laboratory mutants thereof. The “Genes” column shows the size of each module; larger values are shown in darker blue. Under “Enrichment”, a red box indicates module enrichment with any MSigDB motif, any MSigDB gene set, any Gene ontology process, any influenza screen set, or any immune response gene set (**Methods**).

**Fig 3:**
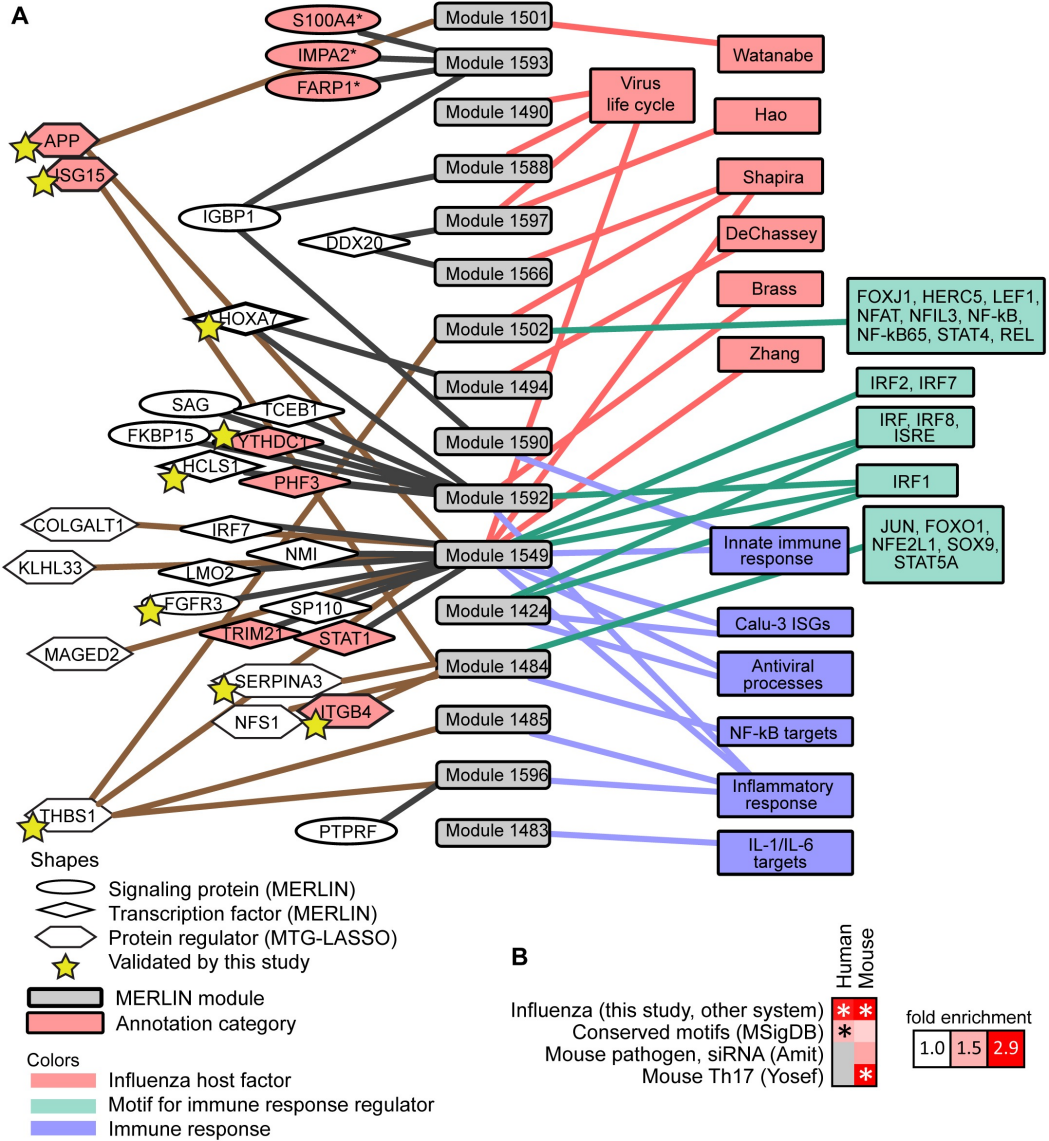
Overview of human influenza response modules and top predicted regulators. **A.** A selection of sixteen human modules (grey, center), with their top predicted regulators (shapes on left, connected to modules), and enriched gene sets relevant to immune response or viral life cycle (colored boxes, right). Modules were included in this figure if they were enriched for one of the following categories: (a) influenza host genes (pink) identified by RNAi, protein-protein interactions, expert curation (Zhang et al. 2009), or enrichment for other virus life cycle gene sets (from GO or KEGG); (b) motifs (green) for transcriptional regulators whose annotations indicate relevance to the innate immune response; (c) innate immune response gene sets (blue) from experimental results or manual curation. One module, Cluster 1593, is also included because its consensus regulator set (but not targets) was enriched for host proteins that are involved in the influenza life cycle from Watanabe et al (2014). Also shown are seventeen (ellipses and diamonds) predicted mRNA-level regulators, and ten predicted protein regulators (hexagons), that are associated with these modules. Regulators with evidence for influenza relevance are shaded in pink. Host genes that significantly impact viral replication identified by this study (APP, FGFR3, HCLS1, HOXA7, SERPINA3) are indicated with yellow stars. **B.** Comparison of the MERLIN inferred mouse and human influenza response networks ("Influenza (this study, other system)") to each other and to other regulatory networks. Fold enrichment of shared edges over expected fraction is visualized in the heat map, with significant comparisons marked with asterisks (hypergeometric *p*-value < 0.05).

The first component of our approach, a regulatory network inference algorithm, was needed because mammalian regulatory networks are incomplete for most biological processes. We used a recently developed network inference algorithm, 'Modular regulatory network learning with per gene information' (MERLIN [22]) that uses genome-wide mRNA levels from multiple biological samples (time points or treatments) to predict regulatory relationships between regulators (e.g. transcription factors or signaling proteins) and target genes. Our rationale for selecting MERLIN was to enable the study of host response regulatory networks both at the individual gene level and at the module level. Alternative methods either infer the regulators for individual genes [23] or for gene modules [13,24], but not both. We applied MERLIN within a stability selection framework to the host transcriptional measurements of mouse lungs and human bronchial epithelial cells (Calu-3 cell line) infected with one of six influenza virus strains exhibiting a range of pathogenicity levels (**Materials and Methods**). On the human Calu-3 cell line (**Fig 2**), we identified 41 consensus modules of at least 10 genes comprising a total of 4,801 genes (~67% of the input, **Table 1**). On the mouse data, we identified 56 modules, encompassing 2,944 genes (41% of the input, **Table 1**). The average expression patterns in each inferred module revealed commonalities and differences between strains and pathogenicity levels (**Fig 2,** Calu-3; **S1 Fig,** mouse). Based on a hypergeometric test with FDR correction (FDR<0.05), 32 out of the 41 human Calu-3 modules (40 of 56 mouse modules) exhibited enrichment in one or more of the annotation categories representing Gene Ontology processes, KEGG pathways, and influenza related gene sets identified from 10 high-throughput RNAi studies and viral-host protein-protein interaction screens (**Fig 2**, **S1 Fig, S1 Table, S2 Table**). Moreover, 17 of the human modules were enriched specifically for immune response related processes (**Fig 3A**) suggesting that the modules were biologically coherent and relevant to immune response to influenza infections. Importantly, compared to ordinary expression-based gene clusters (identified by Gaussian Mixture Modeling, 40 clusters), MERLIN modules exhibited greater fold enrichment in innate immune system categories and motif-based targets of transcription factors (**S3 Table)**. In particular, MERLIN modules had enrichment for targets of immune response-relevant regulatory elements IRF1, 2, 7 and ISRE and targets of inflammatory response regulator NF-kB, while expression-based clusters were not enriched or enriched at a lower level.

**Table 1:** Catalog of MERLIN modules for human and mouse systems.

Host system	Module size (*m*)	Number of modules	Number of genes
**Human (Calu 3)**	*m* ≥ 500	2	3,272
	10 *≤ m ≤* 500	39	1,541
	*m <* 10		2,387
**Mouse (lung)**	*m* ≥ 500	2	1,526
	10 *≤ m ≤* 500	54	1,418
	*m <* 10		4,296

"Module size" refers to the number of genes in a module. 'Number of genes' gives the number of genes covered by modules of a given size.

In addition to the modules, we defined consensus regulatory networks for the Calu-3 and mouse transcriptome data by selecting regulatory edges with a confidence at least 0.3 (**Materials and Methods**). The consensus networks predicted regulatory connections between 1,250 regulators (signaling proteins and TFs) and 7,132 target genes in human, and 1,252 regulators and 7,134 target genes in mouse (**S4 Table, S5 Table**). Both Calu-3 and mouse regulatory networks were significantly enriched in transcription factor target interactions cataloged in MSigDB (**Fig 3B, Conserved Motifs** (**MSigDB**)) suggesting that the predicted regulatory-target connections are supported by sequence specific motifs. We also compared the inferred MERLIN mouse network to two additional networks: a pathogen-responsive regulatory network inferred from gene expression profiles after RNAi-based transcription factor knockdowns (**Fig 3B, Mouse pathogen, siRNA** [5]), and a computationally constructed regulatory network for Th17 cellular response to LPS stimulation (**Fig 3B, Mouse Th17, Yosef**, [21]). The MERLIN mouse network significantly overlaps with both of these networks (Fold enrichment > 1.5, **Fig 3B**), indicating that MERLIN’s predicted regulatory network interactions are recapitulated in other immune response regulatory networks.

To more directly test the predicted edges of MERLIN, we compared the predicted targets of four (IRF7, NMI, STAT1, TCEB1) of our top ranked regulators using two published experimentally generated networks obtained from genome-wide expression profiles in single-gene knockdown siRNA screens in other cell lines [25,26]. We found significant overlap (FDR<0.05) between the MERLIN and independently identified targets of three regulators (IRF7, NMI, STAT1), suggesting that the edges predicted in the MERLIN network are associated with functional changes in expression.

**Fig 4:**
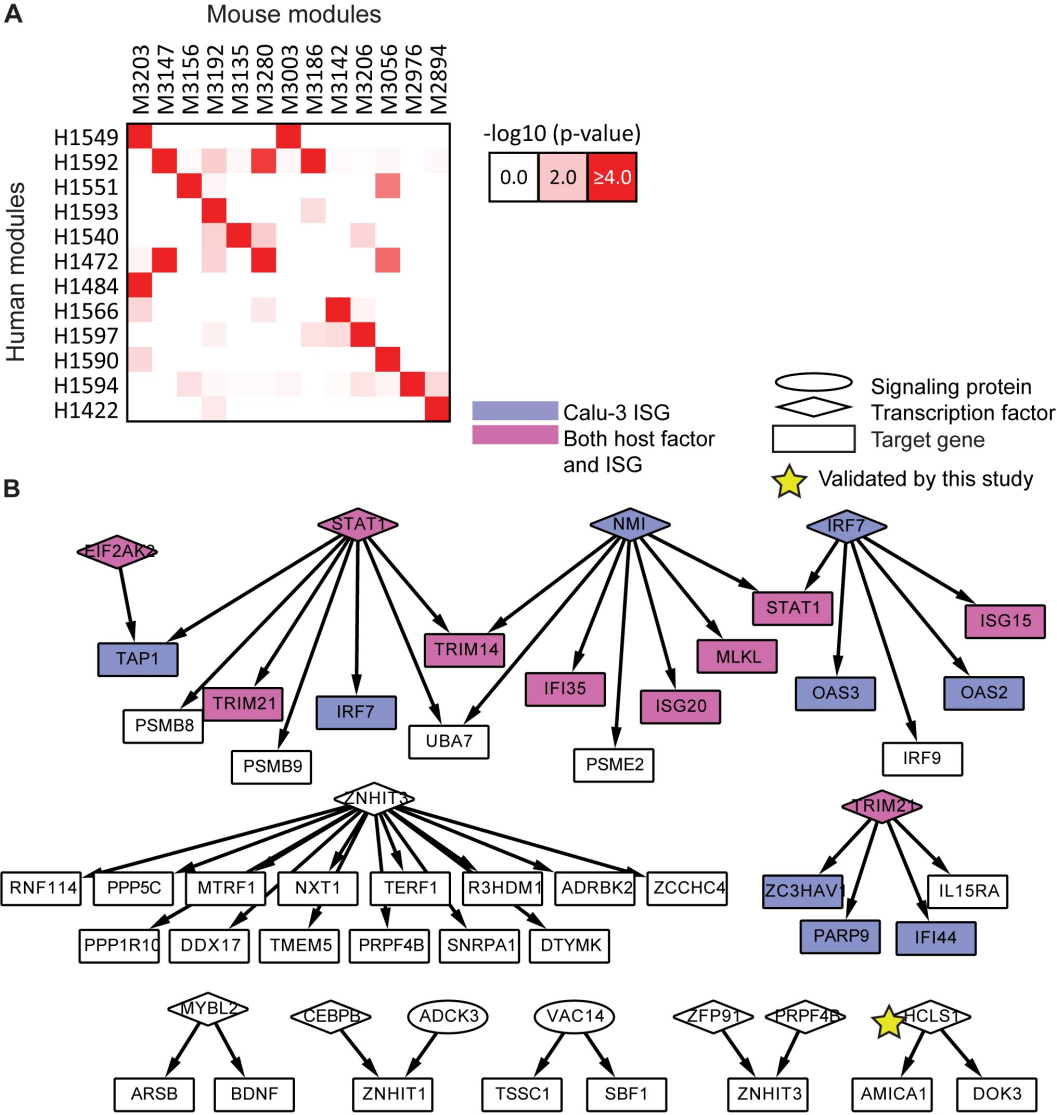
Conservation between human and mouse subnetworks. **A.** Modules conserved between human and mouse. There were fifteen (red shading) significantly overlapping module pairs (hypergeometric p-value < 0.0001), including 12 human modules and 13 mouse modules. **B.** A core set of interactions conserved between the human and mouse consensus regulatory networks. Shown are the 48 of 96 shared interactions that belong to subnetworks with at least three nodes. Shading indicates Calu-3 ISGs (violet) and nodes identified both as known host genes and ISGs (magenta). Star indicates a gene that significantly impacted viral replication on knockdown as identified in this study.

As a final type of evaluation we compared the extent of conservation of immune response of our two host systems to influenza infection (**Materials and Methods)**. At the module level, we estimated the significance of overlap of genes for all module pairs between the two species according to a hypergeometric test. We identified 15 pairs of modules that significantly overlapped in the gene content (*p*-value<0.05) between the two species (**Fig 4A**). The number of genes involved in this overlap was fairly modest, illustrating the challenges of integrating data from two separate organisms, with the mouse lung system being significantly more complex and heterogeneous than the human cell line system. Despite the low overlap in the number of genes, some of the cross-species module pairs shared enriched annotation categories. Specifically, Module 1549 in human and Module 3203 in mouse were both enriched for antigen processing and interferon-stimulated genes (ISGs), and human Module 1549 also had a significant overlap with mouse Module 3003, which was associated with cytokine signaling and innate immune system response. In another case, the mouse Module 3203 significantly overlapped with human Module 1484, with both being enriched with hallmarks of the adaptive immune response, namely, antigen processing, B cell activation and leukocyte and lymphocyte activation. Analogously, at the network level, the networks overlap significantly (hypergeometric test *p*-value<1e-4, fold enrichment 2.9, **Fig 3B**), including a core set of 96 interactions, 48 of which form subnetworks with at least 3 genes (**Fig 4B**). This conserved regulatory network contained many key players from the interferon production and JAK-STAT pathways (STAT1, NMI, IRF7; [27,28]) as well as regulators about which little is known, perhaps representing new relevant host processes. One conserved regulator with many conserved targets was ZNHIT3 (also known as TRIP3), a zinc finger HIT domain-containing protein that binds to thyroid hormone receptor [29], but which is otherwise poorly characterized in the pathway databases.

In summary, the enrichment of immune related functions, motif instances of known immune response TFs, agreement with existing computational and experimentally generated immune response related networks, and the conservation of the key immune related modules and networks between two distinct host systems is indicative of valid regulatory programs that can be explored with further experimental analysis.

### Predictive regulatory network-based prioritization and validation of regulators

We used the MERLIN inferred networks to develop a regulator prioritization strategy wherein regulators were ranked according to the loss in the MERLIN model's predictive accuracy when a regulator was omitted from its targets' regulatory programs (**Materials and Methods; S6 Table**). In comparison to three other ranking schemes (outgoing regression weight, out-degree, and right eigenvector centrality; **S1 Text**), this strategy identified the most known influenza host genes among high-ranking regulators (**S2 Fig**).

We next used these rankings to guide our experimental validation (**Materials and Methods**). We selected 20 regulators based primarily on the human rankings, the known annotations of the regulators, expression of the regulator’s module and to exclude well-studied immune response regulators (IRF7, NMI, STAT1). To experimentally validate our network-based prioritization scheme, we measured H1N1 virus replication in human lung epithelial cells (A549) following knockdown of predicted human regulators of host response by siRNA (**Materials and Methods, S7 Table**). For each gene, four siRNAs were used, in order to mitigate off-target or cytotoxic effects of single siRNAs. We called a gene a high confidence hit using a stringent criteria requiring at least two siRNAs (out of four used for each gene) to yield a statistically significant (t-test *p*-value <0.05) and high-magnitude (10 fold) change in virus titer compared to negative controls, and if none of the siRNAs yielded a significant change in the opposite direction. Using these strict criteria, three out of the twenty tested regulators were called hits: BOLA1, HCLS1, and HOXA7 (**Table 2**). Three additional regulators were medium-confidence hits with > 5 fold change but significant and consistent effects in multiple siRNAs: FGFR3, IRAK3, and YTHDC1. Knockdown of all six of the above consistently resulted in reduced virus titer, suggesting that the genes are important for virus production. Other tested regulators had multiple significant siRNAs, but conferred lower fold changes or showed divergent changes in viral titer between different siRNAs for the same gene. We note that some of the genes in our study for which multiple siRNAs had statistically significant but inconclusive direction or magnitude of effects were identified as hits by genome-wide screens (**S7 Table**). While understanding the detailed role of these predicted regulators in viral replication will require further experiments, these results suggest that our network inference and prioritization method can successfully identify important regulators of host response.

**Table 2:** Summary of results of siRNA validation study of twenty regulators predicted by MERLIN.

Result for four siRNAs	Regulator(s)
**High-confidence hit**: ≥ 2 reduce virus titer ≥ 10-fold, none induce	BOLA1, HCLS1, HOXA7
**Medium-confidence hit**: ≥ 2 reduce virus titer ≥ 5-fold, none induce	IRAK3, FGFR3, YTHDC1
≥ 2 reduce, none induce	KCNIP3, TCEB1
≥ 2 reduce, one induces	DDX20, FIG4, MET, PTPRF, SAG, TP53BP2, WDR81
≥ 2 induce, one reduces	IGBP1
Other	ANKRD2, FKBP15, PHF3, TNS53

Four siRNAs were used per gene. siRNA results are reported for significant changes in viral titer as assessed by T-test compared to negative control. Hits are defined as those that show a strong, significant and consistent effect across multiple siRNAs. Full data and results are available in S7 Table.

### MTG-LASSO enables integration of protein level regulators for gene modules

To integrate proteomic measurements with the host transcriptional response, we used a predictive modeling approach to identify proteins whose levels are predictive of the mRNA levels of gene modules. In theory, the MERLIN network inference algorithm could be used to integrate these proteins as additional regulators of a target gene’s expression levels. However, there were three reasons that prevented us from doing this. First, entire time courses of protein measurements were missing, and integration into the initial network inference step would require either extensive interpolation of entire time courses or excluding many mRNA measurements. Second, only ~20% of our candidate regulators (signaling proteins and TFs) with available mRNA levels were measured at the protein levels (**S4 Table**, **S5 Table**). Third, compared to mRNA levels of regulators, protein levels were not good as predictors of target gene mRNA level, likely because of the smaller dynamic range of proteomic measurements (**S3 Fig**).

**Fig 5:**
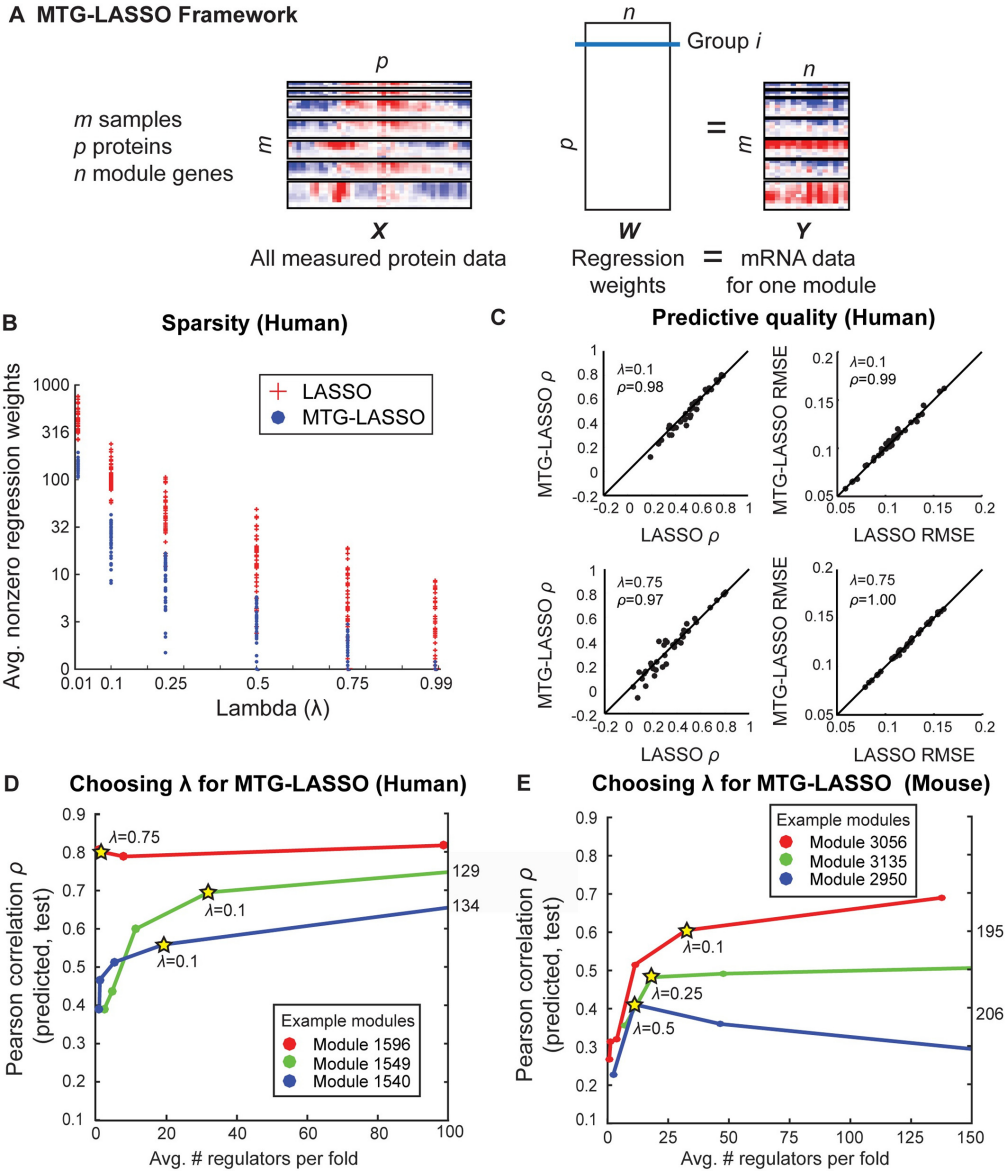
Multi-task Group LASSO (MTG-LASSO) structured-sparsity approach for integration of protein data with expression-based regulatory module networks. **A.** Illustration of MTG-LASSO framework for predicting protein regulators for one module (**Methods**). Horizontal separations in the ***X*** and ***Y*** data boxes represent different virus time course. Rows of ***X*** and ***Y*** represent time points. Columns of ***X*** correspond to proteins and columns of ***Y*** correspond to mRNA levels of genes in a module. **B.** Comparison of number of nonzero regression weights identified by MTG-LASSO and LASSO. Each dot (MTG-LASSO) or plus sign (LASSO) represents the number of non-zero regression weights for one setting of λ (the sparsity parameter) for one module. Number of nonzero weights is averaged over 10 folds of cross-validation. **C.** Comparison of cross-validation predictive quality between MTG-LASSO and LASSO. Results are shown for λ = {0.10, 0.75}; results for other settings are in **S4 Fig.** In each scatterplot, there is one point per module. Left two plots compare methods based on Pearson correlation (*ρ*) of predicted to actual expression values; right plots compare on the basis of root mean squared error (RMSE). Inset *ρ* gives Pearson correlation between MTG-LASSO and LASSO scores. Diagonal line is shown for comparison. **D/E.** Examples of curves used to select λ for individual modules; human (**D**) and mouse (**E**). Y-axis gives Pearson correlation (cross-validation predictive quality); X-axis gives the average number of nonzero regression weights for that module; this value is higher than the final number of high-confidence, high-weight regulators. Stars indicate the chosen value of λ for the example modules.

Our predictive modeling approach used a structured-sparsity based regression framework, called Multi-Task Group LASSO (MTG-LASSO, **Fig 5A**) [30,31]. Our approach is based on using group LASSO for multi-task feature selection [30,31]. Unlike LASSO [32], which solves one regression problem at a time, MTG-LASSO aims to solve multiple regression problems simultaneously, one for each gene in a module. Our regression formulation has two properties: (a) multi-task regression (where each task is the regression problem of each gene in a module) and (b) group LASSO, to enable selection of the same regulators (with possibly different regression weights) for all genes in a module. LASSO-based approaches have been applied extensively for mRNA-based regulatory network inference [24,33]; however, to our knowledge, our MTG-LASSO approach is the first to employ grouping structure in order to integrate sparse protein-level data with comparatively higher-coverage mRNA-level data. A module-based regression enables us to pool information from all genes in the module to select regulators that are informative for all genes in the module.

The MTG-LASSO regression problem is illustrated in **Fig 5A.** The full protein data matrix, ***X*** consists of *m* samples for *p* proteins. All *p* proteins are used as covariates. The target gene expression matrix for a module, ***Y*** is an *m X n* matrix, each column representing the expression profile of a gene in the module. The regression weight matrix, ***W*** is a *p X n* matrix, each row representing the regression weight of a protein for all *n* genes. In the group LASSO framework, a pre-defined grouping structure of covariates is used to select or de-select a group of coefficients together. We define a group as the set of regression weights for a single protein’s association to all module genes, resulting in *p* groups. The framework uses a mixed L1/L2-norm penalty to impose smoothness and sparsity: the number of proteins (groups) with any nonzero regression weights should be small, and the weights within a group should be similar.

We compared the performance of the MTG-LASSO models to models learned from randomized protein data (using one-sample z-tests; **Materials and Methods**). About half of the human modules, and all but two mouse modules, were predicted better than chance for multiple λ values. This result was consistent for both RMSE and Pearson correlation measures of predictive quality. From this analysis, we concluded that the protein data does indeed contain predictive signal for many modules; however, it should be used conservatively as predictive quality is not equally good across modules.

The alternative to our MTG-LASSO approach is to perform traditional LASSO for each gene independently, ignoring the module structure. To assess the advantage of MTG-LASSO over regular LASSO, we applied both methods to each module separately. We compared the methods on the basis of (i) sparsity of the model measured by the average number of regulators chosen for a module across the 10 folds, and (ii) prediction quality measured by Pearson's correlation and the root mean square error (RMSE) between the measured and predicted set using 10-fold cross-validation. Both MTG-LASSO and LASSO have a regularization term, λ, which controls the tradeoff between the model complexity penalty and a model's predictive power. We examined five settings of λ, between 0.99 (highest penalty imposed, requiring few regulators) and 0.01 (least penalty imposed, allowing many regulators). The regularization term λ is a number between 0 and 1. It denotes a fraction of λ_max_ (maximum possible regularization before reaching a 0 solution), and is comparable between MTG-LASSO and LASSO (see **Materials and Methods**).

First, we observe that LASSO identified more regulators per module at every value of λ compared to MTG-LASSO (**Fig 5B, S4A Fig**). Second, when matched at the same λ, MTG-LASSO and LASSO yielded highly similar predictive quality scores per module based on both Pearson correlation and RMSE values (**Fig 5C, S4B and S4C Fig**). The comparable predictive power of MTG-LASSO is observed over all modules and values of λ compared (two-sided Kolmogorov-Smirnov test: human *p*-value = 1.0 Pearson, 0.98 RMSE; mouse *p*-value = 1.0 Pearson, 0.58 RMSE).

The proteins selected by MTG-LASSO were contained within and important to the LASSO models. In particular, when the LASSO proteins were ranked by their average absolute outgoing weight for the same λ, the MTG-LASSO regulators appeared at the top of the list. We quantified the ranking by the area under ROC curve (AUROC), treating MTG-LASSO as the positive class and the additional LASSO regulators as the negative class. The AUROC gives the probability that a randomly chosen MTG-LASSO regulator ranks above a randomly chosen LASSO regulator. For human, AUROC ranged between 0.96–0.99, and for mouse 0.91–0.98 when considering all genes together. The high ranking of MTG-LASSO regulators in the LASSO selected regulators is observed on a per-module level as well (**S4D and S4E Fig**).

Because MTG-LASSO learned a subset of the LASSO regulators, we asked if the regulators that were identified by LASSO but not MTG-LASSO were known to be important based on existing siRNA screening studies. Focusing on modules that were predicted better than random (11 in human and 36 in mouse), we defined consensus regulators per-module as regulators that were selected in at least 6 of 10 folds of cross-validation after determining a module-specific value of λ (**Materials and Methods**, **Fig 5D** and **5E**). Our analysis identified a total of 17 human consensus protein regulators for 11 modules in human (1–6 protein regulators per module, **Table 3;** selected regulators shown in **Fig 3A**) and a total of 33 consensus protein regulators predicted for 36 modules in mouse (1–6 regulators per module, **Table 4, S8 Table**). A similar analysis for LASSO identified 60 different regulators for human and 79 for mouse, which contained the MTG-LASSO regulators entirely (**S9 Table**). Comparable proportions of the consensus regulators identified by each method have been found as hits in published influenza screening studies (for human, MTG-LASSO 23.5% and 18% LASSO; for mouse, MTG-LASSO 18% and LASSO 20%). Because the MTG-LASSO regulators were included in the LASSO rankings we also computed the precision after excluding the MTG-LASSO regulators. In human the precision was markedly lower (16.3%), and slightly lower in mouse (19.6%). Thus for the human cell line data, there is a distinct advantage of using MTG-LASSO to quickly identify the important regulators. The mouse data is more challenging likely due to the heterogeneous nature of the cell populations. Together, these results suggests that MTG-LASSO is able to learn as good a predictive model as regular LASSO, and is particularly advantageous for identifying regulators at the module level. Below we further experimentally validate the MTG-LASSO regulators.

**Table 3:** Consensus human protein regulators identified by MTG-LASSO.

Category	Gene (Protein name/family)	Module(s)
**Splicing**	PRPF31^*B*^ (Pre-mRNA processing)	1487
**Apoptosis**	MAGED2 (Melanoma antigen)	1549
	THBS1 (Thrombospondin)	1434, 1472, 1482, 1485, 1502, 1540, 1543, 1549, 1596
**Inflammation**	APP^*O*^ (Amyloid beta precursor)	1484, 1501, 1549
	SERPINA3 (Serpin peptidase inhibitor)	1484
**Known or potential antiviral**	ISG15^*K*,*S*^ (Interferon-induced)	1484, 1501, 1549
	DDX50 (ATP-dependent RNA helicase)	1482, 1487
**Intracellular transport**	EHD4 (EH-domain containing)	1482
	STMN4 (Stathmin)	1472, 1540
**Cell membrane**	COLGALT1 (Collagen beta(1–0) galactosyltransferase)	1549
	ITGB4^*S*^ (Integrin)	1484
	PMM2 (Phosphomannomutase)	1487
**Mitochondrion**	IARS2 (Isoleucil-tRNA synthetase)	1543
	NFS1 (Cystein desulfurase)	1484
	YME1L (ATP-ase)	1487
**Other**	HIST1H1B (Histone protein)	1472, 1540
	KLHL33 (Kelch-like family)	1549

Genes are categorized by major biological annotation according to NCBI Entrez, GeneCards, or UniProt. The Module column lists the MTG-LASSO predicted target modules of a protein regulator. Genes that have been identified as relevant to influenza by a screening or literature study are marked with a superscript: Brass et al. 2009^B^; Karlas et al. 2010^K^, König et al. 2010^O^; Shapira et al. 2009^S^.

**Table 4:** Top consensus mouse protein regulators identified by MTG-LASSO.

Category	Gene (Protein name/family)	Module(s)
**Influenza life cycle**	Sumo2^*O*^ (Ubiquitin-like modifier)	3135, 3147, 3280
**Immune response**	C3 (Complement protein)	2810, 2975, 3208,3210
	Serpina3k (Serpin peptidase inhibitor)	3029, 3206
	Serpina3m (Serpin peptidase inhibitor)	3029, 3159, 3181, 3184, 3186, 3192, 3206, 3207
	S100-A8	3141, 3154
**Hemolysis**	Hp (Haptoglobin)	3207, 3210
	Hpx (Hemopexin)	2899, 2976, 3159, 3184, 3192, 3198, 3207
**Mitochondrion**	Letmd1 (LETM1-domain containing)	2950, 3047, 3072, 3135, 3139, 3179, 3187
**Splicing**	Snrpf^*K*^ (Spliceosome component)	3056, 3134, 3135, 3139, 3181, 3193
**Other**	Fgb (Fibrinogen bet-chain)	2810, 2977, 3056, 3156
	Hopx (HOP homeobox)	3154, 3280
	2310036022Rik^*W*^ (C19orf43 homolog)	3070, 3159, 3199, 3206, 3249

Genes are categorized by major biological annotation according to NCBI Entrez, GeneCards, or UniProt. The Module column lists the MTG-LASSO predicted target modules of a protein regulator. Because there were 33 total, we limit presentation here to twelve regulators that were linked to at least two modules. A full list of consensus proteins is available in **S8 Table**. Genes for which human homologs have been identified as relevant to influenza by a screening or literature study are marked with a superscript: Karlas et al. 2010^*K*^; König et al.2010^*O*^; Watanabe et al. 2014^*W*^.

### MTG-LASSO predicts protein-level regulators of host response with significant effects on virus replication

We first qualitatively examined our regulators based on known literature and annotation of these regulators. Several of our MTG-LASSO protein regulators are known to be associated with immune response pathways, cellular membranes and intracellular transport, RNA splicing machinery, mitochondrial inflammation, and may be involved in viral replication, entry or transport within the cell **(Tables 3 and 4)**. We experimentally validated seven of the 17 human MTG-LASSO regulators for which we already had siRNA libraries **(Table 5, S7 Table)**, transfecting cells with 4 siRNAs per regulator. For all seven regulators at least two of the four siRNAs resulted in a significant change in virus titer, and had at least one siRNA resulted in a fold-change far beyond 10-fold. Unlike in the mRNA case, where most of the significant and high magnitude changes were in one direction, for several of the protein regulators, different siRNAs targeting the same gene resulted in both significant and high magnitude increase and decrease in viral replication. We therefore used a majority rule on the significant changes to phenotypically characterize the effect of knockdown of a particular protein regulation. Specifically, a gene was called “pro” viral replication if the number of significant decreases in viral replication was greater than the number of significant increases. Similarly, a gene was called “anti” viral replication if knocking it down resulted in more significant increases than decreases in viral replication. Among the seven proteins that were tested, four were classified as “pro” viral replication with more significant decreases in viral replication (HIST1HB, ISG15, PRPF31, THBS1), while three were called as “anti” viral replication with significant increase in viral replication (APP, ITGB4, SERPINA3). PRPF31, which had the strongest, consistent effect across all significant siRNAs, is a pre-mRNA splicing factor that was previously implicated in viral replication by a genome-wide screen. It is known that the host RNA splicing machinery is hijacked by influenza virus [34]. ISG15 is a ubiquitin-like protein that is stimulated by interferon alpha and beta and is associated with diverse cellular functions including cell-to-cell signaling and anti-viral activity. Hence, ISG15’s pro-viral phenotype was surprising. However, this protein has been observed to be attached to both host and viral proteins [35] and has been previously shown to reduce viral replication on knockdown in a previous study [36]. THBS1 is a ligand of CD47 and an inhibitor of T and dendritic cells and may play a role in inhibiting inflammation [37]. SERPINA3 and APP in particular are interesting candidates as potential inhibitors of viral replication. The serine protease inhibitor SERPINA3 (module 1484) and its mouse homologs Serpina3k (two modules) and Serpina3m (eight modules) were identified independently for both species. SERPINA3 belongs to the family of serine protease inhibitors that have diverse roles in innate immunity [38–40]. While SERPINA3 has not been shown to affect viral replication, another member of this family, SERPINE1, was shown to reduce the infectivity of the virus particle [41]. APP has been mostly studied for its role in neurodegenerative diseases: beta amyloid plaques are associated with neuronal cell death in Alzheimer’s Disease [42] but a recent study also suggests it may have an antiviral role, based on observations of decreased influenza A replication (H1N1 and H3N2) in cells treated with beta amyloid [43], consistent with the increase in viral titer observed in this study.

**Table 5:** Summary of results of siRNA validation study of seven human protein regulators predicted by MTG-LASSO.

Regulator	Majority viral phenotype
APP	Anti
HIST1H1B	Pro
ISG15	Pro
ITGB4	Anti
PRPF31	Pro
SERPINA3	Anti
THBS1	Pro

Four siRNAs were used per gene. All genes had multiple siRNAs with significant effects, and all had at least one at least ten-fold. However, for most genes, different siRNAs resulted in different effects on virus titer; therefore we characterize each by its majority viral phenotype among the significant siRNAs. Full data and results are available in **S7 Table**.

Taken together, our results indicate that MTG-LASSO can identify regulator candidates that are relevant to immune responses to viral infections. Overall MTG-LASSO's ability to highly rank the most relevant regulators is an important factor for tractable downstream interpretation and validation of selected regulators.

### Active network components across time points and virus treatments

We next used the integrated mRNA and protein-based networks to examine the temporal dynamics of host response. This type of analysis can be used to predict which network components may be repressed or differentially wired in response to different viruses. We built *time point-specific active networks* by overlaying time point-specific expression on the integrated regulatory network connecting both mRNA and protein regulators to their target genes. For each time point and virus, we obtained a time point-specific regulatory network by including inferred edges between regulator nodes (mRNA or proteins) and target genes (mRNA) that were significantly up-regulated (z-score ≥ 2, compared to all expression values at that time point). For this analysis we focused on the Calu-3 data set.

Examination of the active network size (number of edges, regulators and targets) over time showed that the size of networks for each viral treatment tended to change by adding more edges over time (**S5 Fig**), with the H5N1 mutants and wild-type achieving the largest networks. Interestingly, H1N1 NL's network grew more slowly than that of H1N1 CA04 (**S5 Fig**), although the networks for the two viruses were highly similar in terms of which nodes and edges they contained by the last time point (**S6 Fig**).

**Fig 6:**
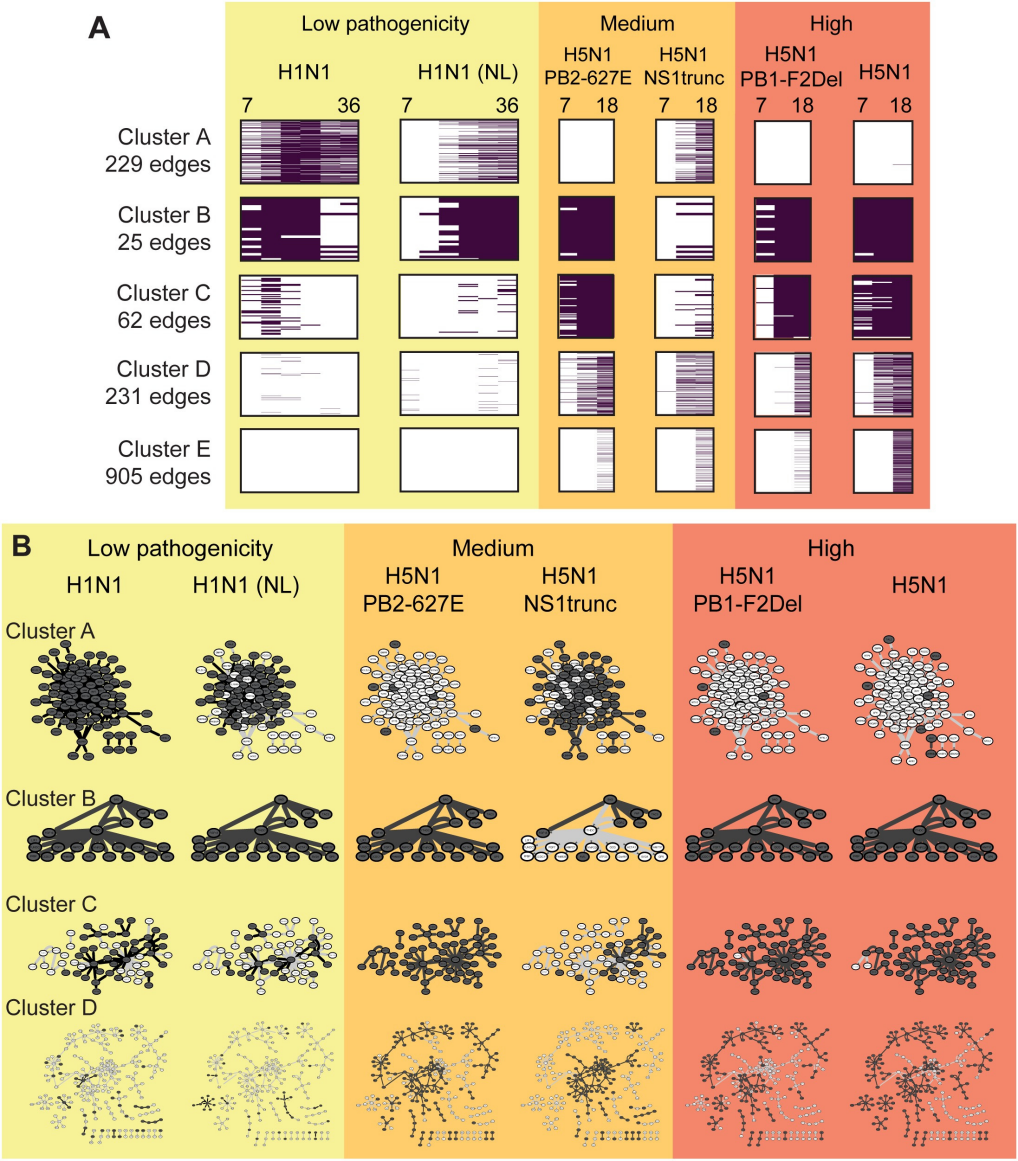
Time-point and virus-specific active subnetwork clusters. **A.** Clusters identified using hierarchical clustering of edges based on the presence/absence pattern of edges across time and virus strain. Each row represents an edge. All clusters are uniformly scaled to the same height, but comprise varying numbers of edges (shown on left). **B.** Active subnetworks defined by clusters in **A** for each virus. The subnetwork for each virus is defined by taking the union of all edges present at any time point for that virus. Dark nodes/edges are active in both the cluster and any time-point for the virus; light nodes/edges are active in all other viruses and part of this cluster.

To identify network components that were common to a subset of viruses or time-points, we clustered the edges from the active networks according to their presence-absence pattern across all samples (**Materials and Methods**, **Fig 6**). We obtained five clusters of edges (**Fig 6A**) four of which included edges active at multiple early time points (**Fig 6A; Clusters A-D**) and a fifth containing edges that were only present in the 18-hour (very late) time points for medium or high-pathogenicity viruses (**Fig 6A; Cluster E**). Further, Clusters A-C exhibited a sustained pattern of edge presence with edges present through the entire time course, while Cluster D was associated with edges present during the later time points.

Clustering was strongly driven by presence-absence pattern of edges in viruses rather than individual time points. Edges from the two low-pathogenicity H1N1 strains (CA04 and NL) tended to be placed in the same cluster (**Fig 6**, Clusters A, B), and edges from the medium-pathogenicity (PB2-627E) and two high-pathogenicity strains (PB1-F2Del, H5N1 wildtype) did as well (Cluster B, C, D). The networks for the medium and high pathogenicity mutants additionally distinguished activation responses that occurred at later time points (Cluster A, D) from those that were present at all time points (Clusters B, C).

To identify specific processes that were associated with these edge clusters, we tested them for enrichment of pathways (**Table 6, Materials and Methods**). Cluster A was significantly enriched with immune response processes, namely anti-viral and interferon response. This cluster was active for H1N1 infections as well as H5N1 NS1trunc. Predicted regulators in this cluster included key immune response regulators at the mRNA (e.g. IRF7, STAT1, TRIM21, NMI) and the protein level (ISG15p). In terms of pathogenicity, the NS1trunc virus is more lethal than the H1N1 strains; however, truncated NS1 protein disrupts the virus' ability to effectively suppress host immune response. The H5N1 NS1trunc mutant in particular displayed an interesting clustering pattern across the other clusters. It was represented in two clusters that represent late response to other medium- and high-pathogenicity viruses (Clusters D, E), but absent from a cluster that represents early-onset sustained edges among H5N1 strains (Cluster C) as well as from Cluster B, which included edges from all other viral strains. Cluster B and Cluster C, which are associated with the two highly pathogenic viruses are associated with GPCR signaling and muscle contraction and include some of our siRNA based regulators (YTHDC1, Cluster B) and (HCLS1, HOXA7, Cluster C). Both Clusters D and E, which include active edges primarily from the high and medium pathogenicity viruses, are associated with signaling pathways including Wnt signaling and the circadian clock. Wnt signaling has been implicated in influenza infections [14,44], while disruptions in circadian clocks have been shown to increase susceptibility of the immune system to infections [45].

**Table 6:** Characterization of time- and virus-specific subnetworks.

Cluster	Timing	In-cluster viruses	Out-of-cluster viruses	Enriched pathways	Enriched regulators
**A**	Sustained	H1N1 CA04 and NL; H5N1 NS1trunc	H5N1 WT, PB1-F2del, PB2-627E	JAK-STAT signaling*, antiviral and interferon response, many immune-response pathways, cell cycle	FOSL1, IRF7, ISG15p, LMO2, MAFF, MLKL, NFS1p, NMI, NUPR1, SP110, SSTR2, STAT1, TRIM21, TRIM5
**B**	Sustained	H1N1 CA04 and NL; H5N1 WT, PB1-F2del, PB2-627E	NS1trunc	GPCR signaling	SAG, YTHDC1
**C**	Sustained	H5N1 WT, PB1-F2del, PB2-627E	H1N1 CA04 and NL; H5N1 NS1trunc	Muscle contraction	DDR2, HCLS1, HOXA7, SAG, TAL2, YTHDC1
**D**	Mid-late	H5N1 WT, NS1trunc, PB1-F2del, PB2-627E	H1N1 CA04 and NL	PPARA*, SCF KIT*, NFG*, FGFR* mutant signaling pathways	CCRN4L, CDKL2, CDKL3, CITED2, ETV3, MXD1, NR4A2, PAPOLG, PPP1R10, SNAI1, TCEB1, TOB1, WNK4
**E**	Very late	H5N1 WT, NS1trunc, PB1-F2del, PB2-627E	H1N1 CA04 and NL	Circadian clock, Wnt signaling	ACVR1B, ERCC2, FBXL19, FOXH1, NKX2-5, PPP1R11, SAP30BP, TLX2

"Timing" describes the general timing under which the cluster is active; a value of 'sustained means that the cluster appears active throughout every time course. "In-cluster" lists the viruses represented in the cluster. 'Out-of-cluster' lists the viruses mostly absent from the cluster. "Enriched pathways" lists major enriched pathway terms

* indicates that the pathway was enriched only among regulators of the cluster network. "Enriched regulators" are the subset of cluster regulators for which their targets are statistically enriched in the cluster relative to all clusters.

In summary, our active network analysis identifies different subnetwork components that are specific to different viruses, and implicates additional processes that might be relevant in a strain-specific manner.

### Physical subnetworks suggest putative mechanistic connections between module regulators

**Fig 7:**
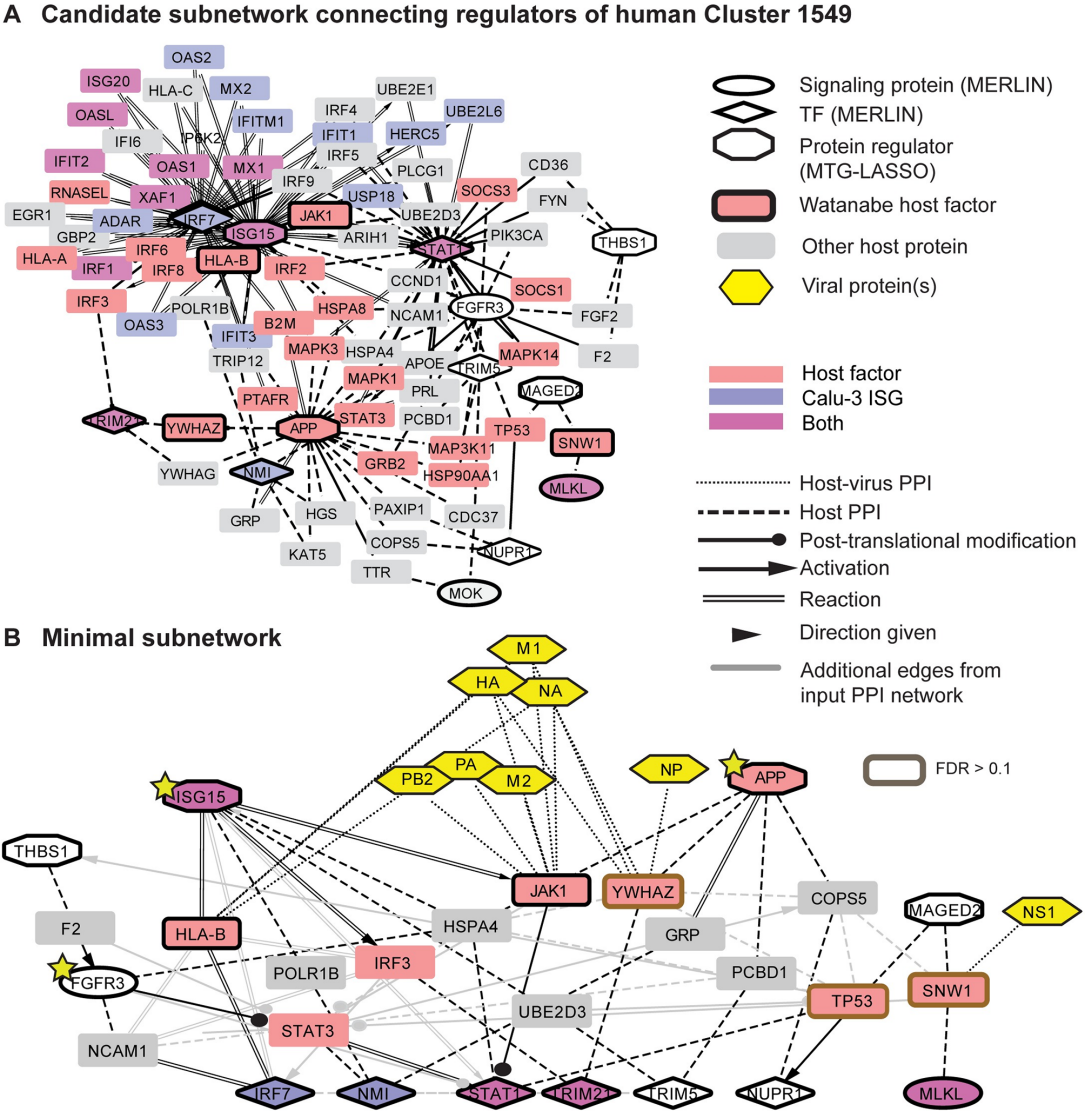
Integration of expression- and protein-based regulators into a physical subnetwork for module 1549. **A.** Candidate subnetwork for module 1549 given as input to the Integer Linear Programming (ILP) approach to find a minimal network. The candidate subnetwork is extracted from the background interaction network by connecting MERLIN (mRNA) and MTG-LASSO (protein) regulators through at most one intermediate node and mRNA signaling proteins to TFs (**Materials and Methods**). Nodes with dark outlines are input to the ILP-based subnetwork inference method: MERLIN regulators (diamonds and ellipses), protein regulators (octagons), and host genes for influenza identified by Watanabe et al (2014) (boxes). Nodes without borders are candidate intermediates between two regulators and were not given special treatment by the method. Node color indicates host genes involved in influenza obtained from siRNA or protein interaction studies and Calu-3 ISGs (as in **Fig 3**); this information is for visualization purposes and is not used in the subnetwork inference method. Only edges between intermediate nodes and input nodes are shown. Additional available protein-protein interactions between intermediate nodes are not shown for visual clarity. **B.** Minimal subnetwork for Cluster 1549's regulators inferred using ILP. Yellow stars indicate host genes for which siRNA knockdown significantly impacted viral replication. Also shown are interactions between Watanabe host genes and viral proteins (yellow hexagons). Grey edges are input to the method but not selected by the approach. All selected edges and all nodes other than YWHAZ, TP53, SNW1 (brown boxes) had FDR ≤ 0.10. FDR was assessed based on permutation tests.

Our analyses thus far inferred two sets of regulators for each module: (1) mRNA-based regulators, signaling proteins and TFs, predicted by MERLIN based on transcriptome changes, and (2) protein-based regulators predicted by MTG-LASSO based on proteome and transcriptome changes. Both these types of regulators were predicted based on patterns of co-variation of regulators and targets (at the individual gene or module levels). What is missing is information about the underlying physical mechanisms that connect the signaling proteins to transcription factors, and the protein-level regulators to the mRNA-level regulators. To gain insight into the underlying physical mechanisms that connect the regulators, and to link them to host genes identified from functional screening studies, we integrated the predicted regulators with physical protein-protein interactions, transcription factor-target interactions, and metabolic reactions from multiple public databases (**Materials and Methods**). Typically, the interaction data do not provide evidence for direct interactions between regulators, requiring the identification of additional intermediate nodes that connect these regulators. However, even allowing only one intermediate node can result in large subnetworks that are unwieldy for interpretation (see for example **Fig 7A**). Furthermore, because available interactions are not condition-specific, they may contain false positive interactions and may be missing relevant connections. To identify high-confidence physical subnetworks that are easily interpretable, we formulated a constrained optimization problem to find directed paths that connect MERLIN-identified signaling proteins to transcription factors and MTG-LASSO-identified proteins to MERLIN regulators using a minimal set of intermediate nodes (**Materials and Methods**), prioritizing the inclusion of host genes that were recently identified by Watanabe et al. [46] using both RNAi and co-immunoprecipitation with viral proteins. Such nodes are important in the virus-specific protein-protein interaction network and provide additional context for interpreting our predicted transcriptome and proteome-based regulators. We applied an Integer Linear Programming (ILP) approach to solve this optimization problem, which has been shown to find more precise solutions compared to heuristic algorithms [47–49].

Using our ILP approach we identified high-confidence physical subnetworks for 16 human modules, which included between 0–8 intermediate nodes, including a subset of the Watanabe hits and protein interaction partners (**S2 Text**, **S1 Table, Supporting Website**). Nine of the module subnetworks included predicted protein regulators. The high-confidence subnetworks were able to connect more true regulators with fewer intermediate nodes than subnetworks inferred from random regulators with the same degree distribution, suggesting that the sparsity of the high-confidence physical subnetworks was not merely due to the degree of the input regulators in the physical interaction network (**S3 Text**). We also used the random input subnetworks to compute an empirical FDR for each protein by measuring the frequency at which the protein appears randomly, and found that it ranged from 0–0.4, demonstrating that many of the proteins used in the subnetworks are unlikely to be identified by chance (**Materials and Methods**). A low FDR is a conservative measure of a protein's importance, as many relevant proteins are network hubs and are likely to be identified by chance.

These modules, together with their subnetworks, provide an integrated view of different types of regulators that are interacting to drive the downstream expression pattern of the host response. We discuss several below and provide all others on a **Supporting Website.**

### Core integrated regulatory module networks represent an interplay of interferon signaling, inflammation and apoptosis components of mammalian immune response

**Fig 8:**
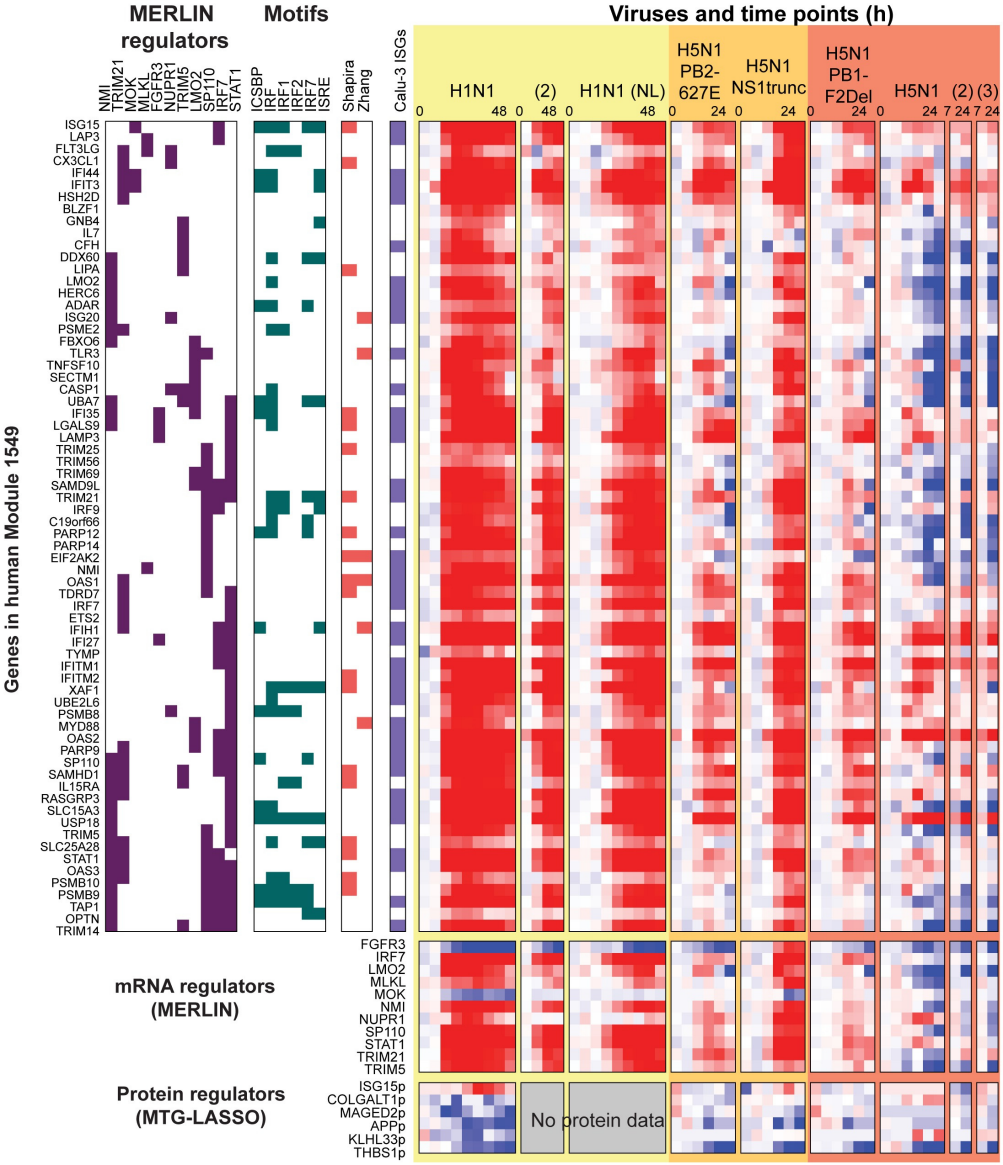
Target and regulator expression profile of human Module 1549. In the “**Regs**” columns, consensus regulators for each gene are marked with purple boxes. In “**Motifs**”, genes containing MSigDB regulatory motifs, including miRNA motifs, are marked in green. Motifs shown are only those that are enriched in the module. Next, colored boxes indicate host genes identified from screening studies (salmon) and immune response gene sets (violet). Below the rows for module genes are rows for MERLIN-predicted mRNA regulators and MTGLASSO-predicted protein regulators. Gene expression values are scaled (-2,2); protein values are scaled (-1,1) to improve visibility. Time courses entirely missing at the protein level are indicated as “No protein data”.

Our integrated network analysis identified several modules that captured different components of the host immune response machinery. One of these modules was Module 1549, which was associated with immune response-related processes by many of our computational analyses (**Figs 7 and 8**). Module 1549 exhibited a particularly interesting strain-specific pattern of induced expression under infection with low-pathogenicity viruses and the medium pathogenicity virus, H5N1-NS1trunc, and repressed expression under infection with high pathogenicity viruses (**Fig 8**). Module 1549 is also enriched for genes associated with interferon signaling, which are critical for mounting the innate immune response. The influenza NS1 ('non-structural') protein is already known to inhibit the host's antiviral type I interferon response [50], suggesting that this module would be a good candidate for further investigation into the mechanism of action of NS1. Furthermore, the genes in Module 1549 overlapped significantly with two mouse modules (**Fig 4A**), and its regulators featured prominently in the conserved regulatory network identified by the intersection of the human and mouse consensus networks (**Fig 4B).** This module is associated with NFS1, APP, SERPINA3, and ITGBP protein regulators, and IRF7, NMI, and STAT1 mRNA regulators, which are well-known members of the interferon response and JAK-STAT antiviral response pathway [27,28]. The subnetwork analysis applied to the regulators of this module highlighted HSPA4, also known as Hsp70, as a hub that connects gene members from multiple immune response pathways **(Fig 7B)**. HSPA4 has been proposed as both an antiviral factor [51] as well as a chaperone required for viral replication [52]. HSPA4's direct subnetwork connections include members and modulators of the antiviral JAK-STAT pathway (ISG15[53], FGFR3, STAT1) and also inflammation (APP). FGFR3 was a hit in our MERLIN-network based prioritization siRNA study and acts as a modulator of the JAK-STAT pathway in growth disorders such as achondroplasia (OMIM). APP was identified as an inhibitor of viral replication in our siRNA validation. Other important genes in this subnetwork are the protein regulator THBS1 and an mRNA-based regulator, MLKL. THBS1 is involved in apoptosis [54] and potentially inhibiting inflammation [37], and our siRNA validation results classify it as a pro-viral replication gene, while MLKL induces necroptosis (inflammatory cell death; [55]). As a whole, the subnetwork for Module 1549 ties together multiple immune response pathways, namely, antiviral interferon signaling, inflammation and apoptosis.

**Fig 9:**
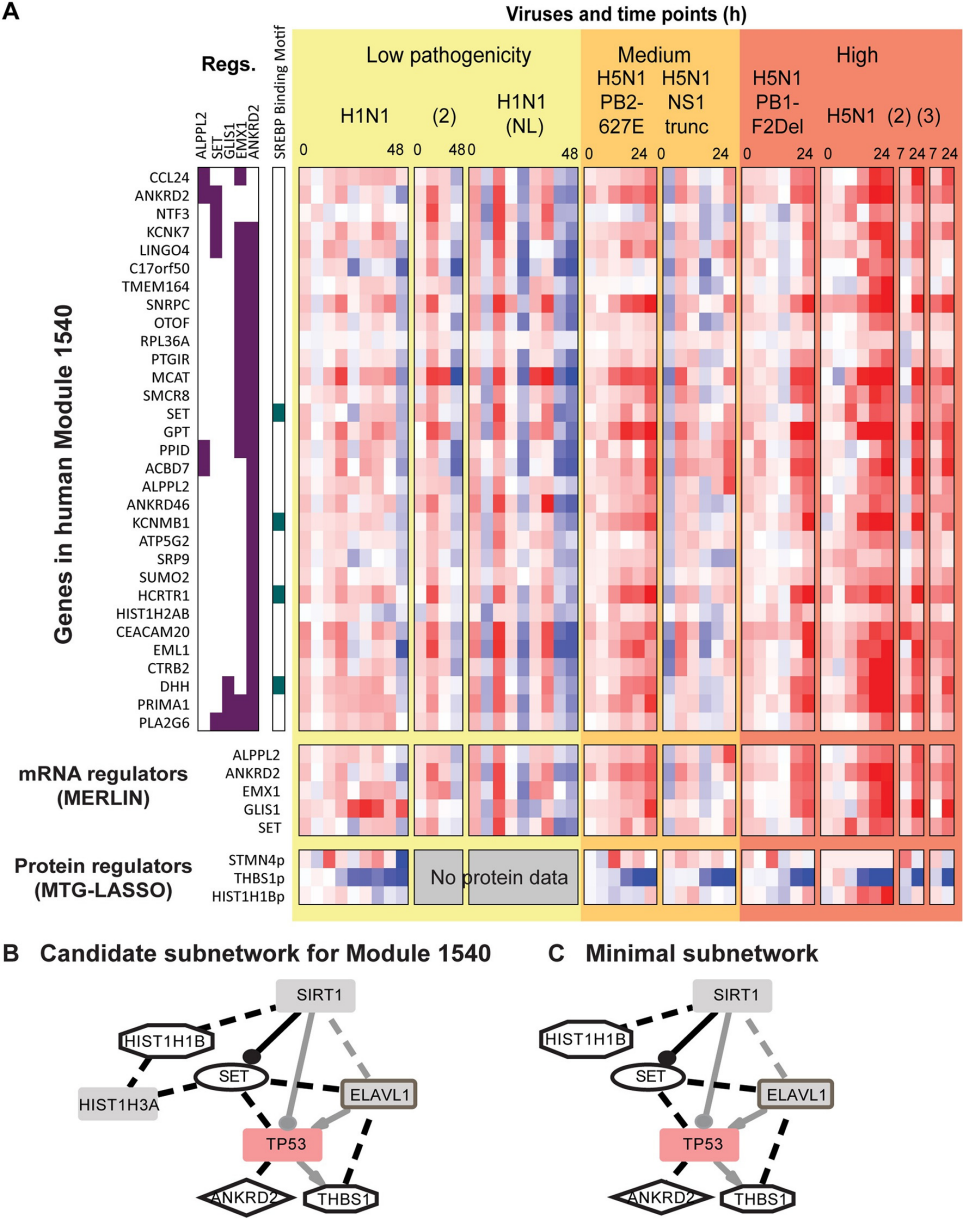
Target and regulator expression profile and the physical regulatory program of Module 1540. **A.** Heatmap visualization of module 1540 genes and regulators. In the “**Regs**” columns, consensus MERLIN regulators for each gene are marked with purple boxes. Next, genes with SREBP binding motif are marked in green. Below the heatmap for module genes are rows for MERLIN-predicted mRNA regulators and MTGLASSO-predicted protein regulators. Gene expression values are scaled (-2,2); protein values are scaled (-1,1) to improve visibility. **B.** Original subnetwork for Cluster 1540. Black edges link regulators to intermediates; these are provided as input to the ILP method. Additional interactions from the background network between regulators and intermediates are shown in grey. See **Fig 7B** for additional legend details. **C.** Minimal subnetwork for Cluster 1540, output from the ILP-based subnetwork inference method. Compared to the original subnetwork, the minimal subnetwork prunes away one protein: HIST1H3A. See **Fig 7B** for legend.

In contrast to Module 1549, Module 1540 (**Fig 9A**) shows a pattern of repressed expression in response to low-pathogenicity viruses, and increased expression over time in response to high-pathogenicity viruses. The subnetwork analysis revealed connections between the mRNA (ANKDRD2, SET) and protein-based regulators (HIST1HB1, THBS1) of this module via intermediate nodes, SIRT1 and ELAVL1 and the Watanabe host gene, TP53 (**Fig 9B**). The SIRT family of proteins have been identified as antiviral factors for multiple viruses [56]. The subnetwork suggests that part of its antiviral activity is mediated through interactions with anti-apoptotic signaling protein SET [57] as well as through direct interactions with histones. The other proteins in the subnetwork have roles in apoptosis and the p53 pathway: TP53 (p53) itself, ELAVL1 (which stabilizes p53 mRNA; [57]), ANKRD2 [58], and THBS1. In summary, Module 1540 identified interactions between histone proteins and various pro- and anti-apoptotic factors, some of which may explain the independently observed antiviral activity of SIRT1.

A third characteristic pattern was exhibited by Module 1472 (**S7 Fig**). Genes in this module were associated with a general pattern of induced expression but differed in the intensity of induction (stronger induction in the high pathogenicity strains compared to the low pathogenicity strains). This module was predicted to be regulated by several transcription factors (ANKRD2, EMX1, EN2, FOXC2, SOX17, RLX2, ZNF205) and two signaling proteins, GRIN1 and SIK1. SIK1 is a protein kinase which is involved in phosphorylation of HDACs (histone deacetylases) which can in turn modulate innate anti-viral responses [59] and interferon signaling genes [60]. Moreover, the subnetwork analysis linked SIK1 to the predicted protein regulator THBS1 (an inflammation inhibitor) through the intermediate node FYN, a tyrosine kinase with potential role in NFKB-mediated adaptive immunity.

Finally, a fourth module of interest was Module 1482 (**S8 Fig**), which exhibited a pattern of high expression in the low pathogenicity viruses compared to both high and medium pathogenicity viruses. Both the candidate and minimal subnetworks for this module provided useful information for generating hypotheses about mechanistic interactions between the regulators. The module was associated with both mRNA and protein regulators as well as hits from the Watanabe study: SNW1, which interacts with viral NS1 protein, and PSMC1, which interacts with viral HA, M1, NA, PA, PB1, and PB2 proteins. The subnetwork analysis connects predicted mRNA regulators (SRC, BTRC, PPM1F) and protein-based regulators (THBS1, EHD4) through SNW1 and PSMC1 (**S8C Fig**). EHD4 is a regulator of endocytosis [61], suggesting a role in virus entry, and SRC is a tyrosine kinase that has been identified to be involved in antiviral signaling [62].

Taken together, our integrated analysis identified the major regulatory modules of host transcriptional response and predicted mechanistic regulatory programs associated with these modules. The modules exhibited pathogenicity or strain-specific patterns and were enriched in immune related processes and predicted to be regulated by genes from diverse pathways including innate immune response and apoptosis. These module case studies support our regulators as important players of host response and provide an integrated view of how host response may be regulated at multiple levels, from mRNA, to protein, to interaction networks.

## Discussion

Identification of the molecular networks that underlie host response to different pathogenic infections is important to understand both the mechanisms of immune response as well as to design better therapeutics. Towards this end, we performed an integrative regulatory network-based analysis that combines transcriptomic, proteomic and existing molecular interaction datasets to identify important genes, modules and subnetworks. We found that the key components of innate immune response processes namely, interferon production and signaling, and important regulators of immune response (STAT1, NMI, IRF7), were conserved between a human cell-line (*in vitro*) and mouse lung (*in vivo*) model system. Our approach was able to predict novel regulators at the mRNA and protein level and implicate molecular pathways that may drive virus-specific host responses.

Prioritization of predictions, including important genes, interactions and networks, is important in systems biology studies, which can easily generate a large number of hypotheses. While a large number of prioritization methods, including network-based strategies, have been proposed [63] and computationally validated, relatively few have been used to inform experimental validation in a medium to high-throughput manner [21,64]. Towards this end we used our inferred MERLIN networks to prioritize important regulators of host response and test 20 regulators using siRNA. Six of the 20 regulators exhibited highly significant and consistent effects on viral replication and included 4 novel mRNA regulators (BOLA1, HOXA1, HCLS1, FGFR3) in addition to IRAK3 and YTHDC1, which were identified by genome-wide studies using a different influenza virus [65,66]. Mice lacking IRAK3 have an increased mortality rate compared to wild type in influenza-induced pneumonia [67]. BOLA1 is a mitochondrial protein, which helps maintain mitochondrial morphology and oxidative stress [68]. Mitochondria provide important innate immune functions, including cellular response to double-stranded viral DNA by induction of cytokines through the MAVS (mitochondrion antiviral signaling) protein [69]. Influenza A virus proteins have also been shown to translocate to mitochondrial membranes and promote mitochondrial fragmentation [70]. HCLS1, which is a hematopoetic lineage specific protein [71], is also interesting due to its role in signal transduction pathways in B and T cells [72,73]. HOXA7 is a member of the homeobox family of transcription factors, known to have critical roles in differentiation and embryonic development. The HOXA7 gene has to our knowledge not been associated with specific immune related functions, however, a closely related gene, HOXA9 was shown to be involved in lymphoid and B cell development, which are important cell types of the immune system [74]. The HOXA7 gene was also shown to code an antigen in specific tumor types [75] and could be involved in differentiation programs of immune cell types in response to influenza infections. Finally, FGFR3, a fibroblast growth factor receptor gene is known to have diverse roles in multiple cellular functions [76–78]. The FGFR1-4 genes were investigated for their role in influenza A viruses [79]. FGFR1 was shown to significantly impact cellular internalization of two influenza A viruses but FGFR3 was not expressed in the cell line tested, leaving open the possibility of its potential role in the influenza life cycle. To our knowledge, these regulators have no previously known role in influenza response, but serve as promising leads for further in-depth validation studies using *in vivo* models.

While approaches to infer and examine networks from mRNA are routinely used in systems biology studies of complex responses [2,6,80], examining proteomic datasets and especially integrating them with transcriptomic data to gain insight into regulatory mechanisms is an open challenge that has been addressed by relatively few approaches [7,49,81]. This is because, unlike mRNA levels, proteomic technologies are still maturing and datasets have lower genome coverage and higher frequency of missing values [82]. To tackle this challenge we introduced a novel structured sparsity inducing approach, Multi-task Group LASSO (MTG-LASSO), which enabled us to leverage the overall signature of expression at the level of modules. Our experiments confirmed that while the MTG-LASSO and LASSO achieved comparable prediction performance on held-aside data, the MTG-LASSO approach identified a sparser set of regulators per module. Proteins with the strongest contributions to module expression prediction were involved in innate immune response pathways, RNA splicing, membrane organization and transport, that are relevant to different parts of virus life cycle. Experimental validation of these regulators further associated pro or anti-viral replication functions with them. Both directions are interesting from the point of view of understanding the mechanisms of immune response as well as for designing vaccines that could disrupt viral replication and growth. PRPF31, which is involved in RNA splicing, was particularly notable as an example of a pro-viral replication regulator, given the emerging role of post-transcriptional process in diverse pathogenic infections including bacterial pathogens [83]. SERPINA3 (anti-viral, serine protease inhibitor) is also interesting as a candidate anti-viral drug target due to the known roles of serpins in innate immunity [38–40]. We also predict a pro-viral role of ISG15, an interferon stimulated protein and that is involved in diverse processes including anti-viral activity. Importantly, several predictive protein regulators were not identified as differentially expressed at the mRNA level (7/17 for human; 22/33 for mouse; **S4 Table, S5 Table**), emphasizing the importance of measuring multiple types of cellular components.

These results were further bolstered with our subnetwork analysis that combined the mRNA and protein regulators through physical interactions (identified as interaction partners in the physical subnetwork). Even though no information about known relevant pathways was provided as input, our approach was able to give interaction-driven predictions for how these different regulators are coordinated. A notable example was a newly identified gene, HSPA4, in module 1549’s regulatory program, that connected FGFR3 (discussed above), STAT1 (a member of the JAK-STAT signaling pathway), and inflammation and cell death pathways (represented by THBS1 and APP). Without the subnetwork analysis, it would not be possible to identify HSPA4, as it was not part of the input set of mRNA and protein regulators.

Our approach identified several important modules that exhibit strain and pathogenic specific patterns of expression. In particular, Module 1549, which was associated with interferon signaling and interferon-stimulated genes, exhibited a striking pattern of differential expression of repression in the wild-type H5N1 virus. Our results are consistent with those of [84], who observed a differential pattern of expression of the ISG genes and showed that the differential expression of these genes were inherently tied to host response. Another module, Module 1540 exhibited an opposite pattern of expression and is associated with cell-cell signaling. One caveat to the validation was that we tested the siRNAs only in pandemic H1N1 and therefore we do not know how impact of these regulators in different virulent strains. The physical regulatory programs together with the expression phenotype associated with these modules enables us to make intriguing hypothesis of potential mechanisms by which upstream regulatory networks drive context-specific expression, which could be followed by further validation.

Some of the same mRNA time courses have been previously studied with computational network approaches [8,85], offering additional points of reference against which to compare our inferred multi-virus regulatory network. Mitchell et al. [8] prioritized influenza host genes from wild-type H1N1 and H5N1 samples. They learned a correlational network from which they identified central nodes as well as modules. They also used a regularized regression approach (Inferelator, [24]) to identify sparse regulator sets that predict the average module expression. Our top predicted regulators (both protein and mRNA) intersected with theirs on only one gene (NMI). The limited overlap may not be surprising as the bulk of our prioritized regulators were restricted to transcription factors and signaling proteins; however, similar gene families were present in our list and theirs (including DDX, HOX, and ISG genes). Additionally, McDermott et al. [85] previously analyzed the H5N1 mRNA time courses using a similar approach: first identifying modules through hierarchical clustering, followed by identification of regulators for the modules' average expression. That study identified a set of conserved clusters across human, mouse, and macaque, and a list of prioritized regulators. We found significant overlap of several of those clusters with several our modules (**S9 Fig**), but no overlap in the presented prioritized regulators. However, both our study and theirs identified shared functional processes (cytokine signaling and production, inflammation, apoptosis, and cell cycle regulation) and gene families (IRF genes).

Our work can be extended in several ways. One limitation of the current subnetwork approach is that the protein-protein interactions employed are not necessarily functionally relevant to the tissues or conditions under study; a future direction of work is to integrate tissue-specific interactions at this step, such as from large-scale computationally-inferred compendia [86,87]. Another direction of future work is to jointly learn modules and their physical regulatory programs using an iterative framework while integrating proteomic measurements. We anticipate that as systems biology studies expand to more viruses, host systems and diseases, approaches such as ours are going to be increasingly useful to characterize host responses at multiple omic levels, prioritize genes and subnetworks for validation. The outcomes from such studies will be important to assemble a comprehensive picture of the mechanisms responsible for healthy and disease states, and ultimately guide the design of effective therapeutics.

## Materials and Methods

### mRNA dataset and processing for MERLIN analysis

We obtained background corrected and between-arrays quantile normalized host mRNA response data from multiple strains and dosages of influenza virus in Calu-3 human cells (GEO Accessions GSE28166, GSE37571, GSE40844, GSE40844, GSE43203, GSE43204) and 20-week old C57BL/6 mice (GSE33263, GSE37569, GSE37572, GSE43301, GSE43302, GSE44441, GSE44445) (full details, [4]). All experiments were performed using Agilent microarrays. In the original work, each array was subject to quality control, background correction, and quantile normalization. We directly used the processed data available.

The viruses include three wild-type and four mutant strains. Wild-type strains include A/California/04/2009 (H1N1), A/Netherlands/602/09 (H1N1), and A/Vietnam/1203/2004 (H5N1). The mutant strains of H5N1 each affect different aspects of the virus life cycle [1]. HAavir, used in the mouse experiments only, has restricted tissue tropism due to a mutated cleavage site in the hemagglutinin glycoprotein. The wild-type H5N1 virus has a lysine at position 627 in the PB2 polymerase protein that is associated with the adaptation of H5N1 viruses to mammals. Mutation of this amino acid to glutamic acid (PB2-627E) reduces polymerase activity in mammalian cells and pathogenicity in mice. NS1trunc has a shortened version of the NS1 protein, thereby interfering with the virus' ability to suppress host antiviral responses through the RIG-1 pathway. PB1-F2del is missing viral protein PB1-F2, which is involved in many aspects of virus pathogenicity, including polymerase activity and host immune regulation.

For each virus infection in human cell line, six or nine time points were collected, spanning 48 hours for low-pathogenicity viruses (at 0, 3, 7, 12, 18, 24, 30, 36, 48 hours) and 24 for medium and high (at 0, 3, 7, 12, 18, 24 hours). Each time point had at least three biological replicates. An additional four-time-point replicate series was collected for H1N1 (hours 0, 12, 24, 48) and two additional two-point series were collected for H5N1 (hours 7, 24). In the mouse system, multiple dosages were available for some viral treatments. All time points were taken at days 1, 2, 4, and 7 after infection, with the exception of the highest dosage of H5N1, which omitted day 7 due to complete lethality. We collapsed replicates of each time point using the median value of a gene’s expression level. In total, after collapsing replicates, there were 50 samples per human gene and 51 per mouse gene.

Using MLD_50_ values [1], we classified the viruses into *high*, *medium* and *low* pathogenicity groups: high pathogenicity included WT H5N1 and H5N1-PB1-F2del; medium pathogenicity included H5N1-NS1-trunc and H5N1-PB2-627E, and low pathogenicity included H5N1-HA-avir and H1N1. Instead of HAavir, the Calu-3 data included a different strain of wild-type H1N1 (A/Netherlands/602/09, or NL); both viruses have the same low level of pathogenicity. Having viruses exhibiting similar extents of pathogenicity enabled us to perform a systematic comparison of host response divergence under the same type of perturbation.

Because our focus was to compare findings between *in vivo* (mouse) and *in vitro* (human cell lines) we started with genes that were conserved (had orthologs) between human and mouse, and exhibited differential patterns of expression between high, medium and low pathogenicities. We first obtained the relative expression value of a gene to the same gene's expression in an untreated mock sample from the same time point. We included a gene if its relative expression profiles compared to mock in either species were significantly different between any two pathogenicity groups (assessed by t-test, *p*-value<0.01 for human and *p*-value<0.05 for mouse). The resulting gene set comprised 7,192 genes in the human cell line and 7,240 genes in mouse lung. As regulators, we selected transcription factor and signaling proteins [5,57,88] that were present in the differentially expressed gene set. This included a total of 1,396 encoded candidate regulators in human and 1,394 candidate regulators in mouse.

### Protein data processing for MTG-LASSO analysis

We used protein level data that was available for a subset of the same samples as the mRNA data from [1,3,4,8,89]. Briefly, peptide-level abundances were obtained by liquid chromatography—mass spectrometry (LC-MS) and matched to protein levels following normalization and quality control.

For human, the H1N1 NL time course and a short replicate time course of H1N1 CA04 were missing (see gray boxes in protein regulator heatmap at the bottom of **Figs 8, 9, S7A and S8A**); for mouse, the missing samples spanned H5N1 HAavir and two of four dosages of H1N1 CA04. These data provided 37 unique samples for human and 42 for mouse. We selected proteins with fewer than 50% missing values (resulting in 3,026 for human and 1,908 for mice), and imputed remaining missing values using the mean value for existing samples (within the same time course). To prepare data for input into the MTG-LASSO method, we normalized both protein and mRNA data by their row means.

### Learning consensus MERLIN regulatory module networks for human and mouse using stability selection

We used MERLIN [22], a network inference algorithm, to learn regulatory module networks for the human and mouse datasets separately. The input of MERLIN is a matrix of gene expression data and a list of candidate regulators (e.g., transcription factors and signaling proteins); the output is a regulatory network and a set of regulatory modules. This dual output is a unique feature of MERLIN compared to other network inference methods. It uses an iterative procedure that alternates between learning the network structure using a greedy search for regulators of individual targets (giving a regulatory program per gene) and performing hierarchical clustering on the target genes based on both co-expression and the similarity in their current assigned regulator sets. The module assignments are also used in the network learning step to provide a prior preference for adding regulators to a gene's regulatory program if the regulator is already assigned to another gene in the module.

We embedded MERLIN in a stability selection framework [90], in which MERLIN networks are learned independently for 40 random sub-samples of the expression data. Each subsample consisted of about 90% of the total samples (45/50 for human, 45/51 for mouse). The resulting ensemble of networks provides a confidence value for each edge in the regulatory network, thereby enabling the identification of a robust consensus regulatory module network.

For each individual network, we set MERLIN's three parameters according to recommendations from the original publication based on simulated data [22], setting *p* = -5, *r* = 4, *h* = 0.6. The parameter *p* controls regulatory network sparsity (more negative values, fewer edges), *r* controls network modularity (higher values, stronger preference for sharing regulators in a module), and *h* specifies a threshold on the distance used to cut a hierarchical clustering into gene modules (lower values, more modules). We derived a consensus regulatory module network in several steps from the ensemble of networks that were produced under stability selection. First, to derive a consensus network of regulator-target edges, we applied a threshold of 0.3 confidence to the confidence-weighted regulator-target edges produced by stability selection. This threshold was picked based on its FDR, assessed by comparing to a random consensus regulatory network generated by running the approach on 40 randomizations of the expression data. We calculated FDR as the ratio of the fraction of edges from the random network that would be accepted at the threshold, over the fraction of edges from the true network that were accepted by the threshold. The FDR of human and mouse networks were 0.30 and 0.17, respectively.

Next, to independently derive co-expressed, co-regulatory modules, we began by hierarchically clustering the genes, defining the similarity between any pair of genes as the frequency at which the two genes were clustered together across the 40 separate module assignments. We then applied a distance threshold of 0.5 on this new clustering to define consensus modules. Finally, we identified consensus regulators for each module (at the module level) by assessing the significance of overlap of each regulator's consensus targets within each consensus module, as measured by the hypergeometric test (FDR < 0.05).

### Enrichment analysis of MERLIN modules

We evaluated the modules based on their enrichment with various sources of gene sets and pathways. We call each gene set or pathway an *annotation category*, and performed enrichment testing independently on groups of annotation categories coming from the same source.

To assess significance of the enrichment of the modules with an annotation category, we computed a hypergeometric *p*-value specifying the probability of observing *k* or more genes from a module with *n* genes to have an annotation *a*, given that there are a total of *M* genes with annotation *a* among a total of *N* genes. Considering together all of the annotation categories for a module, we applied the Benjamini-Hochberg procedure to control FDR at 0.05 and accepted corrected *p*-values < 0.05.

The groups of annotation categories that we tested include Gene Ontology Biological Process [91], targets of transcription factors from MSigDB [92–94] as well as those determined by scanning the promoters of genes using known motifs in the JASPAR database [95] with FIMO [96]. We additionally included gene sets available from MSigDB, Reactome [97], BioCarta (http://www.biocarta.com) and KEGG [98]. In addition to the above general curated pathways, we also used experimental, literature-based, and manually curated gene sets that were specifically associated with influenza and with innate immune response. We assembled hit sets from a group of RNAi and protein-protein interaction screens [14,36,46,65,66,99–103]. We also created an immune response group of annotation categories consisting of Calu-3 interferon stimulated genes [84], curated targets of the NF-kB transcription factor (http://www.bu.edu/nf-kb/gene-resources/target-genes), genes differentially expressed in response to inflammatory interleukins IL-1 or IL-6 [104] and members of curated immune response pathways from InnateDB [105], downloaded November 2014). For the influenza and immune response gene sets, we obtained human-mouse gene orthologs from the Mouse Genome Database [106].

### Comparison of MERLIN network to other experimentally and computationally inferred networks

We used a hypergeometric test and a fold enrichment to assess the significance of the overlap in edges between the MERLIN consensus regulatory network and other immune response and transcriptional regulatory networks described in **Fig 3B** (MSigDB motifs,[92] Mouse pathogen (Amit) [5], Mouse Th17 (Yosef) [21]**)**. We refer to the MERLIN network as the "query" network, and the other network as the "test" network. Because the networks were directed, we first defined the shared universe of regulators and the universe of targets as the intersections of the respective node sets from the two networks. Then, we defined the shared universe of edges as all possible edges between regulators and targets. The size of the universe, *u*, is calculated as the product of the numbers of regulators and targets in the universe. We measure overlap, *o*, as the number of edges in the universe that are common to both query and test. We measure the size of the query and test networks, *q* and *t* respectively, as the number of edges in each network restricted to the shared universe. To test significance of the size of the overlap, we use the hypergeometric distribution to assess the probability of identifying *o* or more overlapping edges in a random draw of *q* edges from a universe of size *u* that contains *t* test edges. Fold enrichment is defined as the ratio of observed to expected fraction of edge overlap between the two networks, or *(o/q)/(t/u)*.

### Identification of strain and pathogen-specific expression patterns in MERLIN modules

A module was considered to exhibit an interesting strain or pathogenicity-specific pattern of expression (module catalogs, **S1 Table, S2 Table)** if the mean expression of genes in that module was significantly higher or lower for any two pairs of conditions. Conditions were defined based on high *vs* low, high *vs* medium, and low *vs* high pathogenicities. In addition, we considered those modules that exhibited different patterns of expression between the two wild-type viruses H1N1 (CA04) and H5N1 (VN1203). For significant expression we used a t-test *p*-value *<* 0.01 for human and 0.05 for mouse. We relaxed the threshold for mouse lung because the data represents transcriptional response from a more heterogeneous collection of cells as compared to the human cell line; we also omitted day 7 from all mouse data due to lack of data (due to lethality) for the high pathogenicity viruses. To classify the modules based on expression differences between different pathogenicities, we used an additional criteria of selecting modules whose mean expression in one pathogenicity type was different in *sign* compared to mean expression in the second pathogenicity type.

### Identification of protein regulators using Multi-Task Group LASSO (MTG-LASSO)

We implemented MTG-LASSO using the mtLeastR function available as part of the Sparse Learning with Efficient Projections package for MATLAB (SLEP 4.1; [104]). The objective function for MTG-LASSO is defined as

$$\underset{W}{\min}\frac{1}{2}{\left|\left|XW-Y\right|\right|}_{2}^{2}-\lambda {\left|\left|W\right|\right|}_{\frac{l_{1}}{l_{2}}}$$

The first term in the objective function is the least squares loss obtained by the difference between the observed gene expression data matrix, ***Y***, and the predicted values from the product of the protein data ***X*** and learned regression weight matrix ***W*** (**Fig 5A**). The second term is the Group LASSO norm penalty on the complexity of the weight matrix. This norm penalizes the number of groups (according to the one-norm) and encourages smoothness among the weights within each group (according to the Euclidean two-norm). The parameter λ controls the trade-off between loss and the regularization term.

We evaluated the MTG-LASSO and LASSO methods over a range of λ, expressed as a fraction of its maximum possible value λ_max_, above which the coefficient vector will be forced to zero. The SLEP package calculates λ_max_ for each model as follows. For MTG-LASSO, λ_max_ is the largest two-norm of rows in ***X'Y***, where ***X'*** is the matrix of protein data (transposed from **Fig 5A**, now with proteins/groups on rows, samples on columns) and ***Y*** is the matrix of mRNA data for one module (samples on rows, genes/tasks on columns). For LASSO, λ_max_ is the largest absolute element in the vector ***X'y***, where ***X'*** is the protein data and ***y*** is the expression vector for one gene. We varied λ between several values from its minimum (0.01, almost no sparsity imposed) to its maximum (0.99, significant sparsity imposed). The complete set of tested values were {0.01, 0.10, 0.25, 0.50, 0.75, 0.99}. Because λ is normalized by the λ_max_ it represents a comparable regularization strength between both regression techniques.

We obtained predicted mRNA values for all protein-matched, mRNA samples of all module genes using a 10-fold cross-validation approach, where a consecutive set of about 10% of samples were held aside from each fold (comprising most of a time course). For the very largest modules (modules 1592, 1594 in human), MTG-LASSO was computationally intractable, and therefore we could not identify any regulators.

### High-confidence protein regulators for individual modules

To select regulators for each module, we first chose a setting of λ for each module based on λ-correlation curves that plotted correlation of the predicted values and the true data against λ for each module (**Fig 5D and 5E, S1 Dataset**). Surprisingly, we observed that the curve did not have the same shape for each module. Among human modules, we found three categories of modules based on these curves. The first consisted of 10 modules for which correlation is roughly constant across all values of λ; that is, all predictive performance on the held-aside data was entirely due to a very small set of regulators. The second consisted of another 10 modules for which performance improved as MTG-LASSO was allowed to use more regulators (as sparsity decreased). A final third category contained those modules that could not be predicted more accurately than random for more than one setting of λ, usually the highest or lowest tested value (all remaining modules).

In contrast to the curves for the human modules, many mouse modules yielded λ-correlation curves that showed a visible inflection point, with high correlation before a particular λ and low correlation after. We grouped the modules based on a visual determination of the inflection point. Only two mouse modules were not predicted more accurately than random for multiple values of λ (based on either RMSE or Pearson).

We only considered λ values for which accuracy was significantly greater than random based on z-tests described in the next section. Plots for Pearson correlation are shown for example human and mouse modules in **Fig 5D and 5E**, with stars indicating the chosen values. All curves are available in **S1 Dataset**. For human modules, we chose λ = 0.75 for modules with constant correlation (such as Module 1596, **Fig 5D**), and λ = 0.10 for modules with correlation that decreased as λ increased (such as Module 1549, **Fig 5D**). For the mouse modules, the curves were not so obviously matched into 'constant' and 'decreasing' categories. We chose based on the visible inflection point in the curve, preferring the next higher (sparser) λ if the drop in correlation was not dramatic.

After choosing λ, we defined consensus regulators using both frequency in cross-validation for the specific λ value and the magnitude of regression weights. First, we considered regulators that received nonzero regression weight in at least 6 of the 10 folds. Next, we applied a threshold on average absolute regression weights (across all genes, across folds with nonzero weight), followed by a Bonferroni-corrected significance z-test (p-value<0.05) to assess whether the same protein would be given a weight above that threshold by chance. We used a $\bar{x}$ = 0.20 for human regulators and $\bar{x}$ = 0.10 for mouse regulators, choices that resulted in approximately 1–6 regulators per module. Mean and standard deviation for the z-test were estimated from the random regression weights. See **S10 Table, S11 Table** for frequencies of chosen regulators across folds.

### z-test to assess significance of mRNA prediction quality

We evaluated the significance of MTG-LASSO and LASSO predictive quality using one-sample z-tests [107]. To assess module-level predictive quality, we obtained one statistic, $\bar{x}$ (Pearson's correlation, RMSE), for a module from all predictions from 10 folds of cross validation. We then assembled a null distribution of statistics by running the method on *N* = 40 random permutations of protein data and real module gene expression data, obtaining the null mean μ_0_ and variance σ_0_. We calculated the Z-score for the statistic as $\frac{\bar{x}-{µ}_{0}}{\sigma_{0}/\sqrt{N}}$ and obtained a one-sided *p*-value from the normal distribution.

### Prioritization of predicted MERLIN regulators

We developed a regulator prioritization score that is based on the loss in predictive power of the consensus regulatory network under *in silico* perturbations. For each regulator *r*, we held aside the regulator from the consensus regulatory network *N*, creating a "lesioned" network, *N-{r}*. We then re-learned regulator-target regression weights for the lesioned network using five-fold cross-validation. We use this network to score each regulator according to the average increase in prediction error when that regulator is removed from each of its targets' regulatory programs:

$$Score(r)=\frac{1}{\left|targets(r,N)\right|}\sum_{t\in targets(r,N)}\;e_{t}^{N-\{r\}}-e_{t}^{N}$$

where *targets(r*,*N)* is the set of *r*'s target genes as predicted by the consensus regulatory network *N*, $e_{t}^{N}$ is the mean squared prediction error for the expression profile of gene *t* using network *N* (obtained by cross-validation), and $e_{t}^{N-\{r\}}$ is the mean squared prediction error for the same gene *t* given by the lesioned regulatory network *N-{r}*.

### Selection criteria for MERLIN network-based prioritization regulators for siRNA validation

We tested 20 regulators from MERLIN prioritization using siRNA. These regulators were selected based on their rankings in human and were additionally informed by their rankings in mouse. Nineteen of these regulators were in the top 40 for human. An additional candidate, FIG4 (rank 42) was added because it was ranked in the top 100 of the mouse regulator list (96). Other regulators that were in the top 100 of mouse included well-studied regulators (IRF7, NMI, STAT1) and were already in the top 40 of human rankings.

### Experimental validation of predicted regulators by siRNA knockdown

For siRNA transfections, human lung epithelial cells (A549) were seeded into 24-well plates (8x10^4^ cells/well) and allowed to settle for 2 hours before transfection with 10 nM siRNA (final concentration) and 1 μl of lipofectamine RNAiMax reagent (Invitrogen). For each candidate regulator a gene-specific package of four preselected siRNAs were used (FlexiTube Genesolution siRNA, Qiagen) **(S7 Table**). The following siRNAs were used as controls: a cell death inducing blend of siRNAs (AllStars Hs Cell Death, catalog number 04381048, Qiagen) for visual confirmation of efficient siRNA delivery, a validated nontargeting siRNA (AllStars Negative Control, cataglog number 1027281, Qiagen) as a negative control and a previously described siRNA targeting influenza virus NP mRNA (NP-1496; synthesized by Qiagen) as a positive control. Each siRNA was evaluated in triplicate. Cells were incubated for 48 h before infection with 500 plaque forming units of A/Oklahoma/vir09-1117003813/2009 (pandemic H1N1) per well. Supernatants were collected from each well 48 h post infection and viral titers were determined by plaque assay in Madin-Darby Canine Kidney epithelial (MDCK) cells.

Results for each siRNA were statistically assessed separately. First, we log-transformed the virus titers obtained by suppressing the expression of the candidate genes using siRNAs. Next, we compared the replicates of each candidate siRNA to the replicates of the negative control (All-star siRNA), using one sided, unpaired T-tests. The *p*-values were not adjusted because the number of candidates was small and by adjusting the *p*-values we would likely lose true positives [106]. Finally, we calculated the fold-change and the log-fold change for each siRNA candidate compared to the negative control, and used these two measures (significance, fold-change) to identify hits that significantly changed the virus titers in the cells.

### Time point and virus-specific regulatory components

To identify coarser temporal and virus-specific patterns among the active regulatory subnetworks derived from each sample, we clustered the edges according to the samples in which they were active. In order to focus on early stage immune response rather than late-stage cell death responses, we held aside the 18-hour time point from the medium and high-pathogenicity virus treatments (H5N1 and all mutants), placing them in their own cluster (Cluster E, **Fig 6**). We performed average-linking hierarchical clustering using Manhattan distance, specifying the number of clusters *k*. We performed clustering for *k =* 3,4,5,6,7, and inspected the resulting clustering by eye and by silhouette index. While *k* = 3 gave the highest silhouette index (which decreased with *k*), we chose *k* = 4 because it made a distinction between a sustained, nearly pan-virus cluster (Cluster B, **Fig 6**) and a sustained medium/high pathogenicity cluster (Cluster C, **Fig 6**).

We used hypergeometric test-based enrichment (0.05 FDR corrected p-value < 0.05) to interpret the clusters using annotated pathways from MSigDB [92–94], Reactome [97], BioCarta (http://www.biocarta.com) and KEGG [98]; results are summarized in **Table 6,** "Enriched pathways". We also used a hypergeometric test to identify whether any regulators were cluster-specific, rather than common to all clusters (**Table 6,** "Enriched Regulators"). First, we took the union of the sample-specific subnetworks and identified the union target set of every regulator. For each of those regulators, we tested whether any cluster subnetwork was enriched for the targets of the regulator relative to the union (FDR corrected p-value < 0.05).

### Identification of physical subnetworks to connect module regulators

To integrate the signaling proteins, transcription factors and protein regulators predicted for each MERLIN module, we used an integer linear programming-based (ILP) method for extracting subnetworks from a background network, similar to previous work [47–49]. The ILPs were modeled using GAMS modeling system v. 24.0.1 and the ILOG CPLEX solver v. 12.4.0. We applied this method separately to each human module.

The subnetworks that are extracted by this approach are composed of paths through a background physical interaction network (provenance described below). We searched for two kinds of paths: (i) paths that begin with MERLIN signaling proteins and terminate in MERLIN TFs (sinks) that share at least three targets in common, and (ii) paths that begin with MTG-LASSO-based protein regulators (sources) and terminate in any MERLIN regulator (signaling protein or TF, sinks). For all paths, we allowed only one intermediate node between the source and sink. For several modules, the sources and sinks were not sufficiently close (or represented) in the background network to allow for the generation of any paths. We also excluded the largest two modules (1592 and 1594), which had a large number of regulators, and only a few regulators could be included in short paths.

For some modules, the union of candidate paths resulted in small and visually interpretable subnetworks, as in **Fig 9**. However, for most modules, the resulting subnetwork was not visually interpretable (as in **Fig 7**). Our ILP-based method can identify high-confidence interpretable subnetworks for all modules that had paths, regardless of size.

The ILP-based approach (described in detail in **S2 Text**) finds an ensemble of connecting subnetworks and assigns confidence values to paths according to how important they are for connecting the regulators using a small number of additional nodes. We extract a subnetwork that connects all regulators by solving an integer linear program in which instructions for how paths may be chosen are expressed as linear constraints, and the objective function optimizes a global property of the subnetwork. Our constraints require the inclusion of reachable predicted regulators, and specify that each protein-protein interaction may only be used in one direction within the subnetwork. In the objective function, we encoded a preference that the subnetwork should use the influenza host genes from Watanabe et al. [46] as intermediates whenever possible, and to otherwise minimize the use of intermediate nodes. Because many subnetworks may satisfy these constraints, we combine multiple solutions to the ILP into an ensemble. We score each path by the fraction of solutions that contain that path. We defined a high confidence subnetwork as the paths that received at least 0.75 confidence over the ensemble.

To estimate the false discovery rate of nodes and edges by this approach, we generated a null distribution of subnetworks by running the method on randomized input data. Randomization was performed as follows. We first replaced the consensus MERLIN regulators with randomly drawn TFs and signaling proteins from the set that was provided as input to MERLIN, maintaining the degree distribution (in the background network) of the entire group. We also drew random replacements for the protein regulators from the input set of proteins. Then, for each module, we mapped the original pairs of MERLIN sources and sinks, and protein sources and MERLIN sinks, to their randomized counterparts, searched for paths to connect them, and assigned confidence values using the ILP approach as we did for the true predicted regulators.

We performed 40 randomizations, and calculated the FDR of a protein or an edge at a particular confidence level as the fraction of the random subnetworks that included it in a path of that confidence level.

### Background network for physical subnetworks

We assembled a human background network composed of protein-protein, protein-DNA, and metabolic interactions from the STRING database v9.1 ([108]; excluding interactions labeled as 'expression'), high-confidence interactions from HIPPIE ([109]; downloaded September 2014, using high-confidence threshold of 0.73 as recommended on the website), low-throughput physical interactions from BioGRID ([110]; downloaded September 2014), and a human kinase-substrate network [111]. Each resulting background network consists of both directed (e.g., post-translational modifications such as phosphorylation and ubiquitination) and undirected (e.g., binding) interactions. We then removed all interactions involving ubiquitin (UBB, UBC, UBD), SUMO (SUMO1-4), and 11 additional ubiquitin fusion proteins. These proteins are used as post-translational modifiers and are recorded as binding partners for large proportions of proteins in the background network. The ubiquitin and SUMO systems are still represented in the background network in the form of directed ubiquitination and sumoylation events between ligases and substrates.

### Network visualization

Networks in figures were developed using Cytoscape [112] and supporting website visualizations were developed with Cytoscape.js (http://cytoscape.github.io/cytoscape.js)

### Supporting website

We provide the inferred modules with their mRNA-based regulators, protein-based regulators, and physical subnetworks (for human only) as a navigable resource at http://pages.discovery.wisc.edu/~sroy/integrative_influenza/.

### Availability

Code for MTG-LASSO and physical subnetwork identification is available from our repository at https://bitbucket.org/roygroup/integrative_networks

## Supporting Information

S1 Table Characterization of human modules. (XLSX)

S2 Table Characterization of mouse modules. (XLSX)

S3 Table Enrichment comparison of MERLIN and GMM modules. (PDF)

S4 Table Comparison of human host response network genes identified by expression and protein levels. (PDF)

S5 Table Comparison of mouse host response network genes identified by expression and protein levels. (PDF)

S6 Table Prioritized MERLIN regulators. (XLSX)

S7 Table Results of siRNA validation studies, including siRNA catalog numbers. (XLSX)

S8 Table Full list of mouse protein regulators predicted by MTG-LASSO. (PDF)

S9 Table Consensus protein regulators predicted by LASSO for human and mouse. (XLSX)

S10 Table Frequencies of human protein regulators across 10-fold cross validation. (PDF)

S11 Table Frequencies of mouse protein regulators across 10-fold cross validation. (PDF)

S1 Fig Overview of mouse influenza response modules identified by MERLIN. Expression patterns of 56 mouse modules with at least 10 genes. The red-blue heat map shows mean expression of all genes in each module for each sample, compared to mock treatment value (similar to **Fig 2**). Blocks of columns are time series (in days) from different viruses and dosages (PFU: Particle forming units). Viruses are ordered from low to high pathogenicity; different dosages of the same virus are placed next to each other. The “Genes” column shows the size of each module; larger values are shown in darker blue. Under “Enrichment”, a red box indicates module enrichment with any MSigDB motif, any curated gene set from Gene Ontology, KEGG, REACTOME or BioCarta (MSigDB curated gene sets), any influenza screen set, or any immune response gene set (described in **Materials and Methods**).
(PDF)

S2 Fig Comparison of gene prioritization schemes. Shown is the precision of predicted top *n* regulators for human (A) and mouse (B) host response identified using different ranking strategies. Precision is defined as the fraction of the top *n* genes that are known host genes identified from screening studies.
(PDF)

S3 Fig Visualization of shared genes measured by mRNA and protein. Human Calu-3 (**A-B**), mouse (**C-D**). Each column is a sample from one of the virus treatment time courses. Rows in each heatmap are genes in the intersection of the complete mRNA and protein data sets for one system after filtering out entries with >50% missing values; before filtering mRNA data set down to differentially expressed genes only. Genes are sorted by hierarchical clustering with average linkage, Manhattan distance followed by optimal leaf ordering of the mRNA data to enhance visualization of patterns. The ordering between mRNA and protein data is the same. Values are scaled between [–1,1] for all heatmaps.
(PDF)

S4 Fig Comparison of sparsity and predictive quality of MTG-LASSO and LASSO. Left column human; right column mouse. **A.** Counts of nonzero regression weights (Y-axis) identified at each level of λ (X-axis) for MTG-LASSO and LASSO for human (left) and mouse (right). The human data is the same as in main manuscript **Fig 5B**. **B.** Scatterplots comparing cross-validation Pearson correlation values for all modules, with one plot per value of λ, per species. In each scatterplot, there is one point per module. Inset ρ gives Pearson correlation between MTG-LASSO and LASSO per module Pearson correlations. Diagonal line is shown for comparison. **C.** Scatterplots comparing cross-validation RMSE values for all modules, with one plot for each value of λ, per species. In each scatterplot, there is one point per module. Inset ρ gives Pearson correlation between MTG-LASSO and LASSO RMSE values. Diagonal line is shown for comparison. **D/E.** Per-module ranking of MTG-LASSO-selected regulators according to LASSO absolute regression weight for human (**D**) and mouse (**E**). One AUROC value is obtained per module. Only modules for which MTG-LASSO predicted the module's expression better than random in at least five of six tested λ settings are shown.
(PDF)

S5 Fig Sizes of human Calu-3 virus-specific regulatory networks over time. Each trajectory represents the count of active network elements of a specific type (edges (**A**), regulators (**B**), targets (**C**)) at a time point for one virus.
(PDF)

S6 Fig **Comparison of Calu-3 active regulatory networks between all virus treatments, all time points, based on edges (A), regulators (B), and targets (C).** Cells are shaded according to 'precision' relative to the network on the row of the matrix. Precision is defined here as the size of the intersection (edges, regulators or targets) between the two networks divided by the size of the row network (edges, regulators or targets, respectively).
(PDF)

S7 Fig Integrated regulatory module network for Human Module 1472. **A.** Heatmap of module genes and regulators from mRNA and protein for module 1472. **B.** Input subnetwork, which is the same as the refined output subnetwork. Nodes and edges follow the same legend as **Fig 8**.
(PDF)

S8 Fig Integrated regulatory module network for Human Module 1482. **A.** Heatmap of module genes and regulators from mRNA and protein for module 1482. **B.** Original input subnetwork connecting module regulators. Black edges link regulators and intermediates. Additional interactions between grey nodes are other background network edges. Nodes and edges follow the same legend as **Fig 7**. **C.** High-confidence physical subnetwork.
(PDF)

S9 Fig Comparison of MERLIN modules to H5N1 Calu-3 clusters identified by McDermott et al. 2011 [85]. McDermott et al. identified modules using hierarchical clustering. Edges between modules represent fold-enrichment of the McDermott module's overlap with the MERLIN module relative to all genes in the intersection of the two sets of modules.
(PDF)

S1 Text Assessment of regulator prioritization schemes. (PDF)

S2 Text Integer linear program for high-confidence subnetwork inference. (PDF)

S3 Text Compactness of inferred subnetworks. (PDF)

S1 Dataset Plots showing MTG-LASSO predictive quality of each module vs. lambda, measured by Pearson correlation. One plot per module, both species.
(GZ) Click here for additional data file.
