# Supplementary material for: Integrating Transcriptomic and Proteomic Data Using Predictive Regulatory Network Models of Host Response to Pathogens
Source: PLoS Comput Biol. 2016 Jul 12;12(7):e1005013. doi: 10.1371/journal.pcbi.1005013 (PMC4942116; doi:10.1371/journal.pcbi.1005013)
Supplement: S3 Table — (PDF) [file pcbi.1005013.s003.pdf]

S3 Table: Enrichment of MERLIN and GMM modules with annotation categories. Annotation categories are described in the main text under Materials and Methods. The background set for enrichment analysis is defined as the intersection between the union of all modules and the genes annotated with any gene set. The 'Genes' column counts the number of genes involved in any enriched gene set. 'Avg. fold enrichment' is calculated as the fold enrichment of the maximally enriched term that a gene participates in, averaging over all genes in the 'Genes' column. 'No. gene sets' is the number of enriched gene sets for any module. Bold values indicate fold enrichment values for which MERLIN performed higher than GMM.

System	Category	Method	Genes	Avg. fold enrichment	No. gene sets
Human	Gene Ontology	GMM	2102	4.279	895
Human	Gene Ontology	MERLIN	1463	4.114	419
Human	Influenza screens	GMM	134	2.923	7
Human	Influenza screens	MERLIN	73	2.683	6
Human	Innate immune system	GMM	164	3.819	4
Human	Innate immune system	MERLIN	52	**9.444**	3
Human	Motifs	GMM	588	3.564	66
Human	Motifs	MERLIN	285	**5.700**	77
Human	Pathways	GMM	686	8.146	339
Human	Pathways	MERLIN	389	7.817	140
Mouse	Gene Ontology	GMM	1538	5.555	772
Mouse	Gene Ontology	MERLIN	1046	5.031	433
Mouse	Influenza screens	GMM	96	3.293	6
Mouse	Influenza screens	MERLIN	74	2.784	3
Mouse	Innate immune system	GMM	97	3.669	3
Mouse	Innate immune system	MERLIN	46	**5.753**	2
Mouse	Motifs	GMM	401	3.708	40
Mouse	Motifs	MERLIN	539	**4.248**	92
Mouse	Pathways	GMM	776	7.352	275
Mouse	Pathways	MERLIN	352	**9.997**	189
