# Supplementary material for: Integrating Transcriptomic and Proteomic Data Using Predictive Regulatory Network Models of Host Response to Pathogens
Source: PLoS Comput Biol. 2016 Jul 12;12(7):e1005013. doi: 10.1371/journal.pcbi.1005013 (PMC4942116; doi:10.1371/journal.pcbi.1005013)
Supplement: S4 Table — (PDF) [file pcbi.1005013.s004.pdf]

S4 Table: Comparison of human input data and inferred host response network regulators identified by expression and protein levels. Each cell gives the size of overlap between the two sets. Diagonal elements give the total number of elements in one set. 'Candidate mRNA target' gives the entire set of differentially expressed targets considered by MERLIN, which also includes the regulators. 'Candidate mRNA regulator' refers to the set of signaling proteins and transcription factors input to MERLIN. 'Candidate protein regulator' refers to the total set of proteins considered for MTG-LASSO. 'Consensus mRNA (protein) regulator' refers to regulators in the high-confidence sets derived from mRNA/protein levels.

	Candidate mRNA	Candidate mRNA regulator	Consensus mRNA regulator	Candidate protein	Consensus protein regulator
Candidate mRNA target	7192	1396	1250	1562	10
Candidate mRNA regulator		1396	1250	228	0
Consensus mRNA regulator			1250	217	0
Candidate protein regulator				3060	17
Consensus protein regulator					17
