# Supplementary material for: Integrating Transcriptomic and Proteomic Data Using Predictive Regulatory Network Models of Host Response to Pathogens
Source: PLoS Comput Biol. 2016 Jul 12;12(7):e1005013. doi: 10.1371/journal.pcbi.1005013 (PMC4942116; doi:10.1371/journal.pcbi.1005013)
Supplement: S5 Table — (PDF) [file pcbi.1005013.s005.pdf]

S5 Table: Comparison of mouse input data and inferred host response network genes identified by expression and protein levels. Each cell gives the size of overlap between the two sets. Diagonal elements give the total number of elements in one set. ‘Candidate mRNA target’ gives the entire set of differentially expressed targets considered by MERLIN, which also includes the regulators. ‘Candidate mRNA regulator’ refers to the set of signaling proteins and transcription factors input to MERLIN. ‘Candidate protein regulator’ refers to the total set of proteins considered for MTG-LASSO. ‘Candidate mRNA (protein) regulator’ refers to regulators in the high-confidence sets derived from mRNA/protein levels.

	Candidate mRNA target	Candidate mRNA regulator	Consensus mRNA regulator	Candidate protein regulator	Consensus protein regulator
Candidate mRNA	7240	1394	1252	938	13
Candidate mRNA regulator		1394	1252	113	1
Consensus mRNA regulator			1252	108	1
Candidate protein			1908	33	
Consensus protein regulator				33	
