# Supplementary material for: Integrating Transcriptomic and Proteomic Data Using Predictive Regulatory Network Models of Host Response to Pathogens
Source: PLoS Comput Biol. 2016 Jul 12;12(7):e1005013. doi: 10.1371/journal.pcbi.1005013 (PMC4942116; doi:10.1371/journal.pcbi.1005013)
Supplement: S8 Table — (PDF) [file pcbi.1005013.s008.pdf]

S8 Table: Full list of mouse protein regulators predicted by MTG-LASSO.

MTG-LASSO protein regulator	Module(s)	Human homolog identified by other influenza study
2310036O22Rik	3070, 3159, 3199, 3206, 3249	Watanabe *et al*, 2014 (46)
C3	2975, 3208, 3210, 2810	
Cpt2	3072	
Ctsb	3154	
Cyp2f2	3249	
Dlg1	3154	
Dtx3l	3056	
Eif4a3	2810	Karlas *et al*, 2010 (36); Shapira *et al*, 2009 (14)
Evpl	2977	
Fgb	2977, 3156, 2810, 3056	
Fgg	2977	
Gm2a	2977	
Hba-x	3179	
Hnrpdl	3193	
Hopx	3154, 3280	
Hp	3207, 3210	
Hpx	2899, 3184, 3192, 2976, 3159, 3198, 3207	
Letmd1	3135, 2950, 3047, 3072, 3139, 3179, 3187	
Nrgn	2899	
Ogfr	3062	
P01673	3135	
Pkn2	2977	
Ripk1	3058	
S100a8	3141, 3154	
Sdhb	3072	de Chassey *et al*, 2013 (102)
Serpina3k	3029, 3206	
Serpina3m	3159, 3206, 3029, 3192, 3207, 3181, 3184, 3186	
Sfxn3	3154	
Snrpf	3135, 3056, 3134, 3139, 3181, 3193	Karlas *et al*, 2010 (36)
Sumo2	3280, 3135, 3147	König *et al*, 2010 (65)
Tnks1bp1	3062	
Ttc33	3198	
Usp9x	3154	Watanabe *et al*, 2014 (46)
