# Supplementary material for: Integrating Transcriptomic and Proteomic Data Using Predictive Regulatory Network Models of Host Response to Pathogens
Source: PLoS Comput Biol. 2016 Jul 12;12(7):e1005013. doi: 10.1371/journal.pcbi.1005013 (PMC4942116; doi:10.1371/journal.pcbi.1005013)
Supplement: S10 Table — (PDF) [file pcbi.1005013.s010.pdf]

S10 Table: Frequencies of human protein regulators across 10-fold cross-validation.

Module	Regulator	Frequency
1434	THBS1	1.0
1472	HIST1H1B	0.8
1472	STMN4	0.9
1472	THBS1	1.0
1482	DDX50	0.9
1482	EHD4	1.0
1482	THBS1	1.0
1484	APP	1.0
1484	ISG15	0.9
1484	ITGB4	0.9
1484	NFS1	0.9
1484	SERPINA3	0.9
1485	THBS1	1.0
1487	DDX50	0.9
1487	PMM2	0.9
1487	PRPF31	0.9
1487	YME1L1	0.8
1501	APP	0.9
1501	ISG15	1.0
1502	THBS1	1.0
1540	HIST1H1B	0.9
1540	STMN4	0.9
1540	THBS1	1.0
1543	IARS2	0.9
1543	THBS1	1.0
1549	APP	1.0
1549	COLGALT1	0.8
1549	ISG15	1.0
1549	KLHL33	0.9
1549	MAGED2	0.9
1549	THBS1	1.0
1596	THBS1	1.0
