# Supplementary material for: Integrating Transcriptomic and Proteomic Data Using Predictive Regulatory Network Models of Host Response to Pathogens
Source: PLoS Comput Biol. 2016 Jul 12;12(7):e1005013. doi: 10.1371/journal.pcbi.1005013 (PMC4942116; doi:10.1371/journal.pcbi.1005013)
Supplement: S11 Table — (PDF) [file pcbi.1005013.s011.pdf]

S11 Table: Frequencies of mouse protein regulators across 10-fold cross-validation.

Module	Regulator	Frequency	Module	Regulator	Frequency
2810	Eif4a3	0.8	3154	Ctsb	0.8
2810	Fgb	1.0	3154	S100a8	1.0
2899	Hpx	0.9	3154	Usp9x	0.9
2899	Nrgn	1.0	3154	Sfxn3	0.9
2950	Letmd1	1.0	3154	Hopx	0.9
2975	C3	1.0	3154	Dlg1	0.9
2976	Hpx	1.0	3156	Fgb	0.9
2977	Gm2a	0.9	3159	Hpx	1.0
2977	Fgb	0.7	3159	Serpina3m	0.7
2977	Evpl	0.9	3159	2310036O22Rik	1.0
2977	Fgg	1.0	3179	Letmd1	1.0
2977	Pkn2	0.8	3179	Hba-x	1.0
3029	Serpina3m	0.9	3181	Serpina3m	1.0
3029	Serpina3k	0.9	3181	Snrpf	1.0
3047	Letmd1	1.0	3184	Hpx	0.9
3056	Snrpf	1.0	3184	Serpina3m	1.0
3056	Fgb	1.0	3186	Serpina3m	1.0
3056	Dtx3l	1.0	3187	Letmd1	1.0
3058	Ripk1	0.8	3192	Hpx	0.9
3062	Tnks1bp1	0.9	3192	Serpina3m	0.9
3062	Ogfr	1.0	3193	Snrpf	1.0
3070	2310036O22Rik	1.0	3193	Hnrpdl	1.0
3072	Cpt2	1.0	3198	Hpx	1.0
3072	Letmd1	1.0	3198	Ttc33	0.9
3072	Sdhb	0.9	3199	2310036O22Rik	1.0
3134	Snrpf	1.0	3206	Serpina3m	0.7
3135	Snrpf	0.9	3206	2310036O22Rik	1.0
3135	Letmd1	0.9	3206	Serpina3k	0.9
3135	P01673	1.0	3207	Hp	1.0
3135	Sumo2	1.0	3207	Hpx	1.0
3139	Letmd1	1.0	3207	Serpina3m	0.9
3139	Snrpf	1.0	3208	C3	1.0
3141	S100a8	1.0	3210	Hp	1.0
3147	Sumo2	1.0	3210	C3	1.0
3249	2310036O22Rik	1.0	3280	Sumo2	0.9
3249	Cyp2f2	0.7	3280	Hopx	1.0
