# Supplementary material for: Integrating Transcriptomic and Proteomic Data Using Predictive Regulatory Network Models of Host Response to Pathogens
Source: PLoS Comput Biol. 2016 Jul 12;12(7):e1005013. doi: 10.1371/journal.pcbi.1005013 (PMC4942116; doi:10.1371/journal.pcbi.1005013)
Supplement: S1 Fig — Expression patterns of 56 mouse modules with at least 10 genes. The red-blue heat map shows mean expression of all genes in each module for each sample, compared to mock treatment value (similar to Fig 2). Blocks of columns are time series (in days) from different viruses and dosages (PFU: Particle forming units). Viruses are ordered from low to high pathogenicity; different dosages of the same virus are placed next to each other. The “Genes” column shows the size of each module; larger values are shown in darker blue. Under “Enrichment”, a red box indicates module enrichment with any MSigDB motif, any curated gene set from Gene Ontology, KEGG, REACTOME or BioCarta (MSigDB curated gene sets), any influenza screen set, or any immune response gene set (described in Materials and Methods). (PDF) [file pcbi.1005013.s012.pdf]

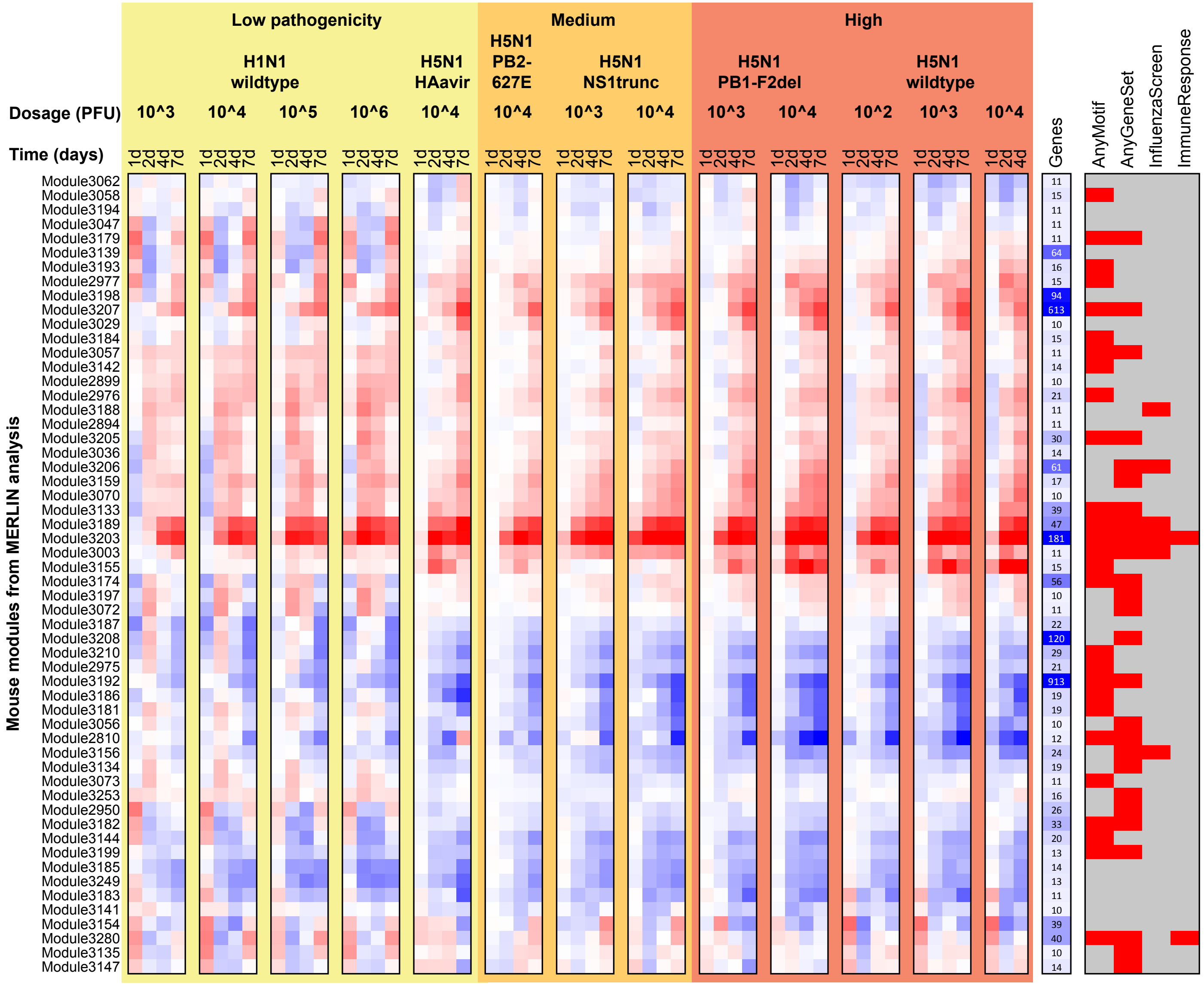
Low pathogenicity
Medium
High
H1N1 wildtype
H5N1 HAavir
H5N1 PB2-627E
H5N1 NS1trunc
H5N1 PB1-F2del
H5N1 wildtype
Dosage (PFU)
10^3
10^4
10^5
10^6
10^4
10^4
10^3
10^4
10^3
10^4
10^2
10^3
10^4
Time (days)
1d
2d
4d
7d
Genes
AnyMotif
AnyGeneSet
InfluenzaScreen
ImmuneResponse
Mouse modules from MERLIN analysis
Module3062
Module3058
Module3194
Module3047
Module3179
Module3139
Module3193
Module2977
Module3198
Module3207
Module3029
Module3184
Module3057
Module3142
Module2899
Module2976
Module3188
Module2894
Module3205
Module3036
Module3206
Module3159
Module3070
Module3133
Module3189
Module3203
Module3003
Module3155
Module3174
Module3197
Module3072
Module3187
Module3208
Module3210
Module2975
Module3192
Module3186
Module3181
Module3056
Module2810
Module3156
Module3134
Module3073
Module3253
Module2950
Module3182
Module3144
Module3199
Module3185
Module3249
Module3183
Module3141
Module3154
Module3280
Module3135
Module3147
11
15
11
11
11
64
16
15
94
613
10
15
11
14
10
21
11
11
30
14
61
17
10
39
47
181
11
15
56
10
11
22
120
29
21
913
19
19
10
12
24
19
11
16
26
33
20
13
14
13
11
10
39
40
10
14
