# Supplementary figures and images for: Integrating Transcriptomic and Proteomic Data Using Predictive Regulatory Network Models of Host Response to Pathogens
Source: PLoS Comput Biol. 2016 Jul 12;12(7):e1005013. doi: 10.1371/journal.pcbi.1005013 (PMC4942116; doi:10.1371/journal.pcbi.1005013)

**A**

Precision of top ranked genes (Human)

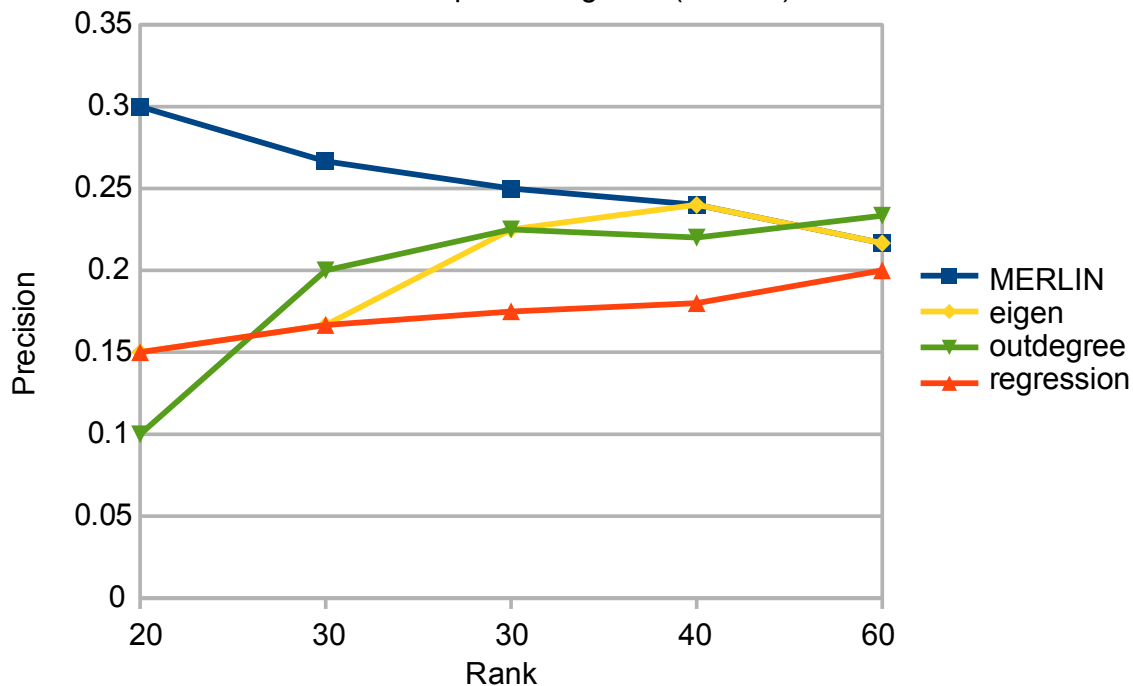**B**

Precision of top ranked genes (Mouse)

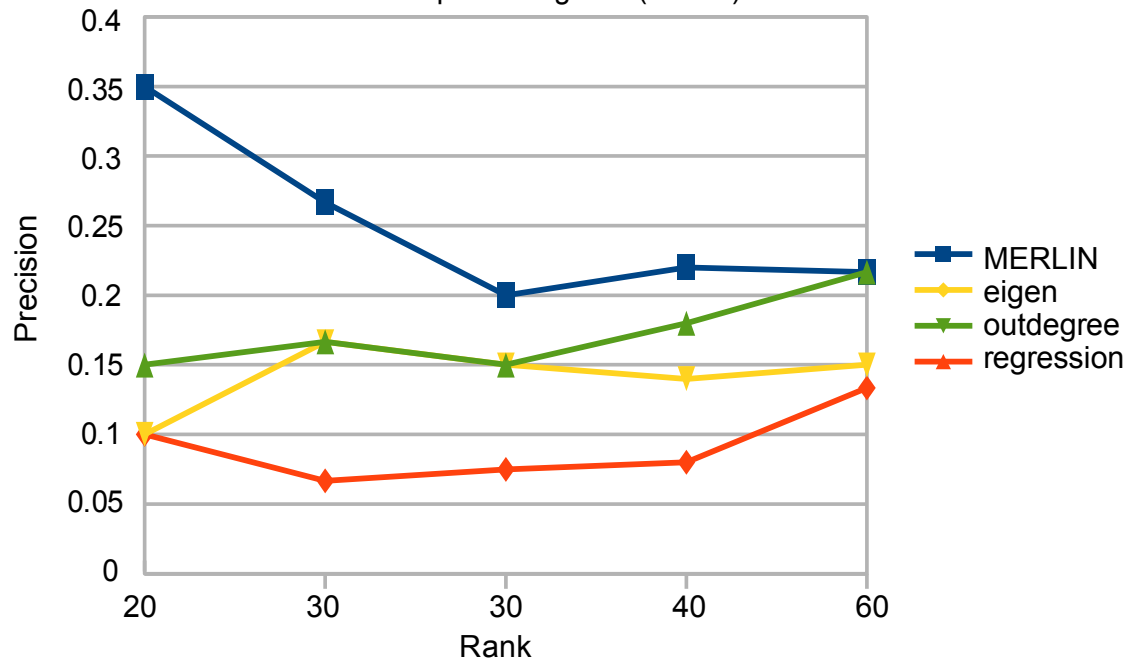

Supplement: S2 Fig — Shown is the precision of predicted top n regulators for human (A) and mouse (B) host response identified using different ranking strategies. Precision is defined as the fraction of the top n genes that are known host genes identified from screening studies. (PDF) [file pcbi.1005013.s013.pdf]

**A**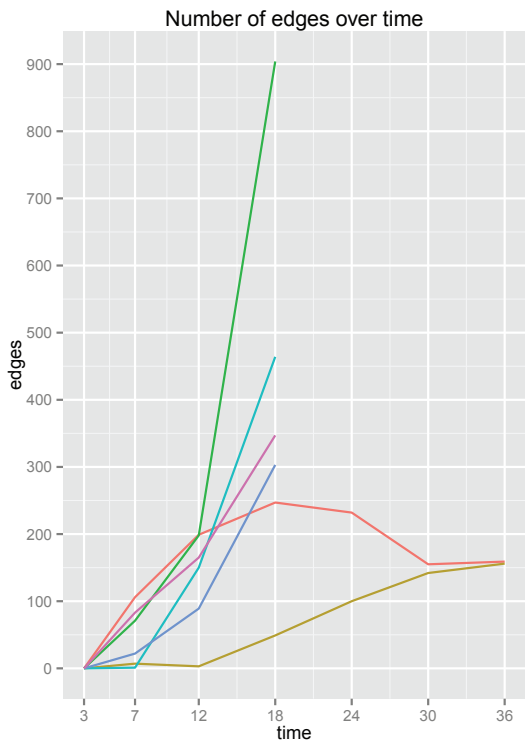**B**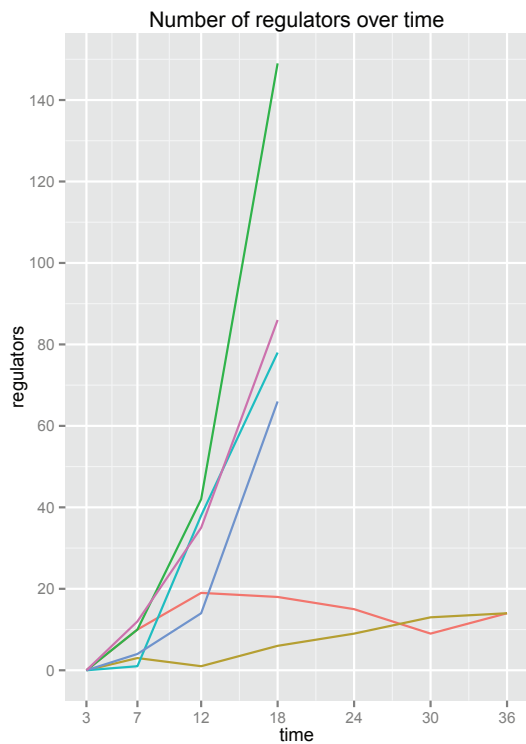**C**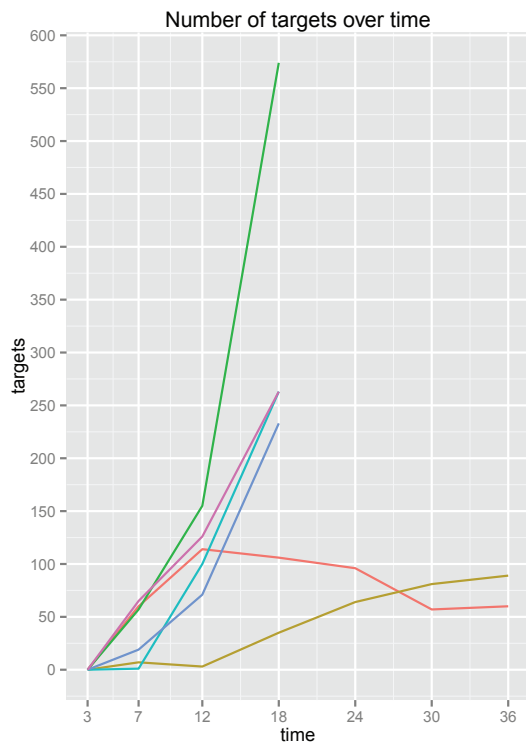

Supplement: S5 Fig — Each trajectory represents the count of active network elements of a specific type (edges (A), regulators (B), targets (C)) at a time point for one virus. (PDF) [file pcbi.1005013.s016.pdf]

Human Module 1472.  
A. Module expression

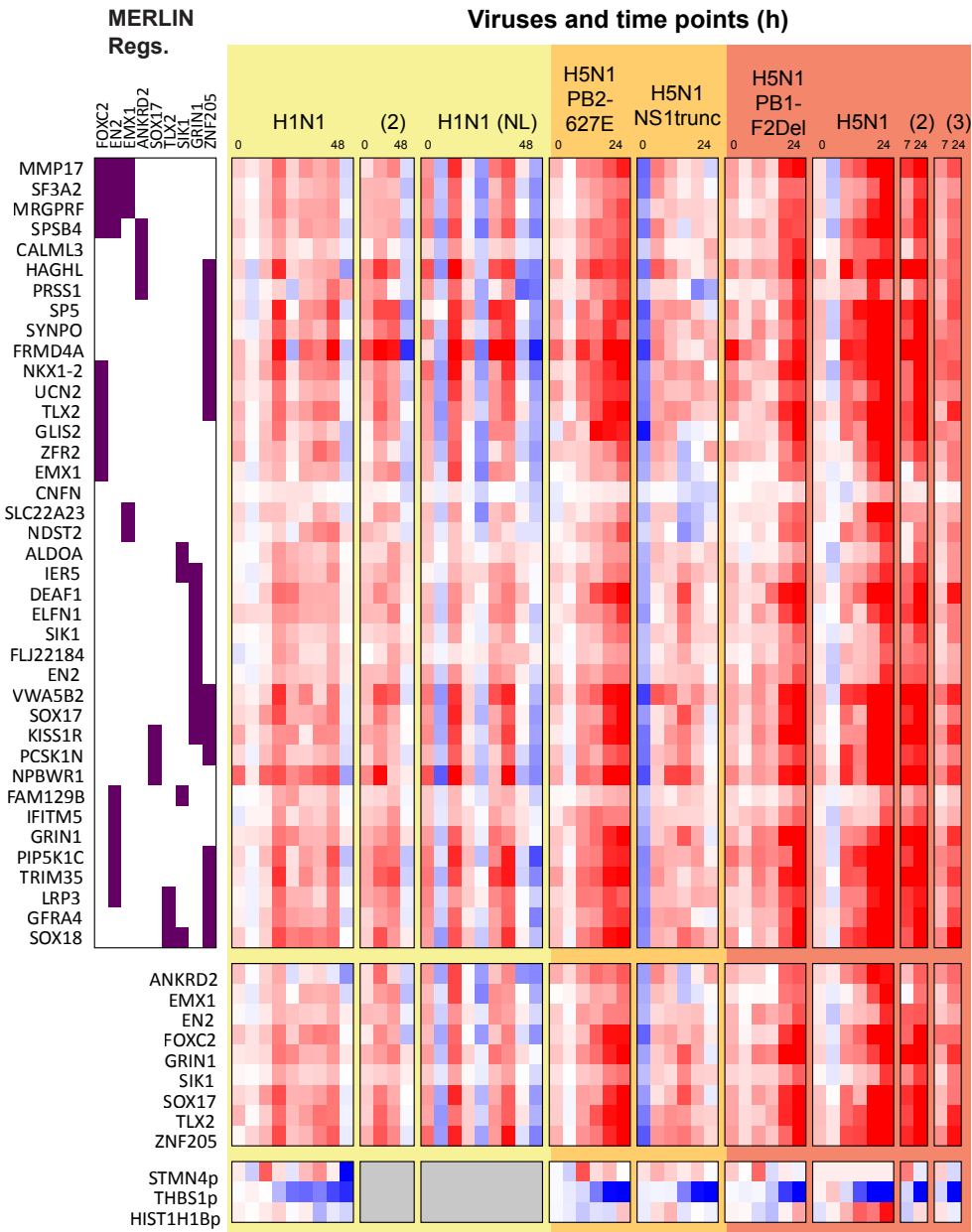

B. Candidate and high-confidence subnetwork

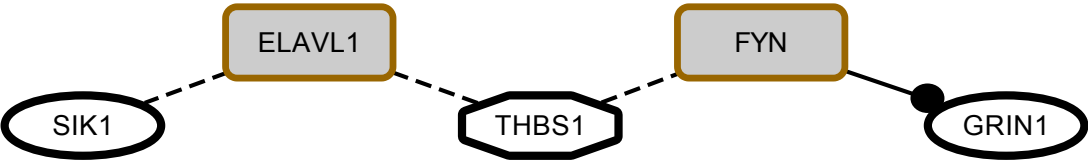

Supplement: S7 Fig — A. Heatmap of module genes and regulators from mRNA and protein for module 1472. B. Input subnetwork, which is the same as the refined output subnetwork. Nodes and edges follow the same legend as Fig 8. (PDF) [file pcbi.1005013.s018.pdf]

Human Module 1482  
A. Module expression

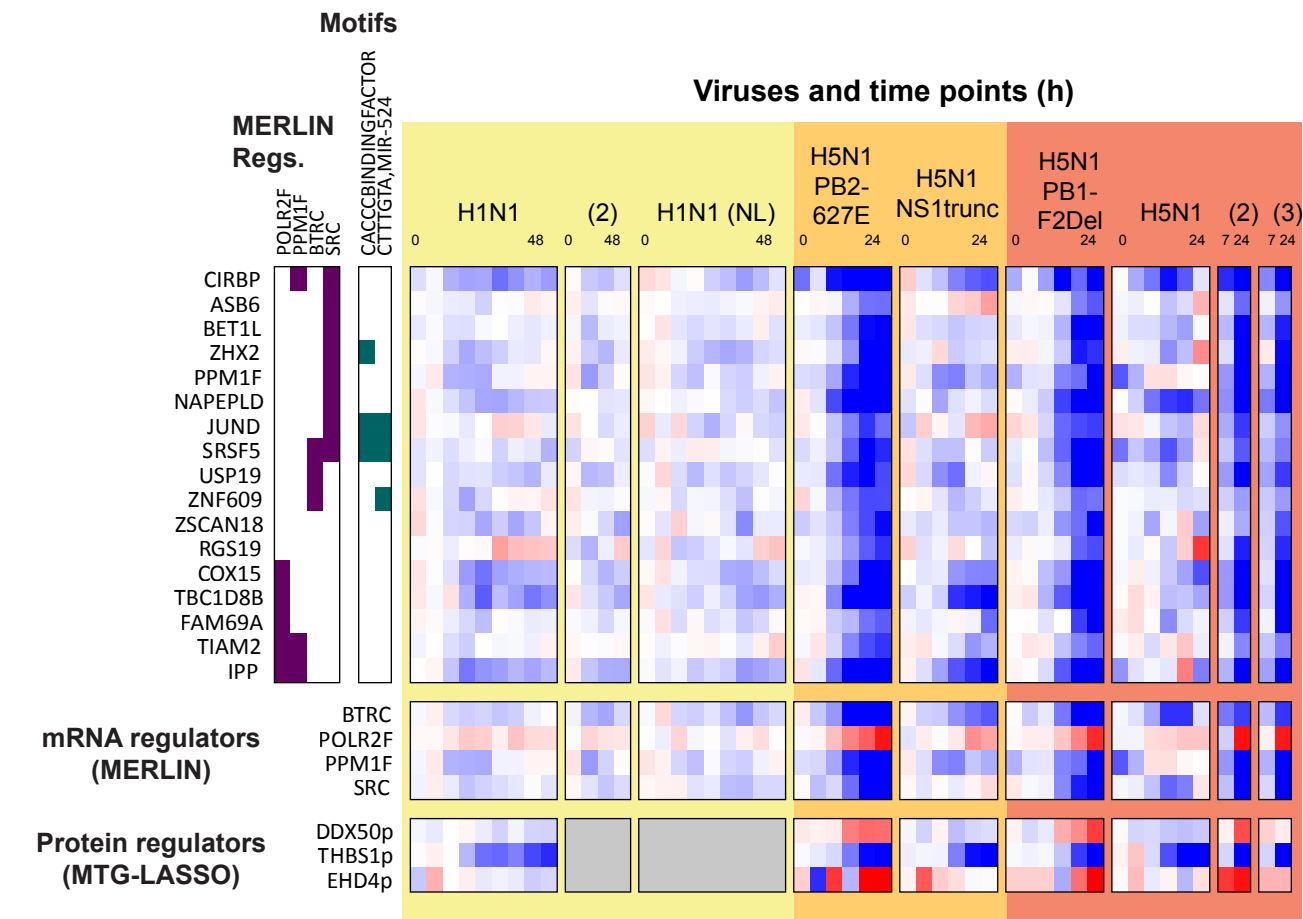

B. Candidate subnetwork

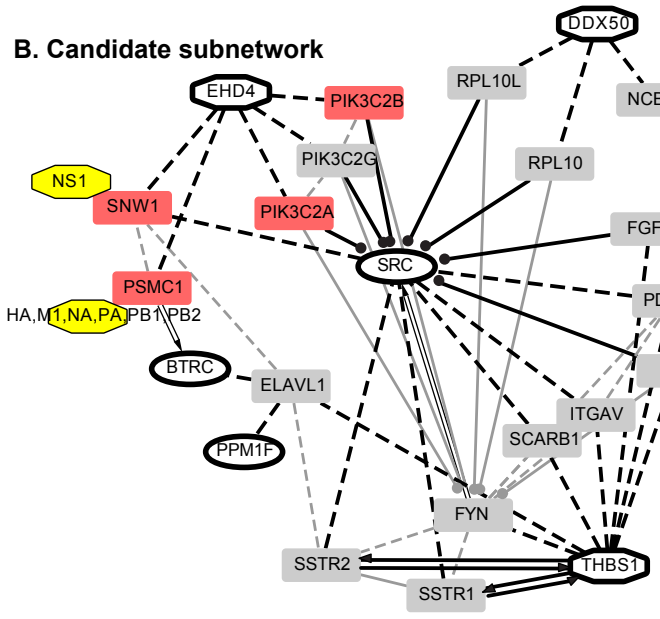

C. High-confidence subnetwork

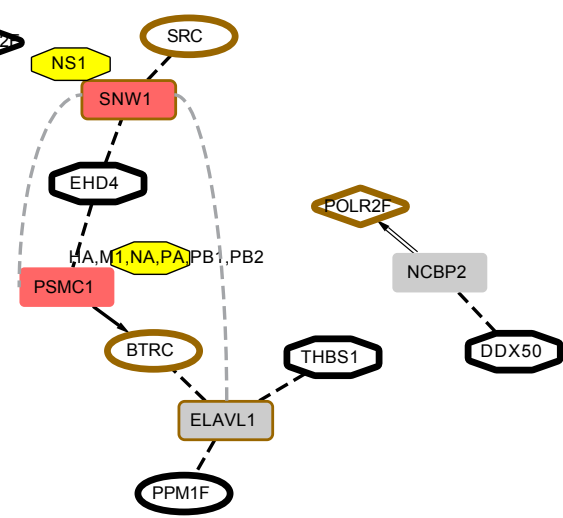

Supplement: S8 Fig — A. Heatmap of module genes and regulators from mRNA and protein for module 1482. B. Original input subnetwork connecting module regulators. Black edges link regulators and intermediates. Additional interactions between grey nodes are other background network edges. Nodes and edges follow the same legend as Fig 7. C. High-confidence physical subnetwork. (PDF) [file pcbi.1005013.s019.pdf]

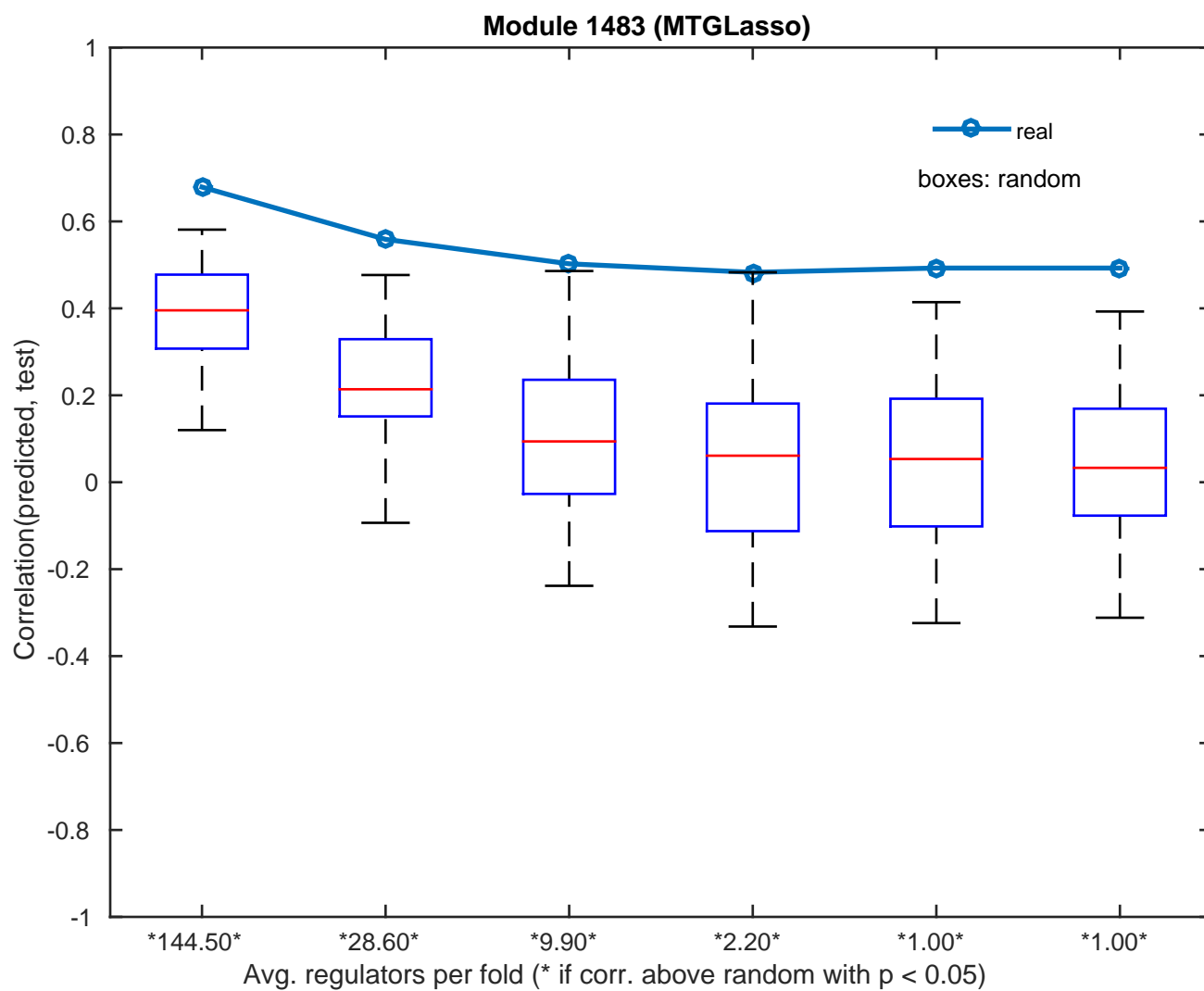

Supplement: S1 Dataset — One plot per module, both species. (GZ) [file pcbi.1005013.s024.gz › human_correlation_vs_lambda/human_module1483_mtglasso_all_lambdas.pdf]

### Module 1551 (MTGLasso)

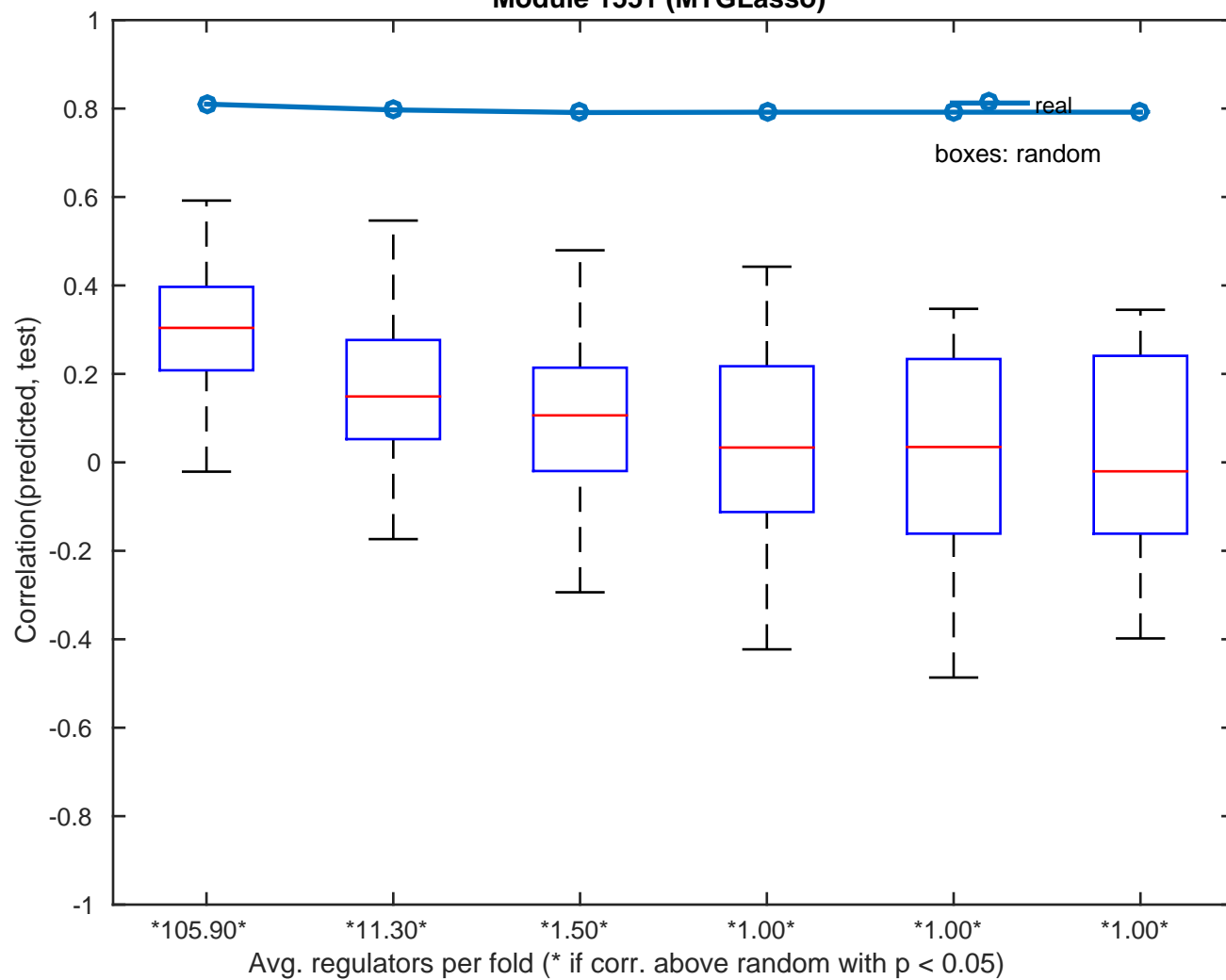

Supplement: S1 Dataset — One plot per module, both species. (GZ) [file pcbi.1005013.s024.gz › human_correlation_vs_lambda/human_module1551_mtglasso_all_lambdas.pdf]

### Module 1591 (MTGLasso)

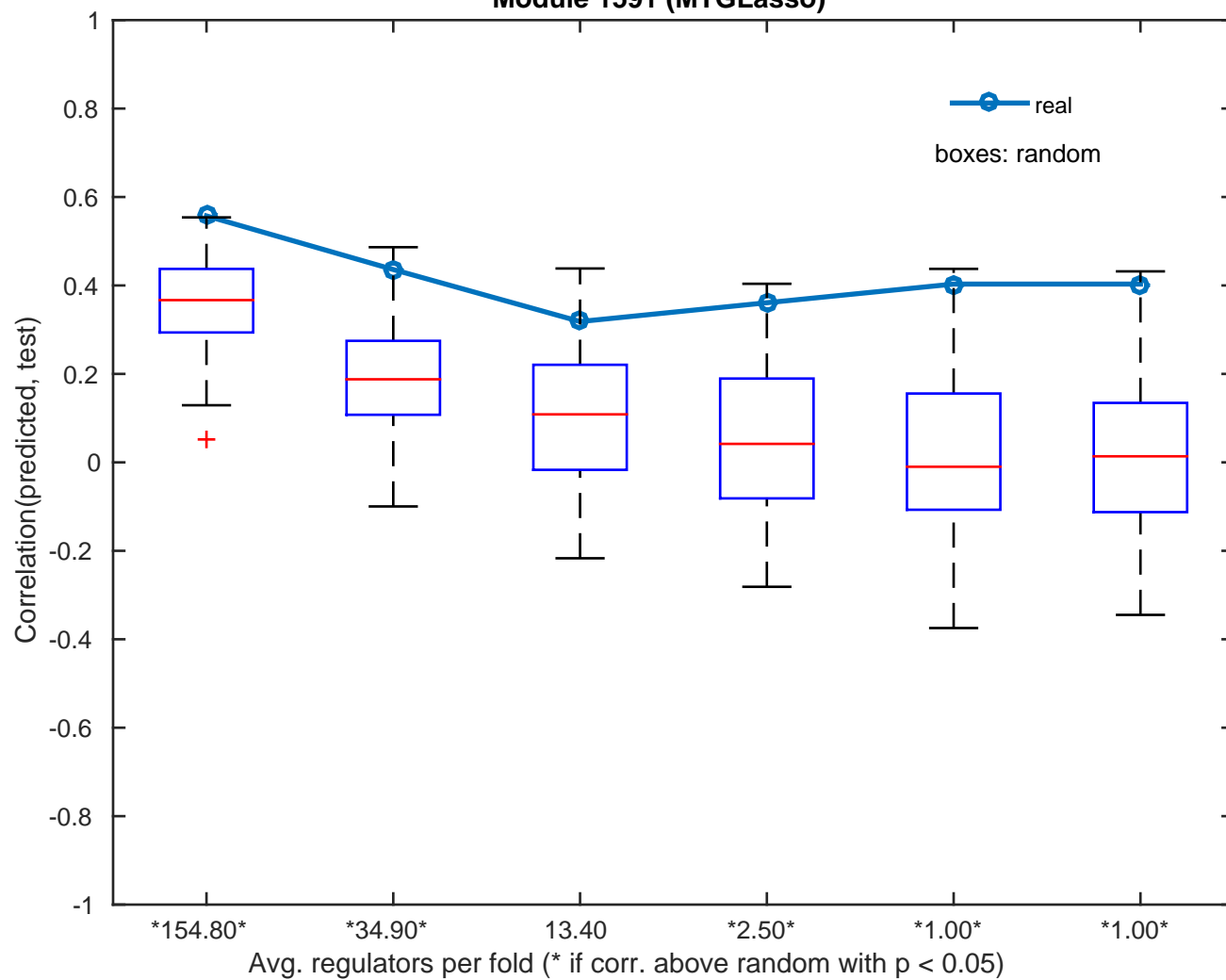

Supplement: S1 Dataset — One plot per module, both species. (GZ) [file pcbi.1005013.s024.gz › human_correlation_vs_lambda/human_module1591_mtglasso_all_lambdas.pdf]

Module 1469 (MTGLasso)

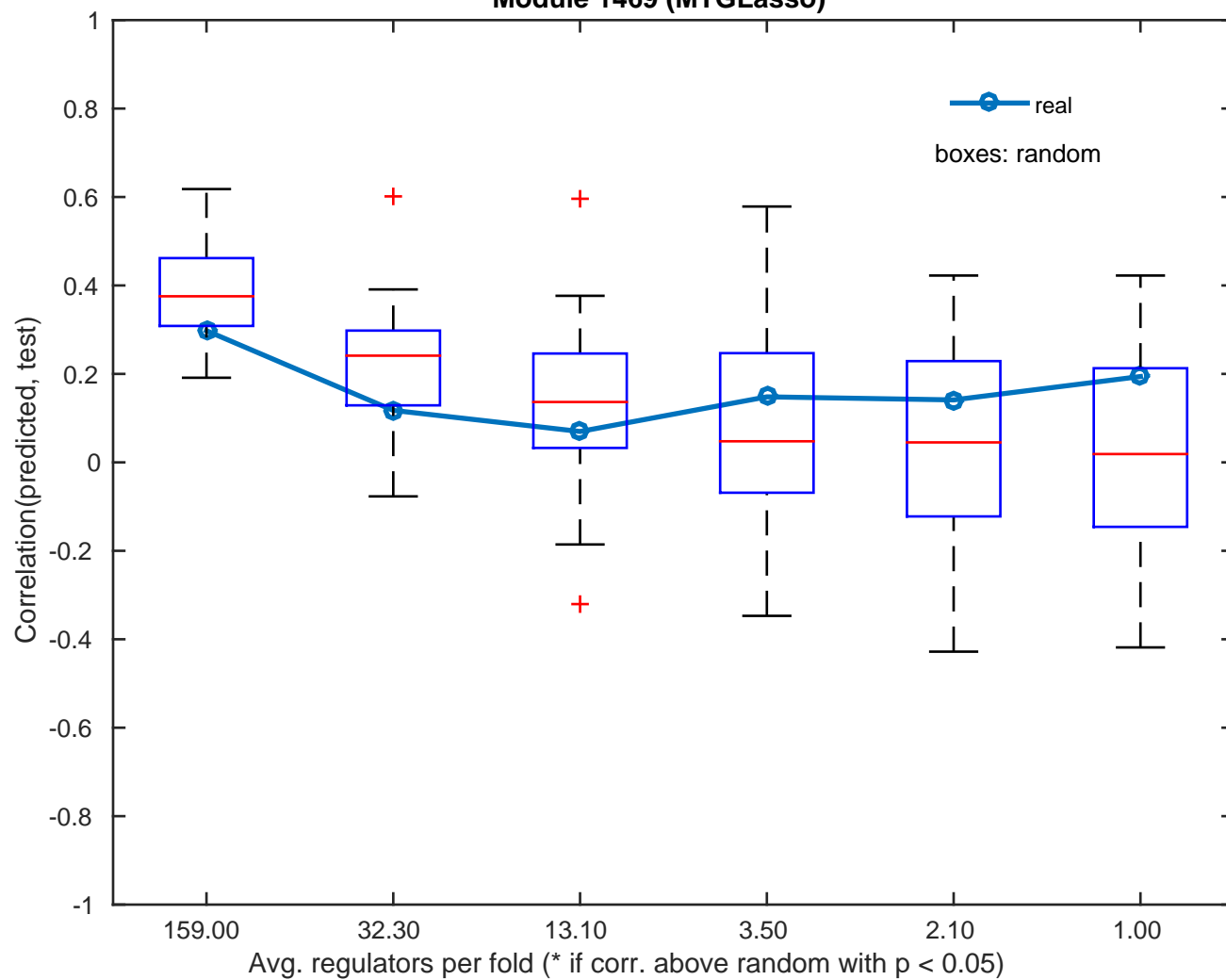

Supplement: S1 Dataset — One plot per module, both species. (GZ) [file pcbi.1005013.s024.gz › human_correlation_vs_lambda/human_module1469_mtglasso_all_lambdas.pdf]

Module 1482 (MTGLasso)

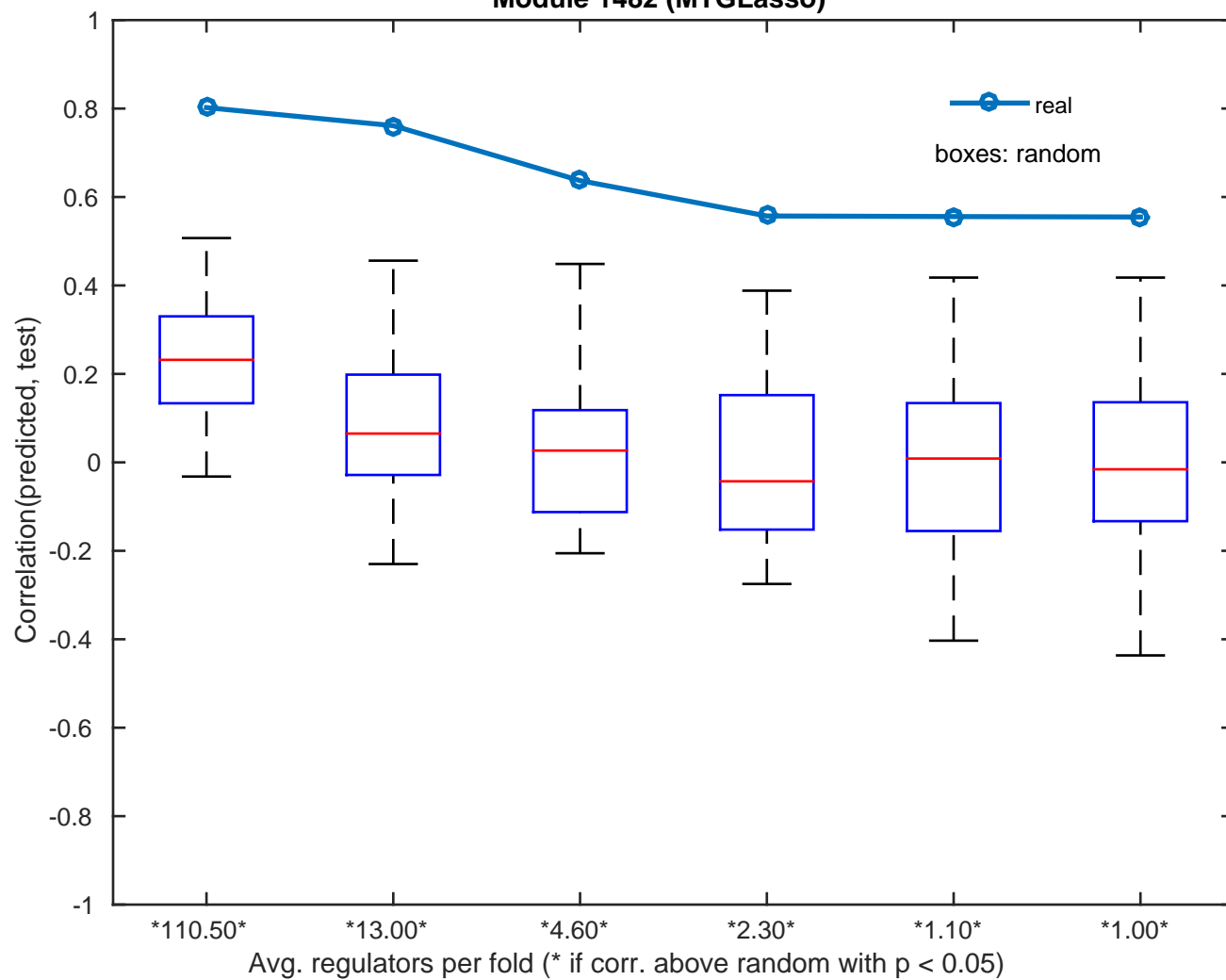

Supplement: S1 Dataset — One plot per module, both species. (GZ) [file pcbi.1005013.s024.gz › human_correlation_vs_lambda/human_module1482_mtglasso_all_lambdas.pdf]

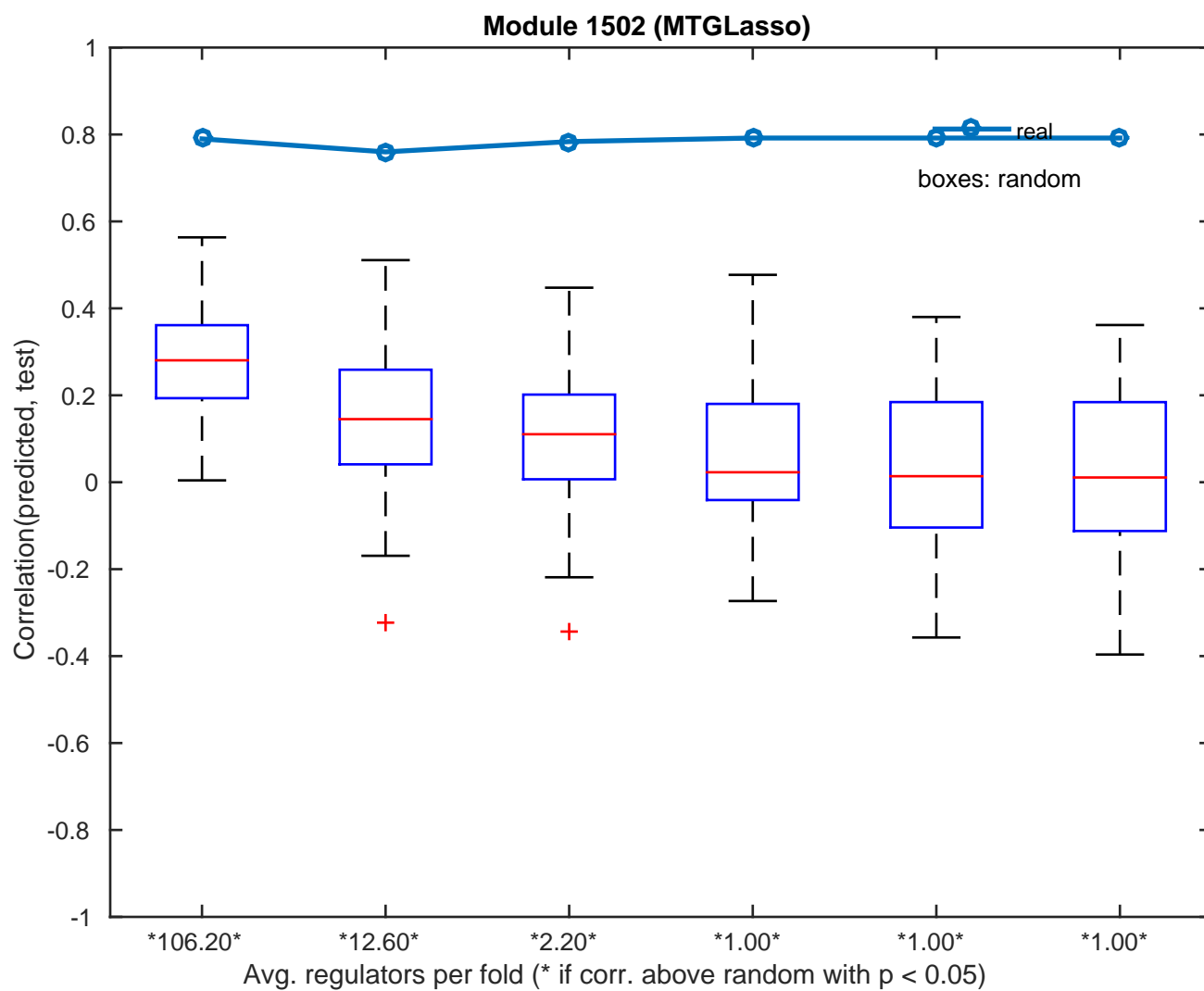

Supplement: S1 Dataset — One plot per module, both species. (GZ) [file pcbi.1005013.s024.gz › human_correlation_vs_lambda/human_module1502_mtglasso_all_lambdas.pdf]

Module 1553 (MTGLasso)

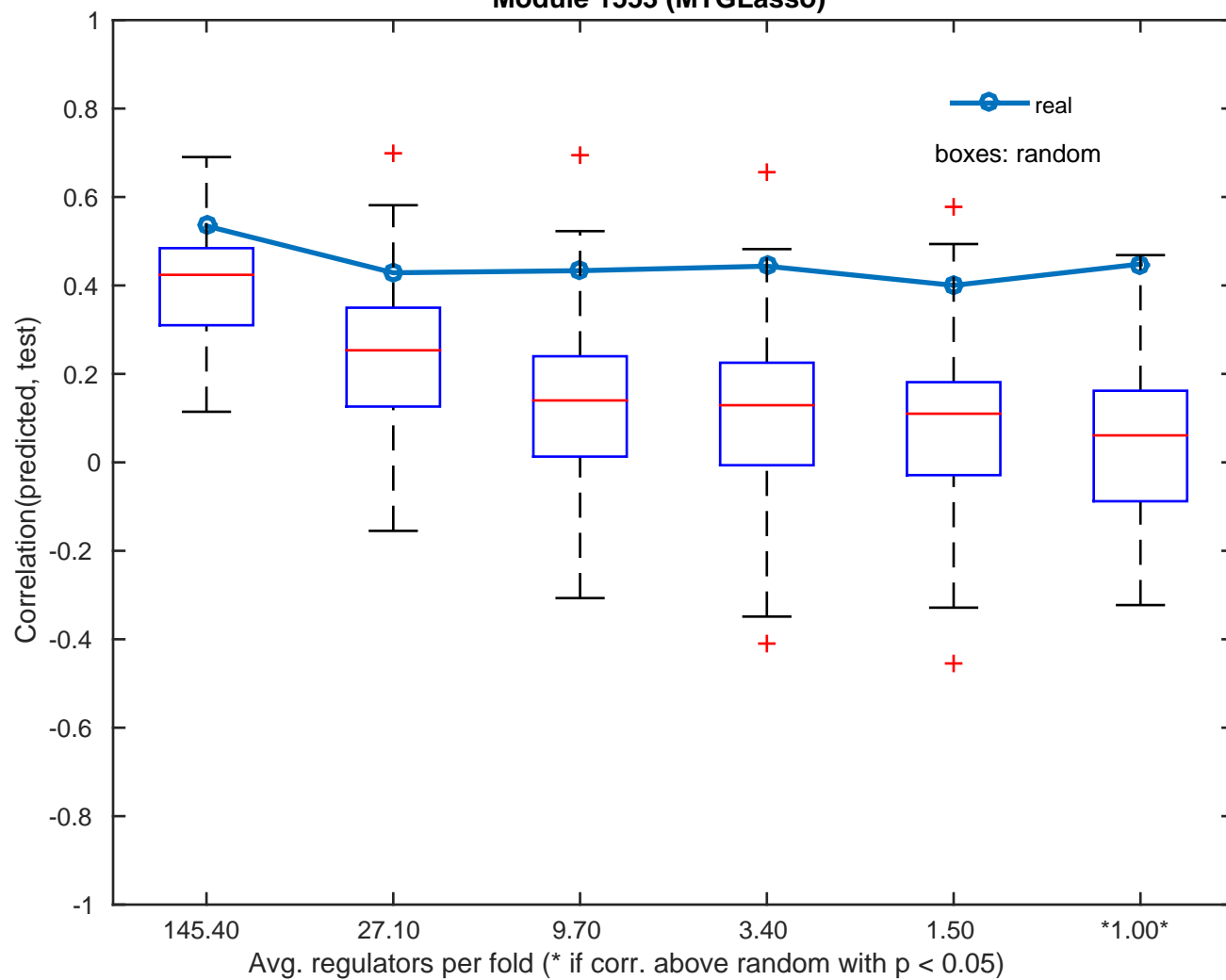

Supplement: S1 Dataset — One plot per module, both species. (GZ) [file pcbi.1005013.s024.gz › human_correlation_vs_lambda/human_module1553_mtglasso_all_lambdas.pdf]

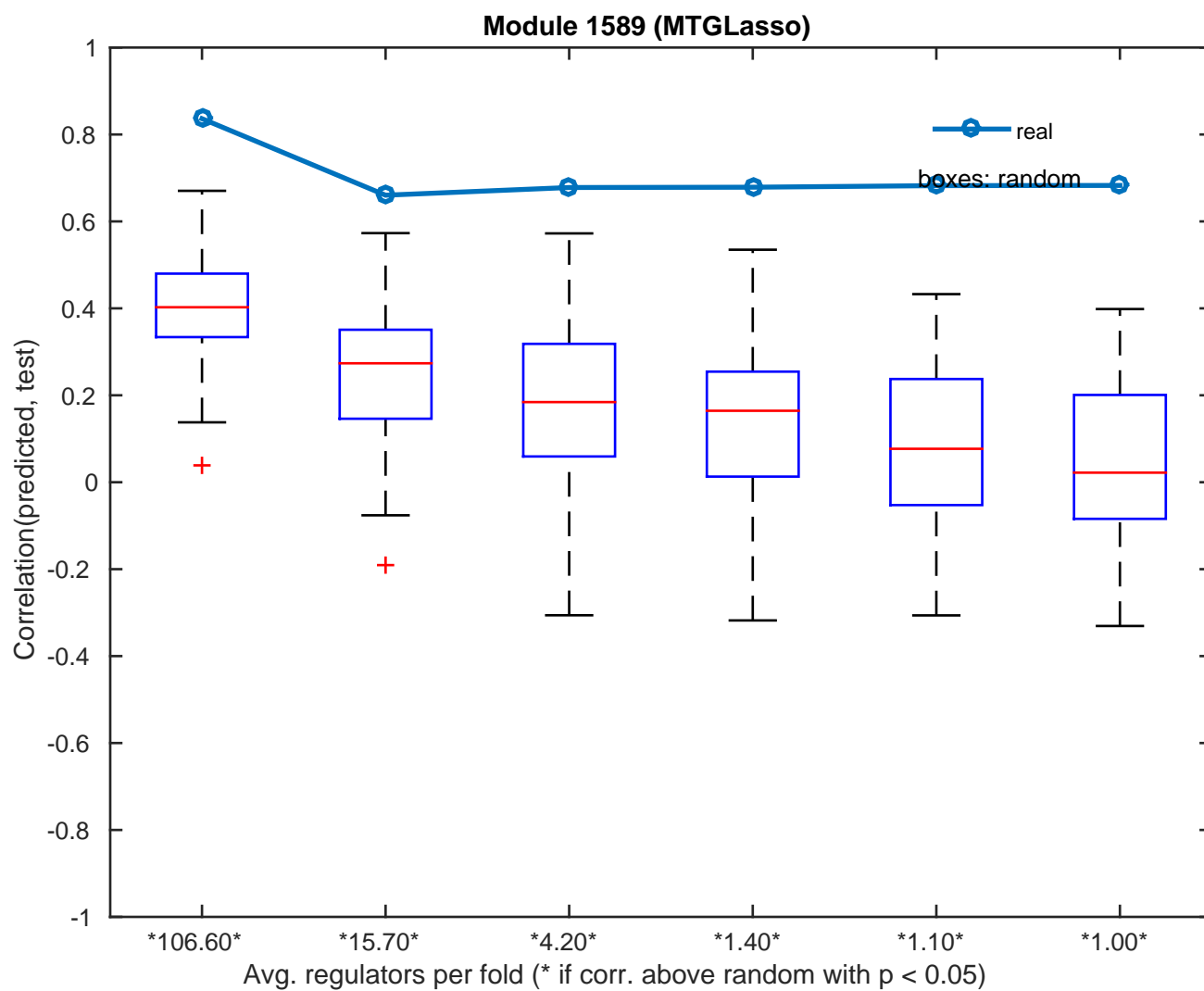

Supplement: S1 Dataset — One plot per module, both species. (GZ) [file pcbi.1005013.s024.gz › human_correlation_vs_lambda/human_module1589_mtglasso_all_lambdas.pdf]

Module 1593 (MTGLasso)

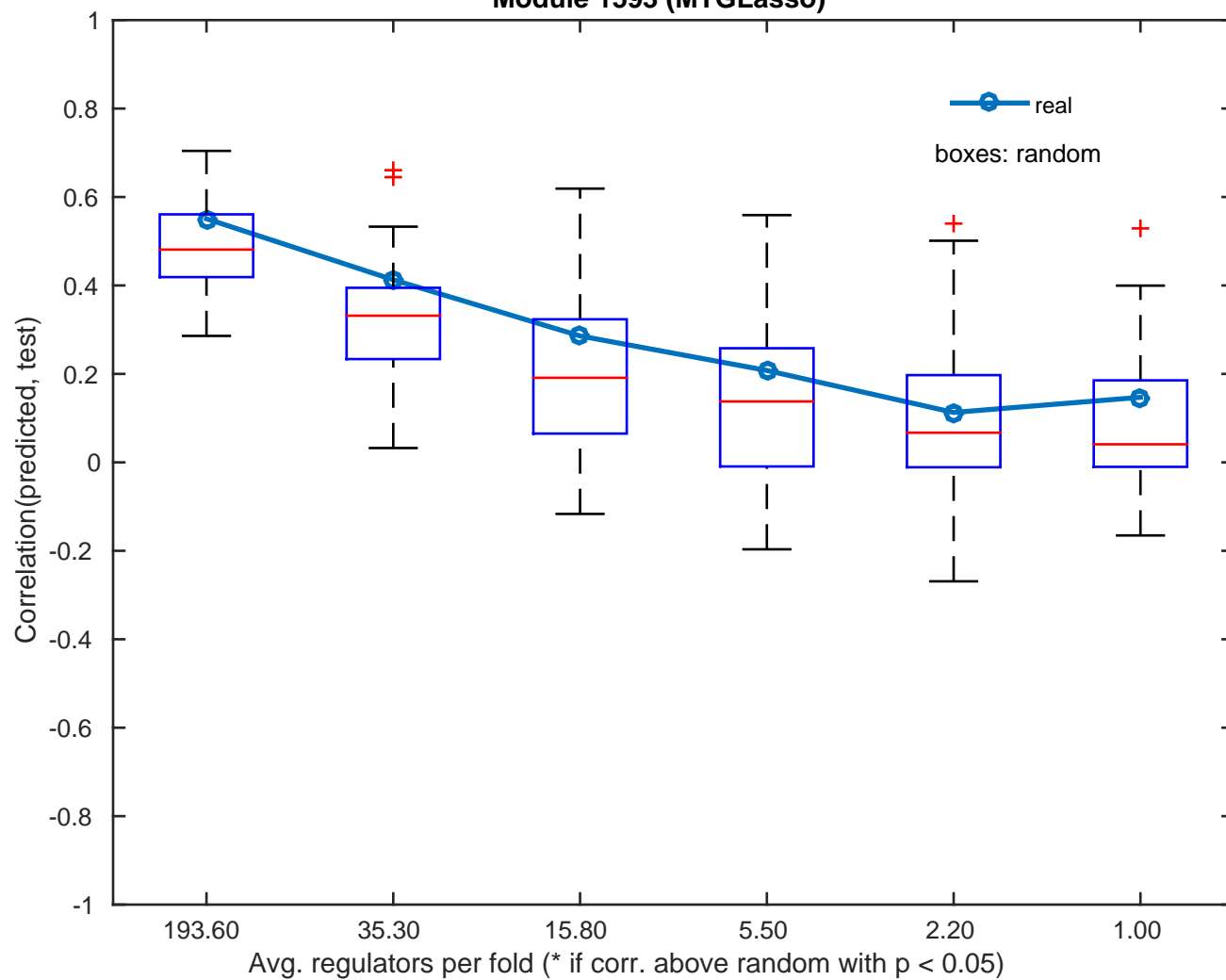

Supplement: S1 Dataset — One plot per module, both species. (GZ) [file pcbi.1005013.s024.gz › human_correlation_vs_lambda/human_module1593_mtglasso_all_lambdas.pdf]

Module 1597 (MTGLasso)

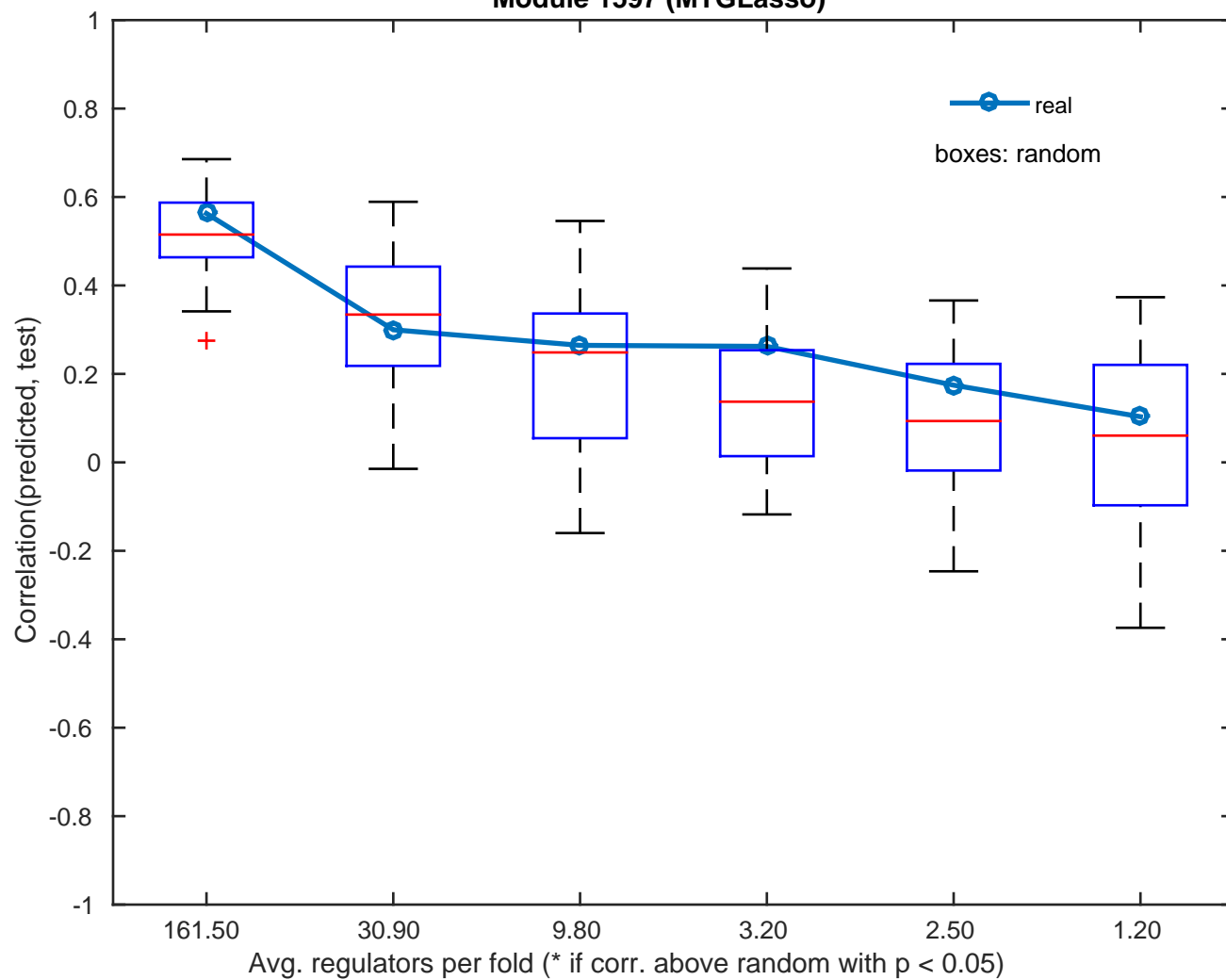

Supplement: S1 Dataset — One plot per module, both species. (GZ) [file pcbi.1005013.s024.gz › human_correlation_vs_lambda/human_module1597_mtglasso_all_lambdas.pdf]

Module 1329 (MTGLasso)

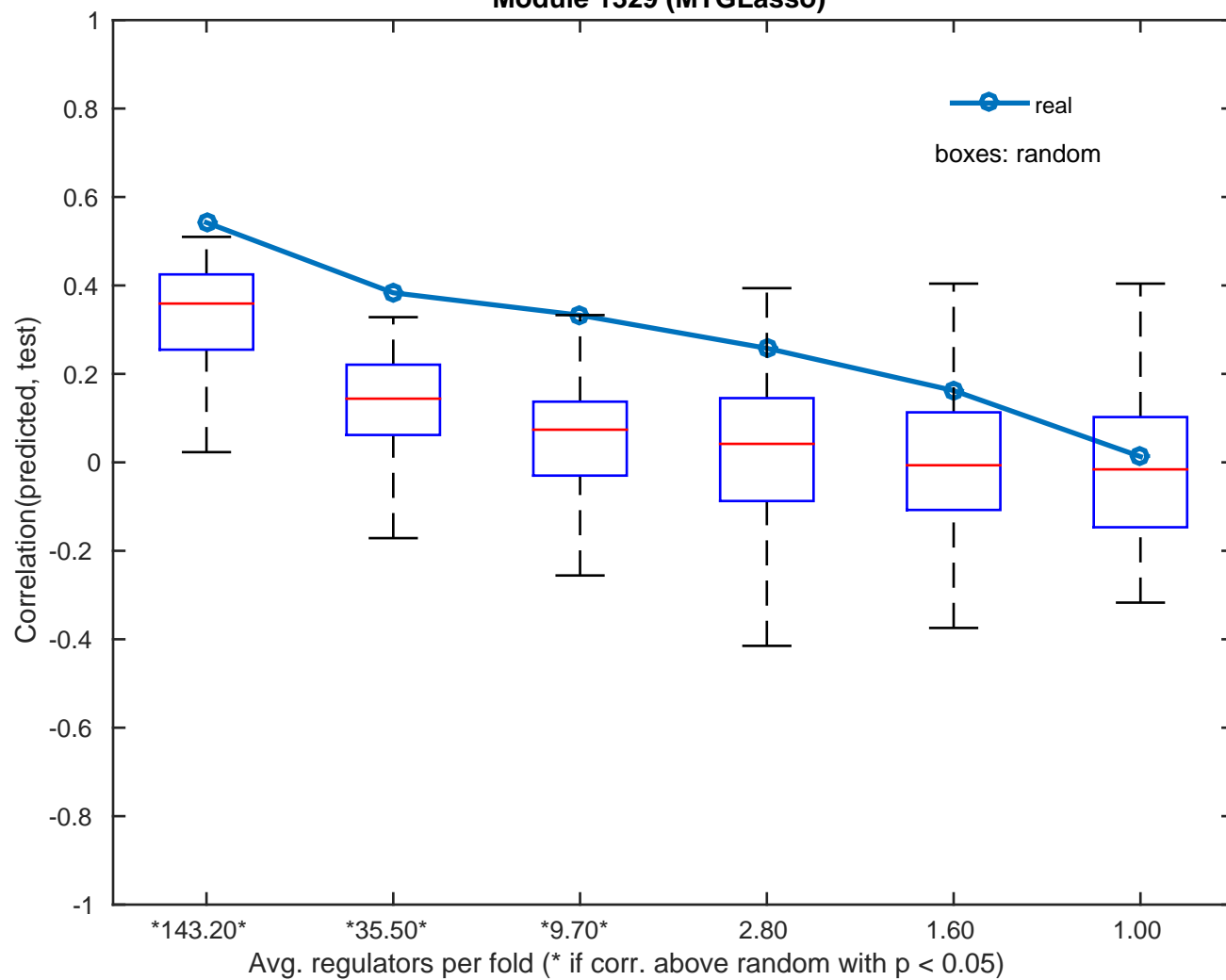

Supplement: S1 Dataset — One plot per module, both species. (GZ) [file pcbi.1005013.s024.gz › human_correlation_vs_lambda/human_module1329_mtglasso_all_lambdas.pdf]

Module 1490 (MTGLasso)

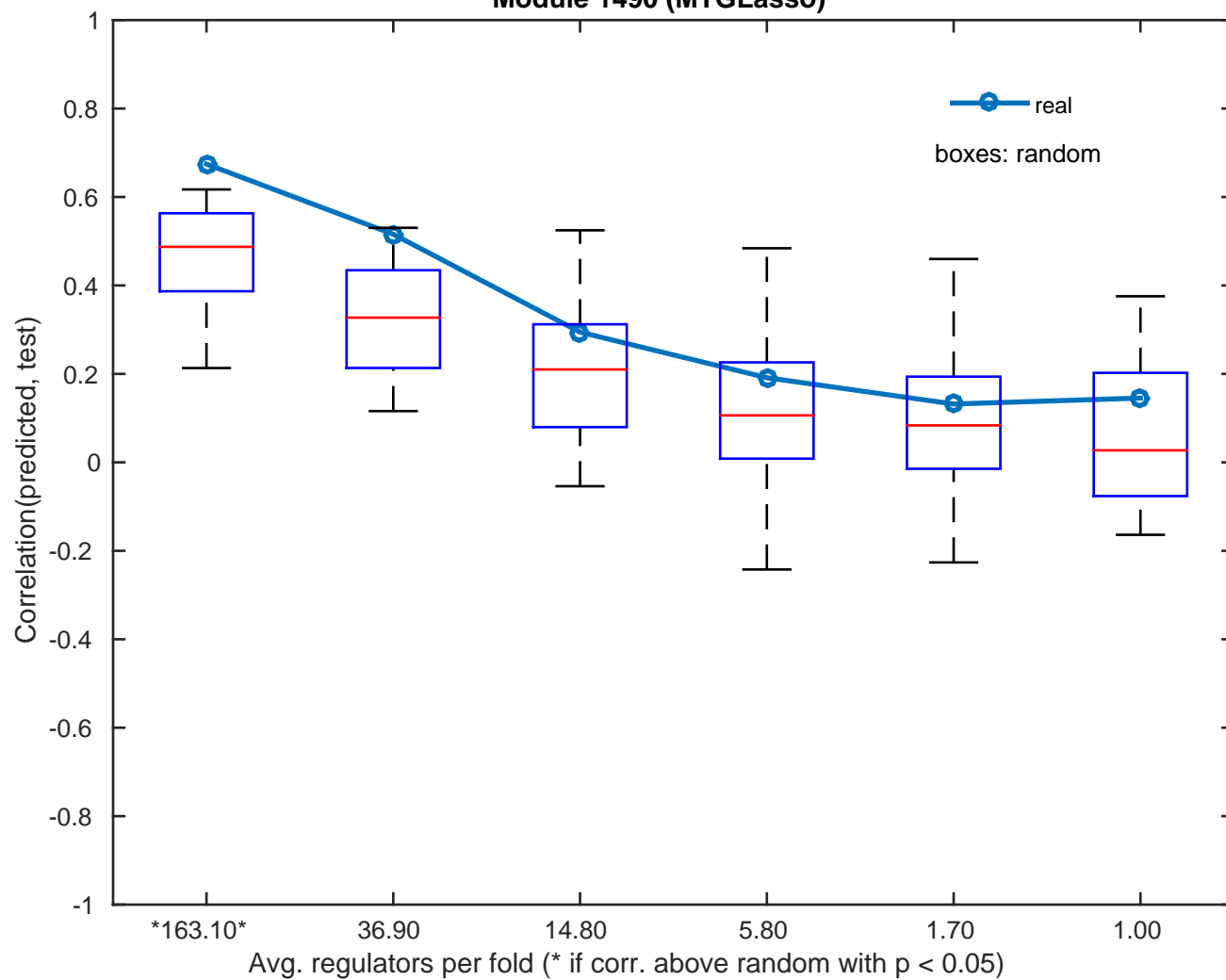

Supplement: S1 Dataset — One plot per module, both species. (GZ) [file pcbi.1005013.s024.gz › human_correlation_vs_lambda/human_module1490_mtglasso_all_lambdas.pdf]

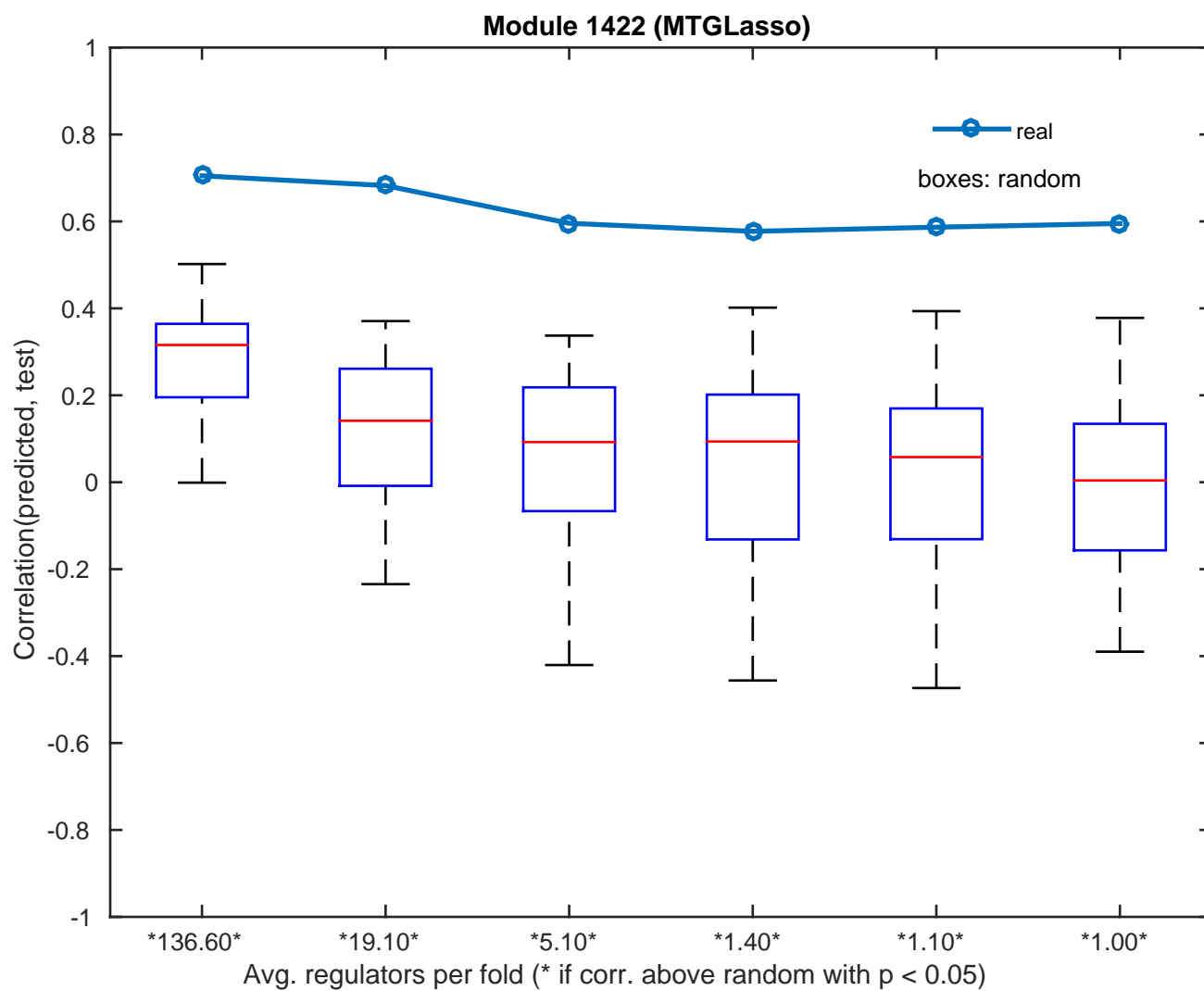

Supplement: S1 Dataset — One plot per module, both species. (GZ) [file pcbi.1005013.s024.gz › human_correlation_vs_lambda/human_module1422_mtglasso_all_lambdas.pdf]

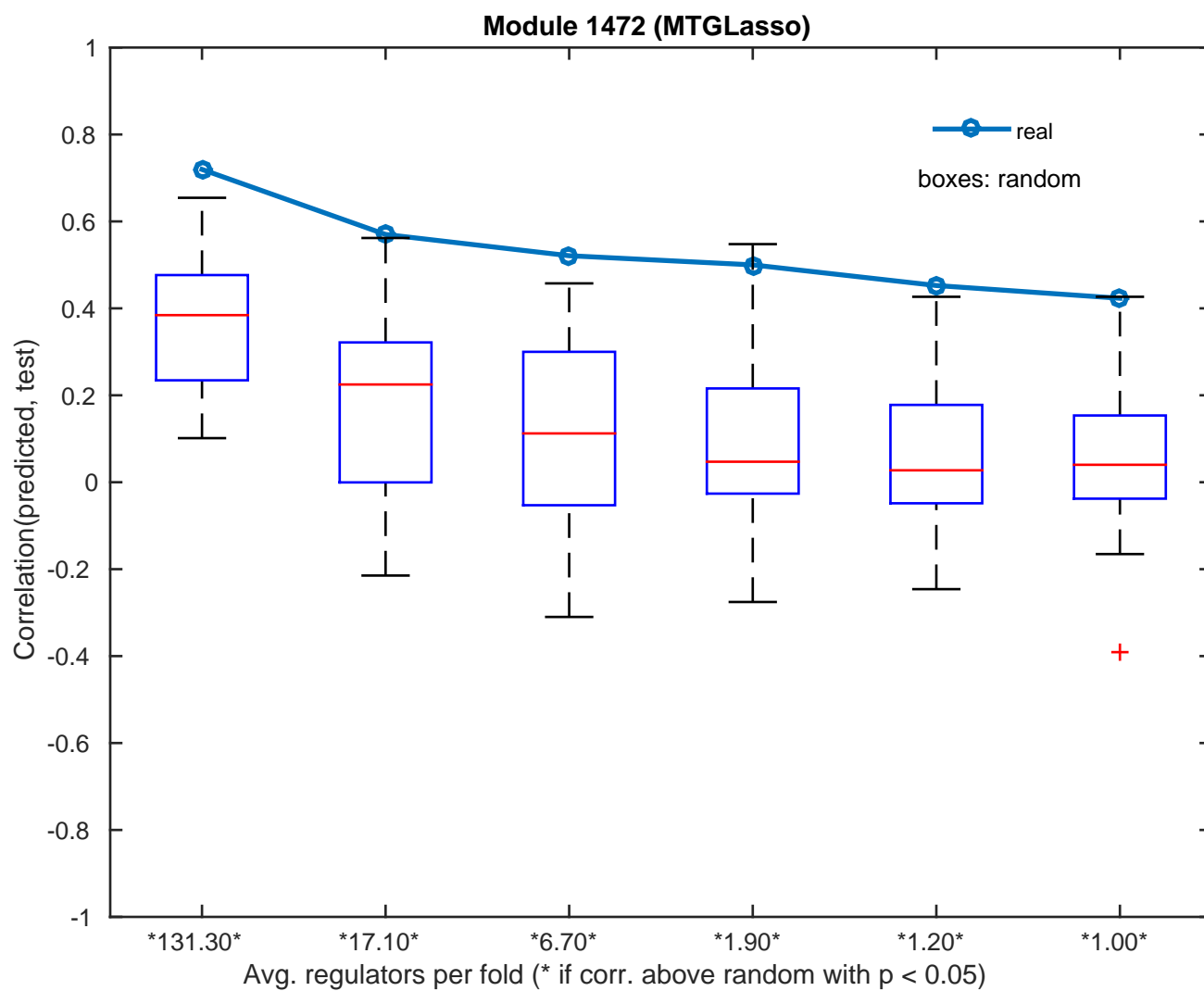

Supplement: S1 Dataset — One plot per module, both species. (GZ) [file pcbi.1005013.s024.gz › human_correlation_vs_lambda/human_module1472_mtglasso_all_lambdas.pdf]

Module 1423 (MTGLasso)

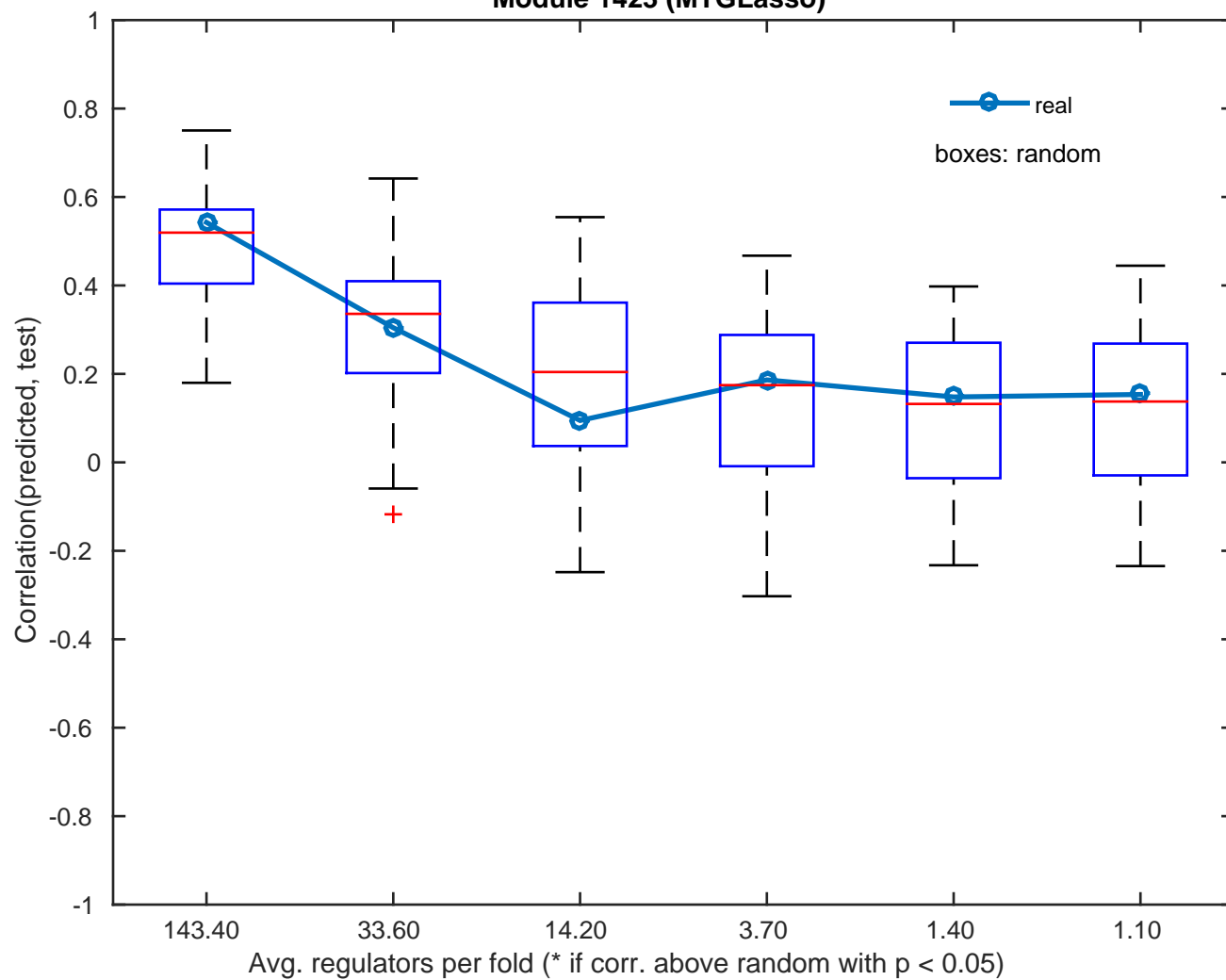

Supplement: S1 Dataset — One plot per module, both species. (GZ) [file pcbi.1005013.s024.gz › human_correlation_vs_lambda/human_module1423_mtglasso_all_lambdas.pdf]

Module 1484 (MTGLasso)

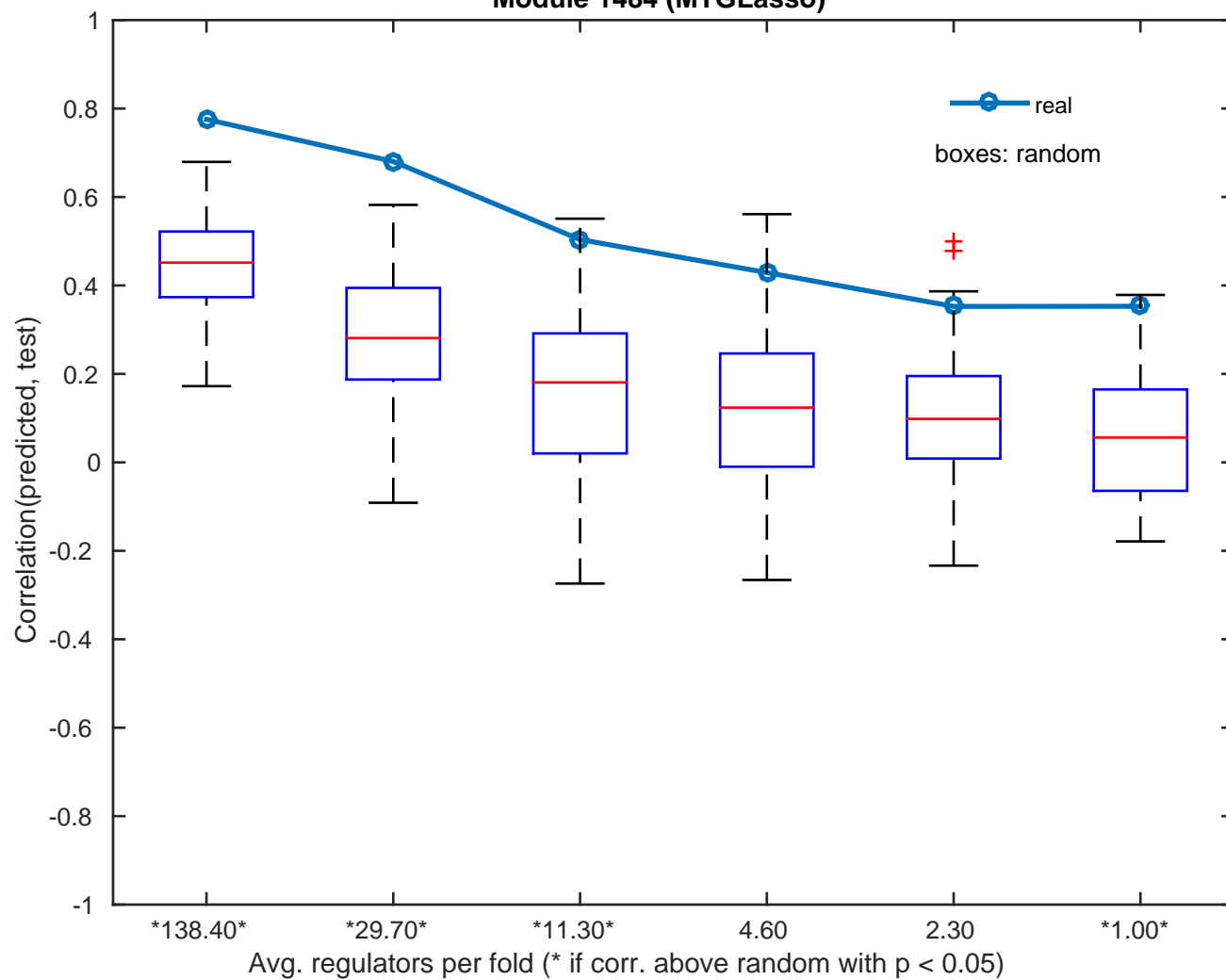

Supplement: S1 Dataset — One plot per module, both species. (GZ) [file pcbi.1005013.s024.gz › human_correlation_vs_lambda/human_module1484_mtglasso_all_lambdas.pdf]

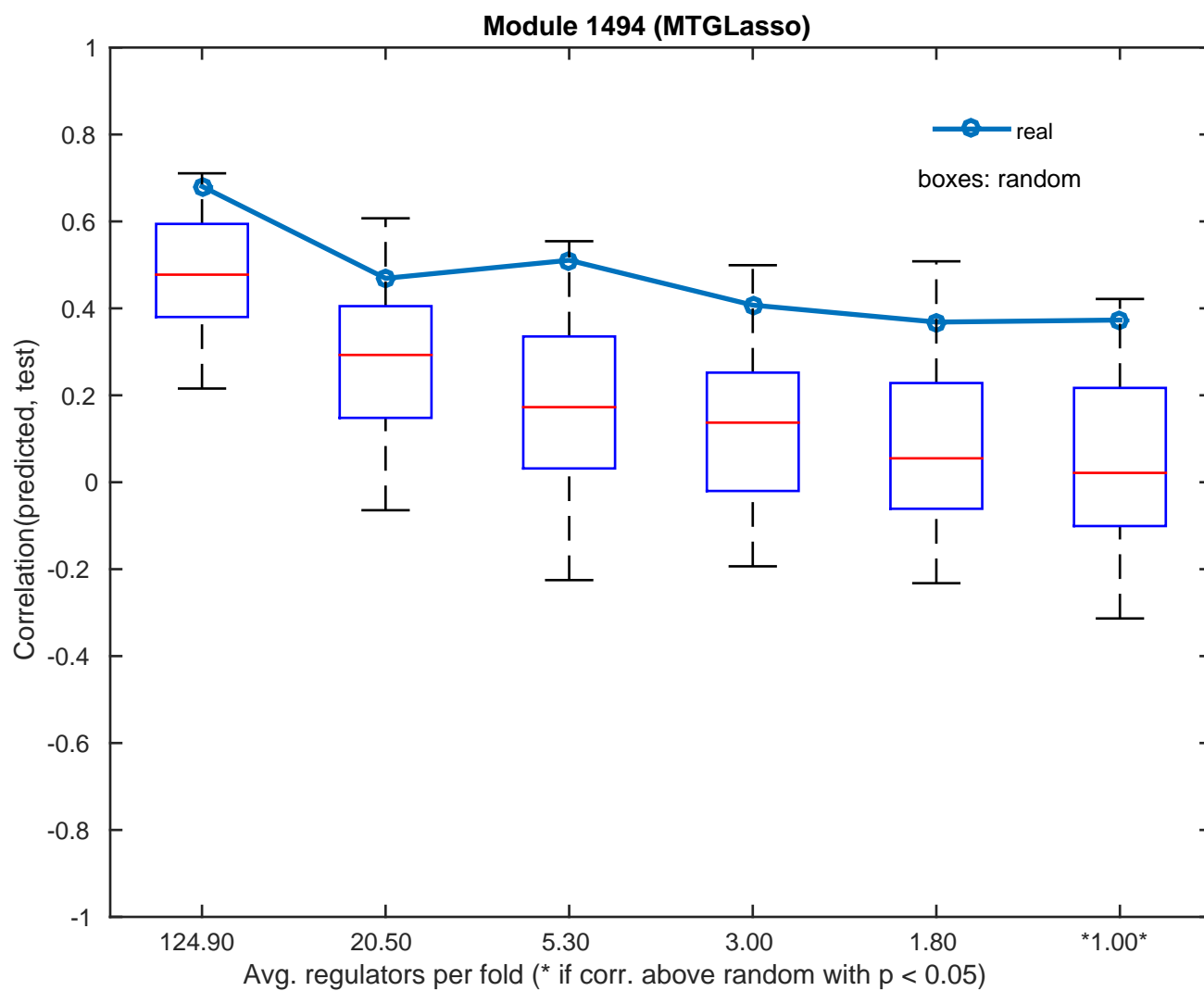

Supplement: S1 Dataset — One plot per module, both species. (GZ) [file pcbi.1005013.s024.gz › human_correlation_vs_lambda/human_module1494_mtglasso_all_lambdas.pdf]

Module 1566 (MTGLasso)

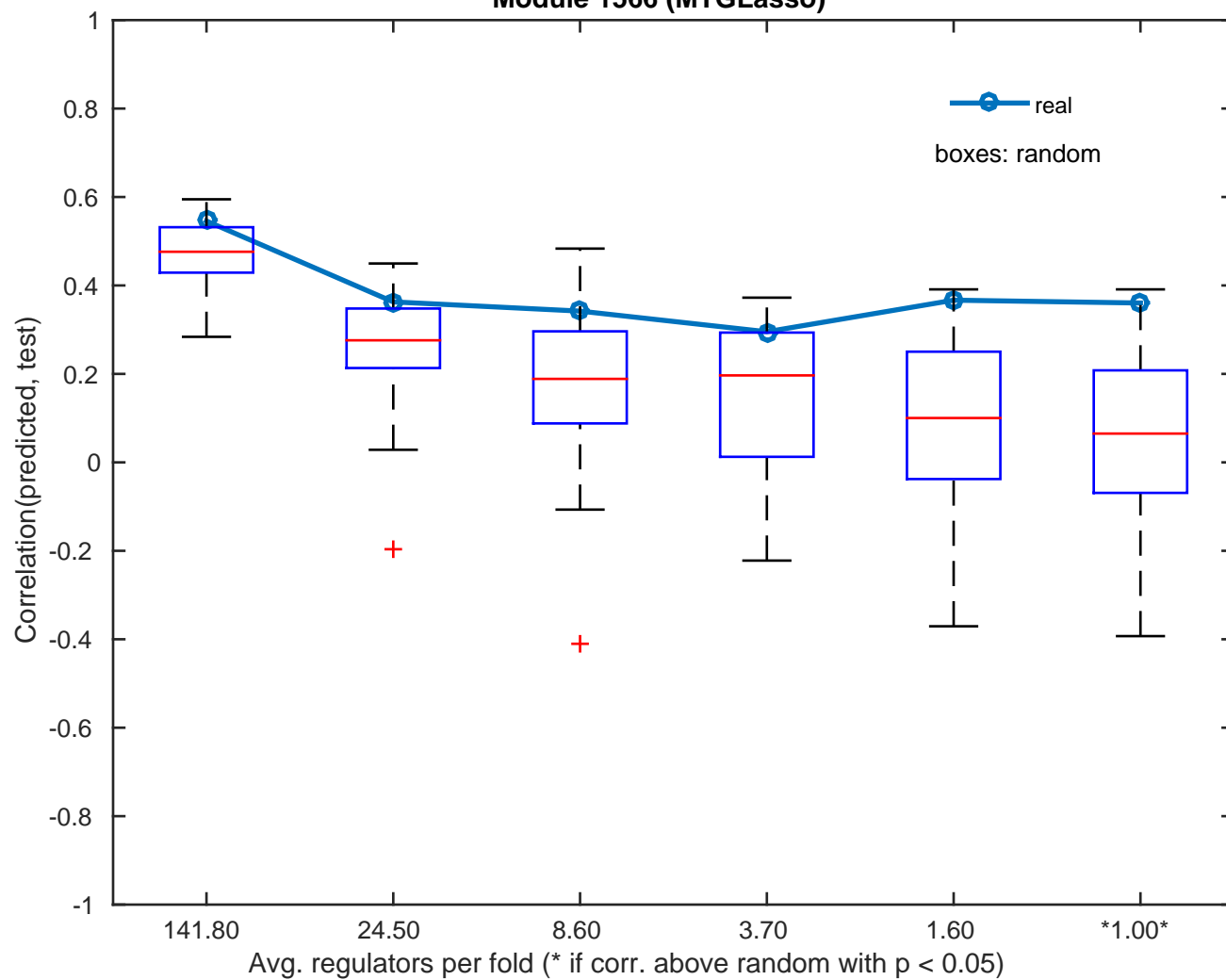

Supplement: S1 Dataset — One plot per module, both species. (GZ) [file pcbi.1005013.s024.gz › human_correlation_vs_lambda/human_module1566_mtglasso_all_lambdas.pdf]

Module 1590 (MTGLasso)

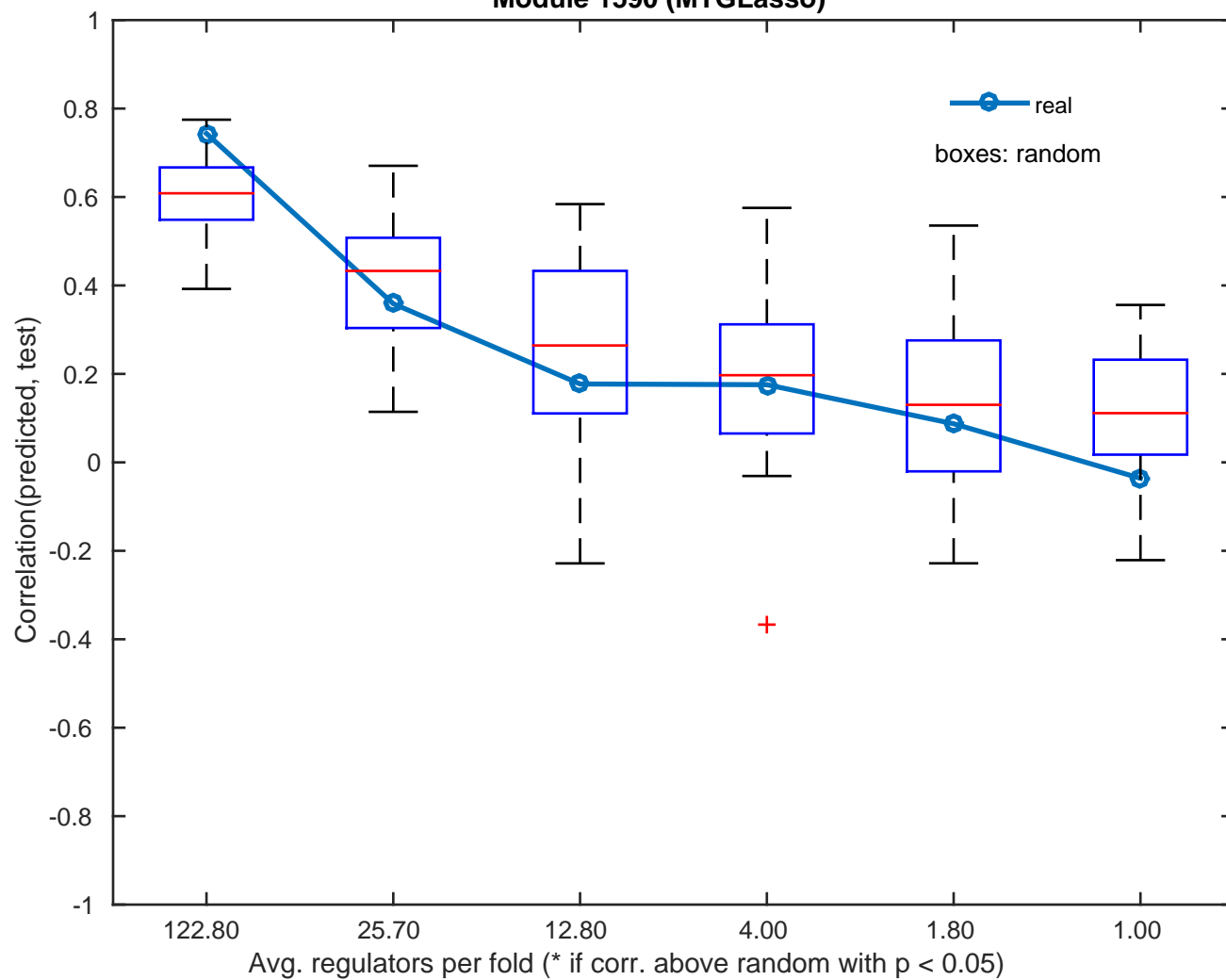

Supplement: S1 Dataset — One plot per module, both species. (GZ) [file pcbi.1005013.s024.gz › human_correlation_vs_lambda/human_module1590_mtglasso_all_lambdas.pdf]

Module 1424 (MTGLasso)

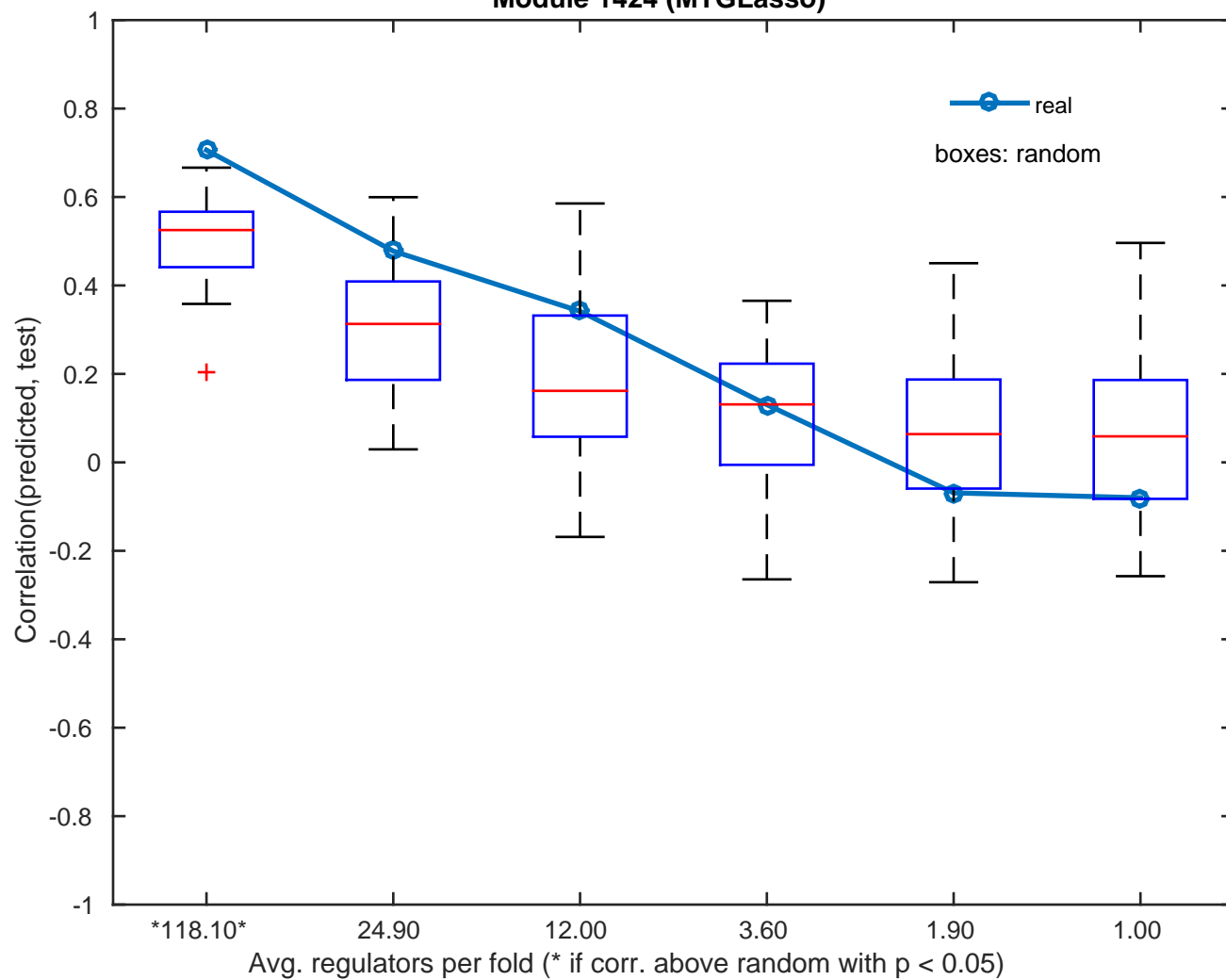

Supplement: S1 Dataset — One plot per module, both species. (GZ) [file pcbi.1005013.s024.gz › human_correlation_vs_lambda/human_module1424_mtglasso_all_lambdas.pdf]

Module 1499 (MTGLasso)

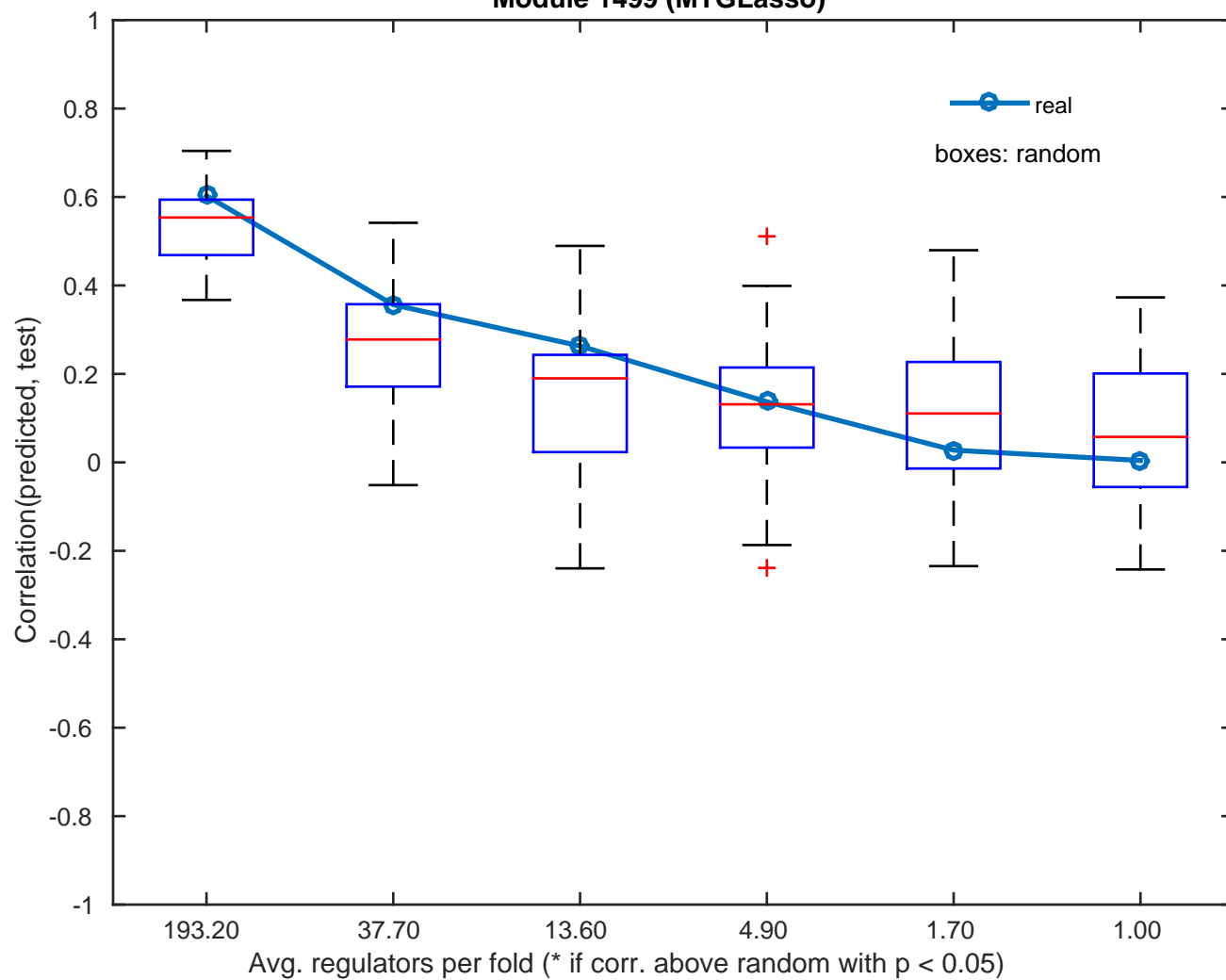

Supplement: S1 Dataset — One plot per module, both species. (GZ) [file pcbi.1005013.s024.gz › human_correlation_vs_lambda/human_module1499_mtglasso_all_lambdas.pdf]

Module 1586 (MTGLasso)

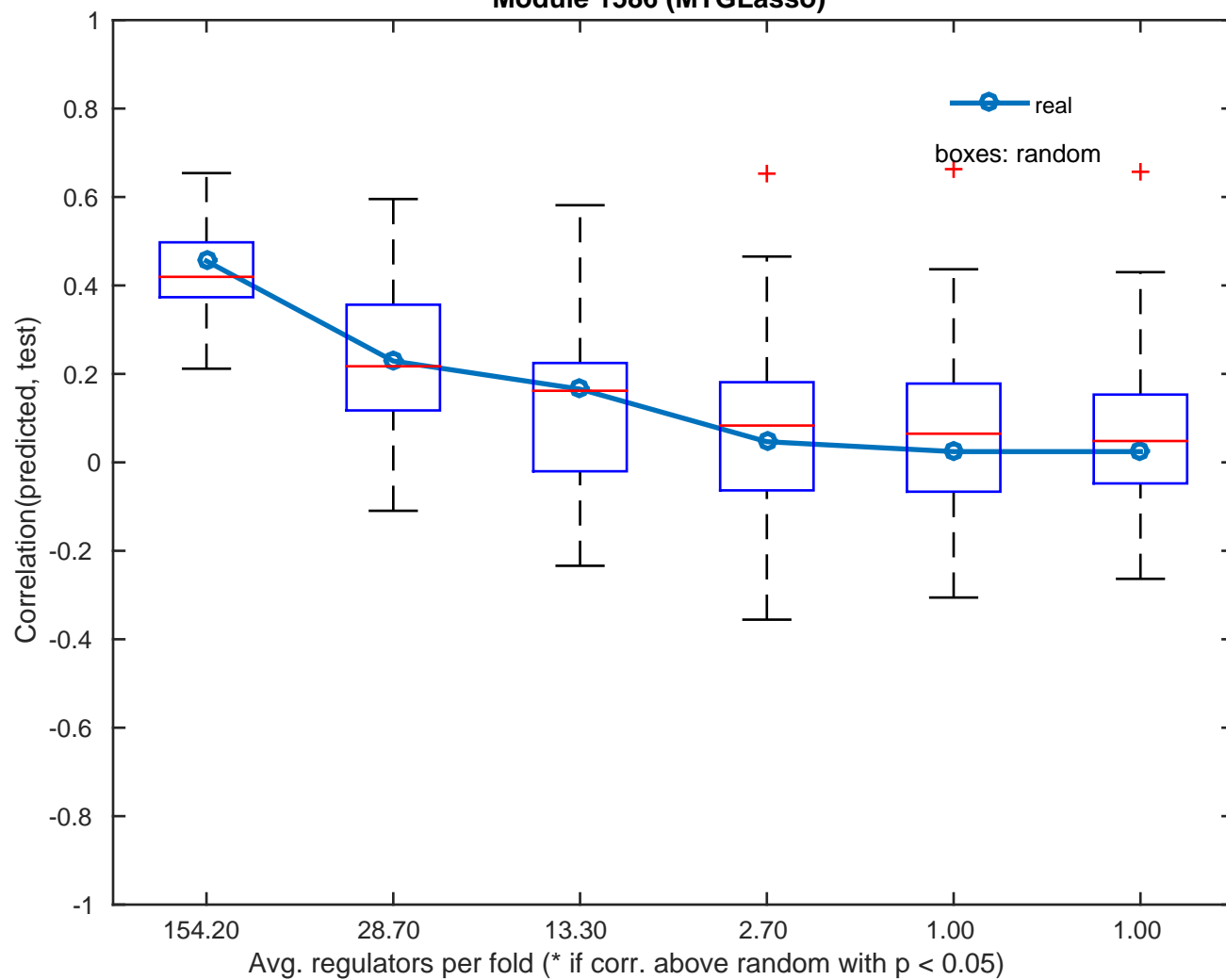

Supplement: S1 Dataset — One plot per module, both species. (GZ) [file pcbi.1005013.s024.gz › human_correlation_vs_lambda/human_module1586_mtglasso_all_lambdas.pdf]

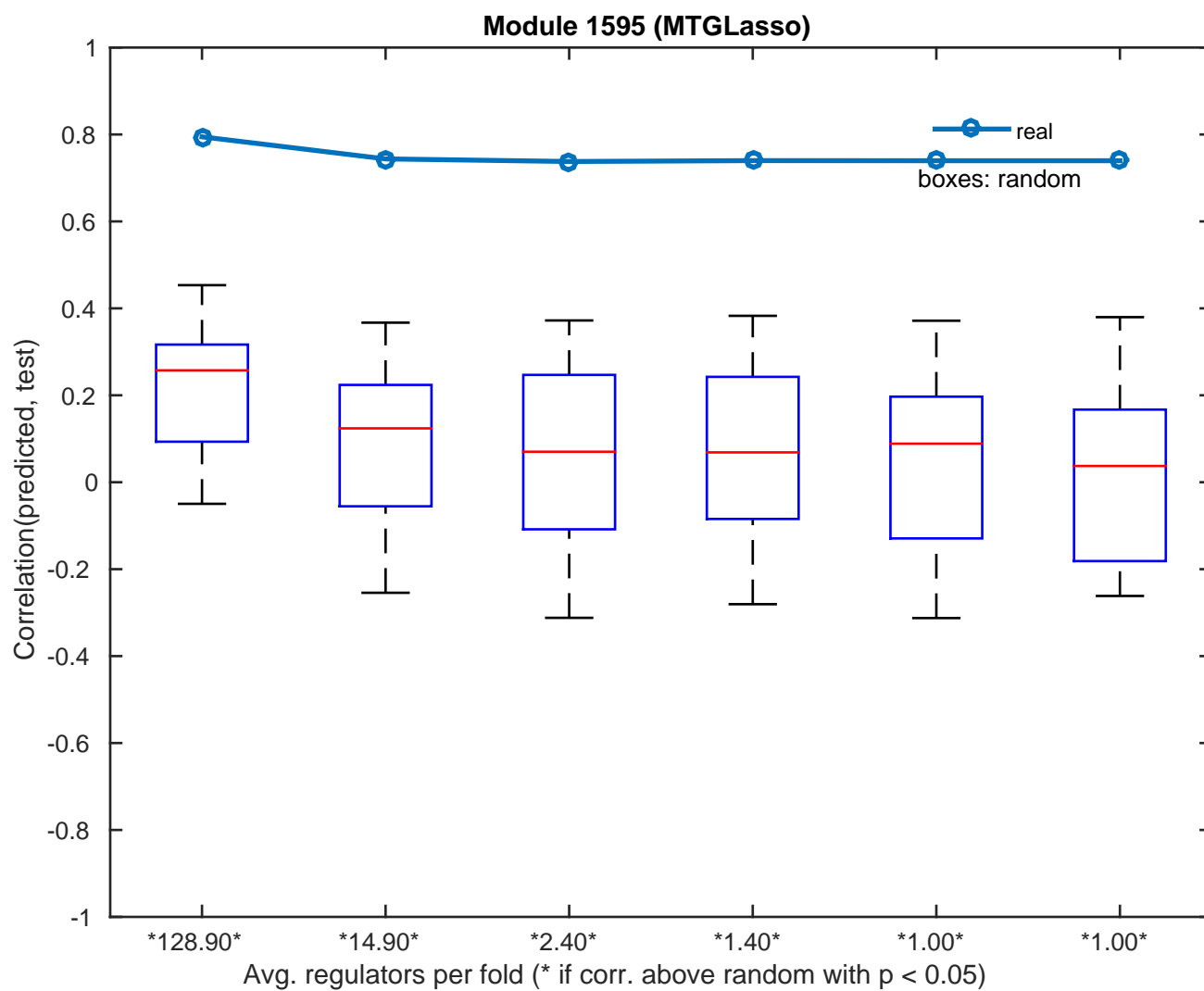

Supplement: S1 Dataset — One plot per module, both species. (GZ) [file pcbi.1005013.s024.gz › human_correlation_vs_lambda/human_module1595_mtglasso_all_lambdas.pdf]

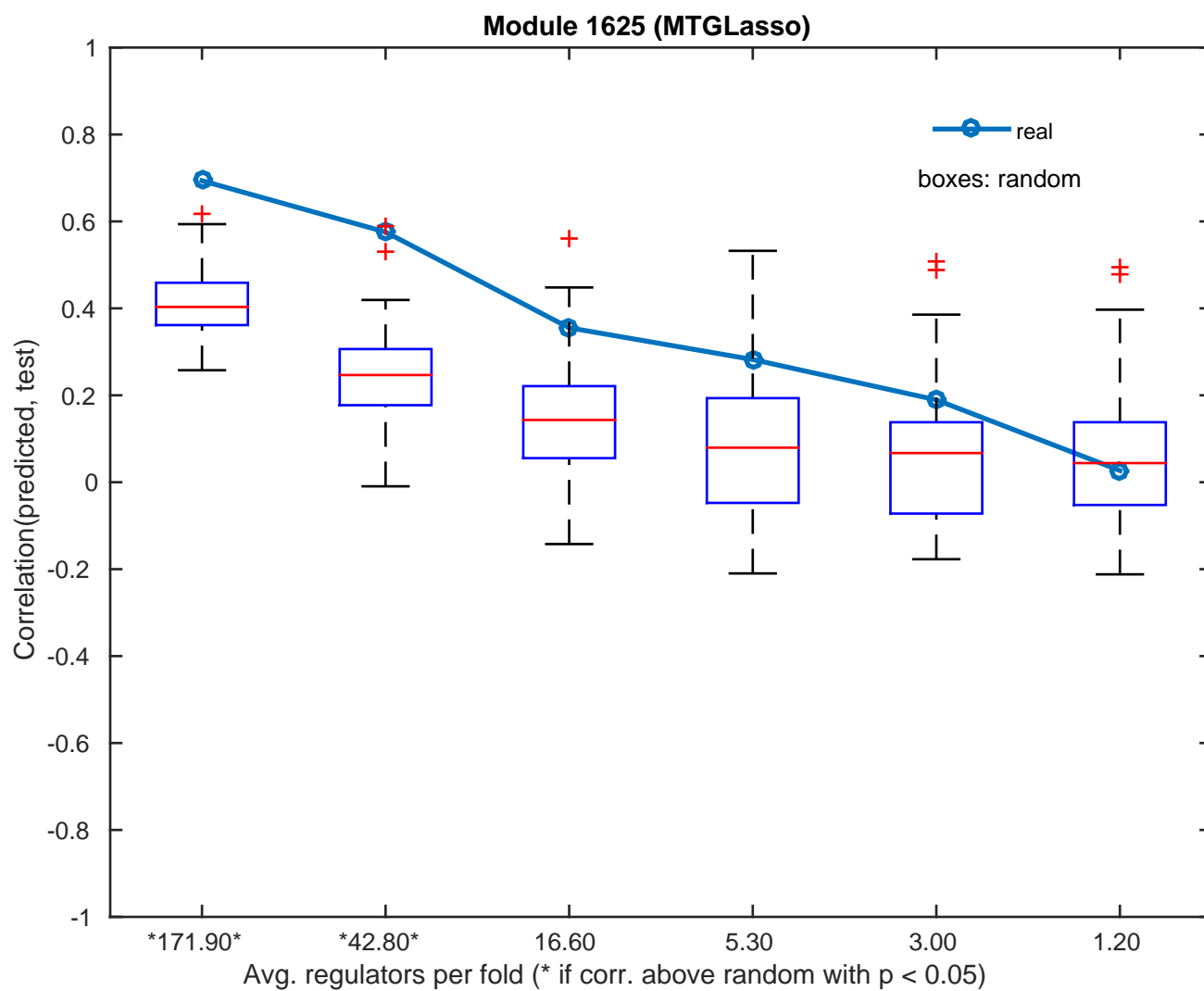

Supplement: S1 Dataset — One plot per module, both species. (GZ) [file pcbi.1005013.s024.gz › human_correlation_vs_lambda/human_module1625_mtglasso_all_lambdas.pdf]

Module 1433 (MTGLasso)

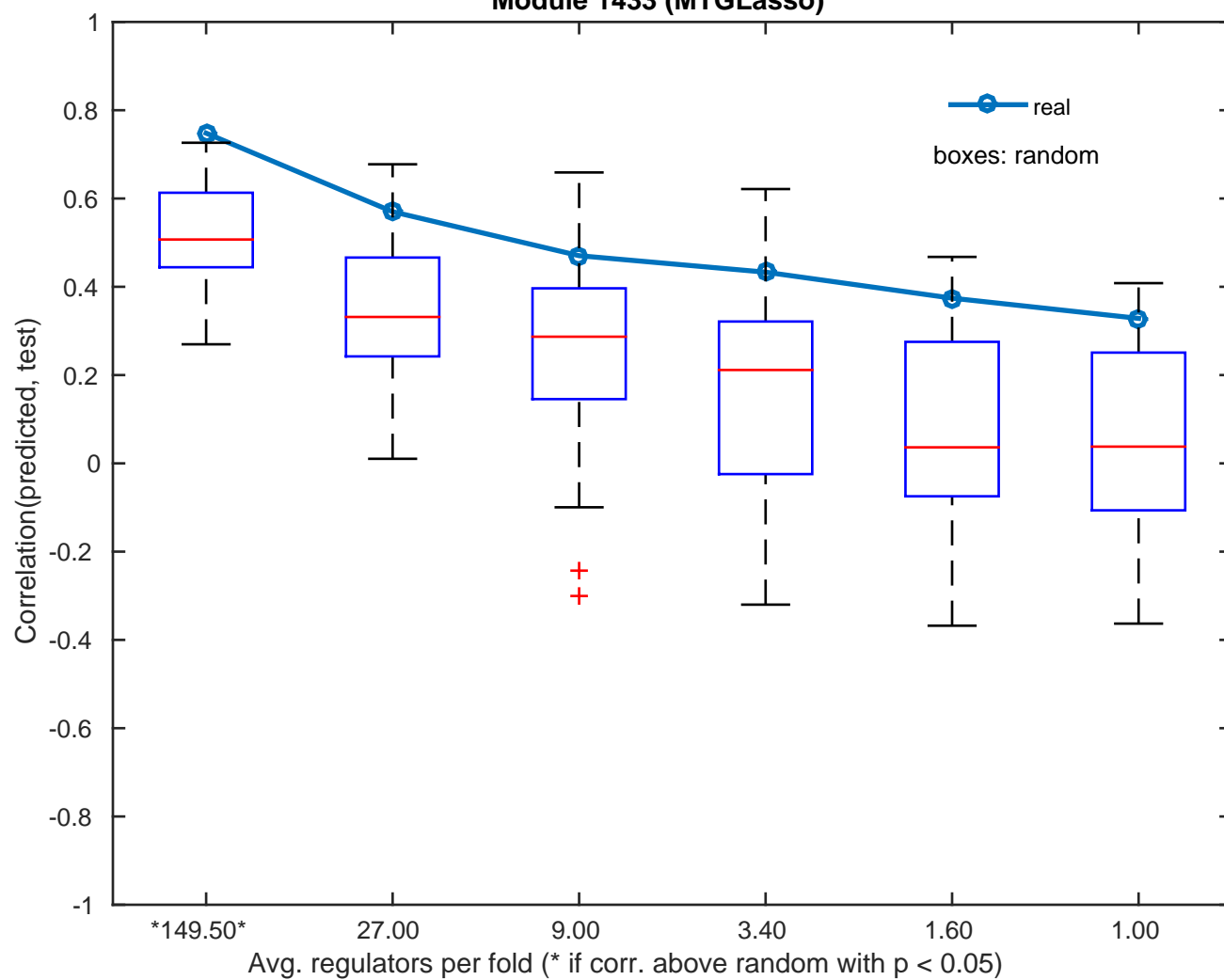

Supplement: S1 Dataset — One plot per module, both species. (GZ) [file pcbi.1005013.s024.gz › human_correlation_vs_lambda/human_module1433_mtglasso_all_lambdas.pdf]

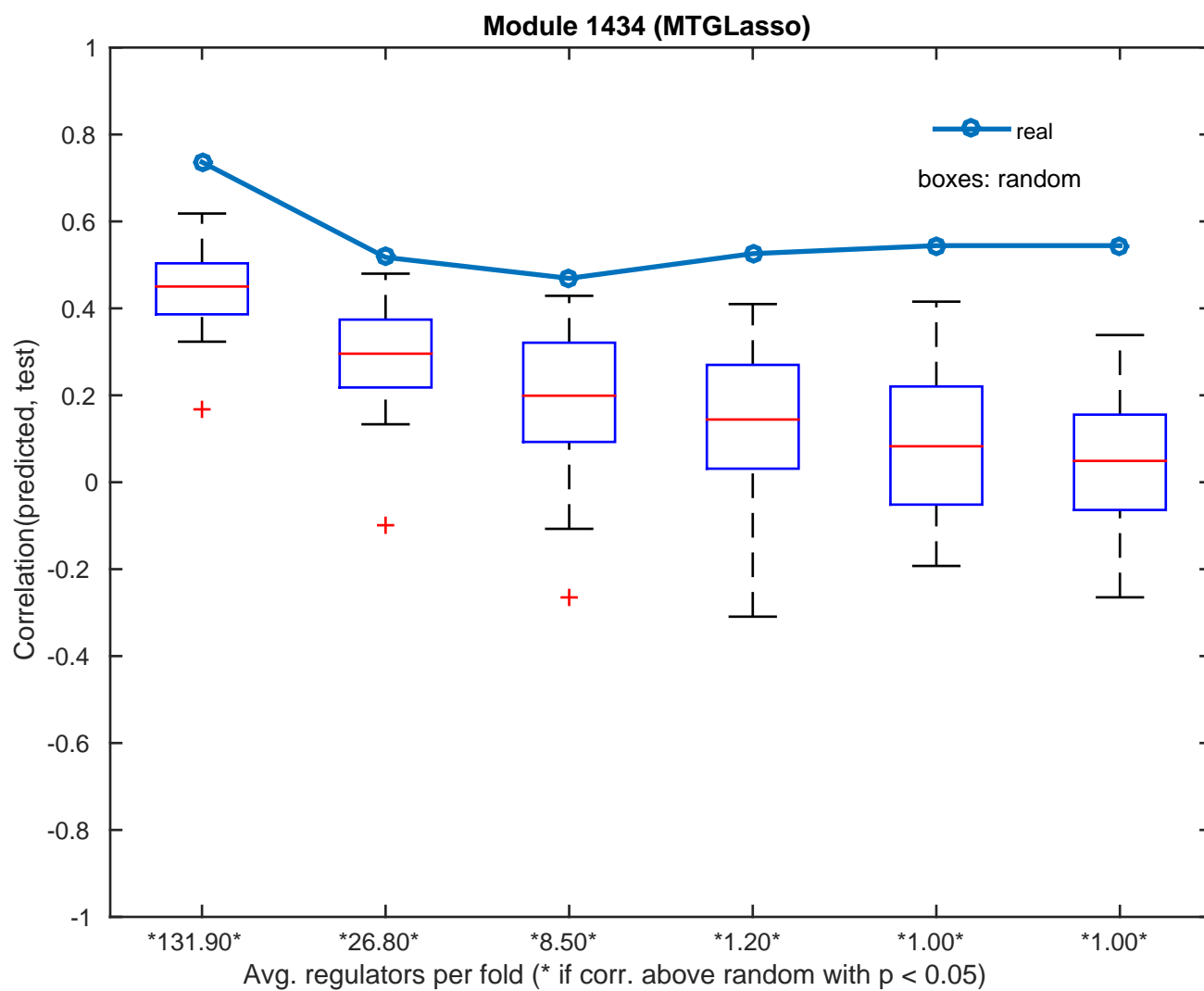

Supplement: S1 Dataset — One plot per module, both species. (GZ) [file pcbi.1005013.s024.gz › human_correlation_vs_lambda/human_module1434_mtglasso_all_lambdas.pdf]

Module 1501 (MTGLasso)

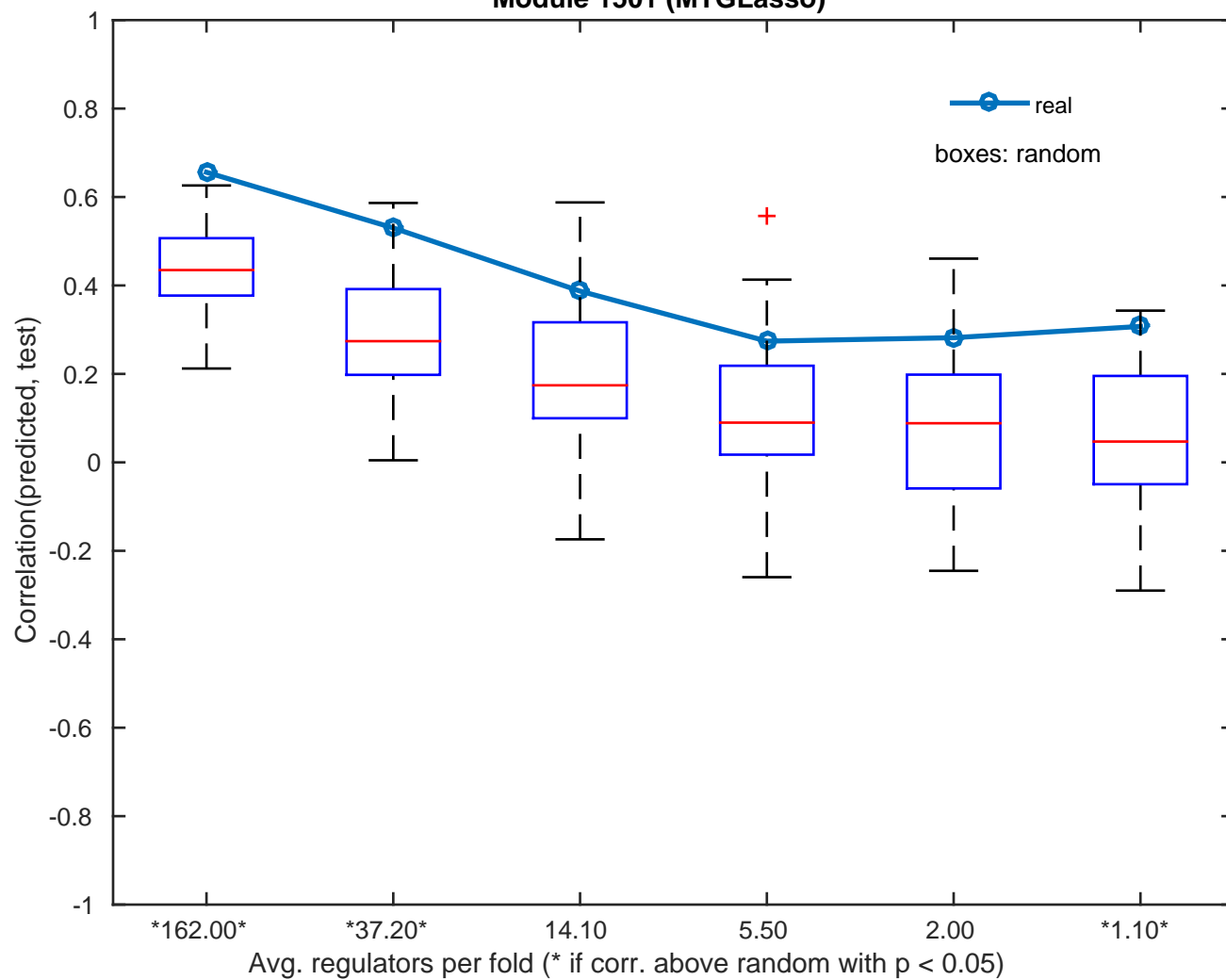

Supplement: S1 Dataset — One plot per module, both species. (GZ) [file pcbi.1005013.s024.gz › human_correlation_vs_lambda/human_module1501_mtglasso_all_lambdas.pdf]

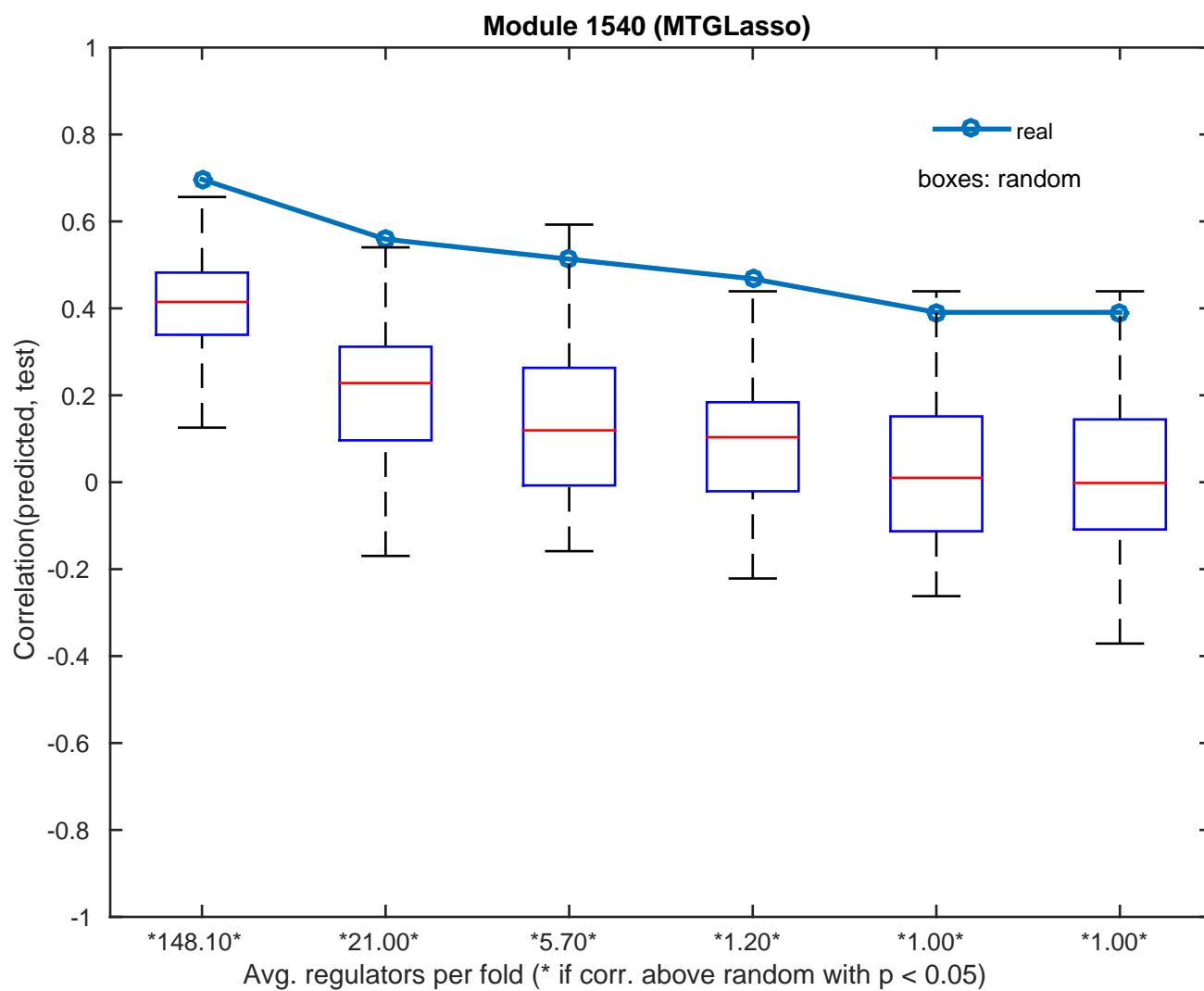

Supplement: S1 Dataset — One plot per module, both species. (GZ) [file pcbi.1005013.s024.gz › human_correlation_vs_lambda/human_module1540_mtglasso_all_lambdas.pdf]

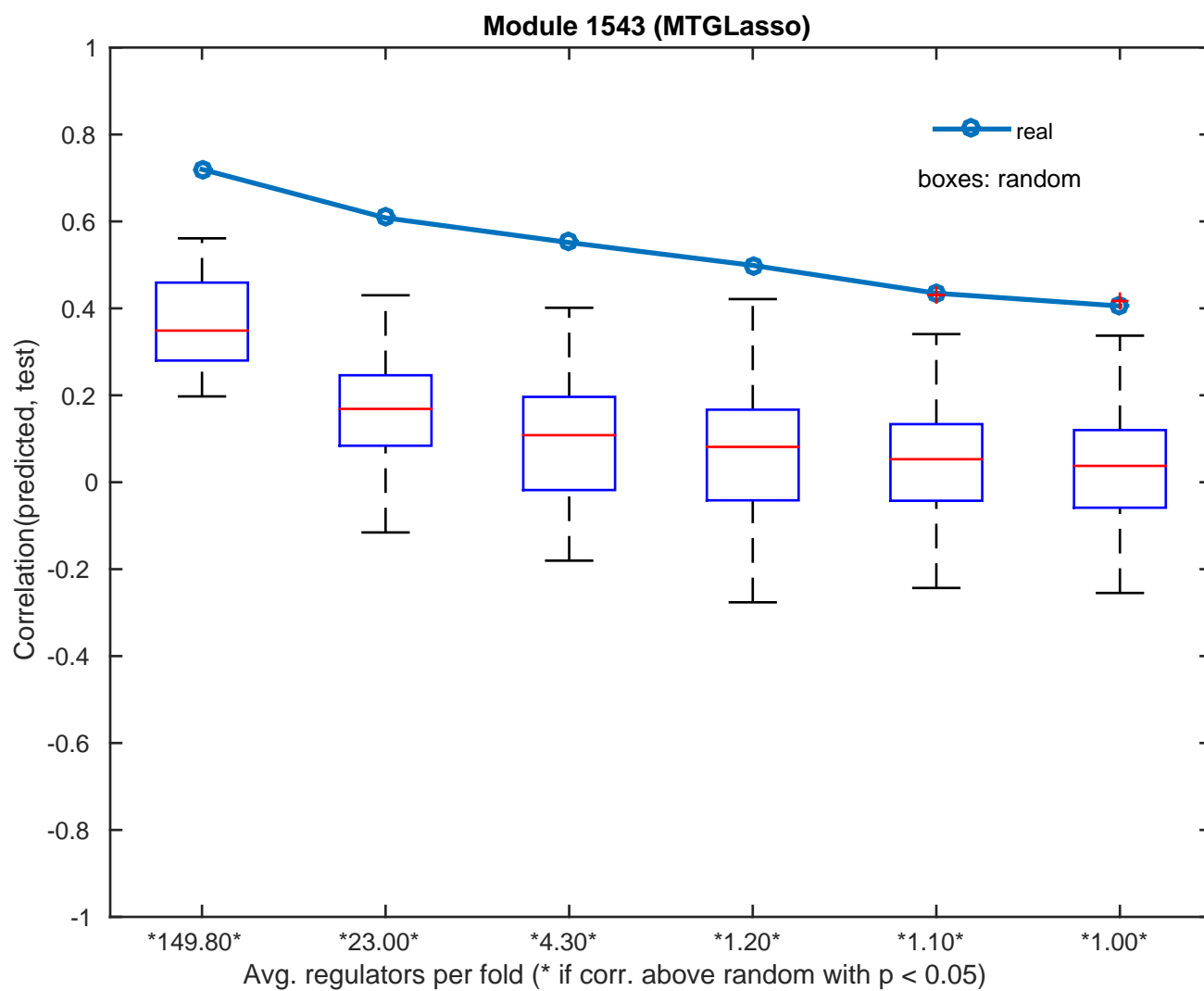

Supplement: S1 Dataset — One plot per module, both species. (GZ) [file pcbi.1005013.s024.gz › human_correlation_vs_lambda/human_module1543_mtglasso_all_lambdas.pdf]

Module 1588 (MTGLasso)

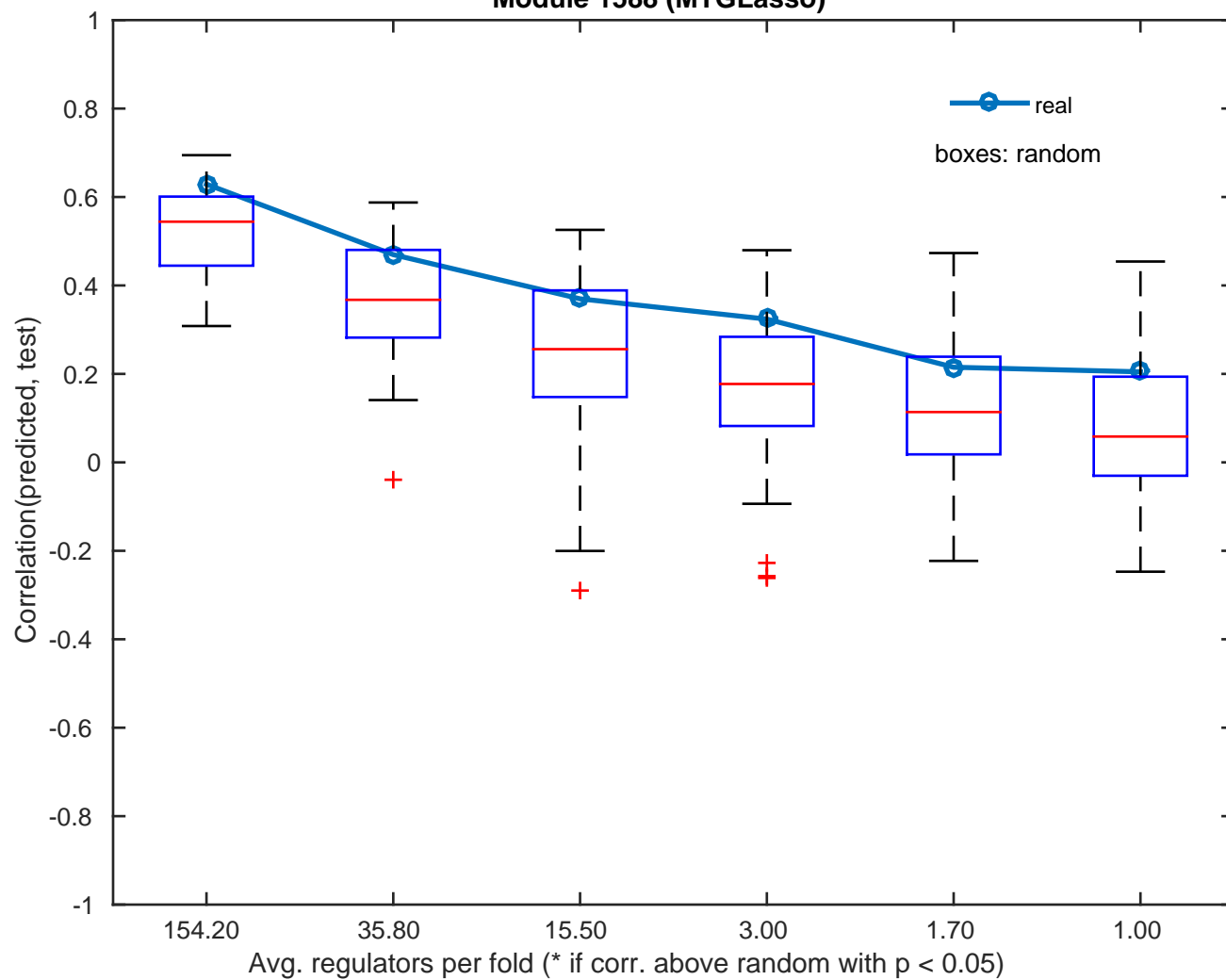

Supplement: S1 Dataset — One plot per module, both species. (GZ) [file pcbi.1005013.s024.gz › human_correlation_vs_lambda/human_module1588_mtglasso_all_lambdas.pdf]

Module 1485 (MTGLasso)

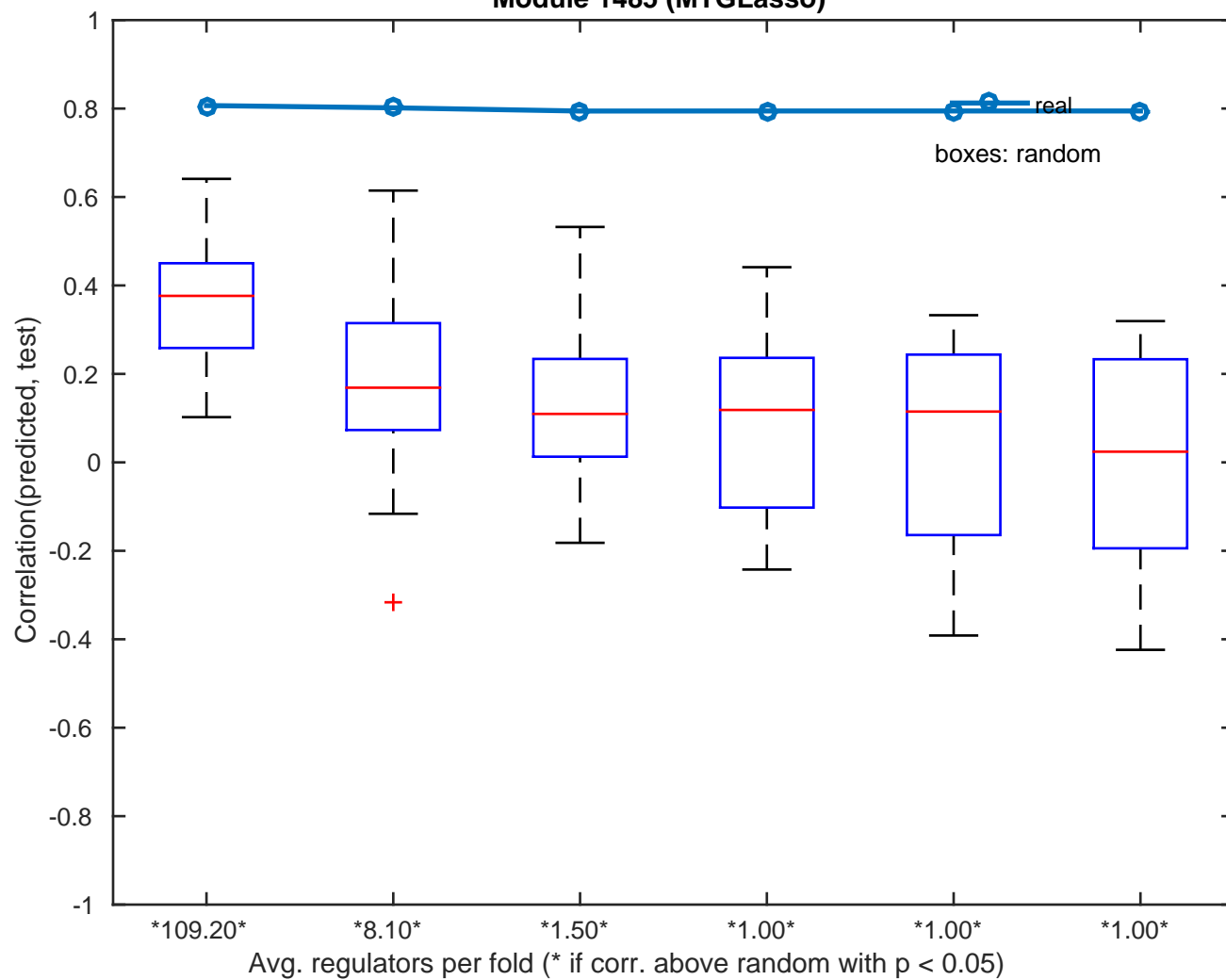

Supplement: S1 Dataset — One plot per module, both species. (GZ) [file pcbi.1005013.s024.gz › human_correlation_vs_lambda/human_module1485_mtglasso_all_lambdas.pdf]

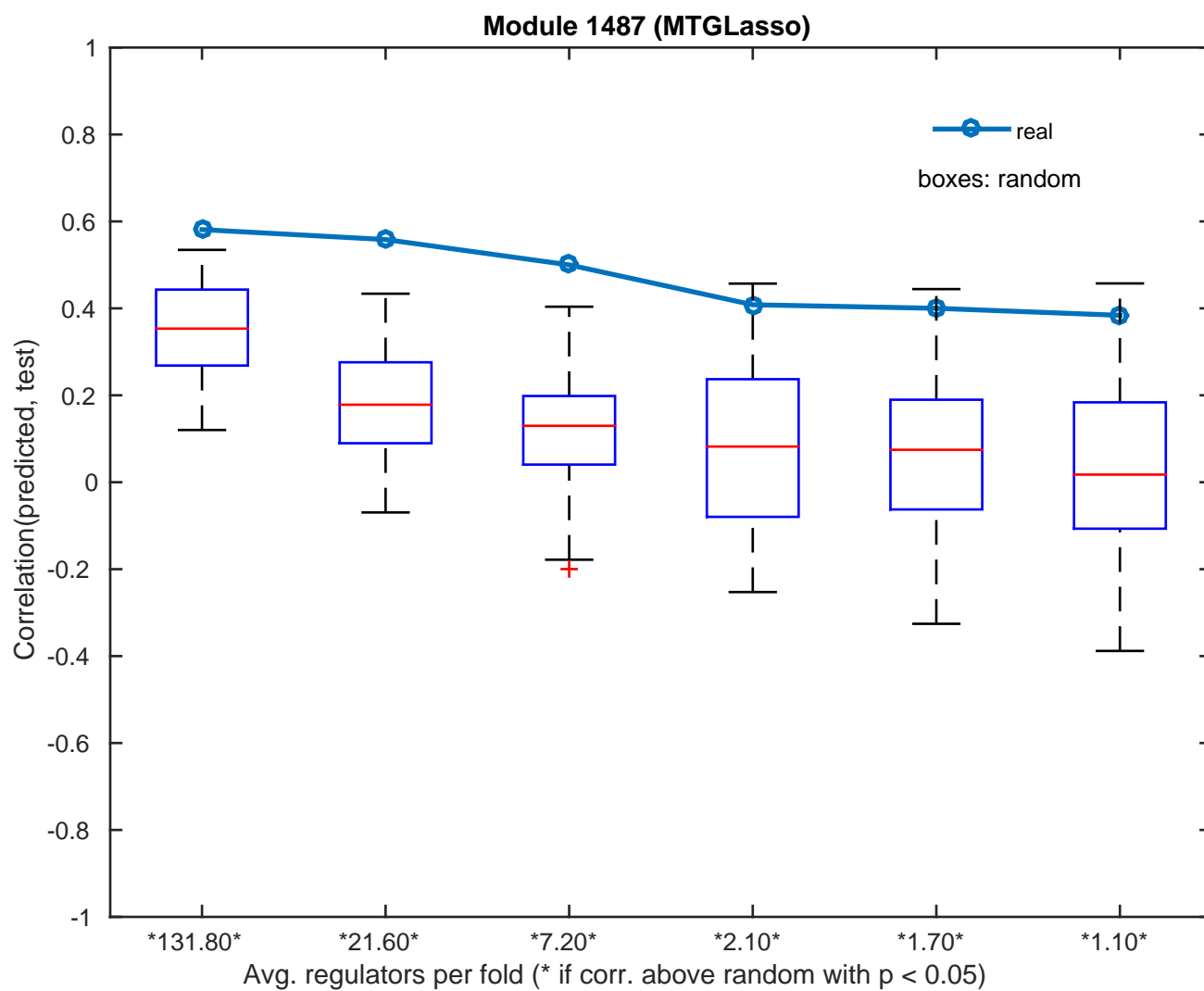

Supplement: S1 Dataset — One plot per module, both species. (GZ) [file pcbi.1005013.s024.gz › human_correlation_vs_lambda/human_module1487_mtglasso_all_lambdas.pdf]

Module 1500 (MTGLasso)

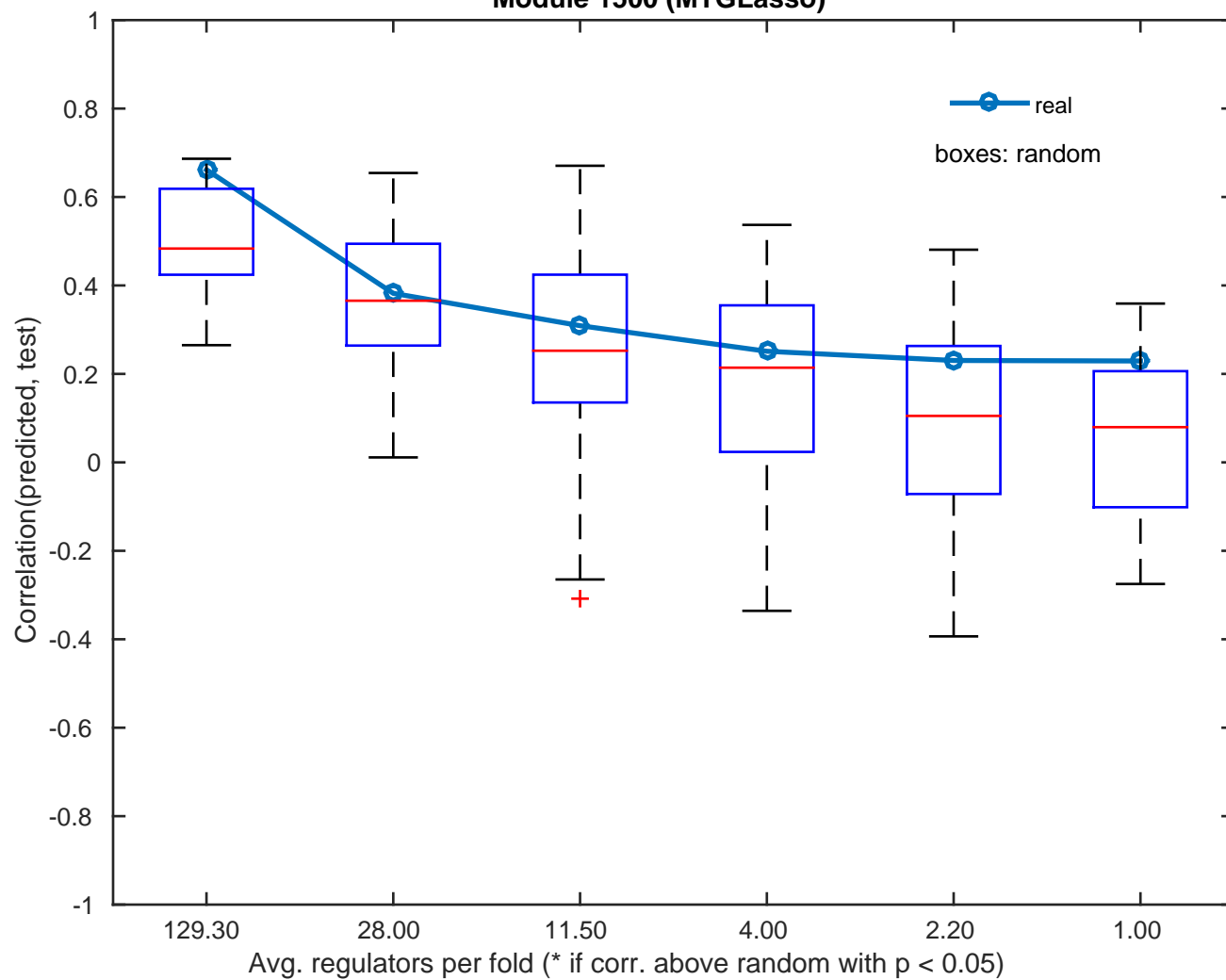

Supplement: S1 Dataset — One plot per module, both species. (GZ) [file pcbi.1005013.s024.gz › human_correlation_vs_lambda/human_module1500_mtglasso_all_lambdas.pdf]

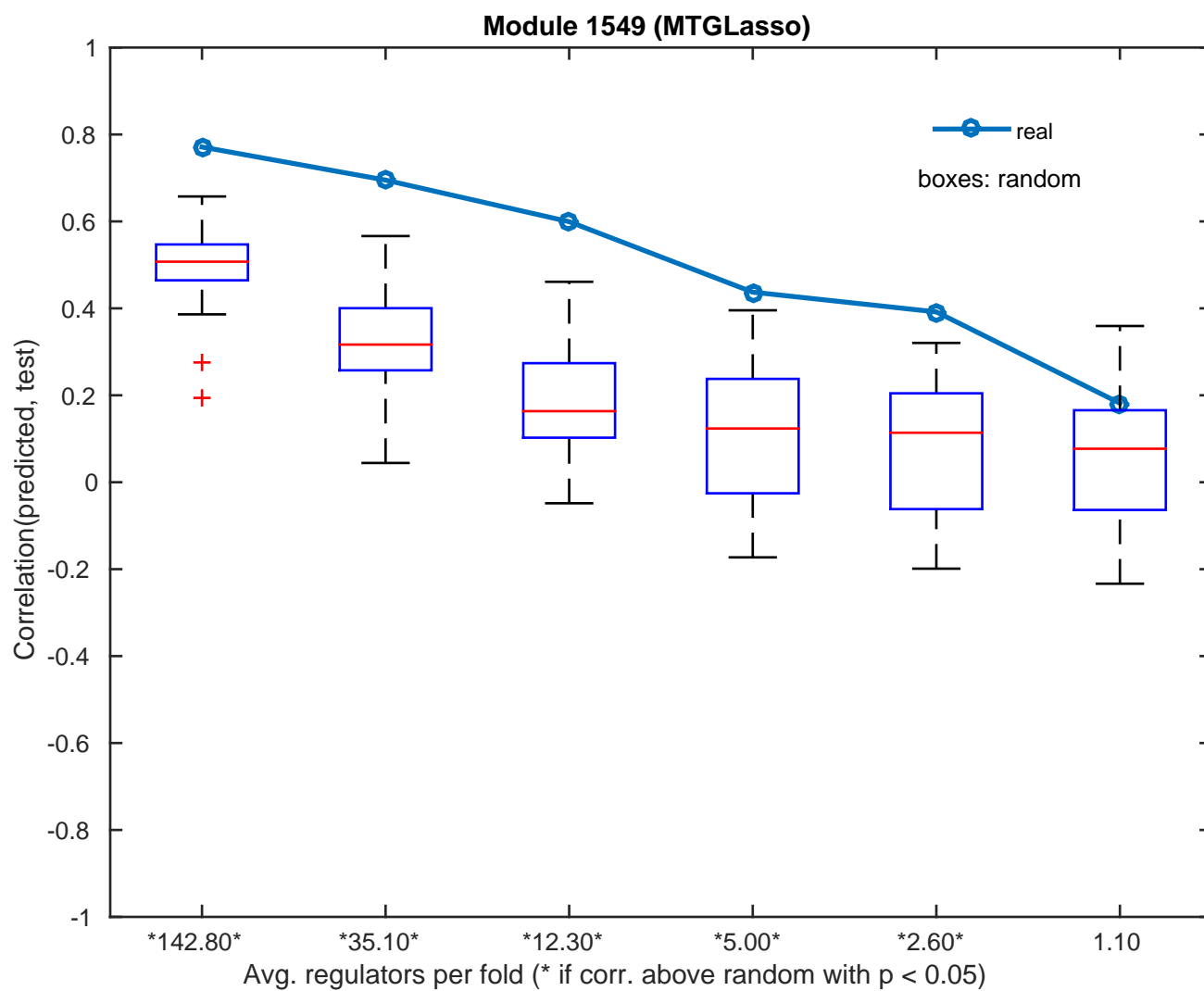

Supplement: S1 Dataset — One plot per module, both species. (GZ) [file pcbi.1005013.s024.gz › human_correlation_vs_lambda/human_module1549_mtglasso_all_lambdas.pdf]

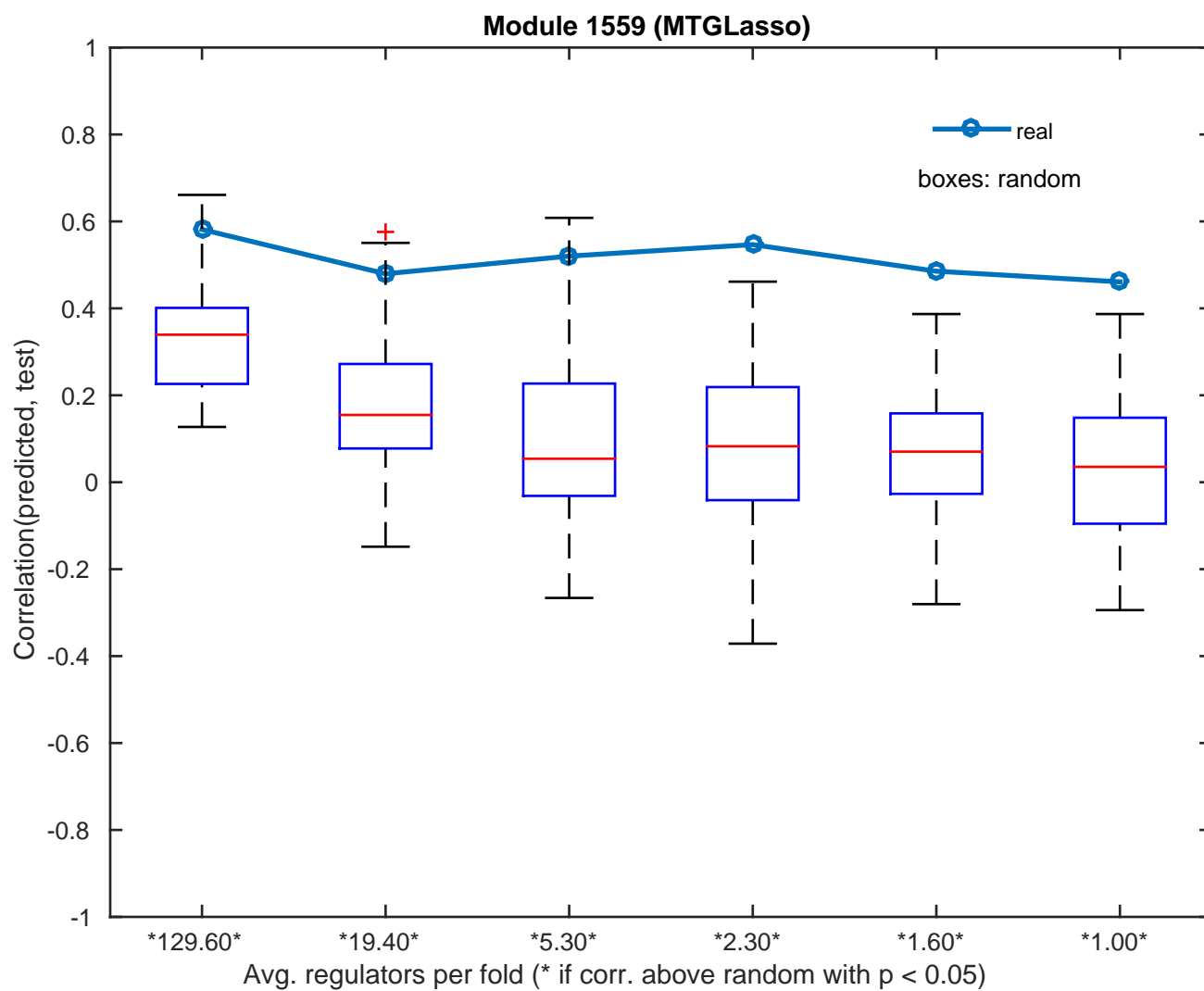

Supplement: S1 Dataset — One plot per module, both species. (GZ) [file pcbi.1005013.s024.gz › human_correlation_vs_lambda/human_module1559_mtglasso_all_lambdas.pdf]

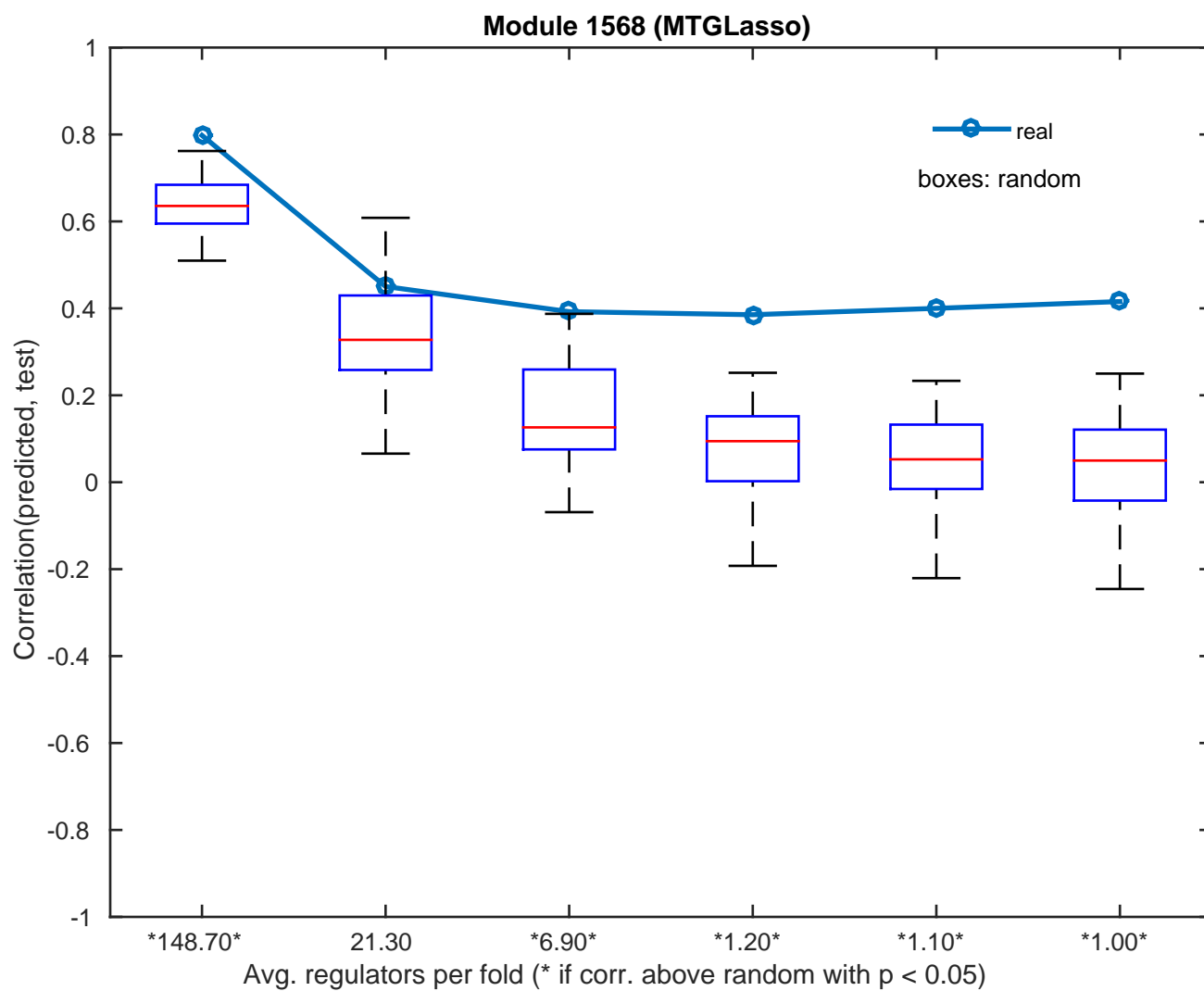

Supplement: S1 Dataset — One plot per module, both species. (GZ) [file pcbi.1005013.s024.gz › human_correlation_vs_lambda/human_module1568_mtglasso_all_lambdas.pdf]

Module 1571 (MTGLasso)

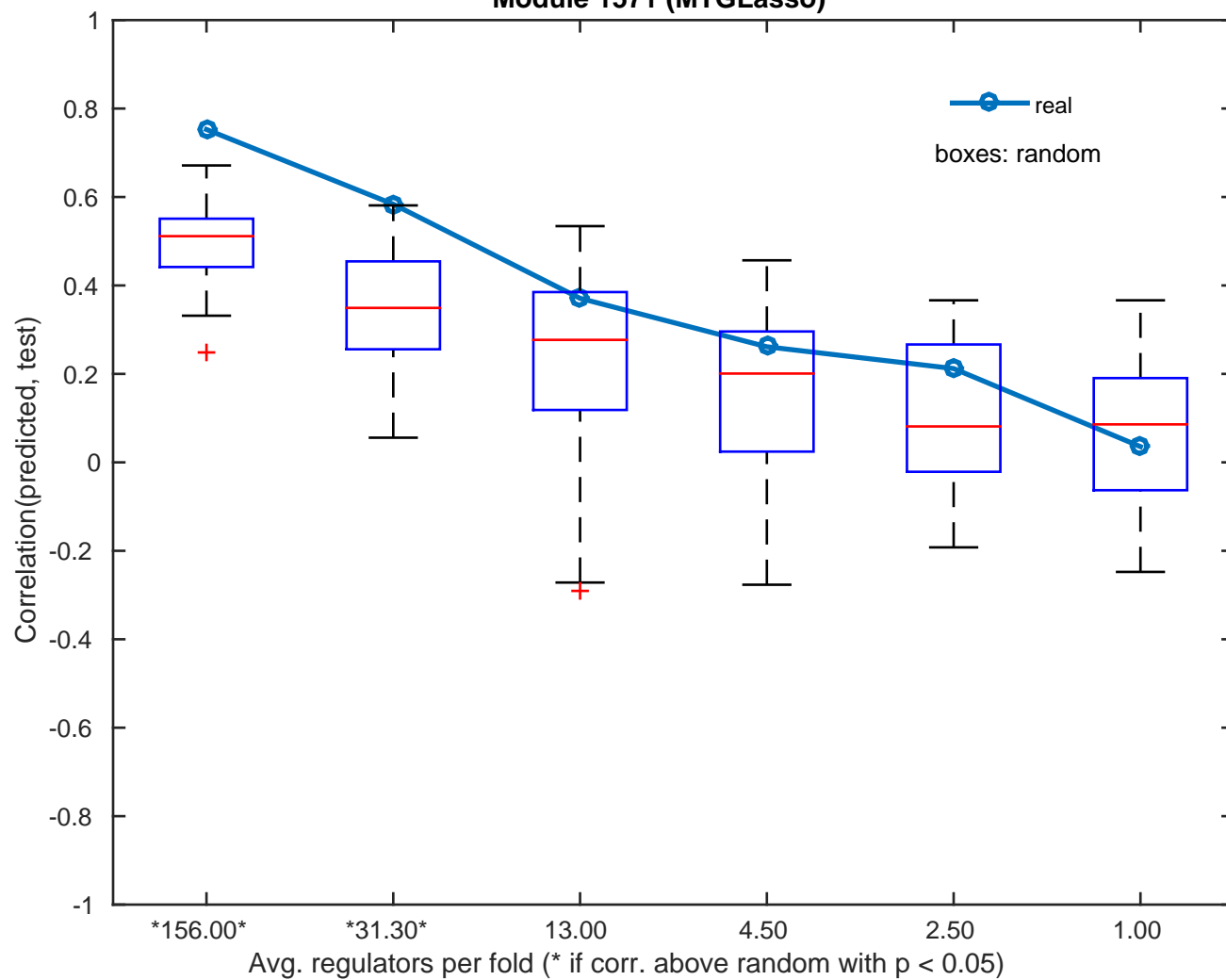

Supplement: S1 Dataset — One plot per module, both species. (GZ) [file pcbi.1005013.s024.gz › human_correlation_vs_lambda/human_module1571_mtglasso_all_lambdas.pdf]

Module 1578 (MTGLasso)

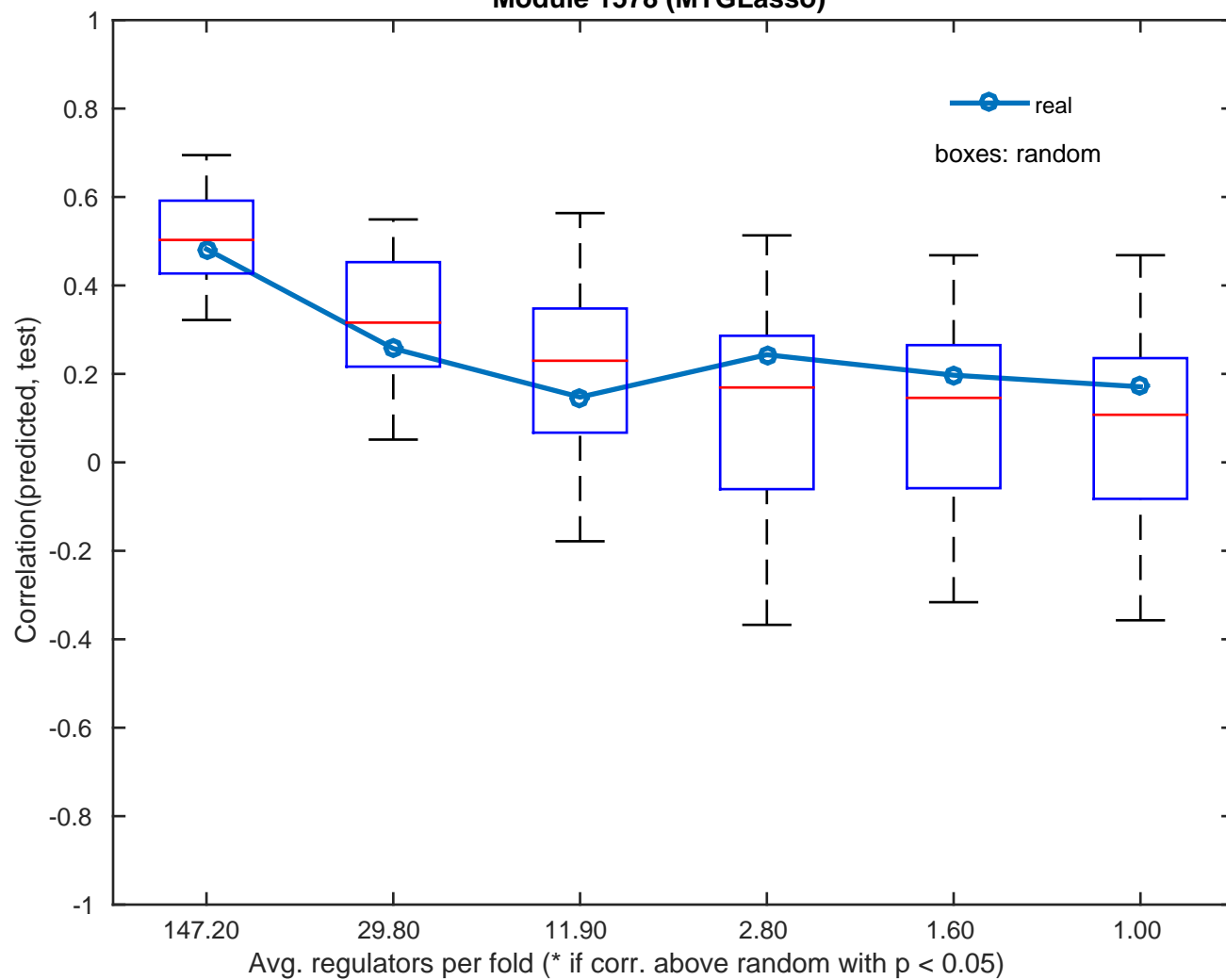

Supplement: S1 Dataset — One plot per module, both species. (GZ) [file pcbi.1005013.s024.gz › human_correlation_vs_lambda/human_module1578_mtglasso_all_lambdas.pdf]

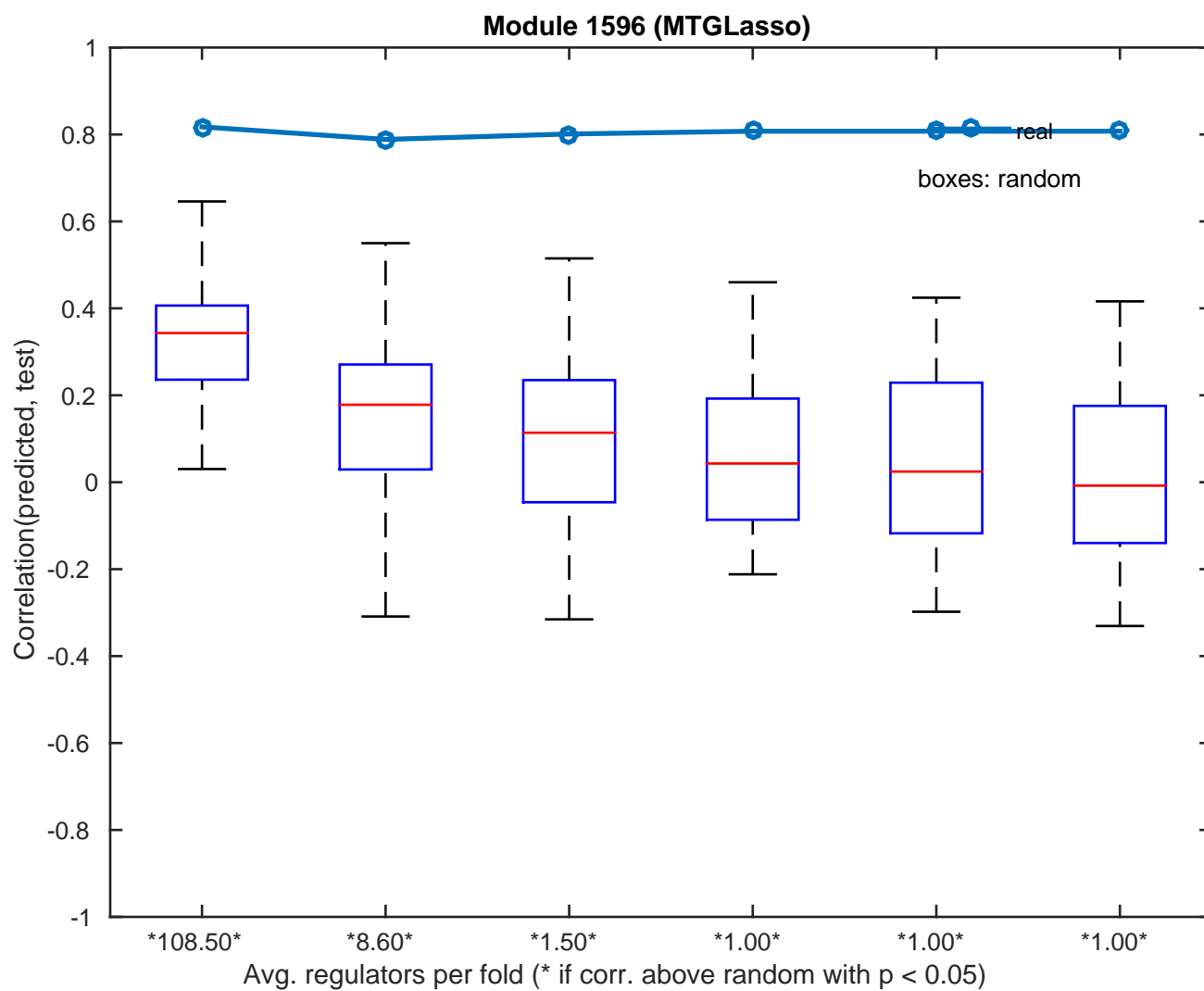

Supplement: S1 Dataset — One plot per module, both species. (GZ) [file pcbi.1005013.s024.gz › human_correlation_vs_lambda/human_module1596_mtglasso_all_lambdas.pdf]

Module 3029 (MTGLasso)

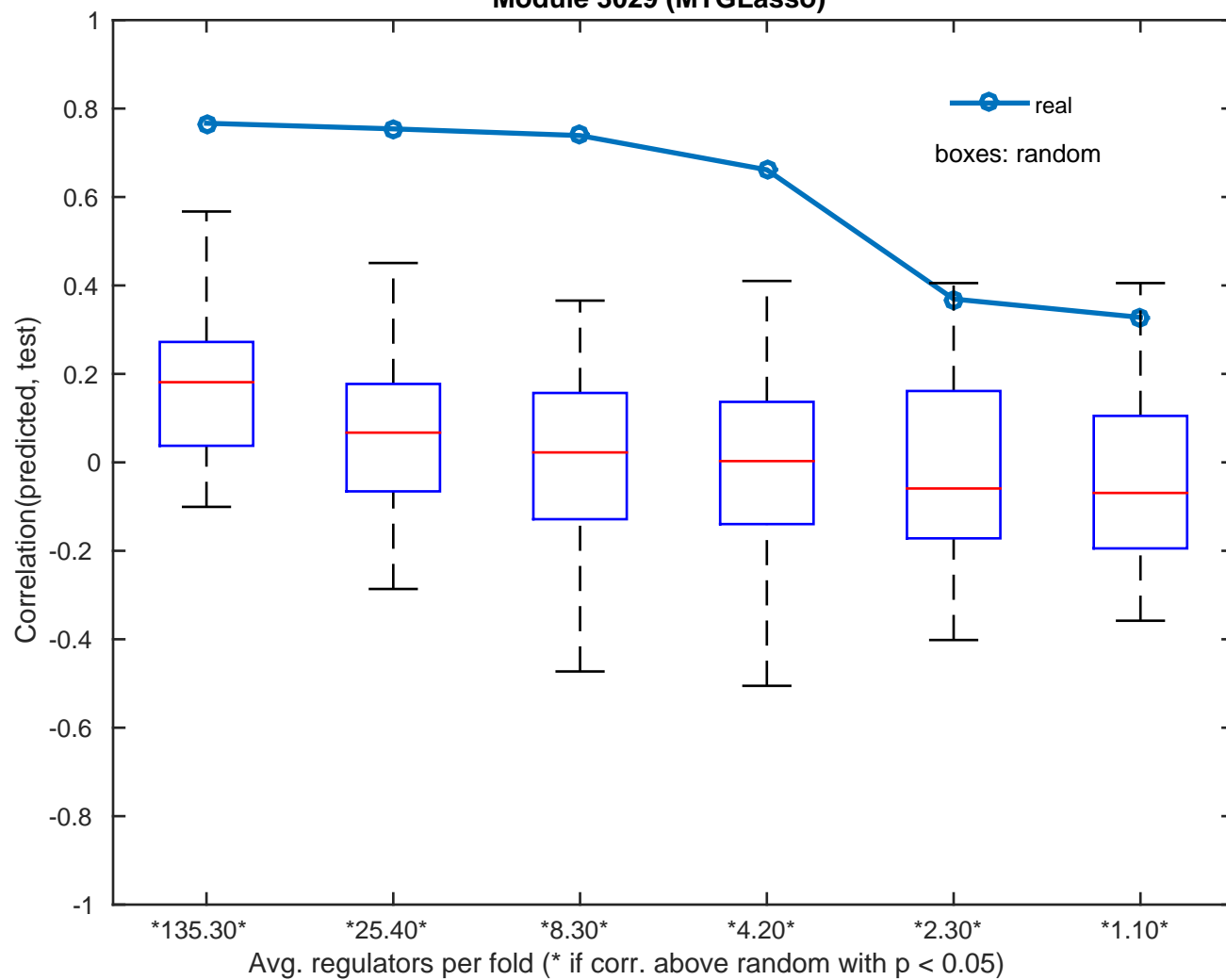

Supplement: S1 Dataset — One plot per module, both species. (GZ) [file pcbi.1005013.s024.gz › mouse_correlation_vs_lambda/mouse_module3029_mtglasso_all_lambdas.pdf]

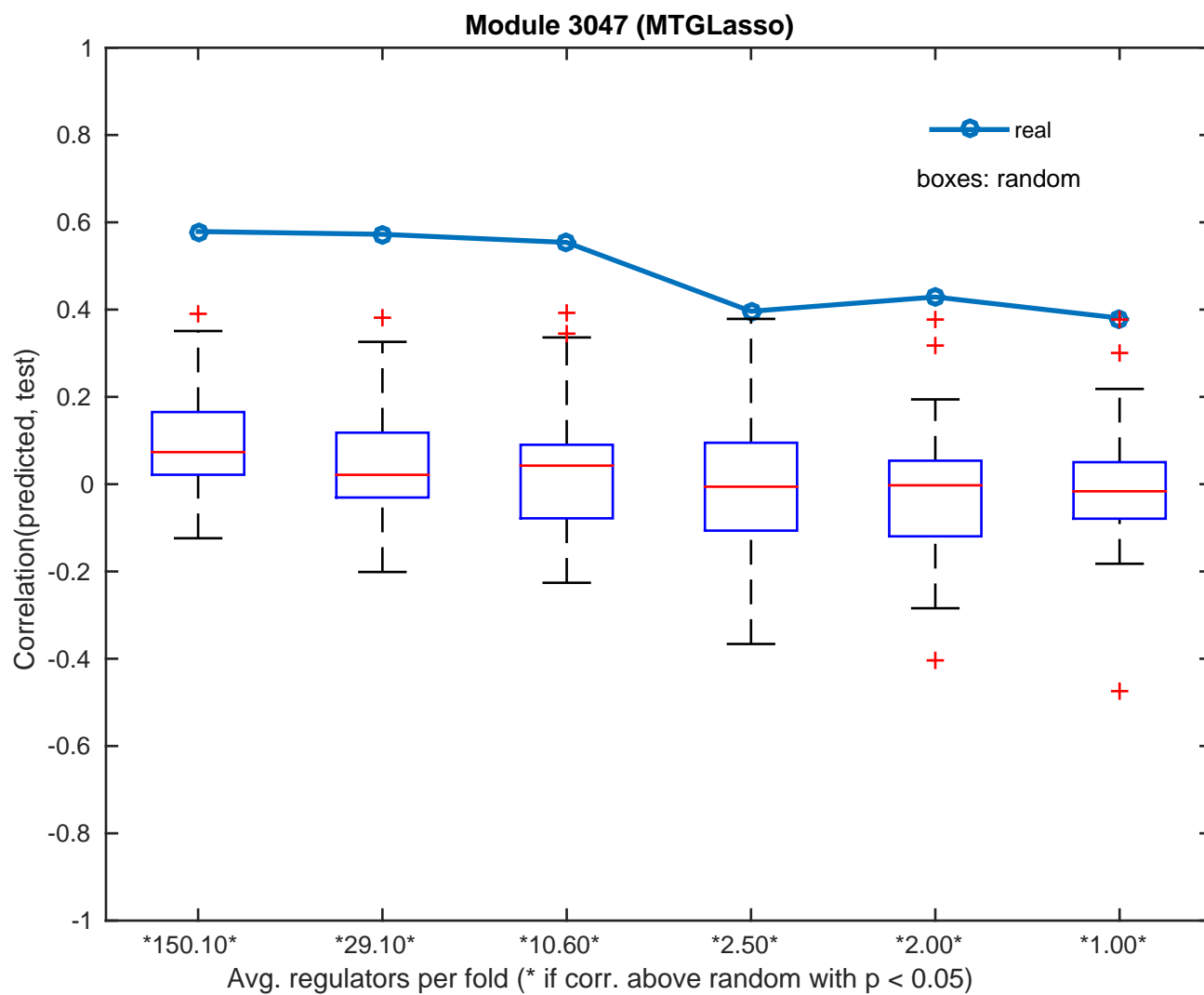

Supplement: S1 Dataset — One plot per module, both species. (GZ) [file pcbi.1005013.s024.gz › mouse_correlation_vs_lambda/mouse_module3047_mtglasso_all_lambdas.pdf]

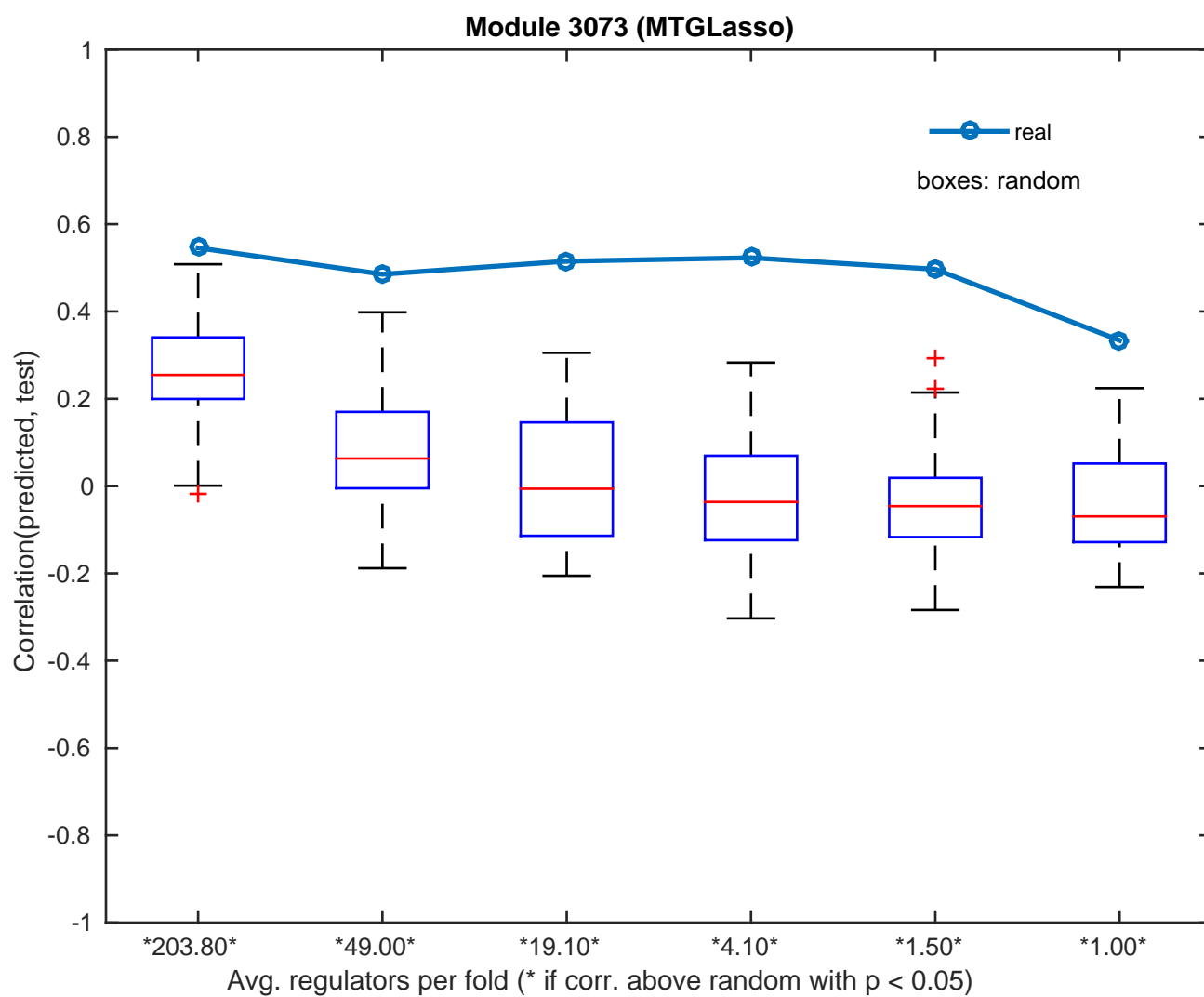

Supplement: S1 Dataset — One plot per module, both species. (GZ) [file pcbi.1005013.s024.gz › mouse_correlation_vs_lambda/mouse_module3073_mtglasso_all_lambdas.pdf]

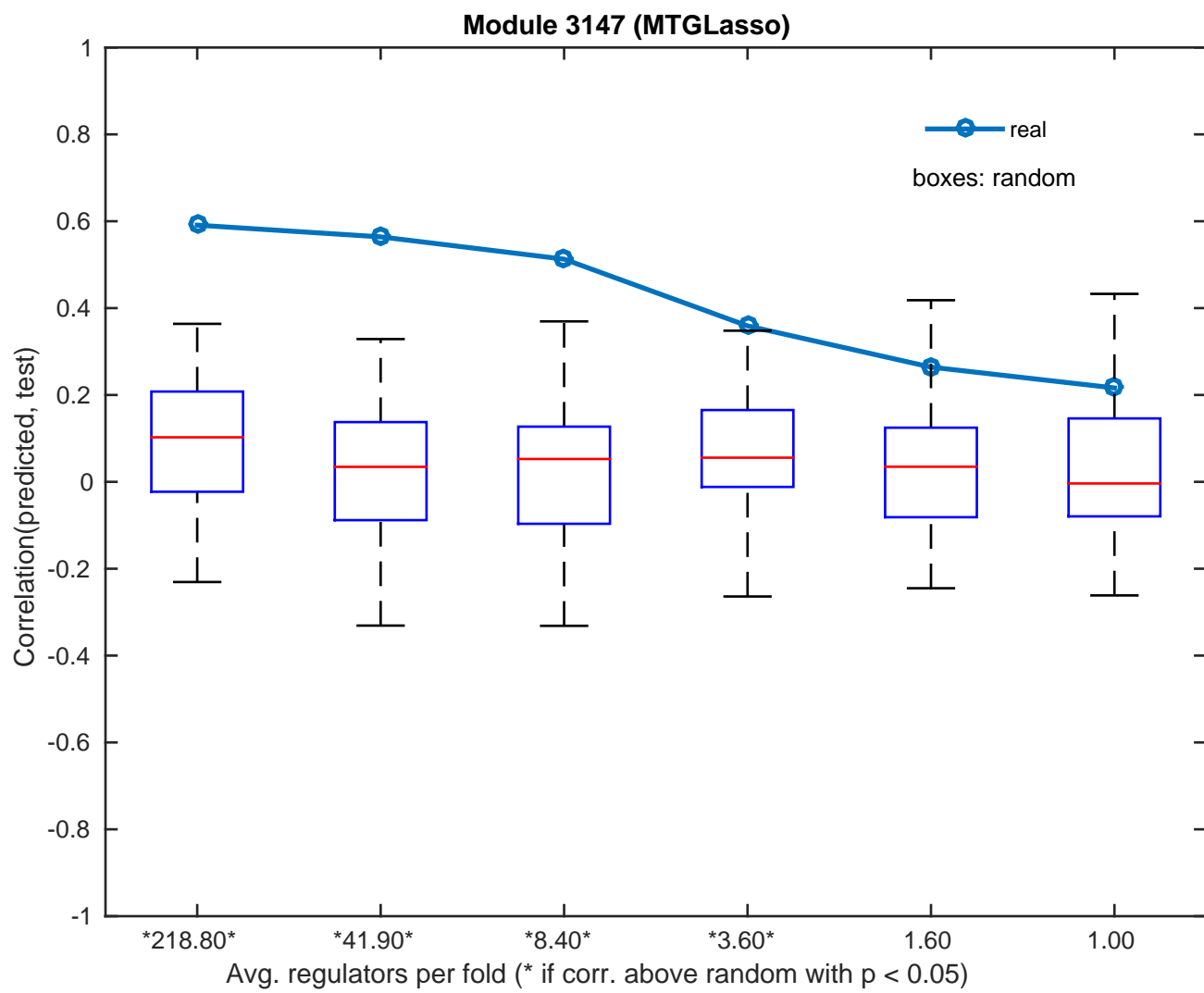

Supplement: S1 Dataset — One plot per module, both species. (GZ) [file pcbi.1005013.s024.gz › mouse_correlation_vs_lambda/mouse_module3147_mtglasso_all_lambdas.pdf]

Module 3155 (MTGLasso)

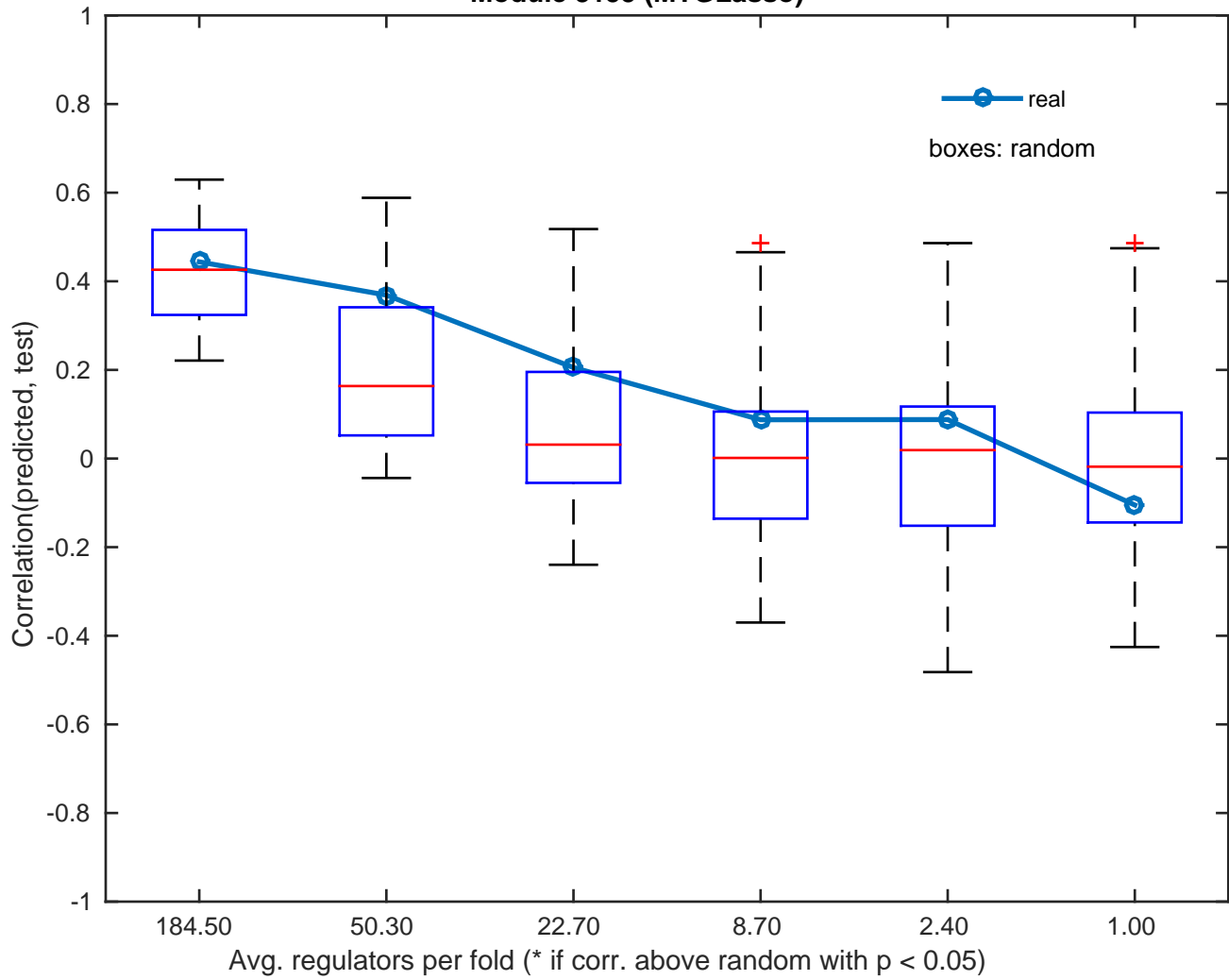

Supplement: S1 Dataset — One plot per module, both species. (GZ) [file pcbi.1005013.s024.gz › mouse_correlation_vs_lambda/mouse_module3155_mtglasso_all_lambdas.pdf]

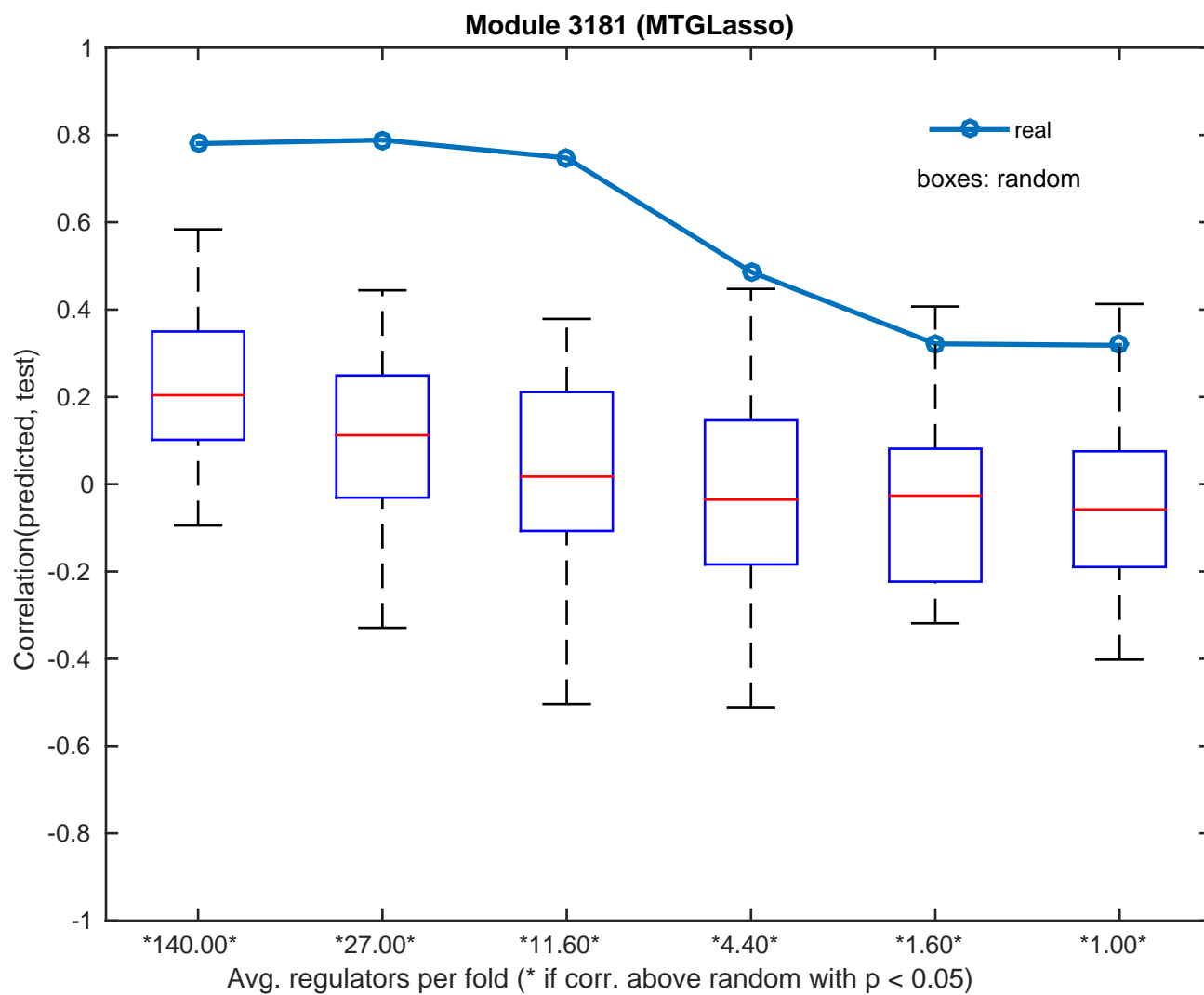

Supplement: S1 Dataset — One plot per module, both species. (GZ) [file pcbi.1005013.s024.gz › mouse_correlation_vs_lambda/mouse_module3181_mtglasso_all_lambdas.pdf]

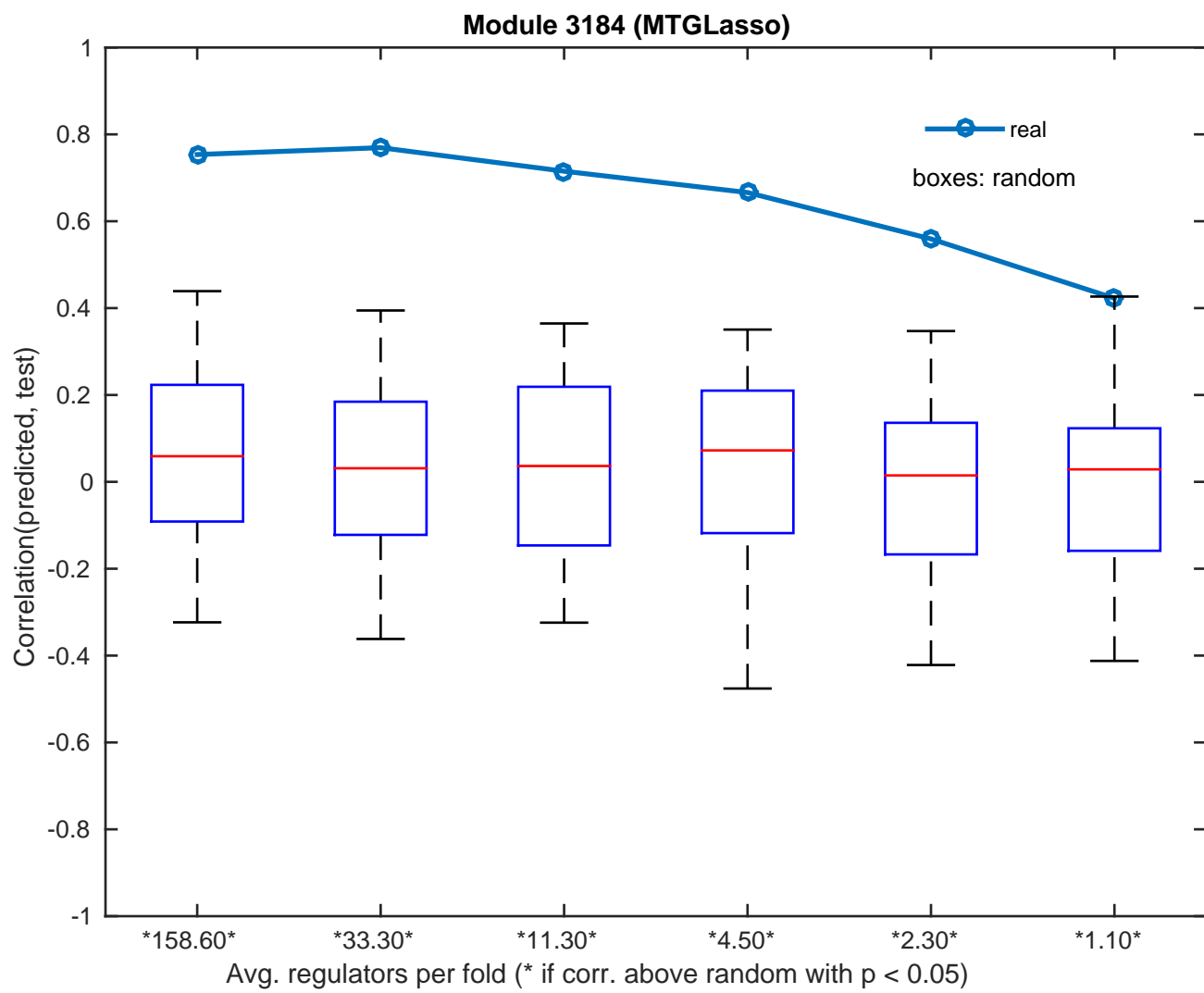

Supplement: S1 Dataset — One plot per module, both species. (GZ) [file pcbi.1005013.s024.gz › mouse_correlation_vs_lambda/mouse_module3184_mtglasso_all_lambdas.pdf]

Module 3205 (MTGLasso)

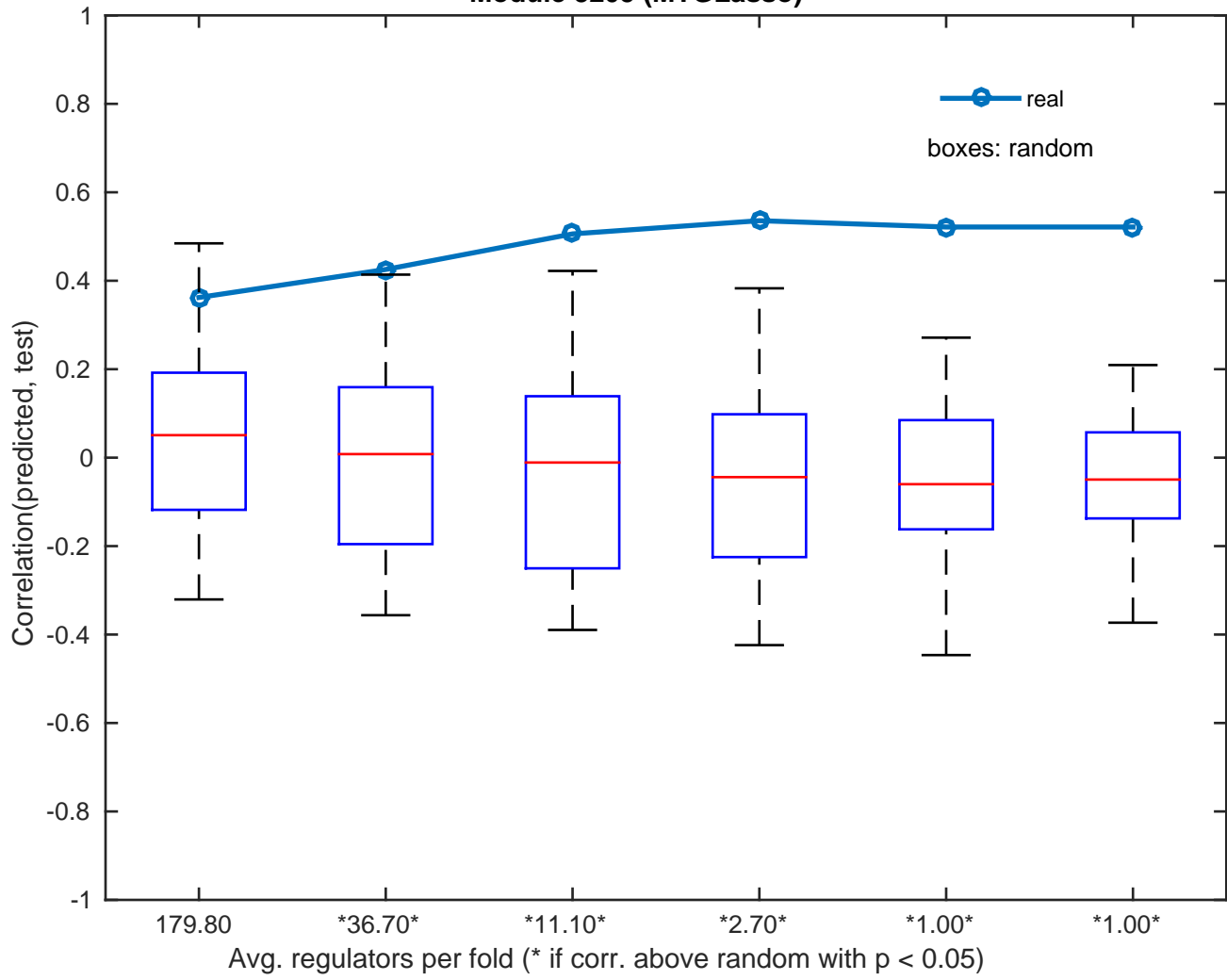

Supplement: S1 Dataset — One plot per module, both species. (GZ) [file pcbi.1005013.s024.gz › mouse_correlation_vs_lambda/mouse_module3205_mtglasso_all_lambdas.pdf]

Module 2977 (MTGLasso)

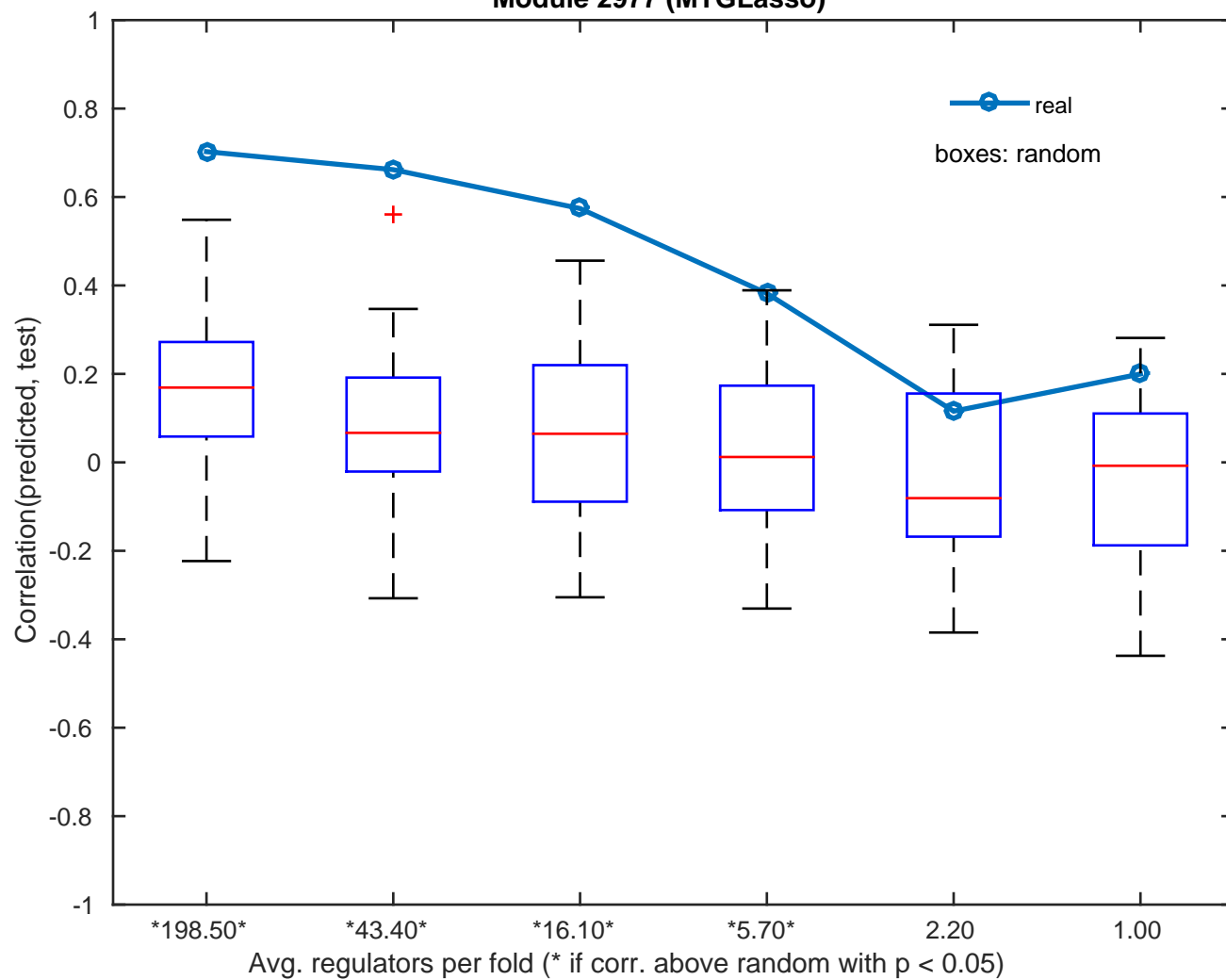

Supplement: S1 Dataset — One plot per module, both species. (GZ) [file pcbi.1005013.s024.gz › mouse_correlation_vs_lambda/mouse_module2977_mtglasso_all_lambdas.pdf]

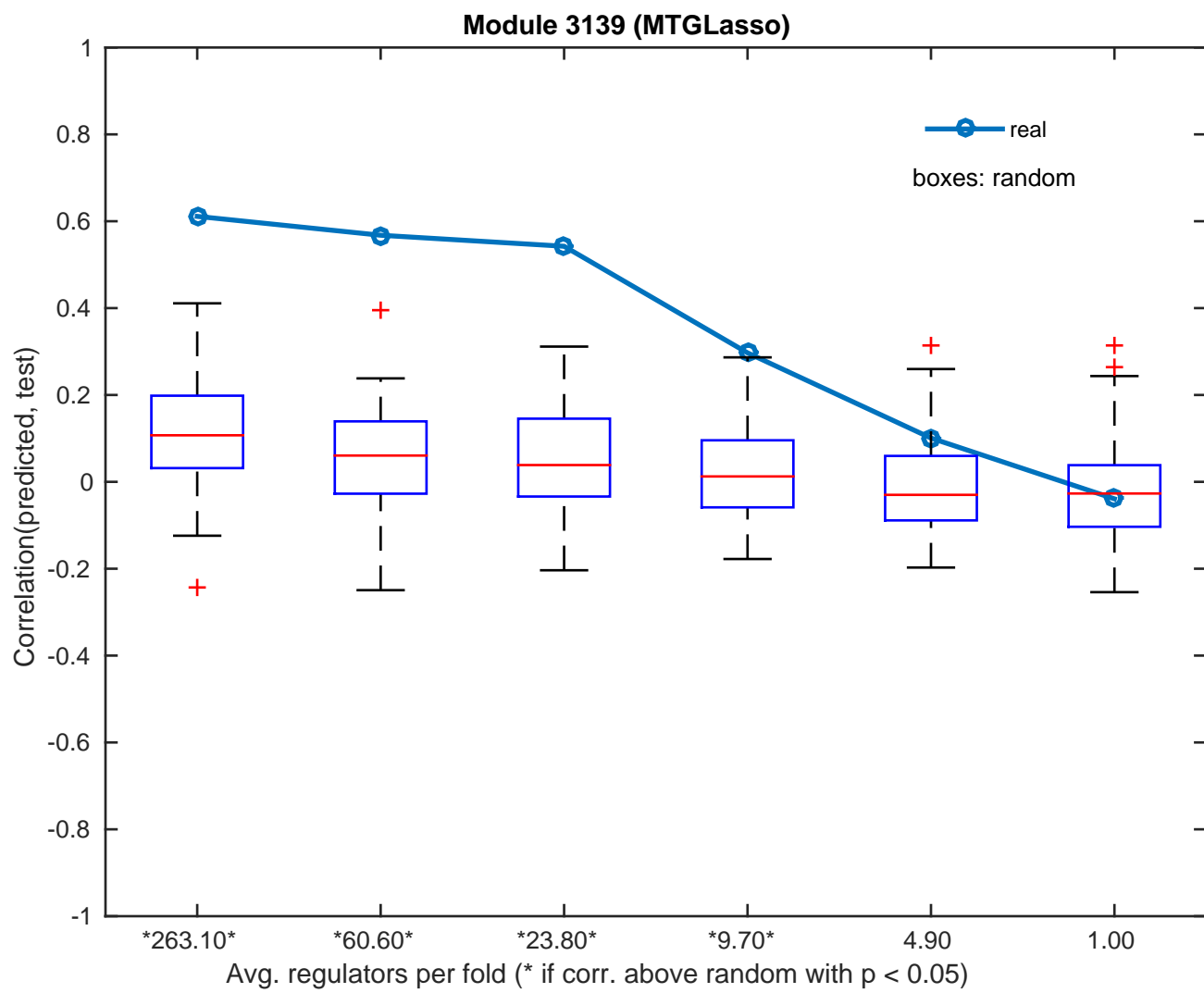

Supplement: S1 Dataset — One plot per module, both species. (GZ) [file pcbi.1005013.s024.gz › mouse_correlation_vs_lambda/mouse_module3139_mtglasso_all_lambdas.pdf]

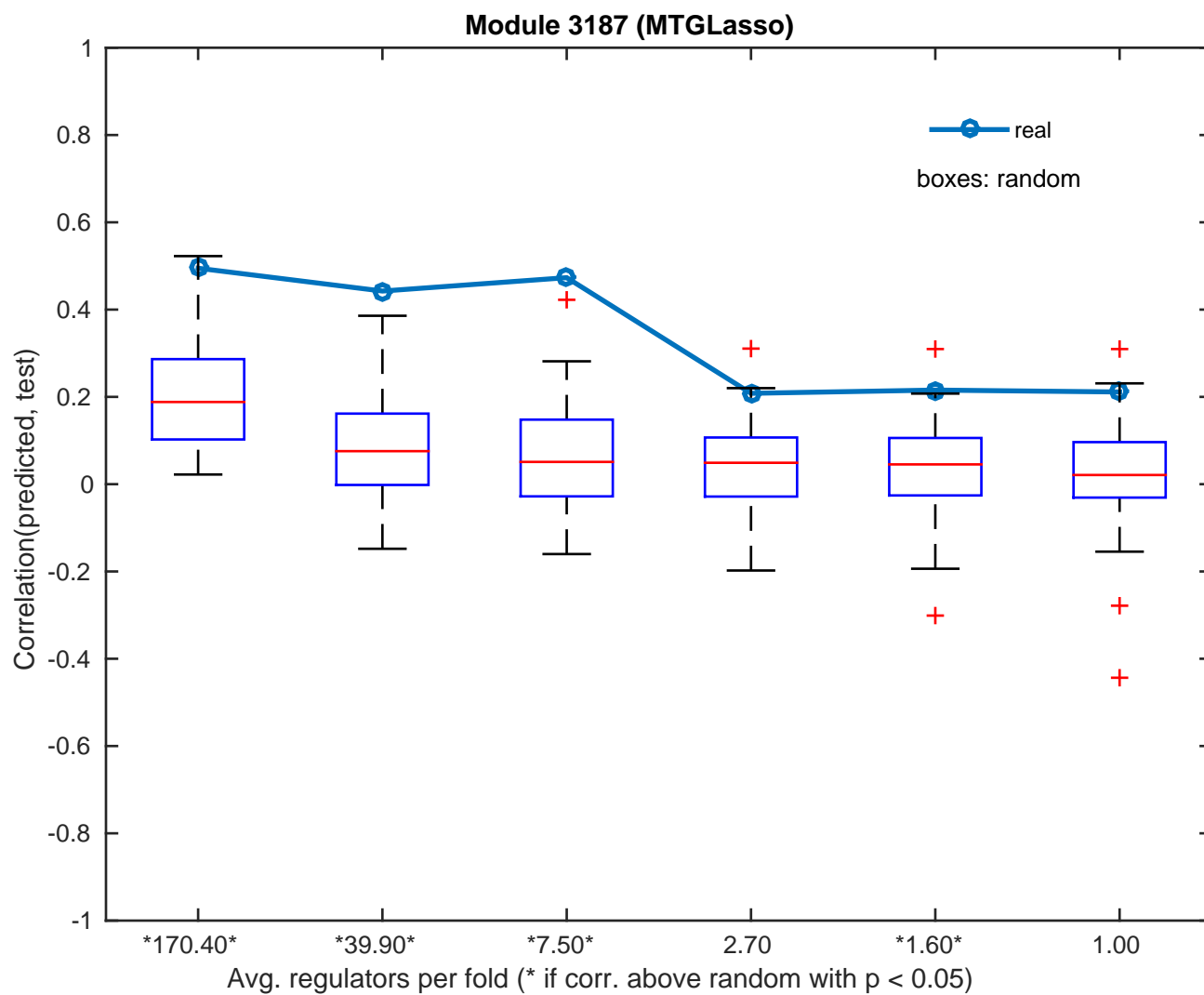

Supplement: S1 Dataset — One plot per module, both species. (GZ) [file pcbi.1005013.s024.gz › mouse_correlation_vs_lambda/mouse_module3187_mtglasso_all_lambdas.pdf]

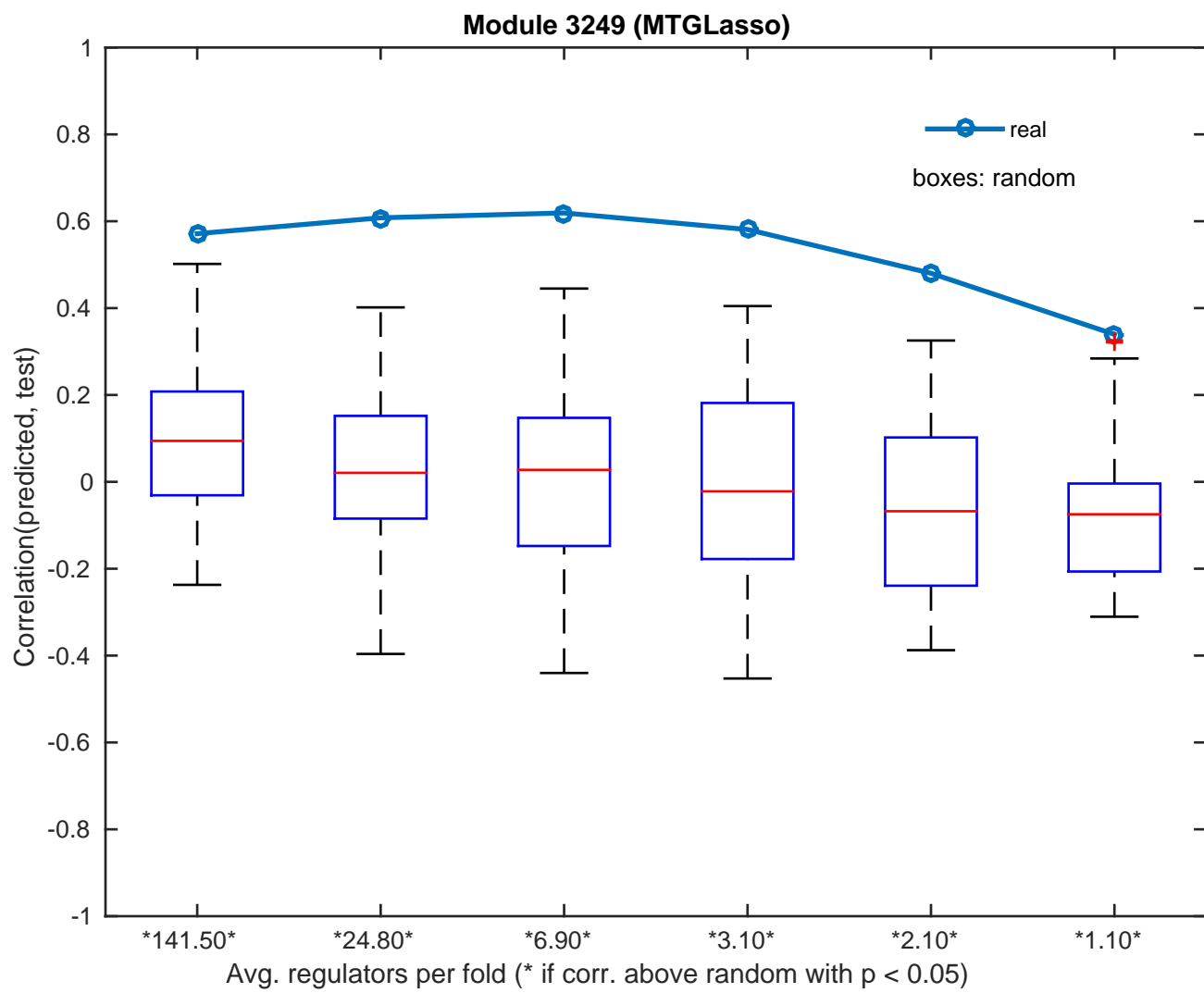

Supplement: S1 Dataset — One plot per module, both species. (GZ) [file pcbi.1005013.s024.gz › mouse_correlation_vs_lambda/mouse_module3249_mtglasso_all_lambdas.pdf]

Module 2950 (MTGLasso)

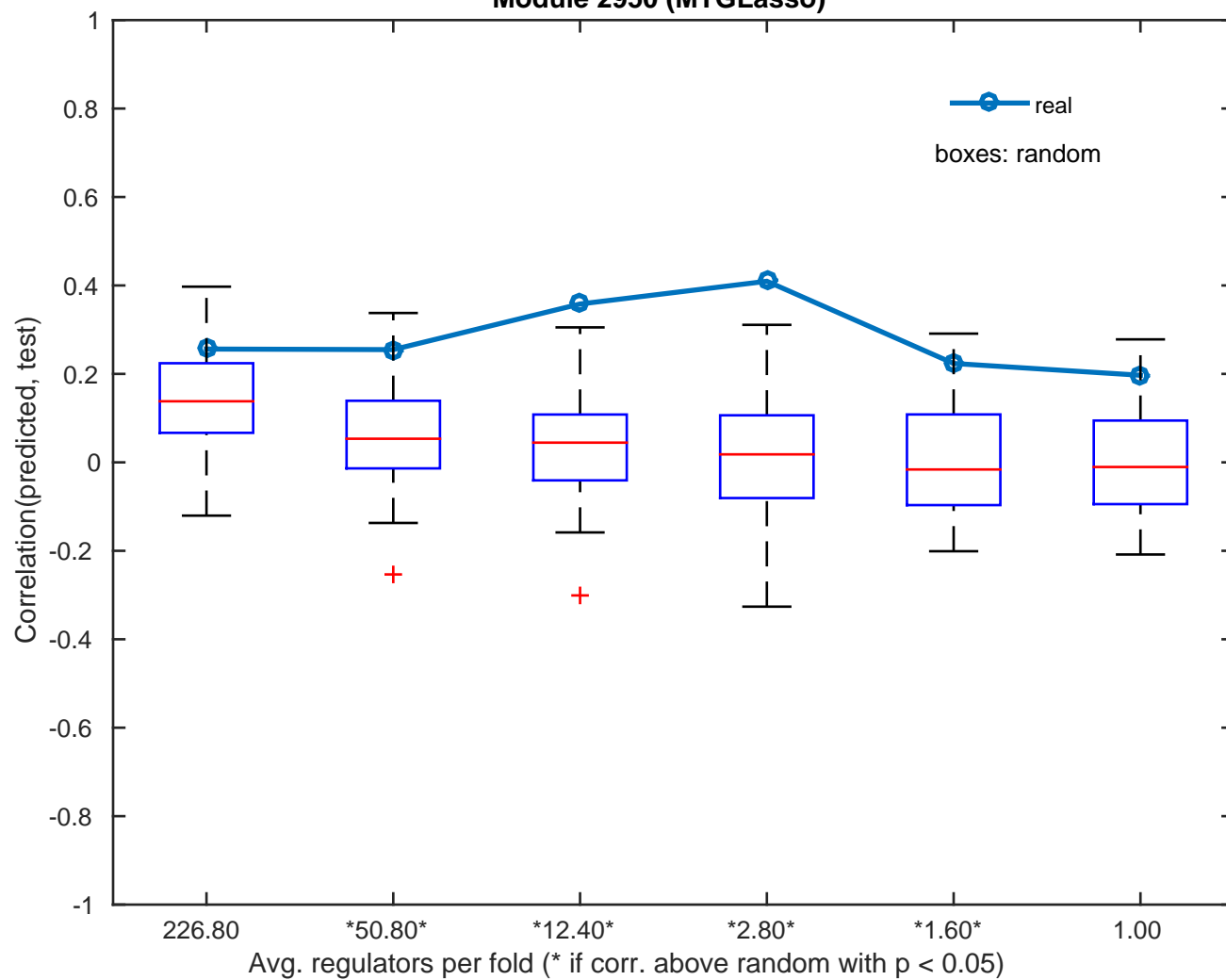

Supplement: S1 Dataset — One plot per module, both species. (GZ) [file pcbi.1005013.s024.gz › mouse_correlation_vs_lambda/mouse_module2950_mtglasso_all_lambdas.pdf]

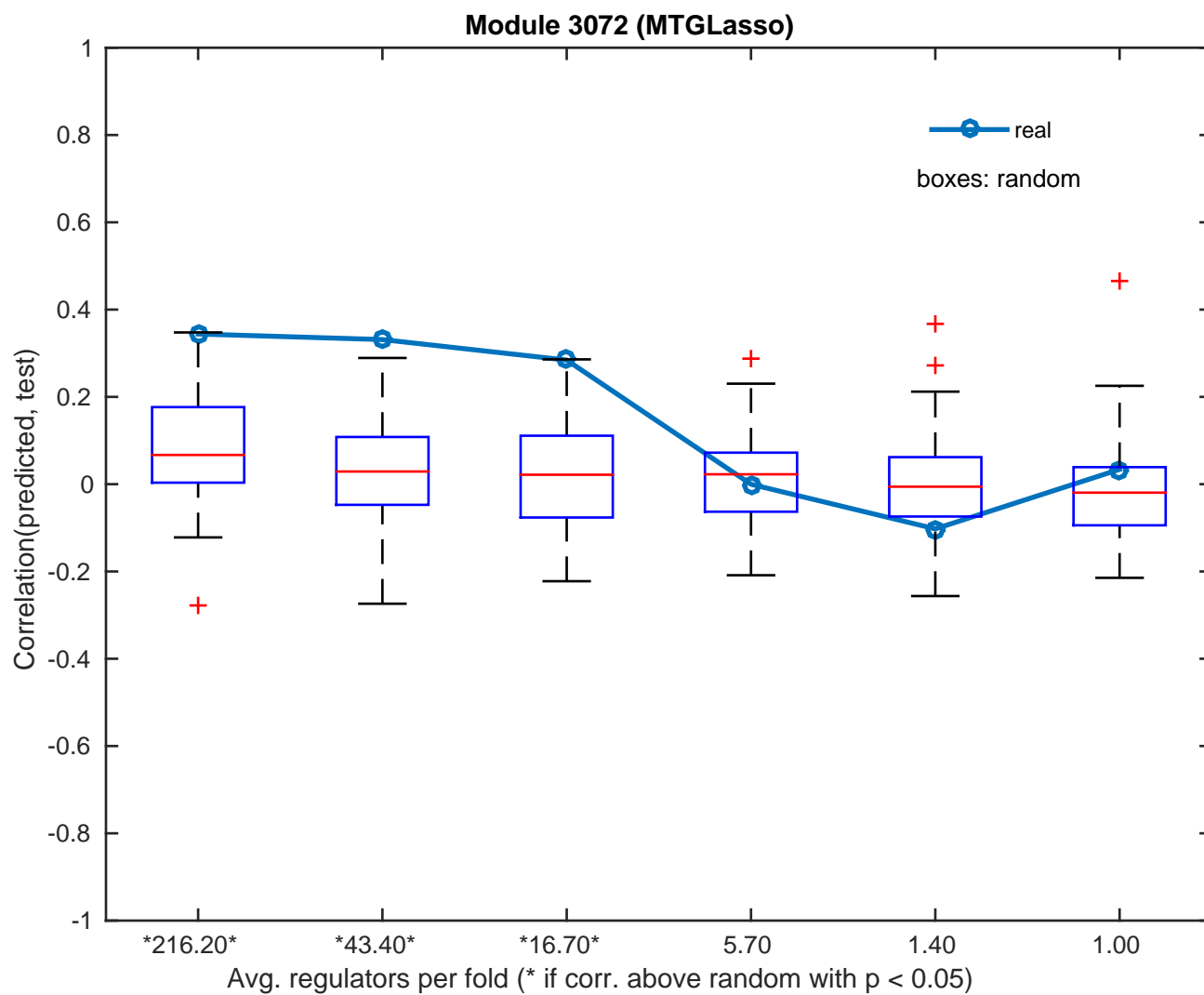

Supplement: S1 Dataset — One plot per module, both species. (GZ) [file pcbi.1005013.s024.gz › mouse_correlation_vs_lambda/mouse_module3072_mtglasso_all_lambdas.pdf]

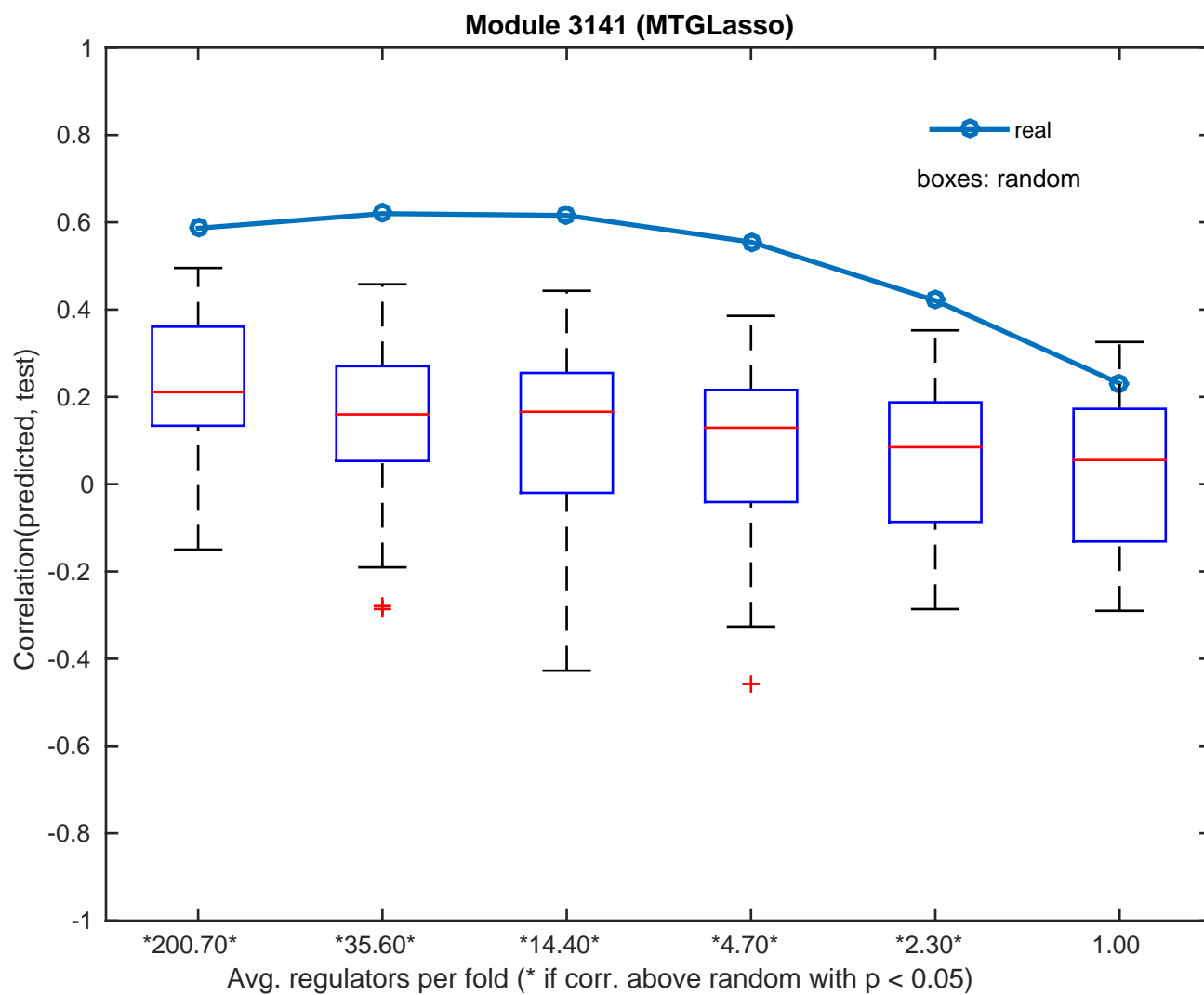

Supplement: S1 Dataset — One plot per module, both species. (GZ) [file pcbi.1005013.s024.gz › mouse_correlation_vs_lambda/mouse_module3141_mtglasso_all_lambdas.pdf]

Module 3156 (MTGLasso)

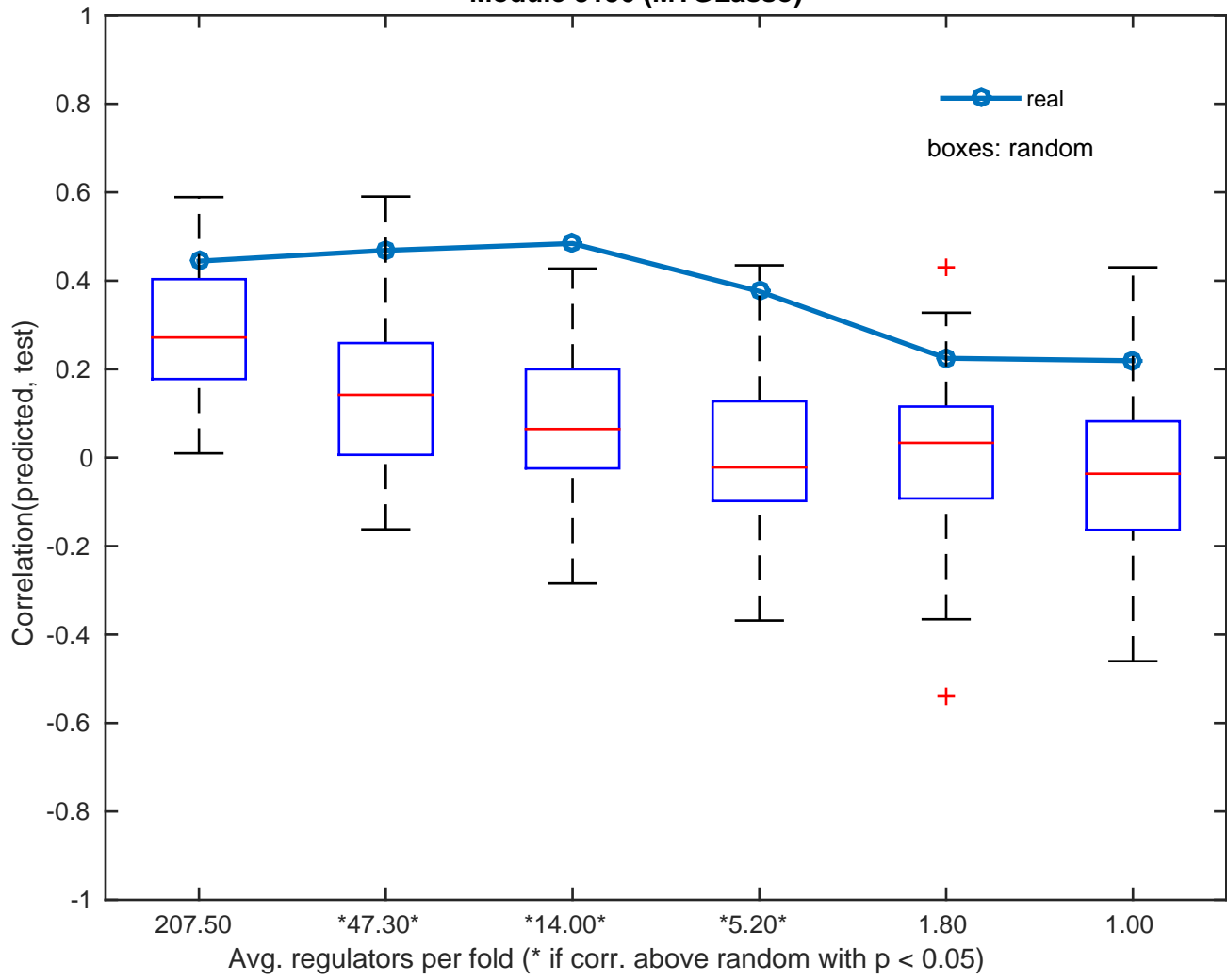

Supplement: S1 Dataset — One plot per module, both species. (GZ) [file pcbi.1005013.s024.gz › mouse_correlation_vs_lambda/mouse_module3156_mtglasso_all_lambdas.pdf]

Module 3174 (MTGLasso)

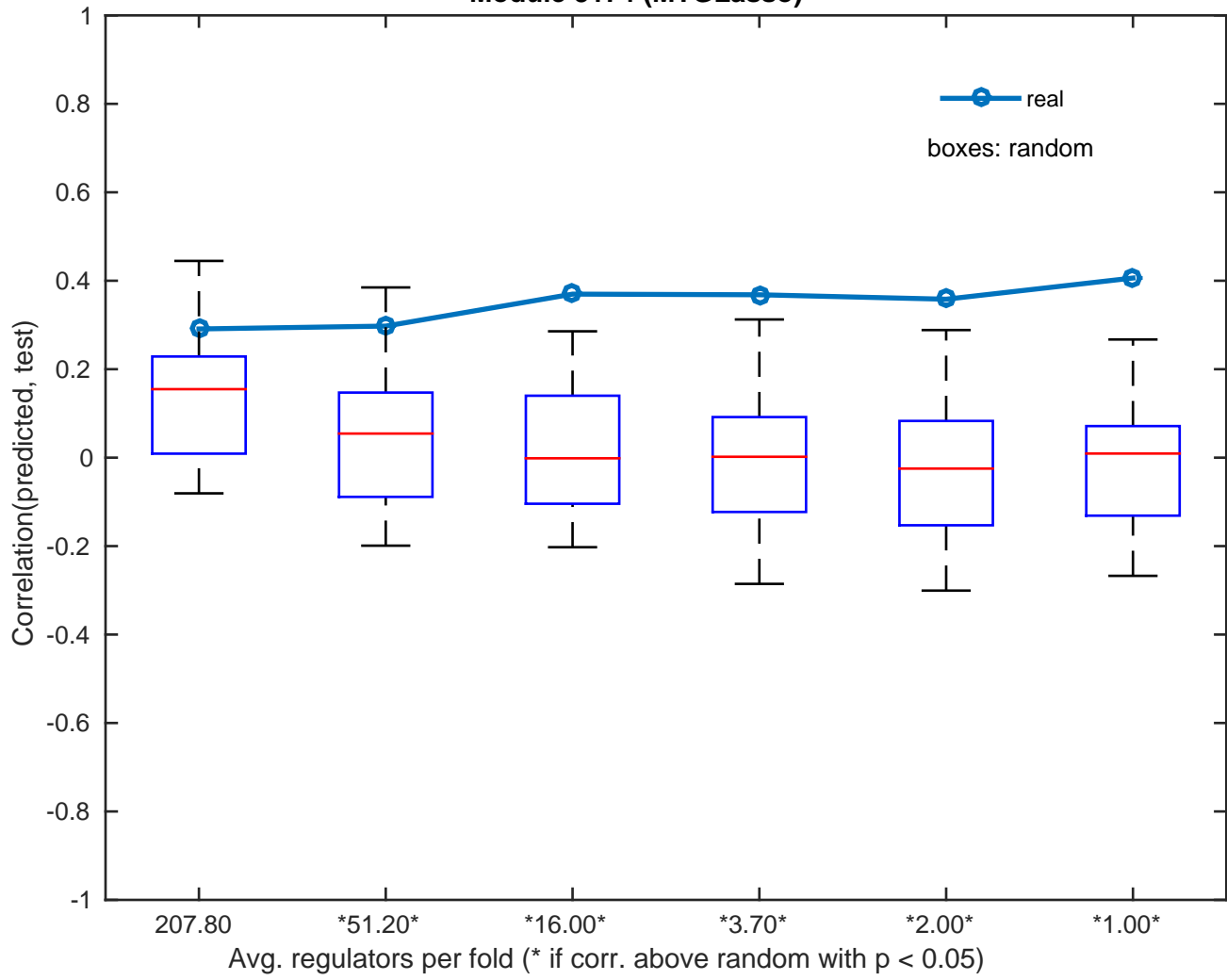

Supplement: S1 Dataset — One plot per module, both species. (GZ) [file pcbi.1005013.s024.gz › mouse_correlation_vs_lambda/mouse_module3174_mtglasso_all_lambdas.pdf]

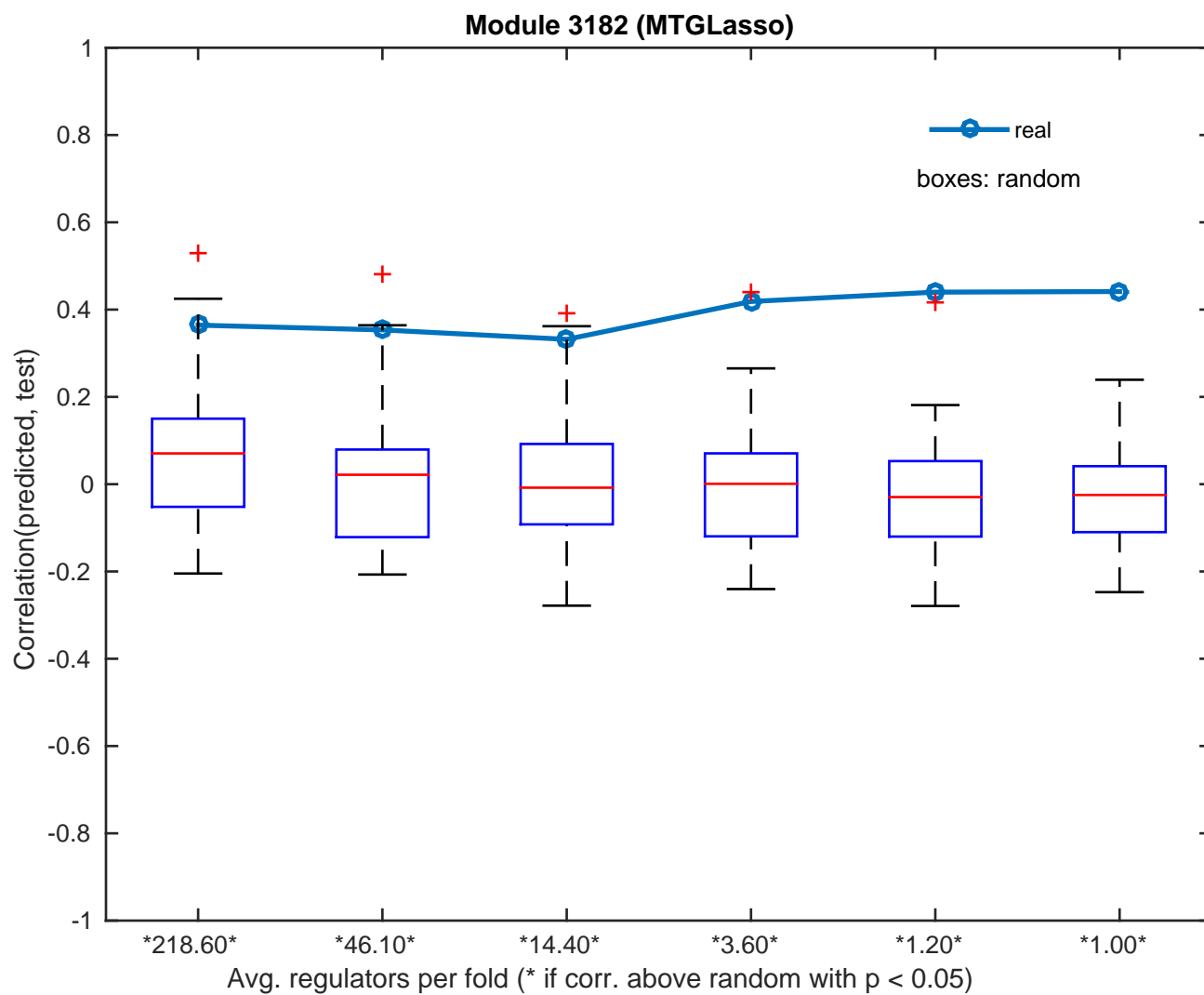

Supplement: S1 Dataset — One plot per module, both species. (GZ) [file pcbi.1005013.s024.gz › mouse_correlation_vs_lambda/mouse_module3182_mtglasso_all_lambdas.pdf]

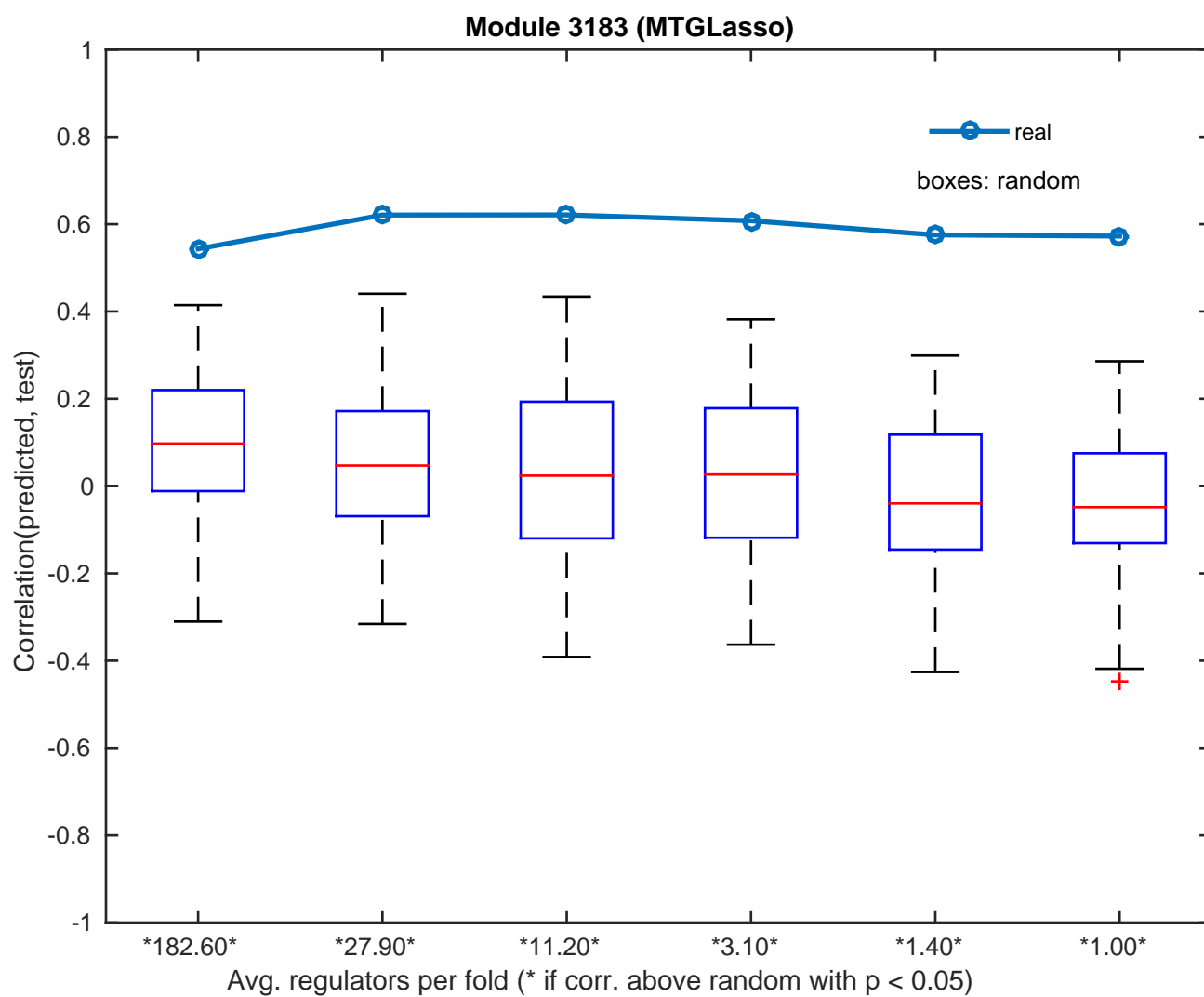

Supplement: S1 Dataset — One plot per module, both species. (GZ) [file pcbi.1005013.s024.gz › mouse_correlation_vs_lambda/mouse_module3183_mtglasso_all_lambdas.pdf]

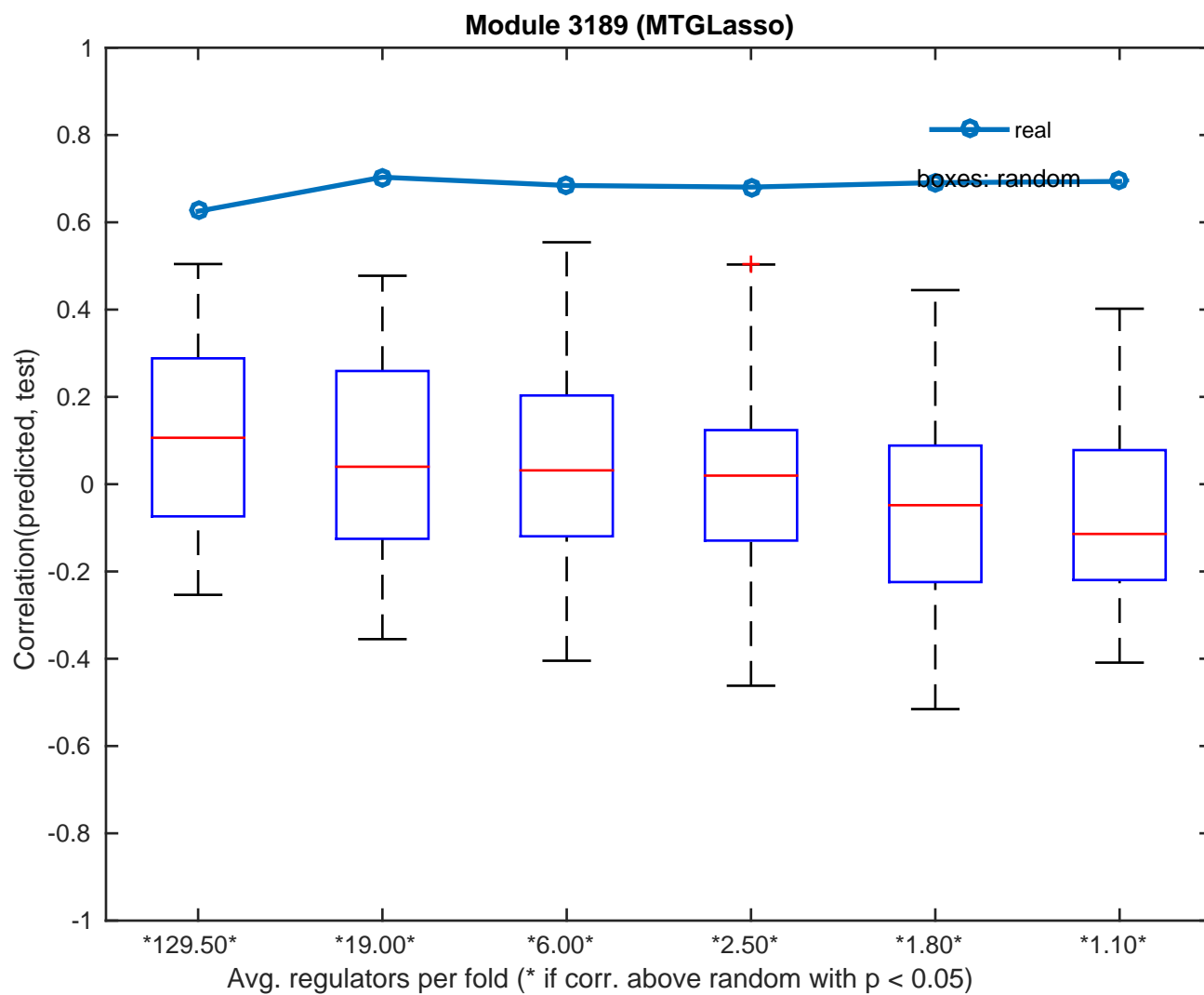

Supplement: S1 Dataset — One plot per module, both species. (GZ) [file pcbi.1005013.s024.gz › mouse_correlation_vs_lambda/mouse_module3189_mtglasso_all_lambdas.pdf]

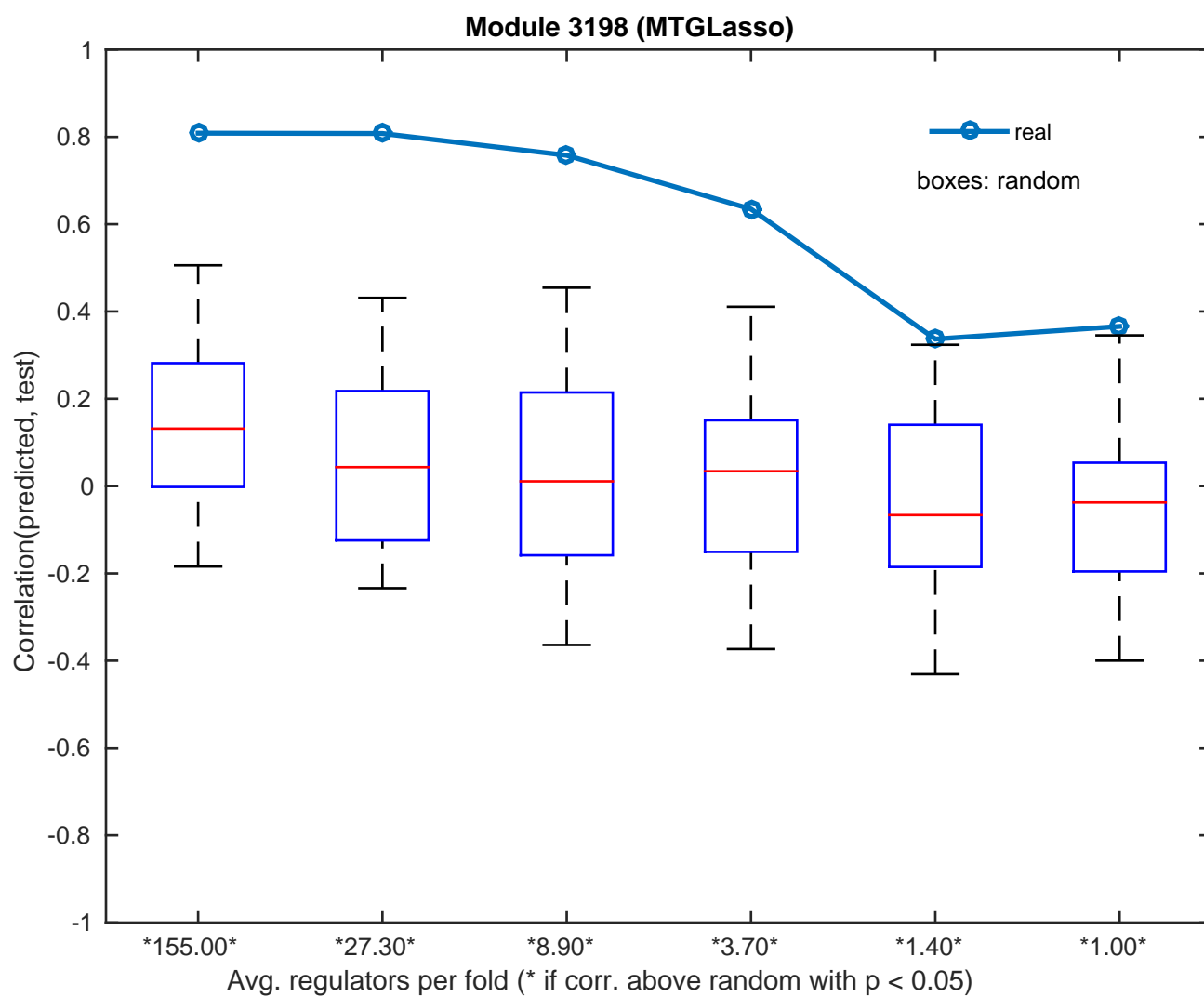

Supplement: S1 Dataset — One plot per module, both species. (GZ) [file pcbi.1005013.s024.gz › mouse_correlation_vs_lambda/mouse_module3198_mtglasso_all_lambdas.pdf]

Module 2894 (MTGLasso)

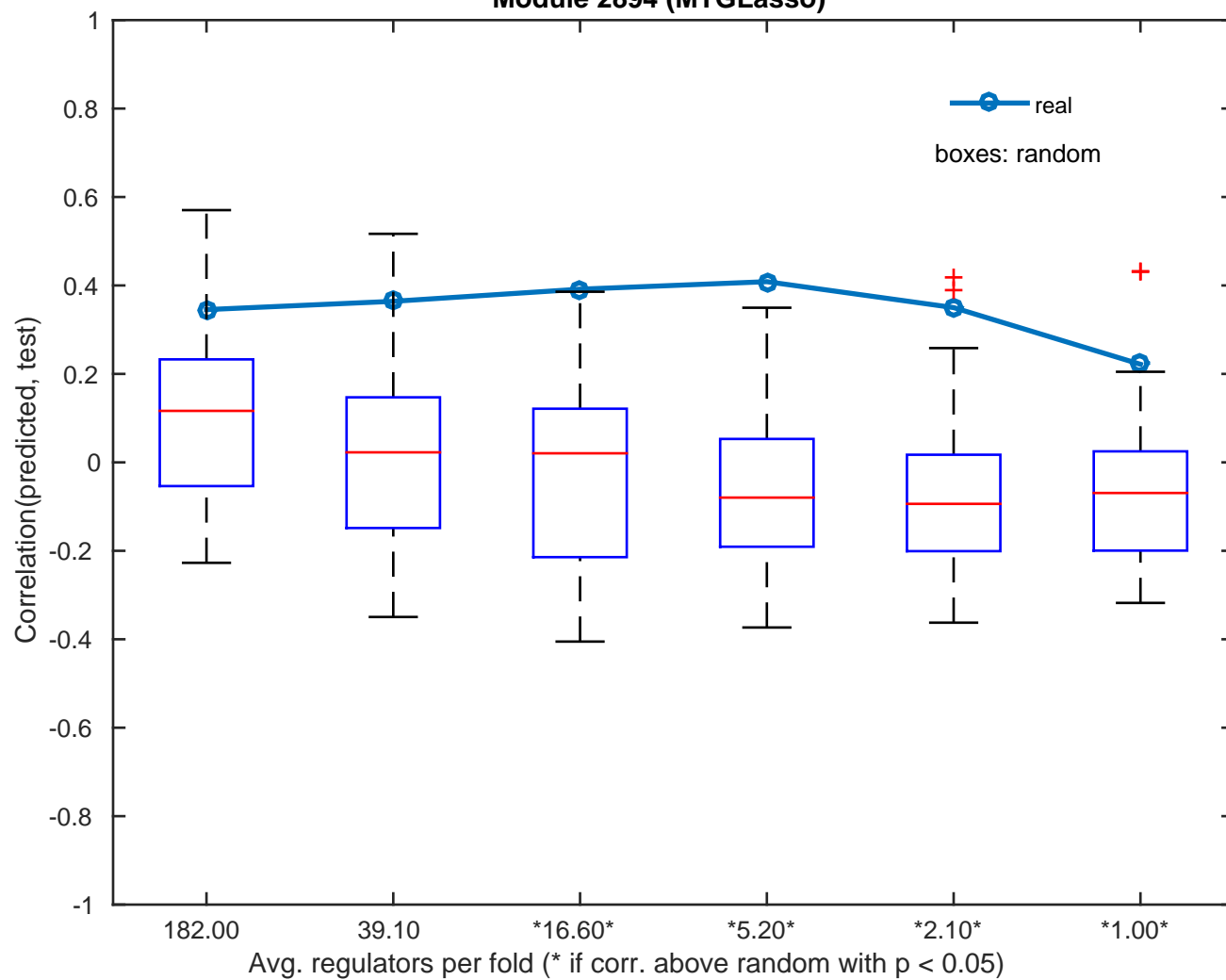

Supplement: S1 Dataset — One plot per module, both species. (GZ) [file pcbi.1005013.s024.gz › mouse_correlation_vs_lambda/mouse_module2894_mtglasso_all_lambdas.pdf]

Module 3036 (MTGLasso)

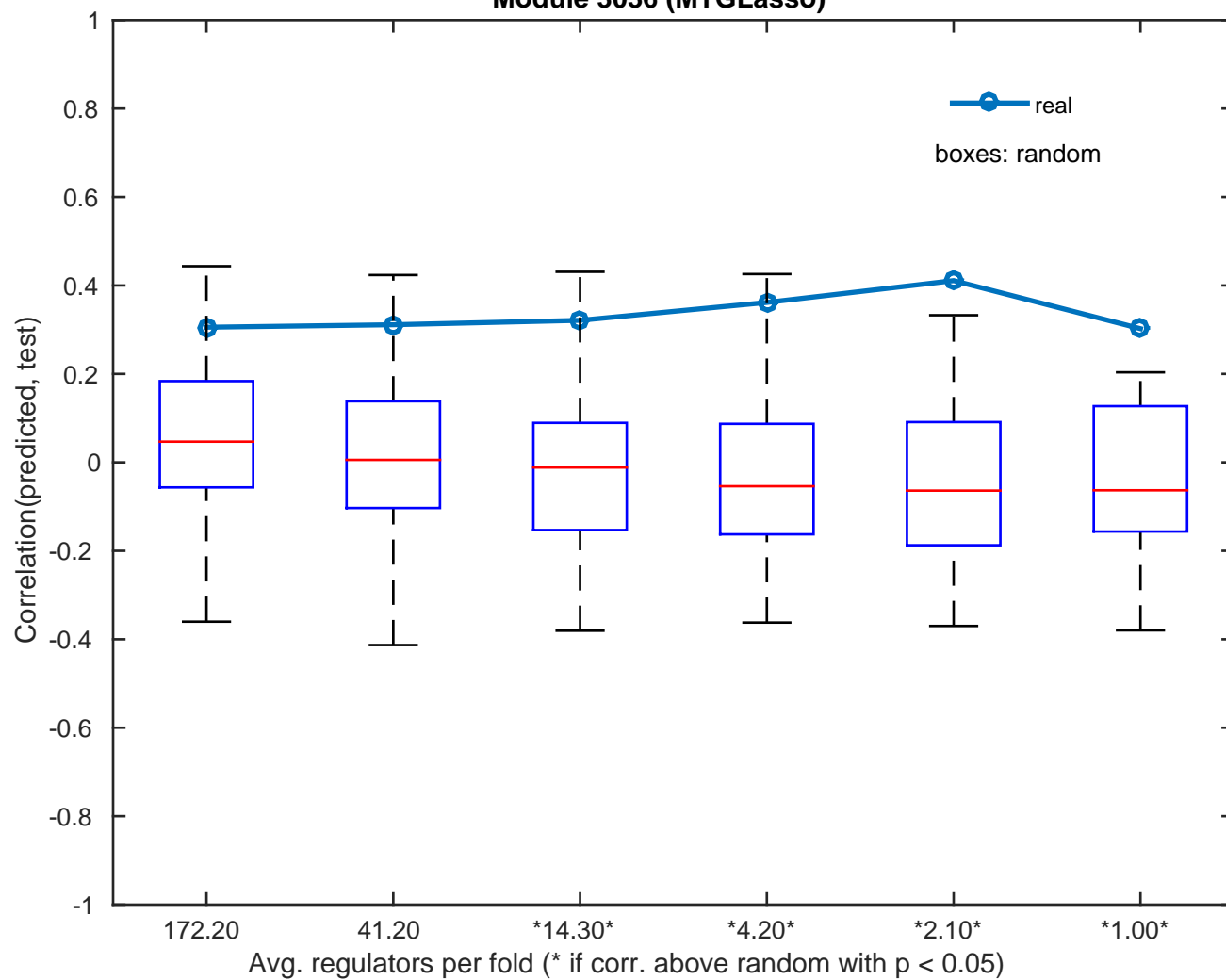

Supplement: S1 Dataset — One plot per module, both species. (GZ) [file pcbi.1005013.s024.gz › mouse_correlation_vs_lambda/mouse_module3036_mtglasso_all_lambdas.pdf]

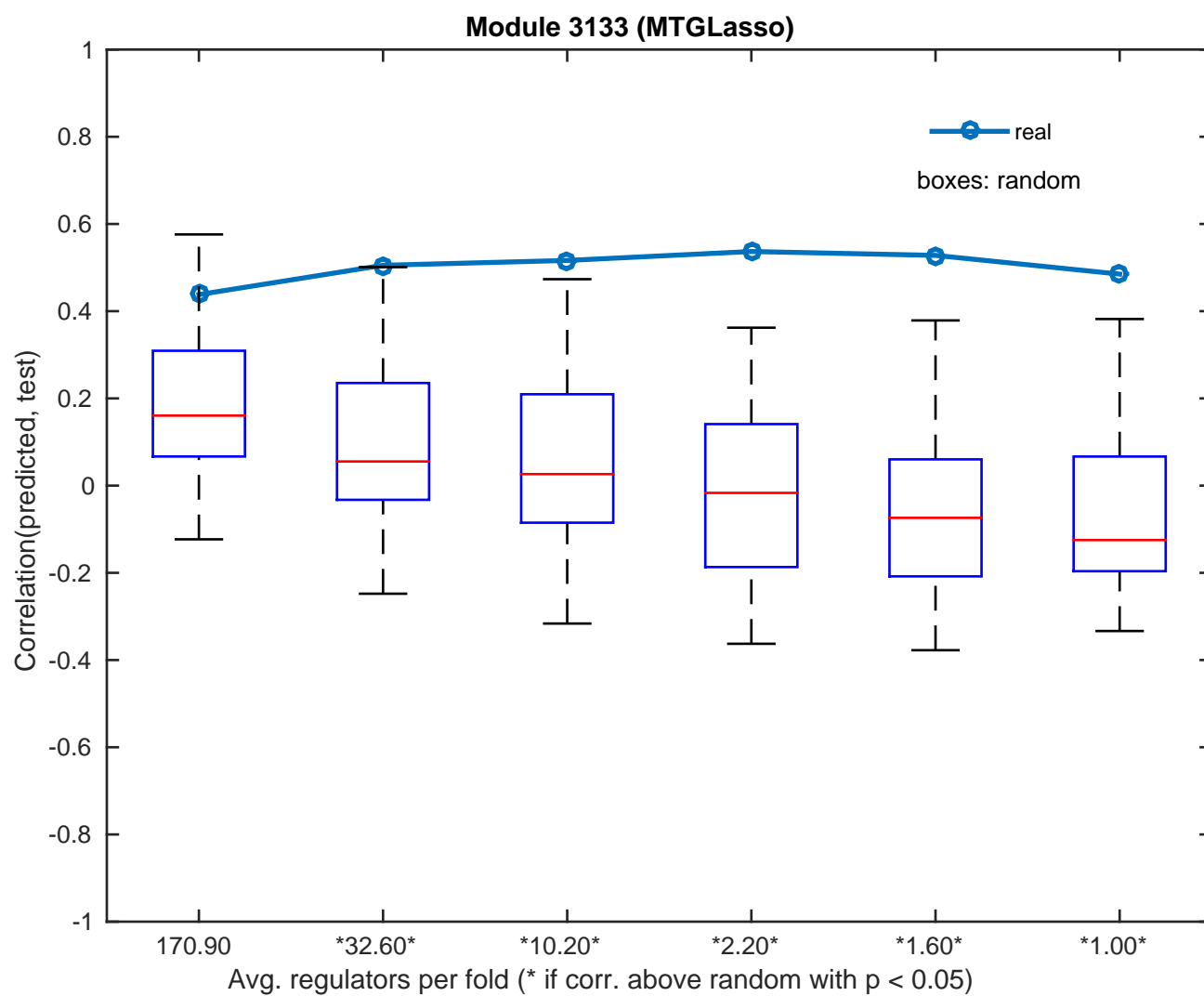

Supplement: S1 Dataset — One plot per module, both species. (GZ) [file pcbi.1005013.s024.gz › mouse_correlation_vs_lambda/mouse_module3133_mtglasso_all_lambdas.pdf]

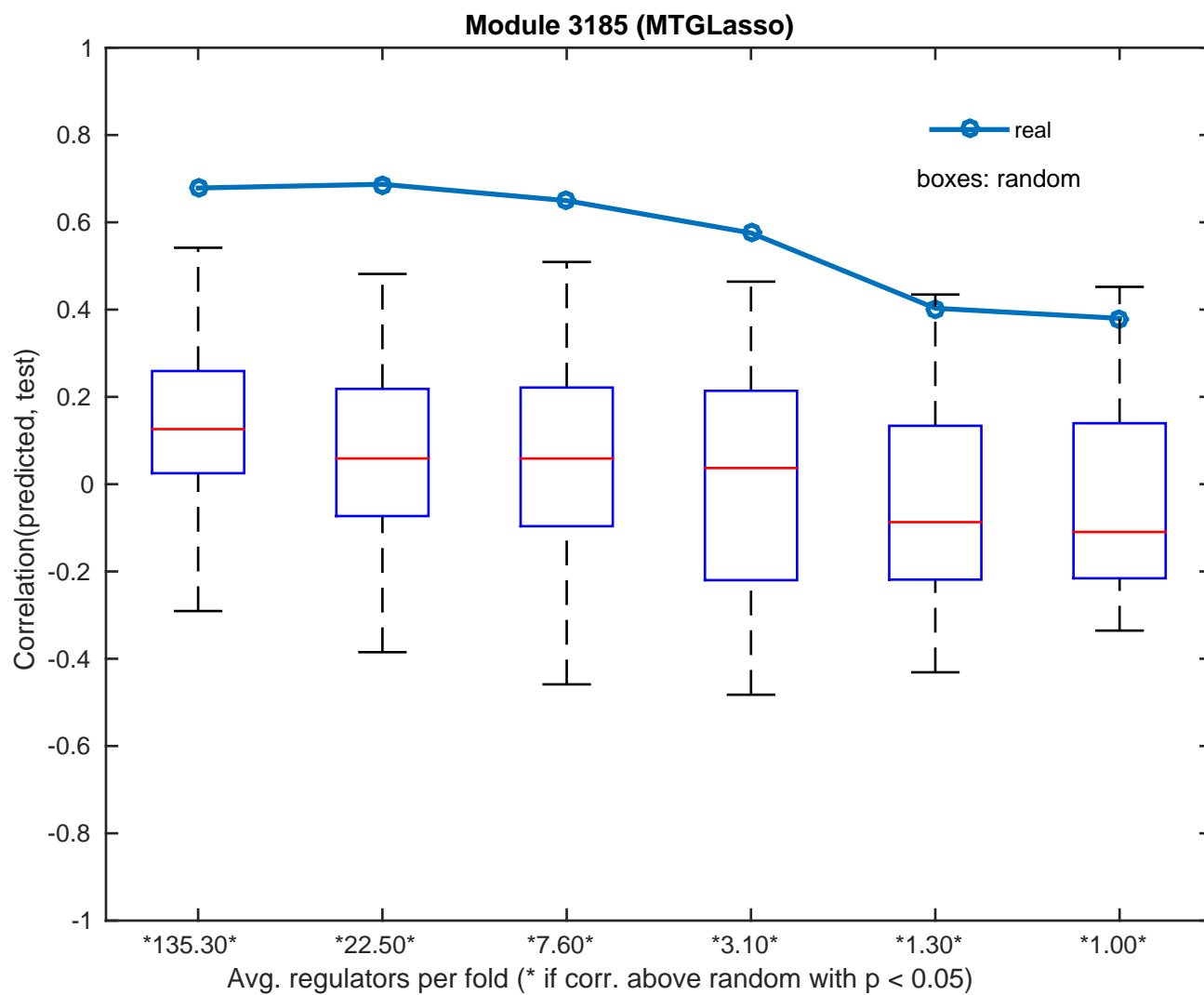

Supplement: S1 Dataset — One plot per module, both species. (GZ) [file pcbi.1005013.s024.gz › mouse_correlation_vs_lambda/mouse_module3185_mtglasso_all_lambdas.pdf]

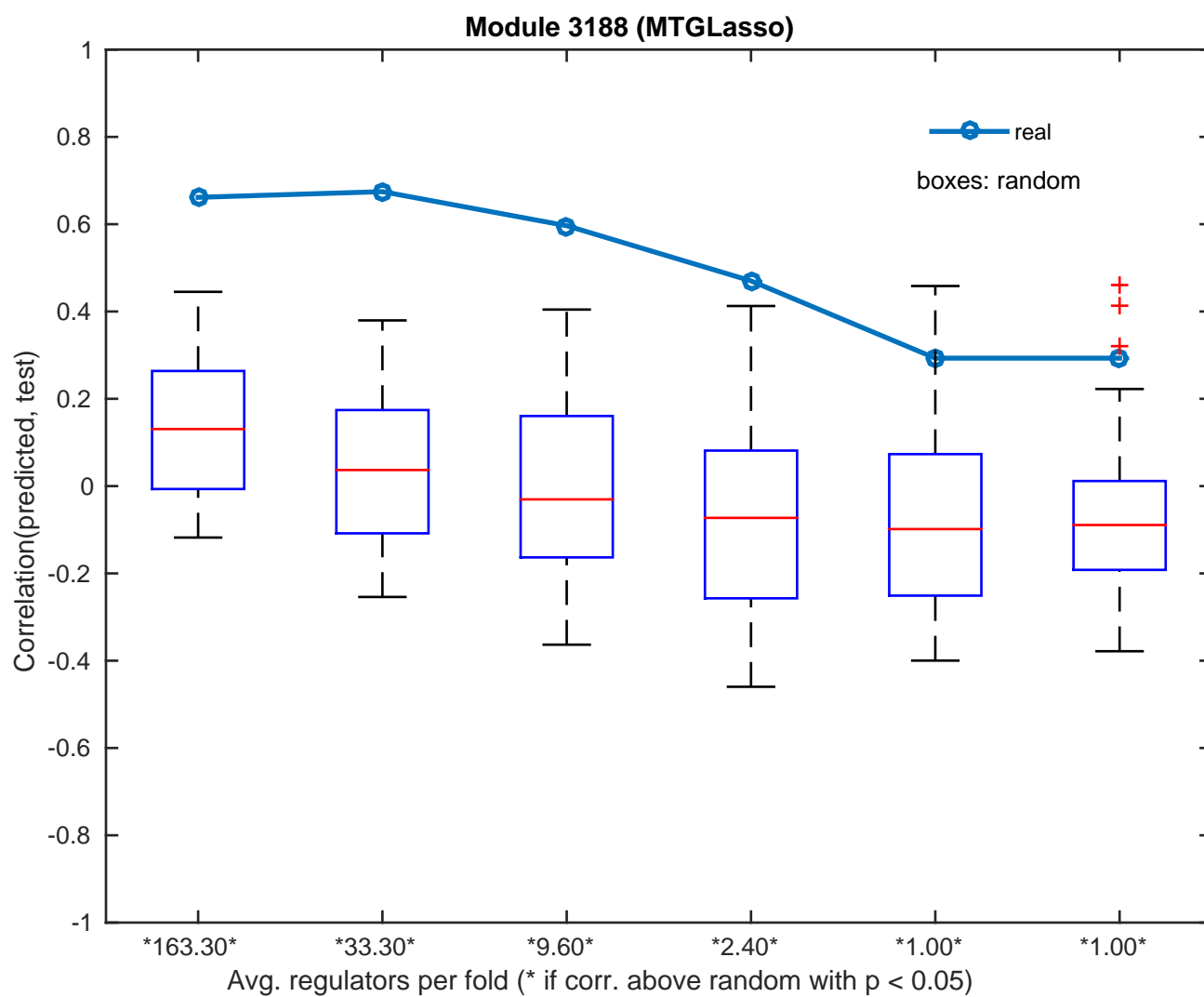

Supplement: S1 Dataset — One plot per module, both species. (GZ) [file pcbi.1005013.s024.gz › mouse_correlation_vs_lambda/mouse_module3188_mtglasso_all_lambdas.pdf]

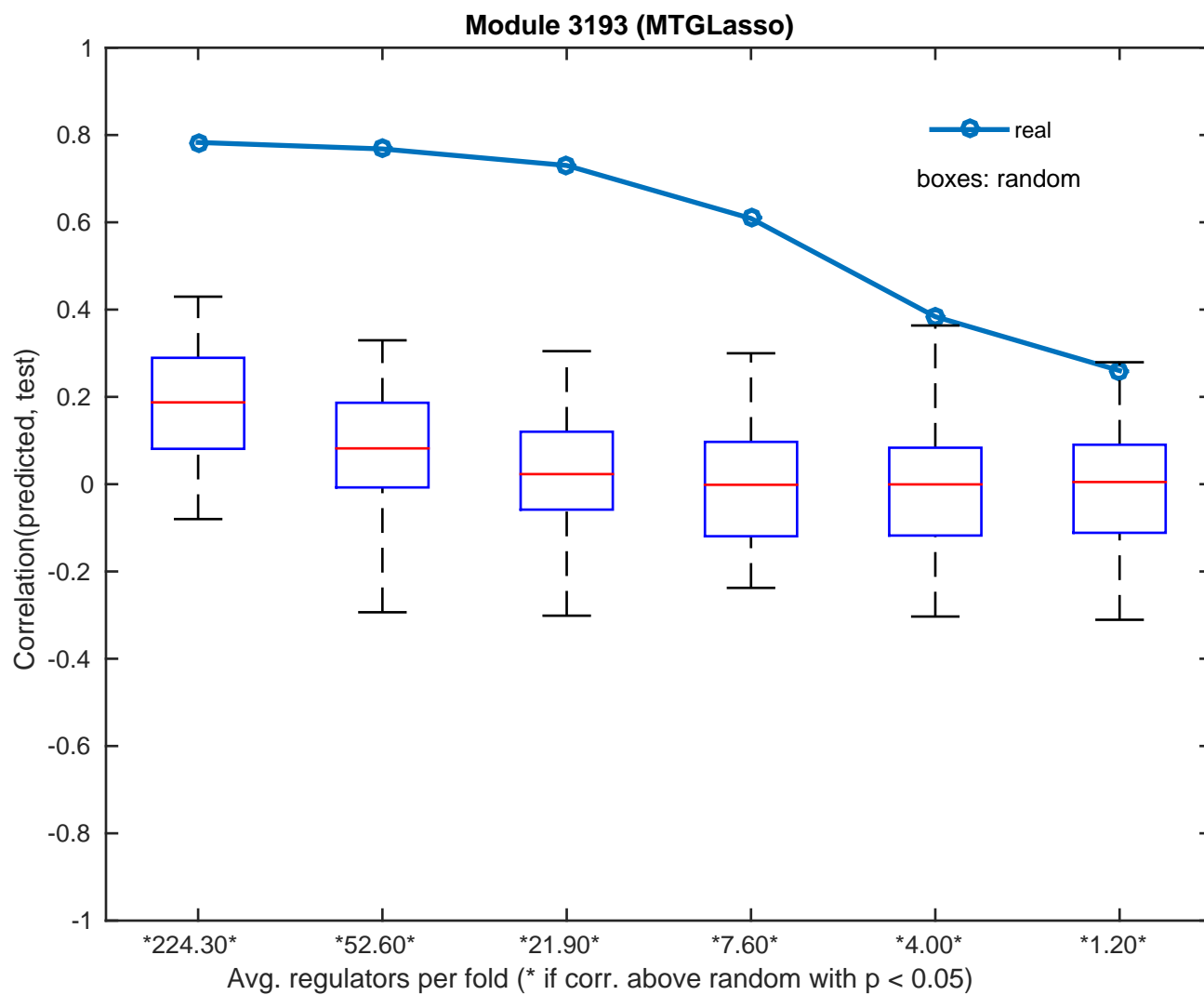

Supplement: S1 Dataset — One plot per module, both species. (GZ) [file pcbi.1005013.s024.gz › mouse_correlation_vs_lambda/mouse_module3193_mtglasso_all_lambdas.pdf]

Module 3197 (MTGLasso)

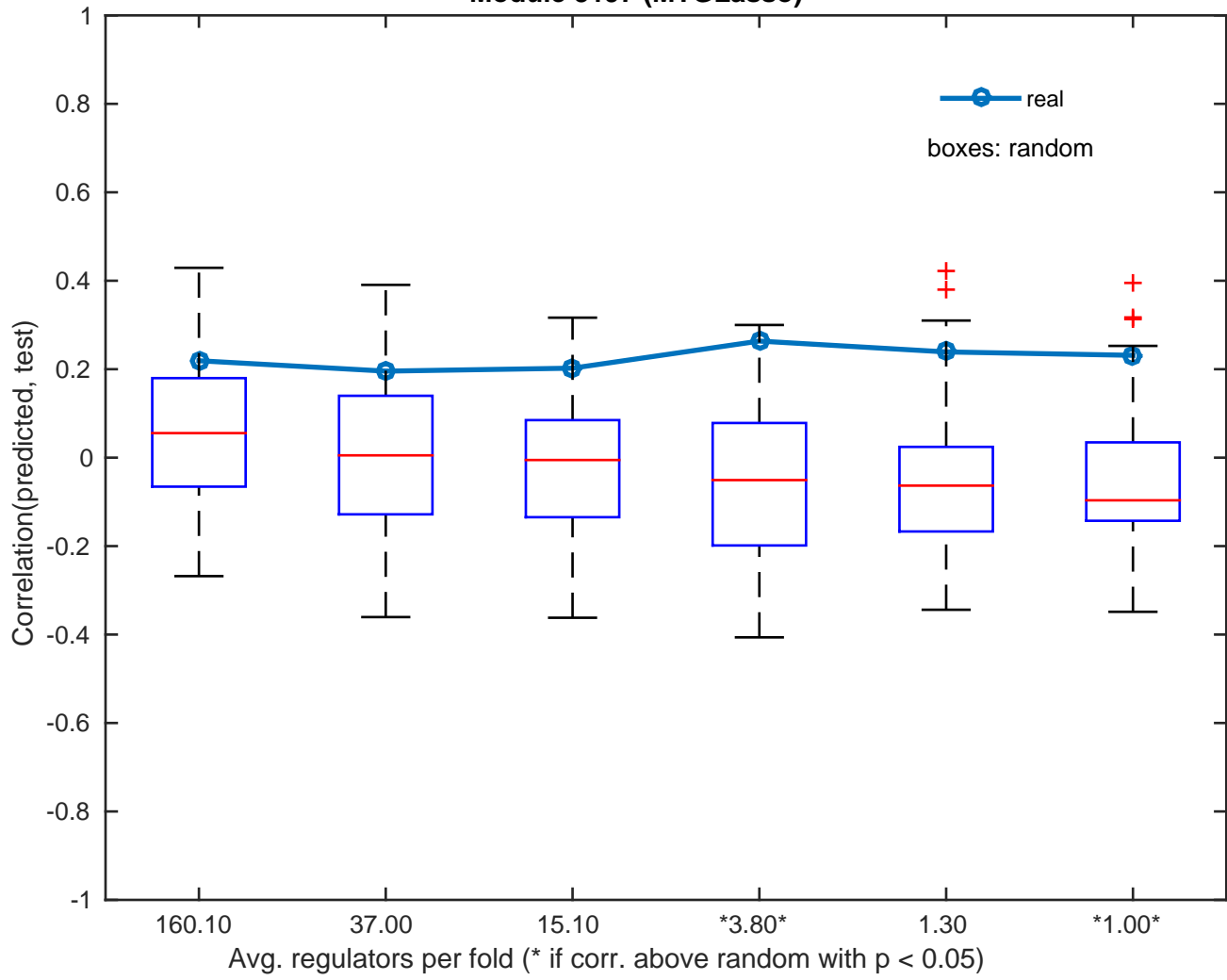

Supplement: S1 Dataset — One plot per module, both species. (GZ) [file pcbi.1005013.s024.gz › mouse_correlation_vs_lambda/mouse_module3197_mtglasso_all_lambdas.pdf]

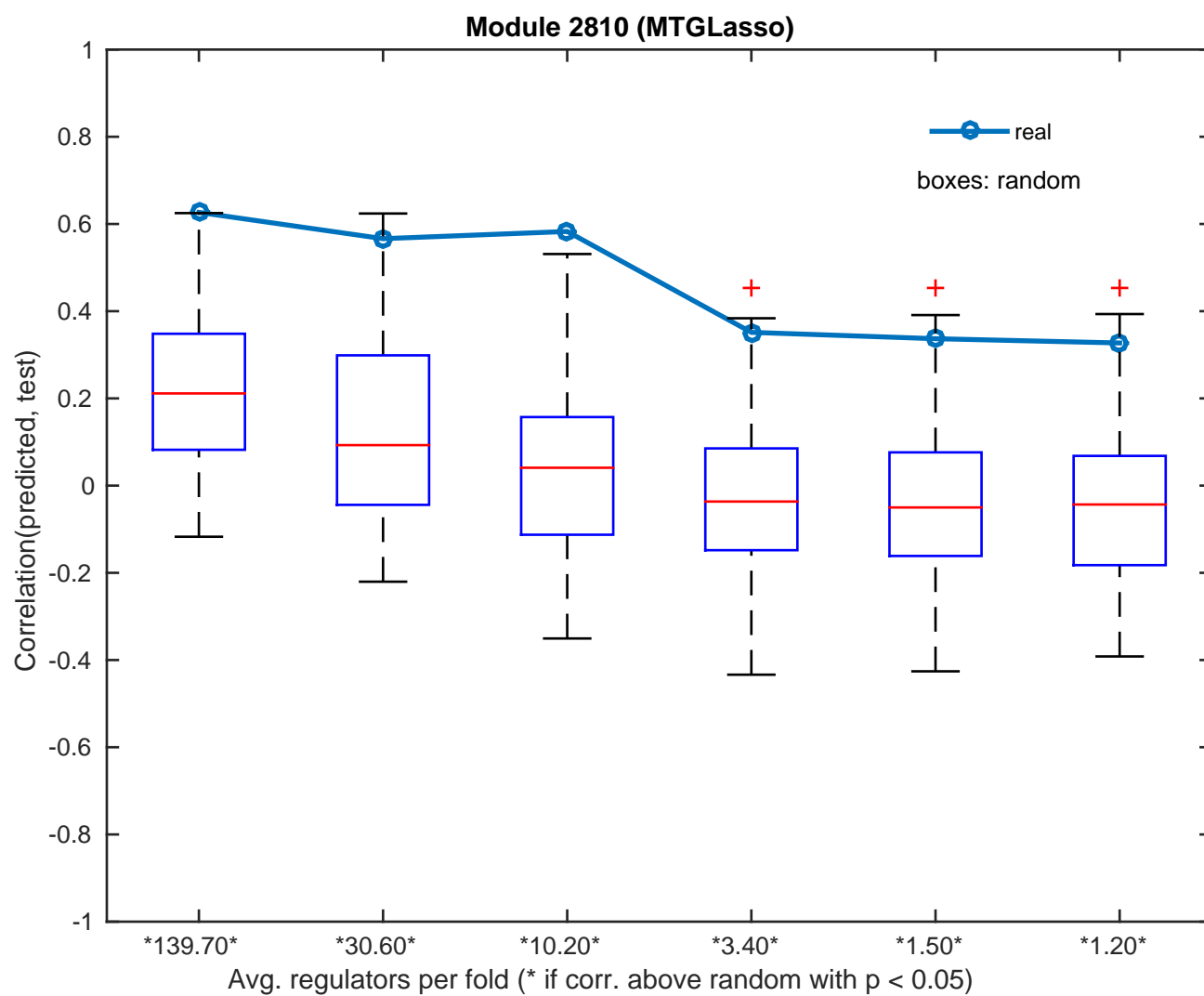

Supplement: S1 Dataset — One plot per module, both species. (GZ) [file pcbi.1005013.s024.gz › mouse_correlation_vs_lambda/mouse_module2810_mtglasso_all_lambdas.pdf]

Module 2975 (MTGLasso)

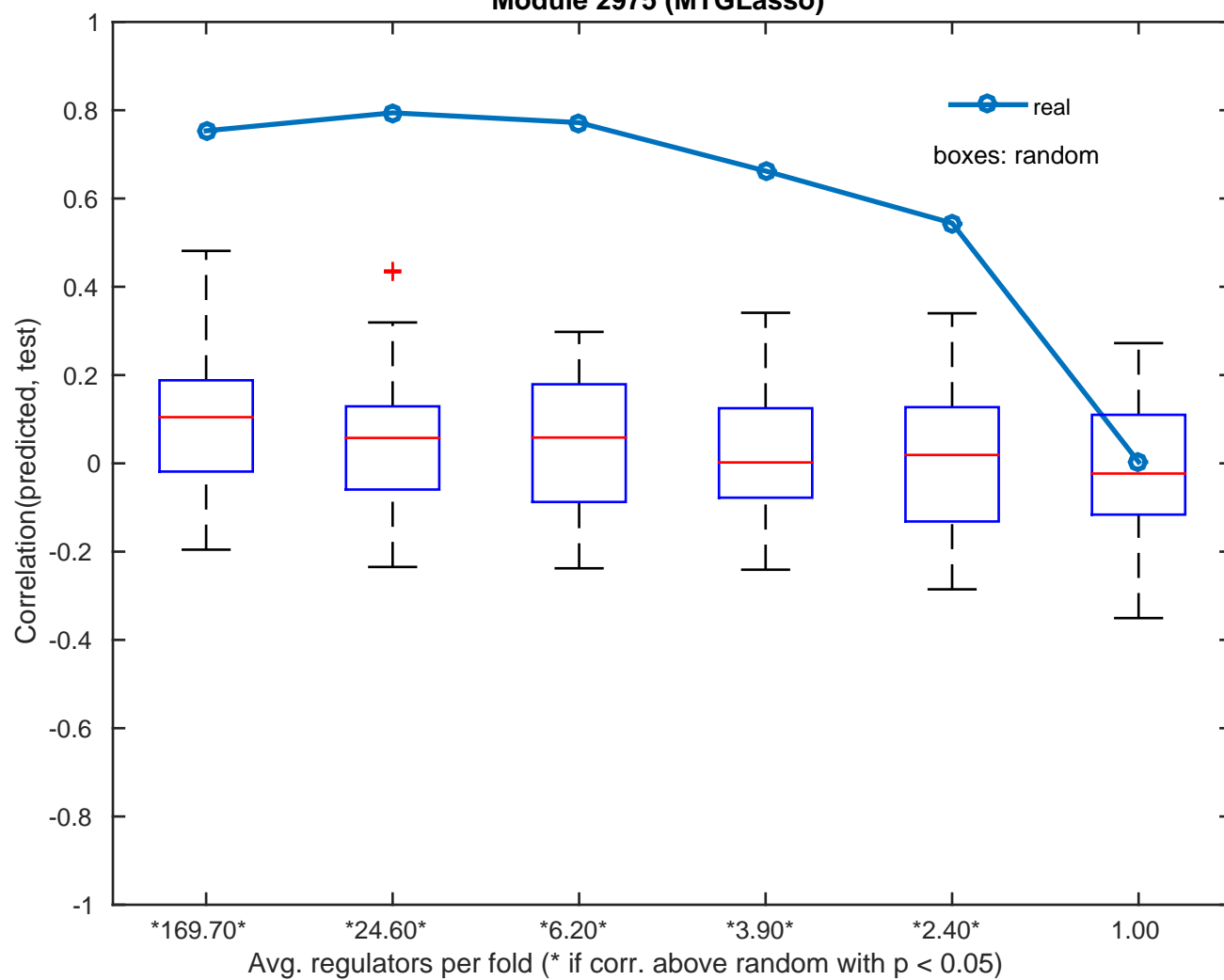

Supplement: S1 Dataset — One plot per module, both species. (GZ) [file pcbi.1005013.s024.gz › mouse_correlation_vs_lambda/mouse_module2975_mtglasso_all_lambdas.pdf]

Module 3057 (MTGLasso)

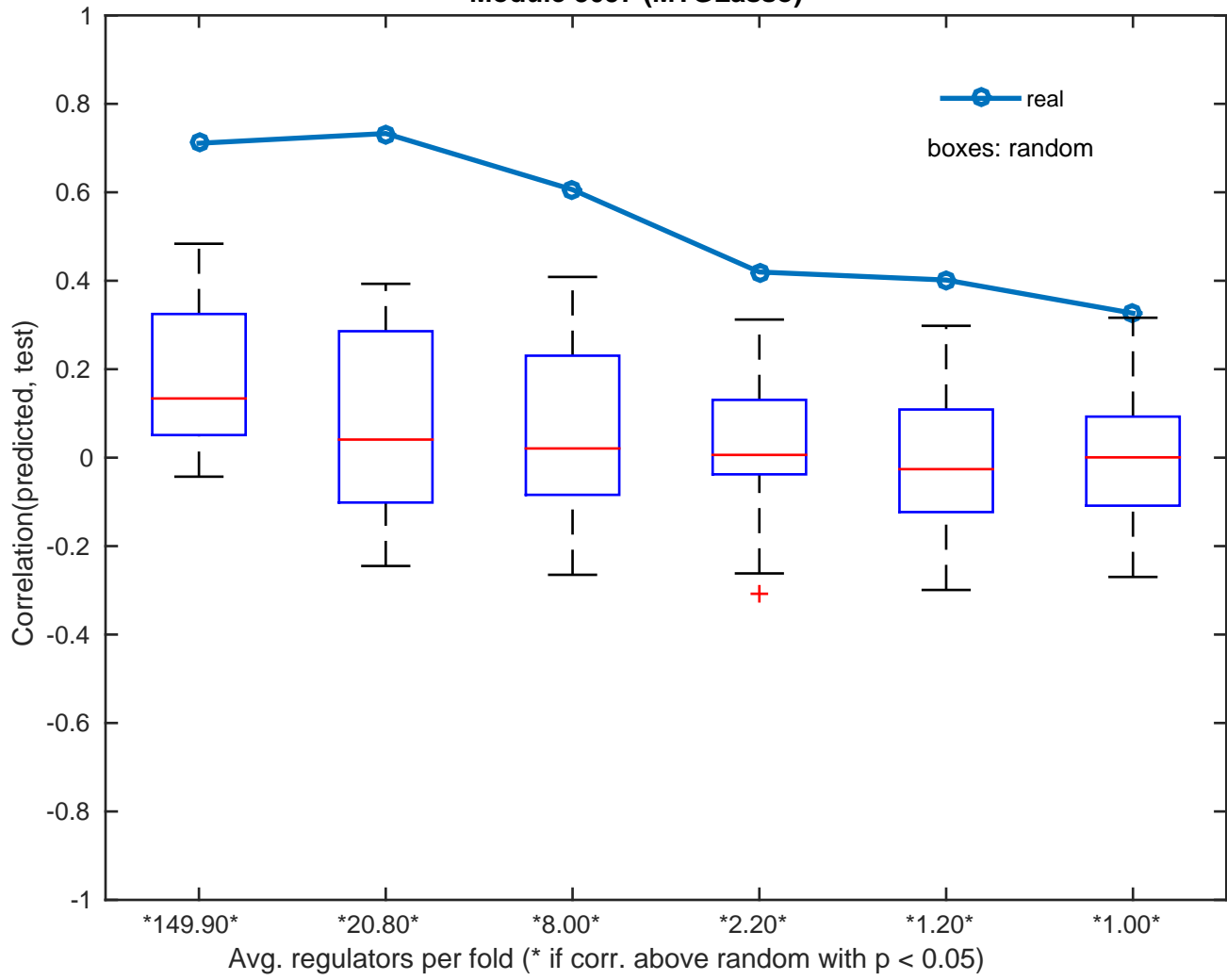

Supplement: S1 Dataset — One plot per module, both species. (GZ) [file pcbi.1005013.s024.gz › mouse_correlation_vs_lambda/mouse_module3057_mtglasso_all_lambdas.pdf]

Module 3070 (MTGLasso)

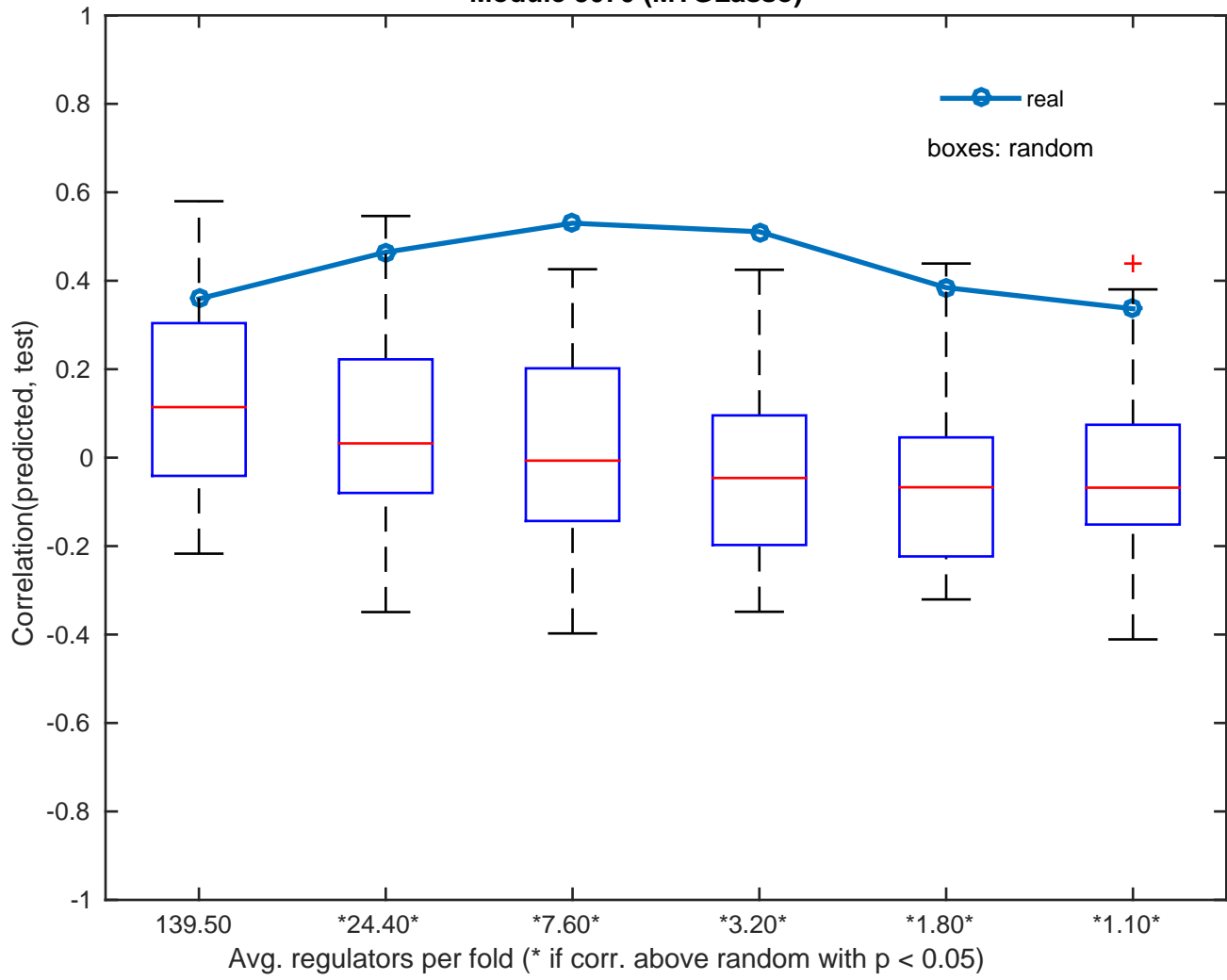

Supplement: S1 Dataset — One plot per module, both species. (GZ) [file pcbi.1005013.s024.gz › mouse_correlation_vs_lambda/mouse_module3070_mtglasso_all_lambdas.pdf]

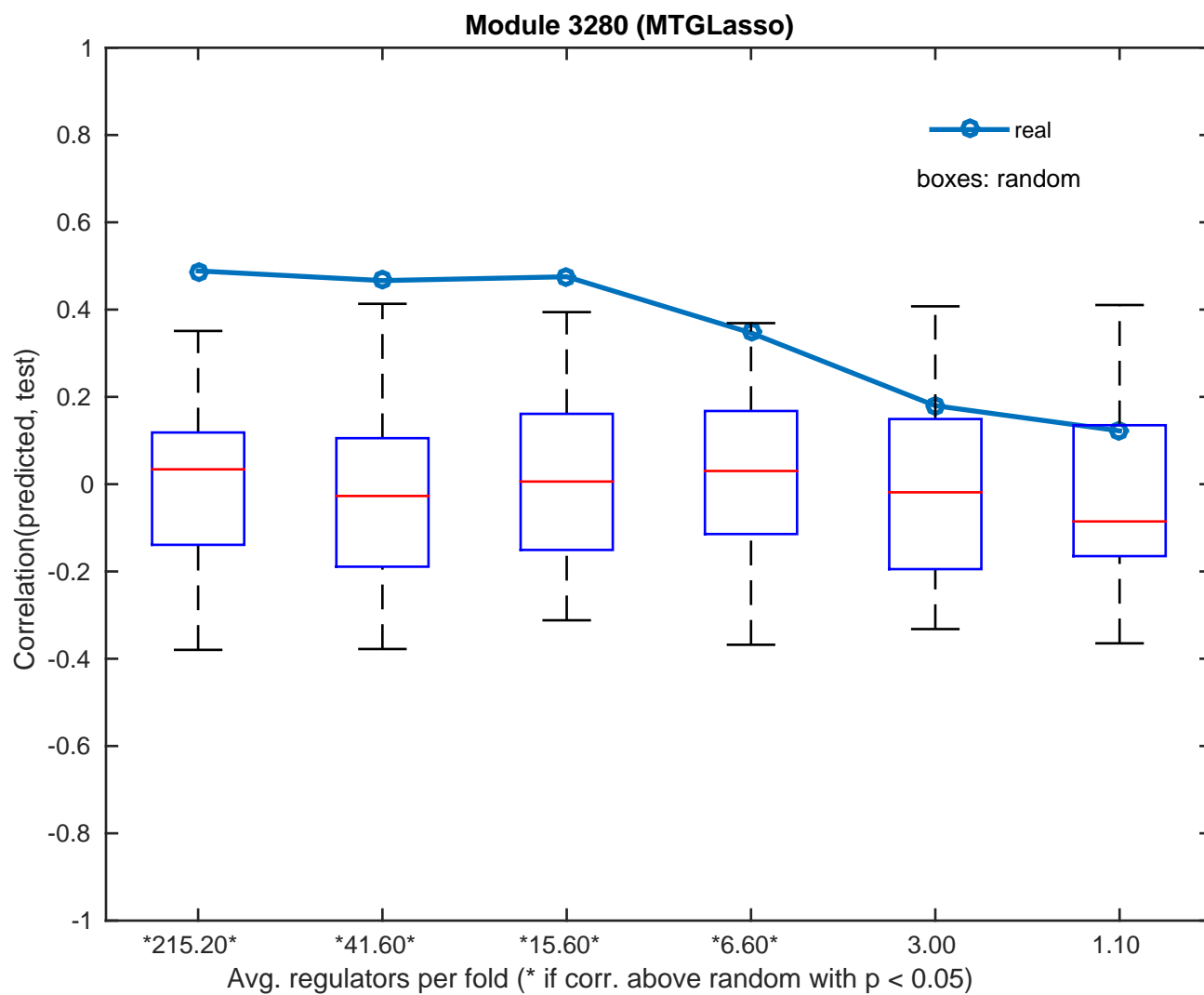

Supplement: S1 Dataset — One plot per module, both species. (GZ) [file pcbi.1005013.s024.gz › mouse_correlation_vs_lambda/mouse_module3280_mtglasso_all_lambdas.pdf]

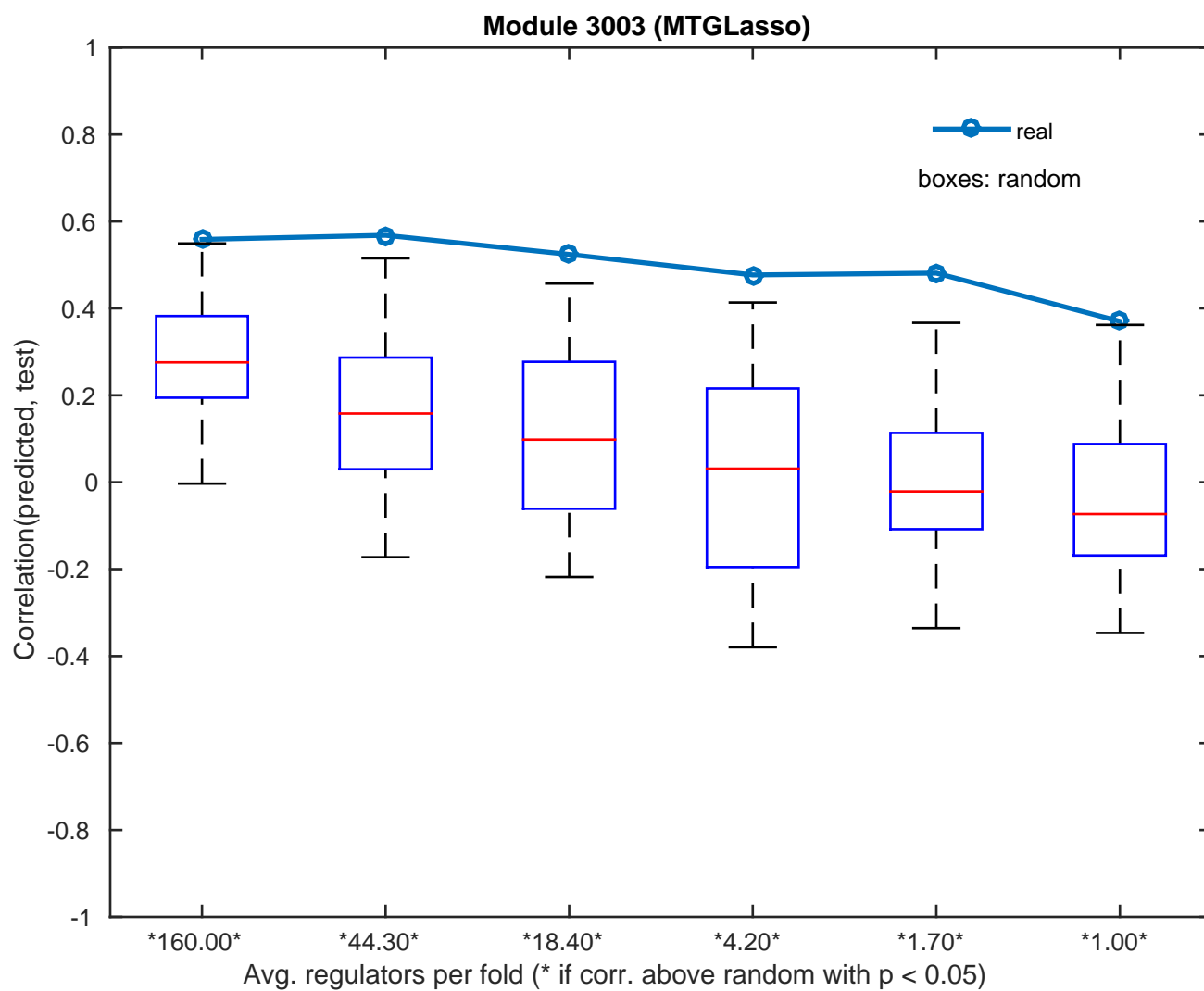

Supplement: S1 Dataset — One plot per module, both species. (GZ) [file pcbi.1005013.s024.gz › mouse_correlation_vs_lambda/mouse_module3003_mtglasso_all_lambdas.pdf]

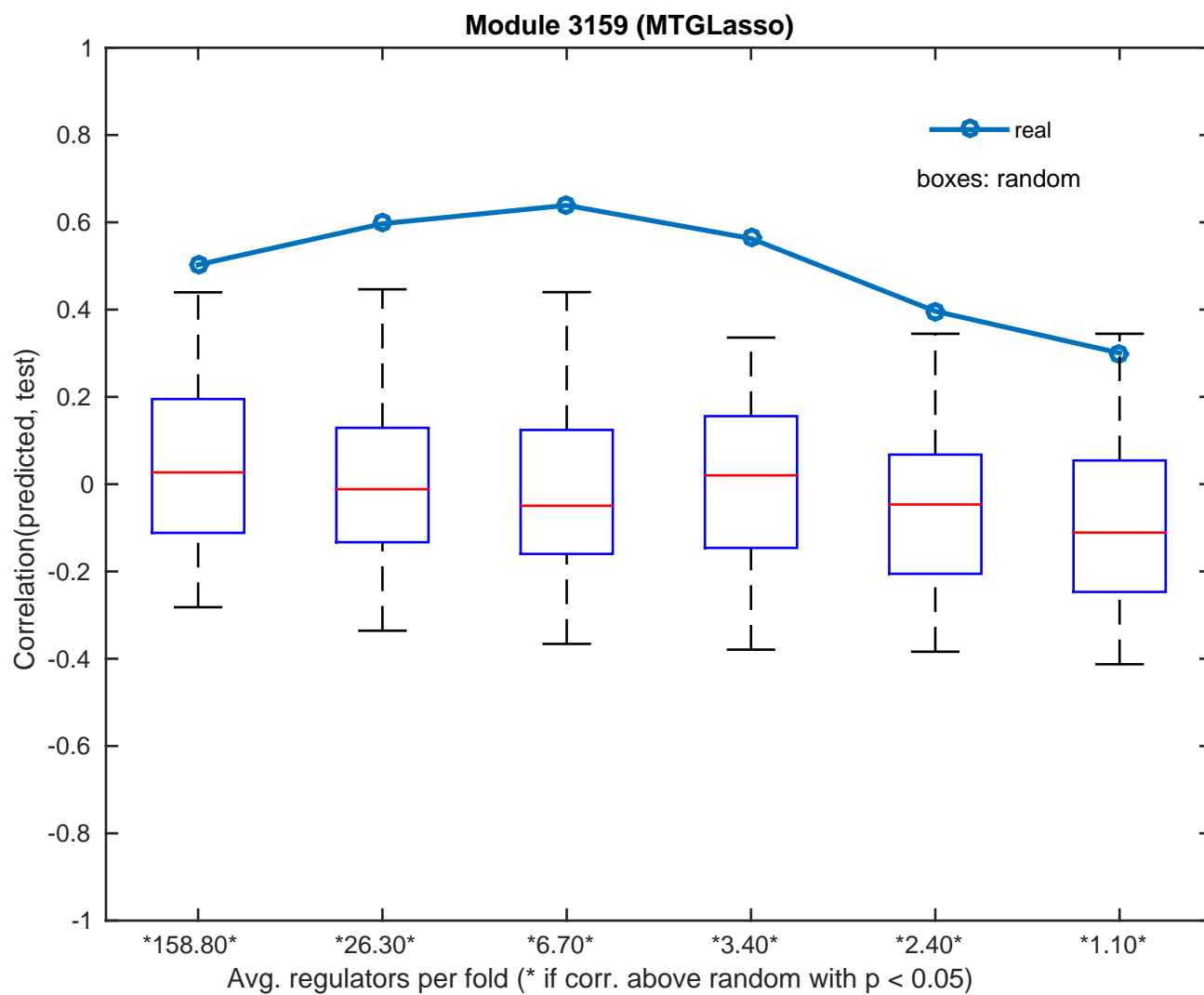

Supplement: S1 Dataset — One plot per module, both species. (GZ) [file pcbi.1005013.s024.gz › mouse_correlation_vs_lambda/mouse_module3159_mtglasso_all_lambdas.pdf]

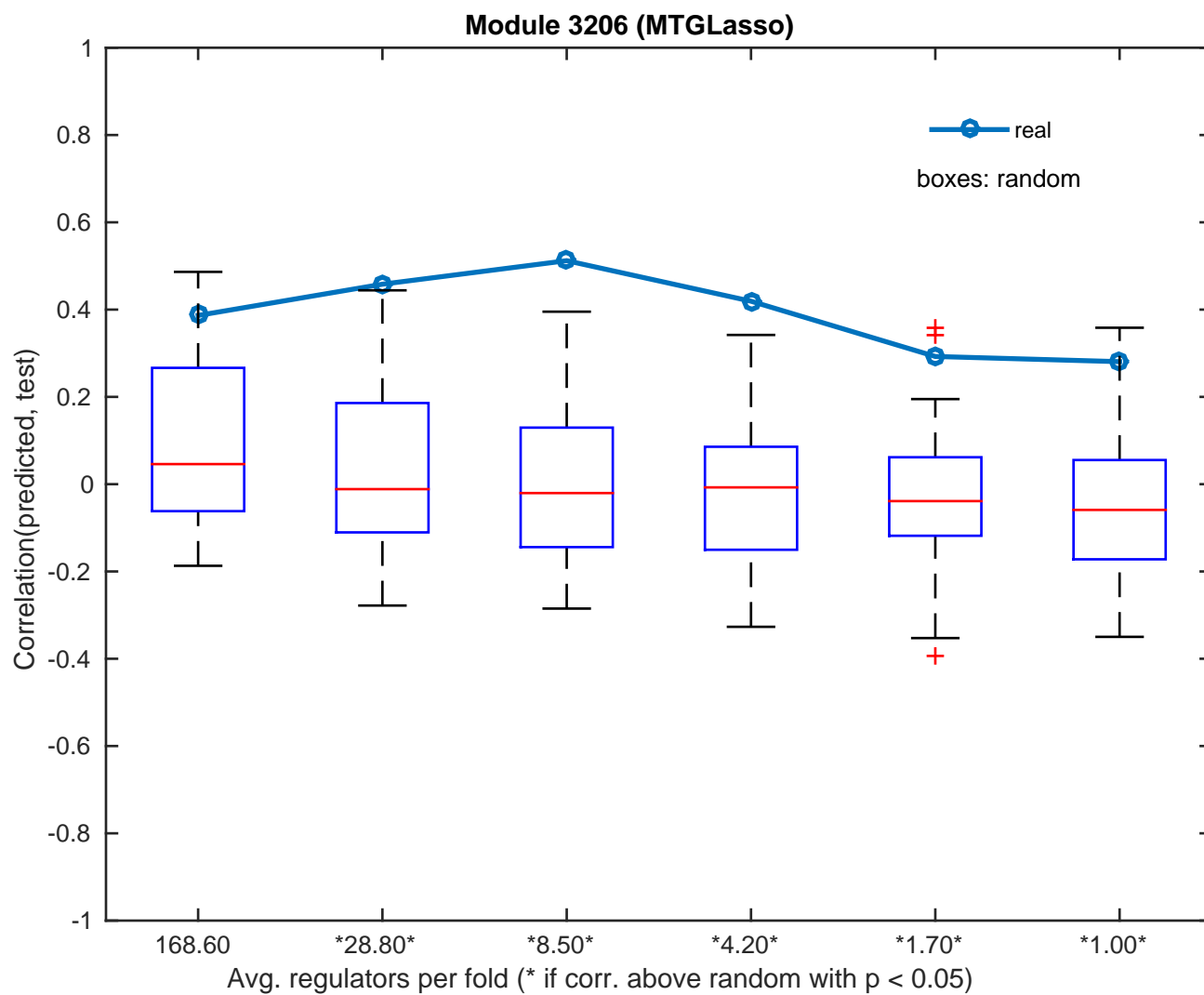

Supplement: S1 Dataset — One plot per module, both species. (GZ) [file pcbi.1005013.s024.gz › mouse_correlation_vs_lambda/mouse_module3206_mtglasso_all_lambdas.pdf]

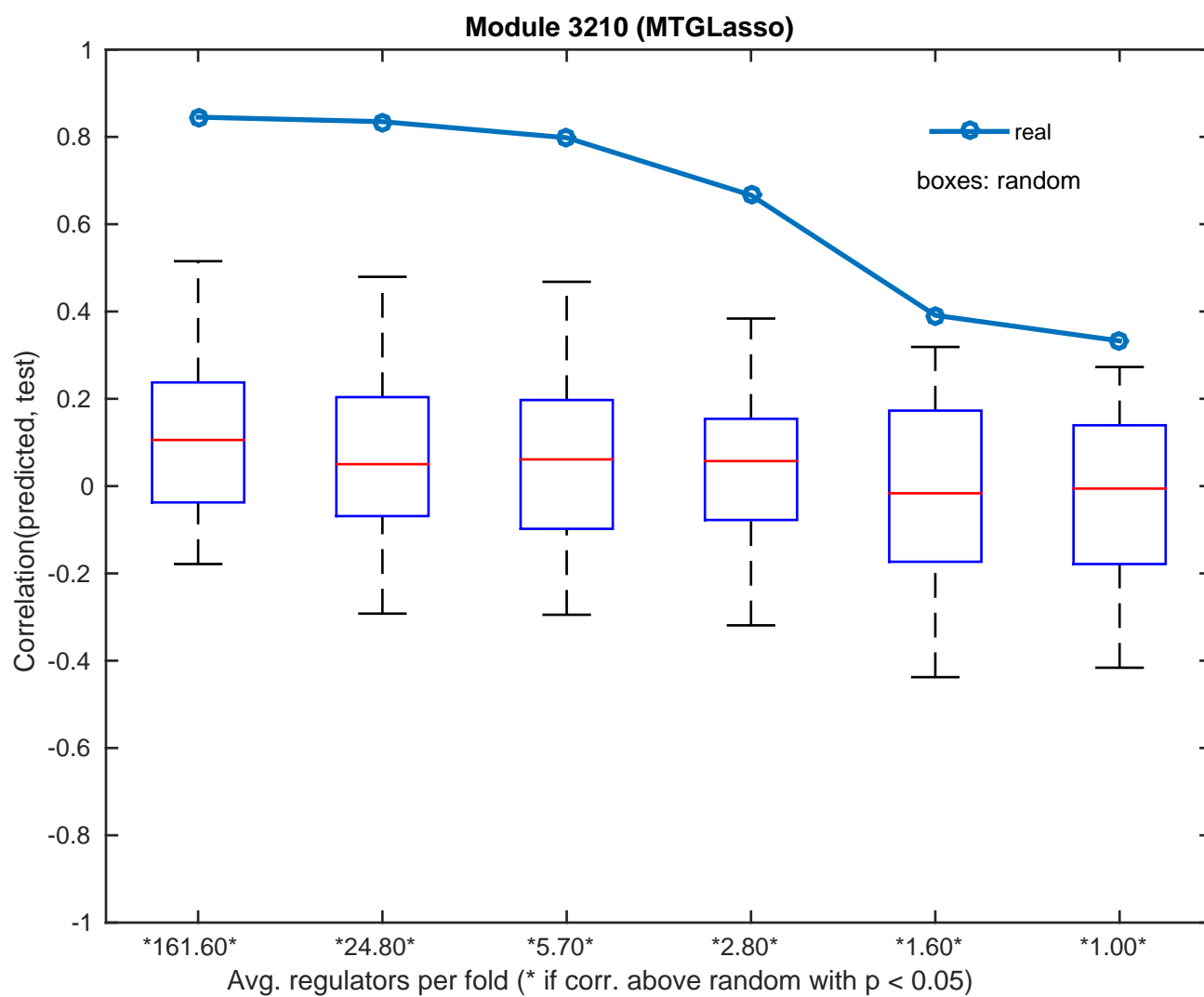

Supplement: S1 Dataset — One plot per module, both species. (GZ) [file pcbi.1005013.s024.gz › mouse_correlation_vs_lambda/mouse_module3210_mtglasso_all_lambdas.pdf]

Module 2899 (MTGLasso)

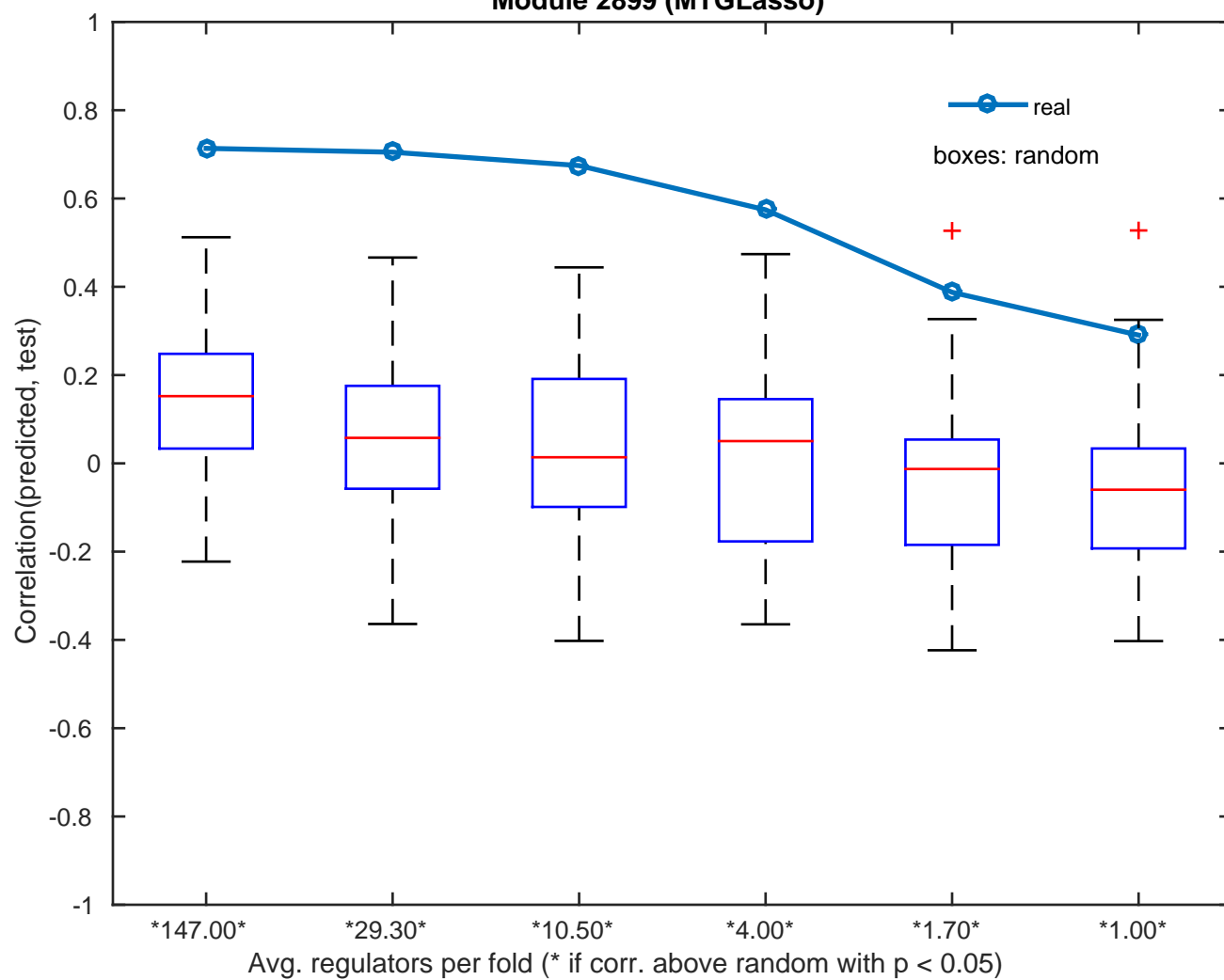

Supplement: S1 Dataset — One plot per module, both species. (GZ) [file pcbi.1005013.s024.gz › mouse_correlation_vs_lambda/mouse_module2899_mtglasso_all_lambdas.pdf]

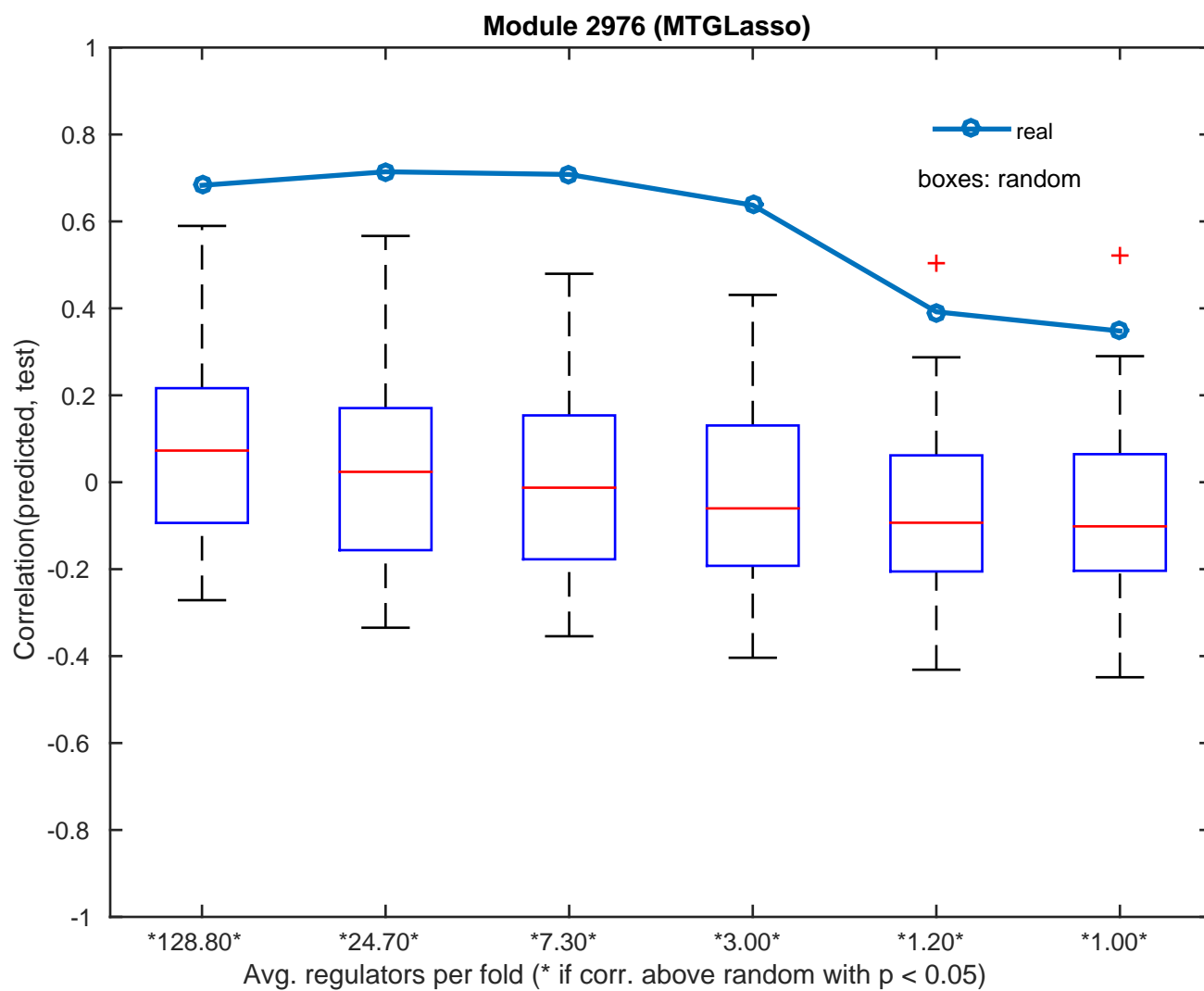

Supplement: S1 Dataset — One plot per module, both species. (GZ) [file pcbi.1005013.s024.gz › mouse_correlation_vs_lambda/mouse_module2976_mtglasso_all_lambdas.pdf]

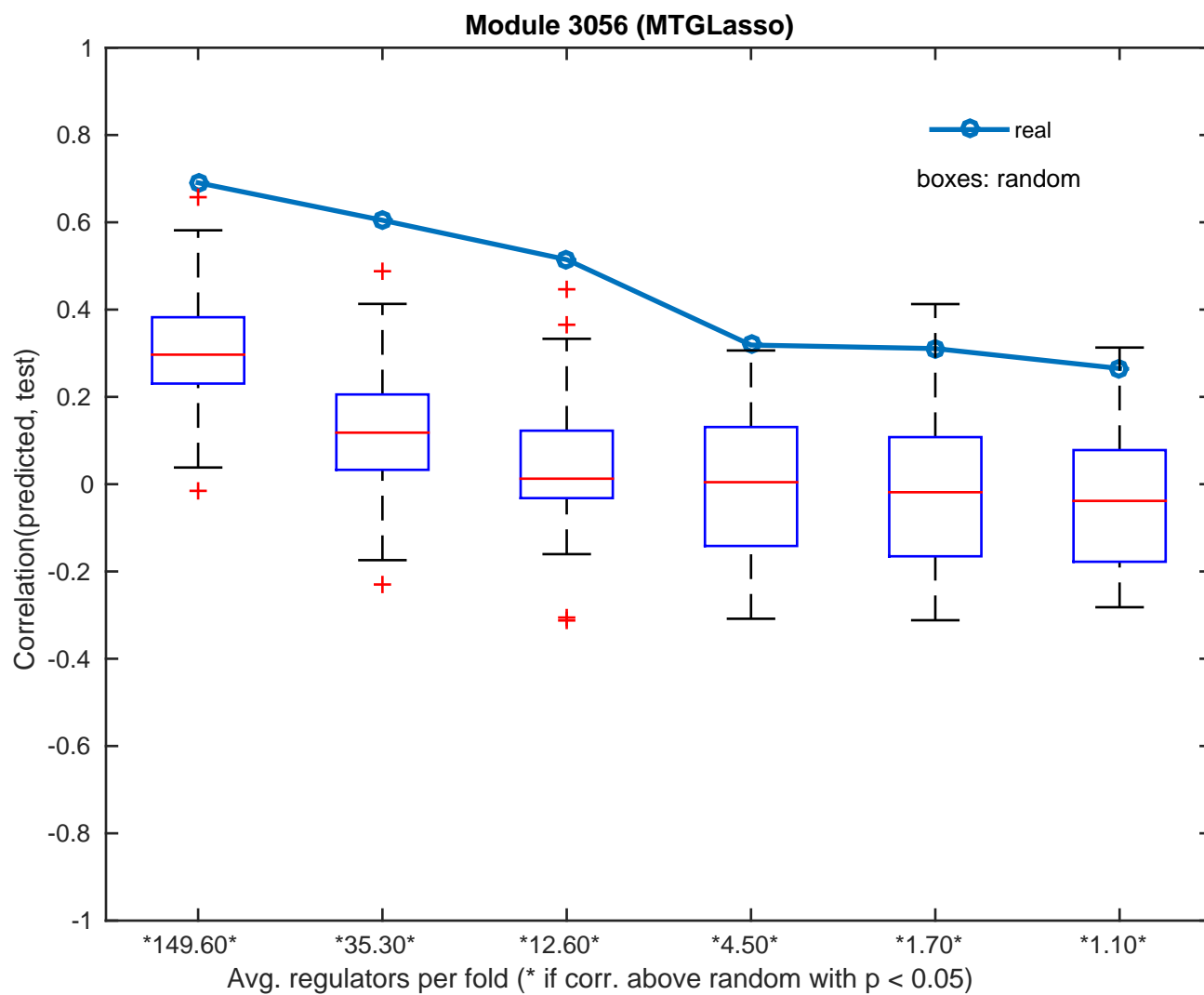

Supplement: S1 Dataset — One plot per module, both species. (GZ) [file pcbi.1005013.s024.gz › mouse_correlation_vs_lambda/mouse_module3056_mtglasso_all_lambdas.pdf]

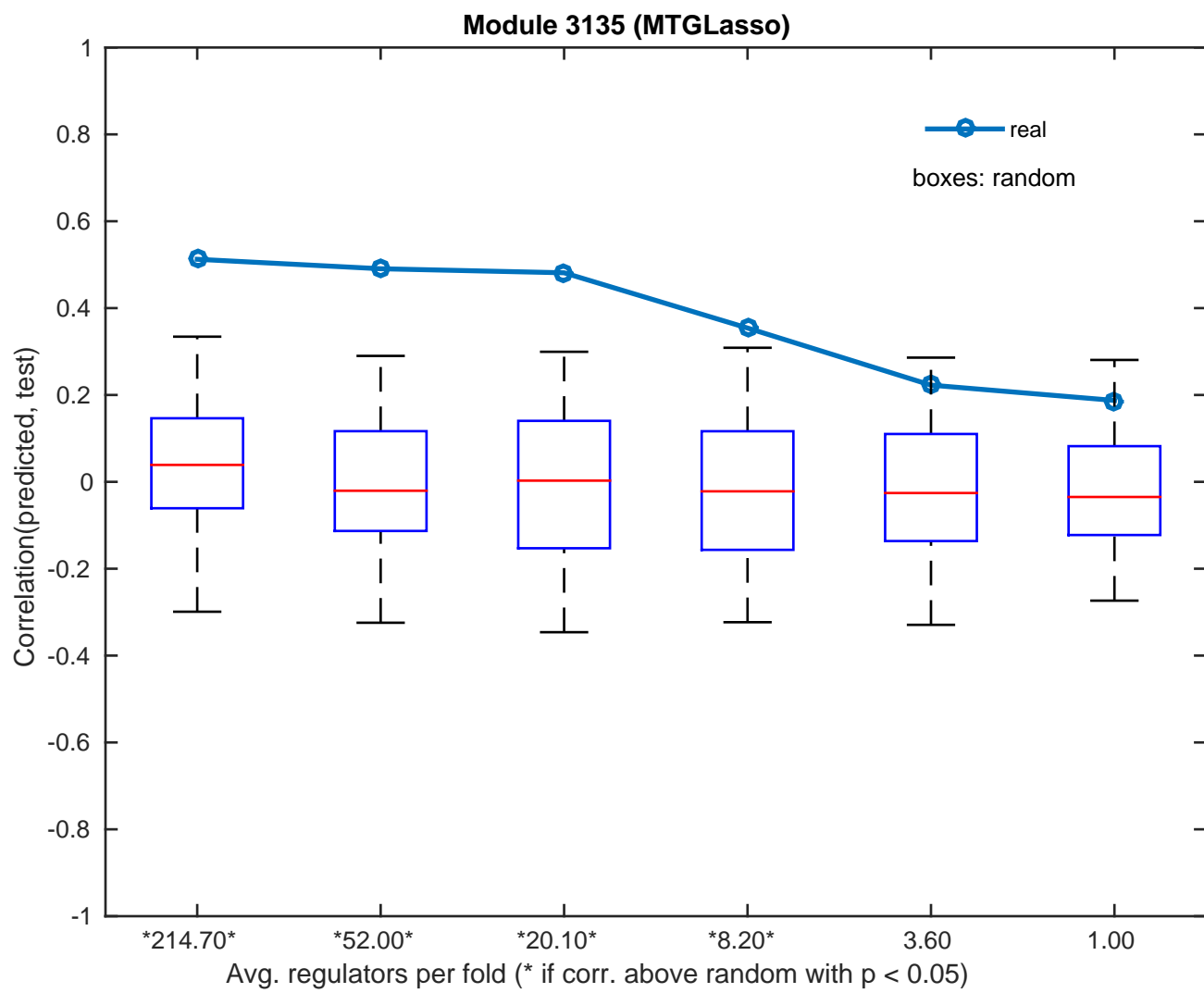

Supplement: S1 Dataset — One plot per module, both species. (GZ) [file pcbi.1005013.s024.gz › mouse_correlation_vs_lambda/mouse_module3135_mtglasso_all_lambdas.pdf]

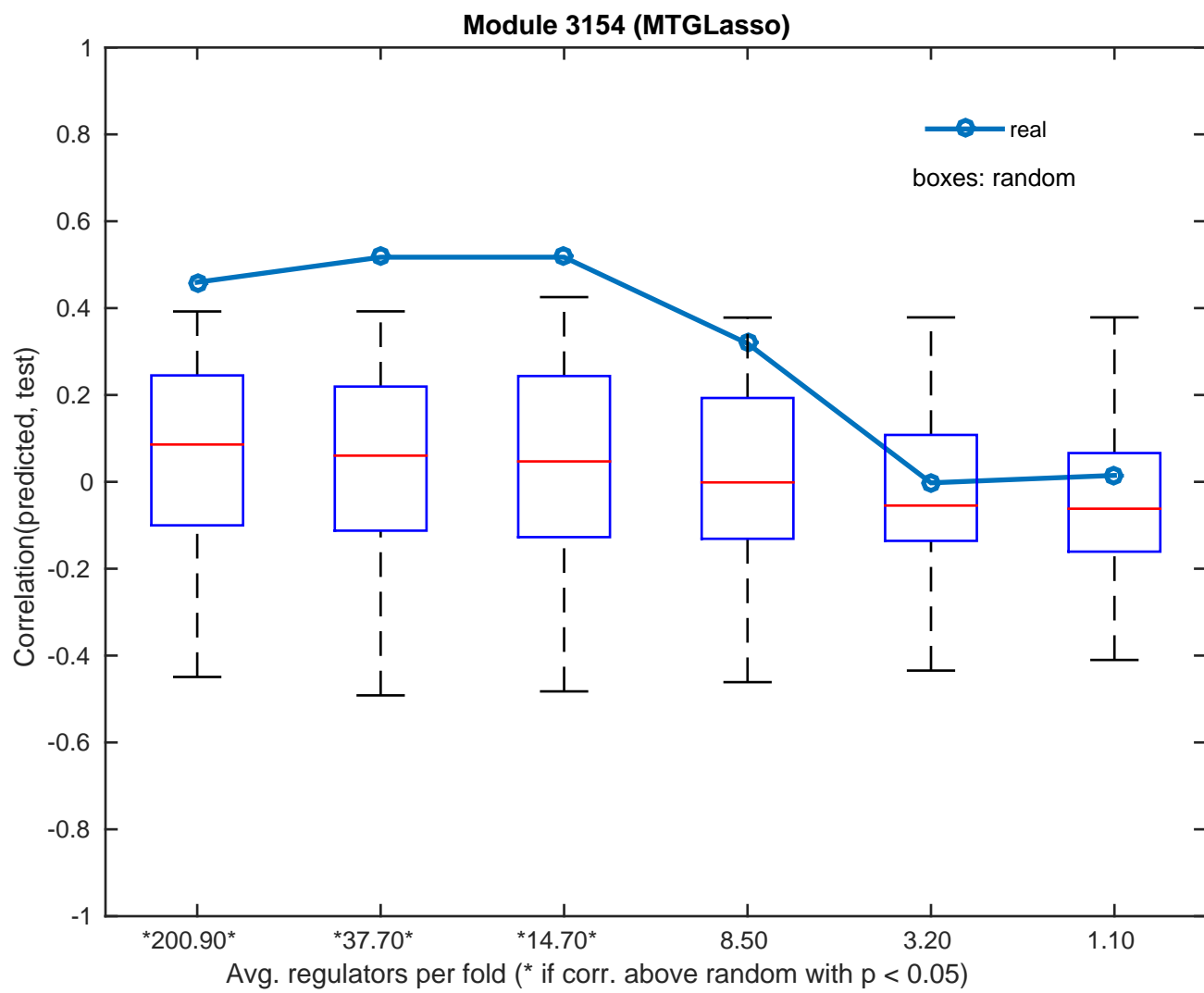

Supplement: S1 Dataset — One plot per module, both species. (GZ) [file pcbi.1005013.s024.gz › mouse_correlation_vs_lambda/mouse_module3154_mtglasso_all_lambdas.pdf]

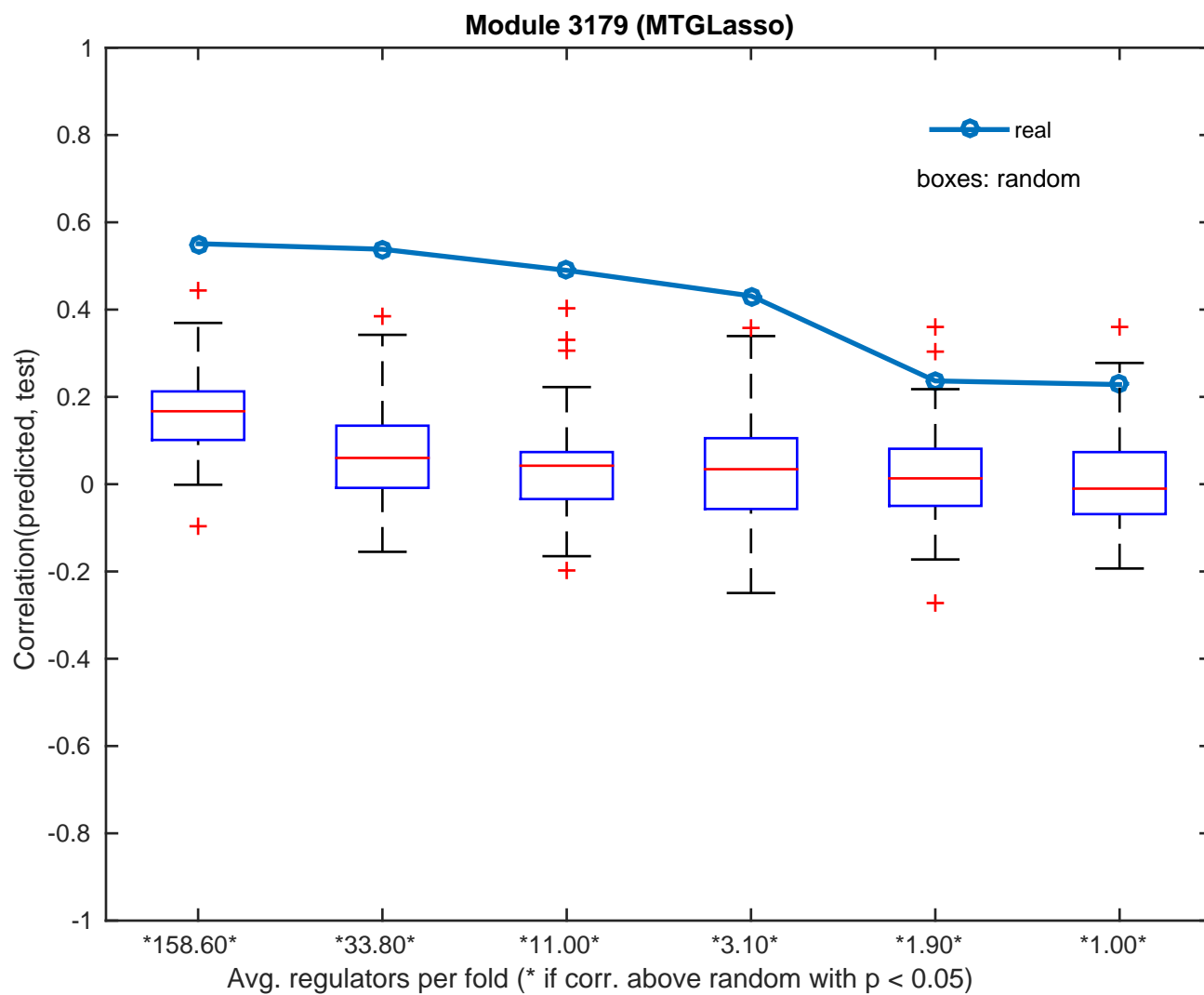

Supplement: S1 Dataset — One plot per module, both species. (GZ) [file pcbi.1005013.s024.gz › mouse_correlation_vs_lambda/mouse_module3179_mtglasso_all_lambdas.pdf]

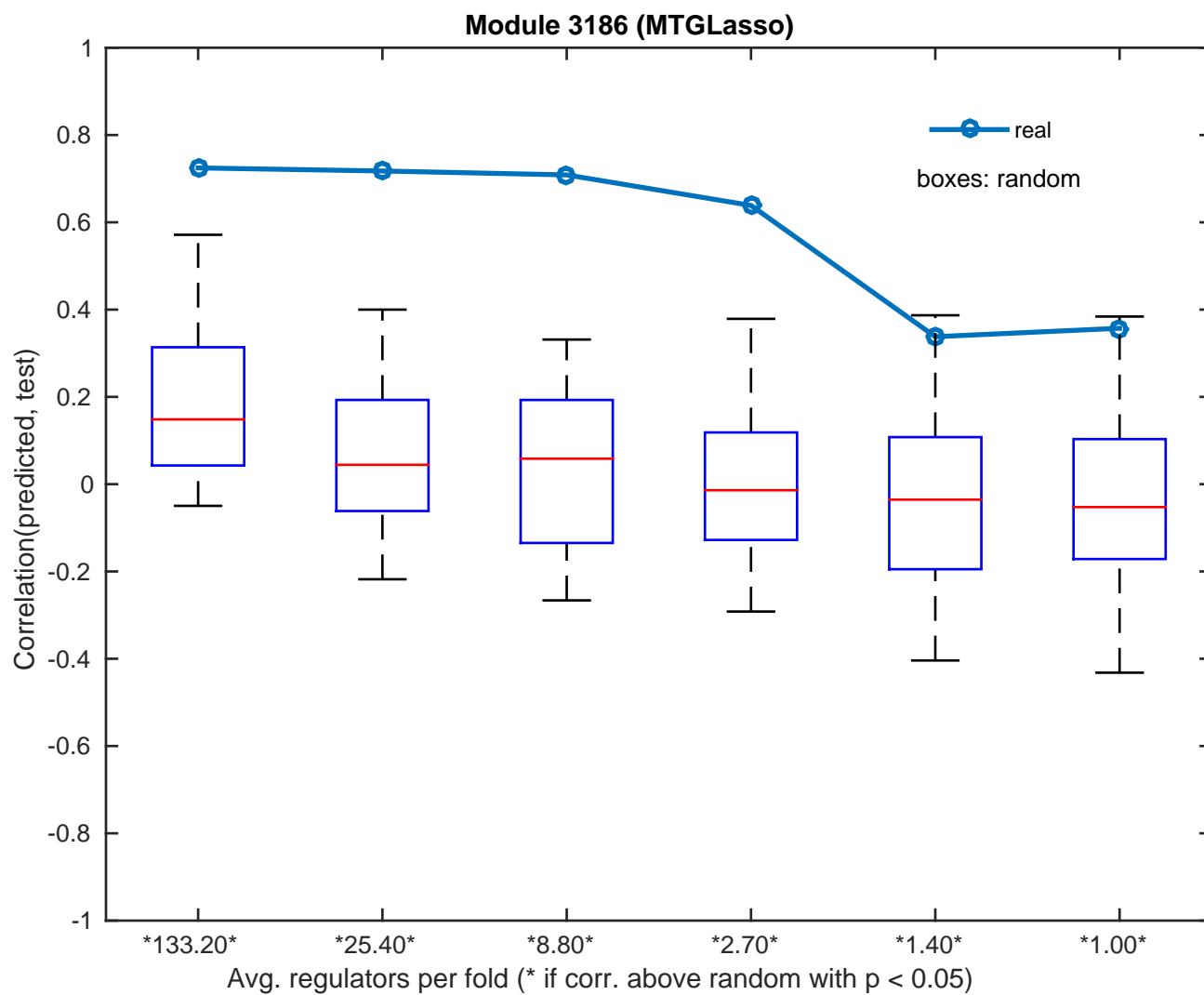

Supplement: S1 Dataset — One plot per module, both species. (GZ) [file pcbi.1005013.s024.gz › mouse_correlation_vs_lambda/mouse_module3186_mtglasso_all_lambdas.pdf]

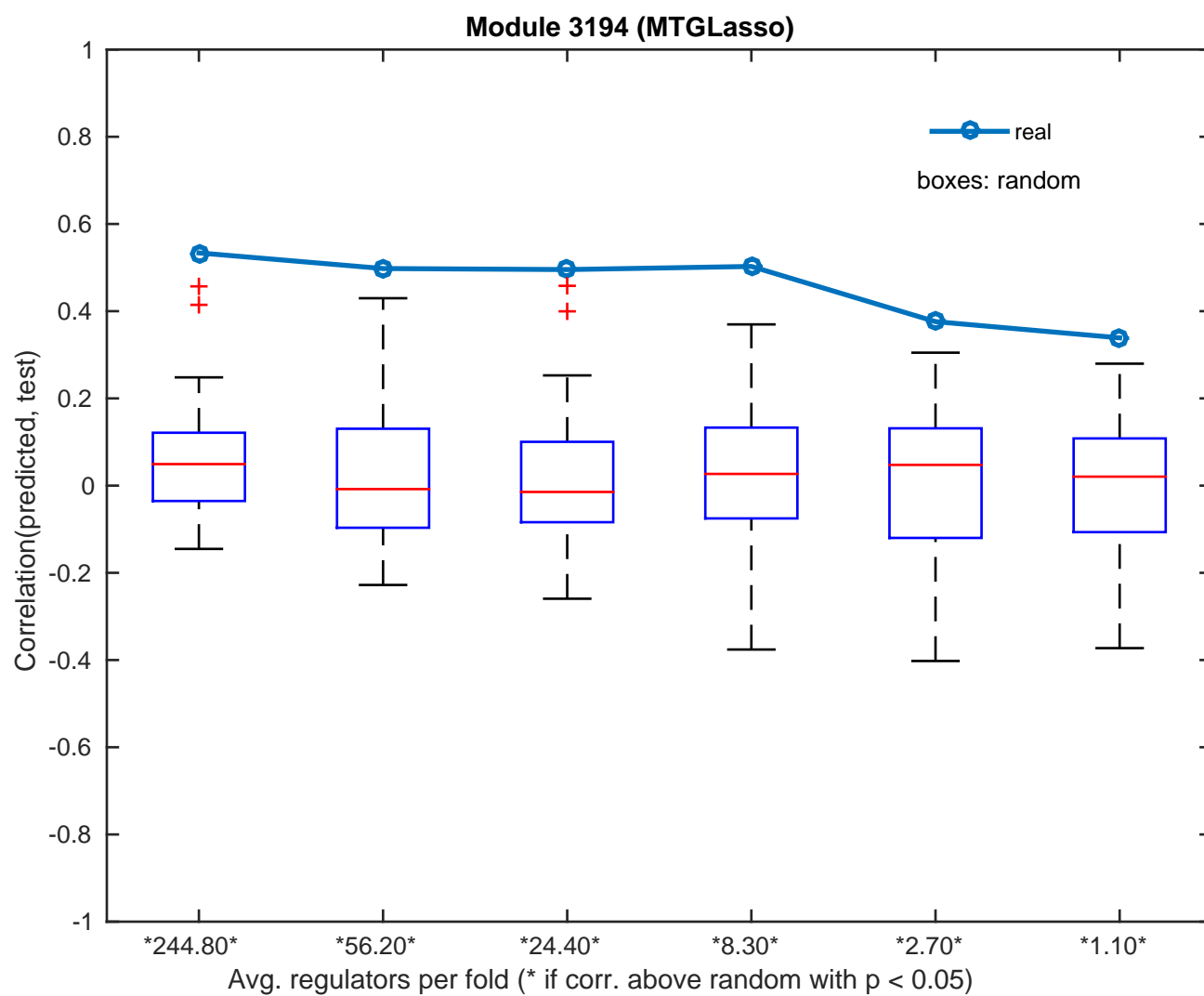

Supplement: S1 Dataset — One plot per module, both species. (GZ) [file pcbi.1005013.s024.gz › mouse_correlation_vs_lambda/mouse_module3194_mtglasso_all_lambdas.pdf]

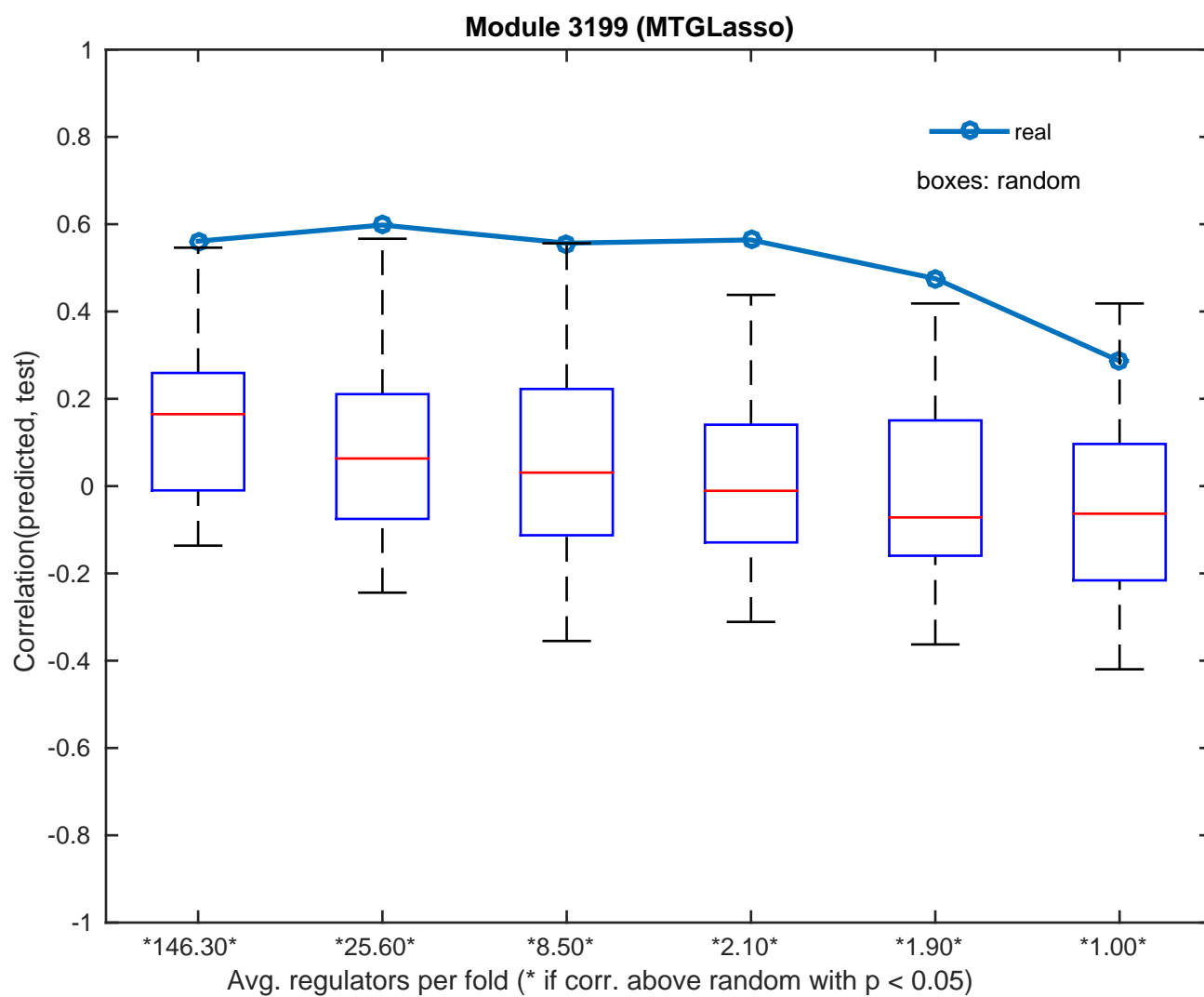

Supplement: S1 Dataset — One plot per module, both species. (GZ) [file pcbi.1005013.s024.gz › mouse_correlation_vs_lambda/mouse_module3199_mtglasso_all_lambdas.pdf]

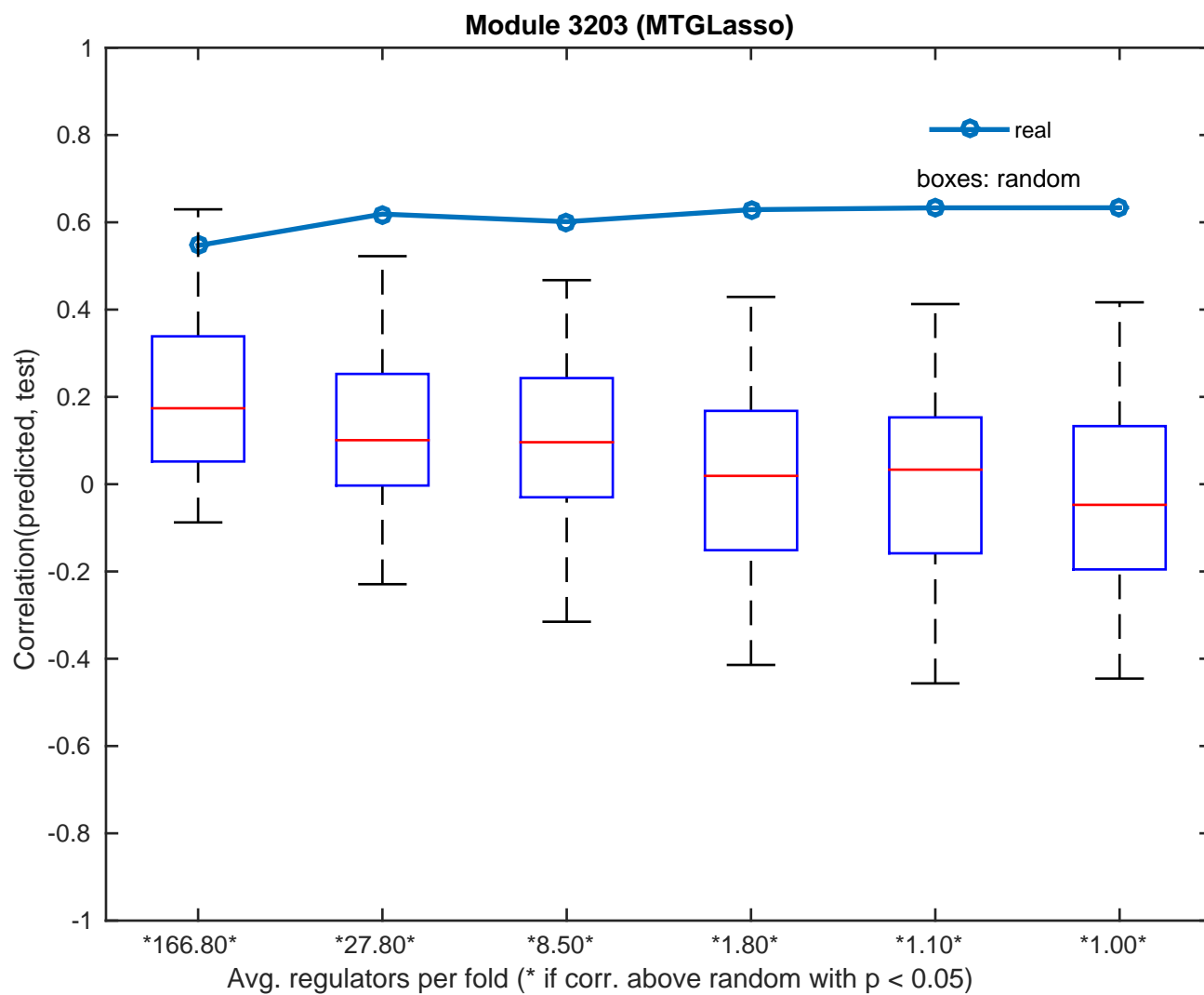

Supplement: S1 Dataset — One plot per module, both species. (GZ) [file pcbi.1005013.s024.gz › mouse_correlation_vs_lambda/mouse_module3203_mtglasso_all_lambdas.pdf]

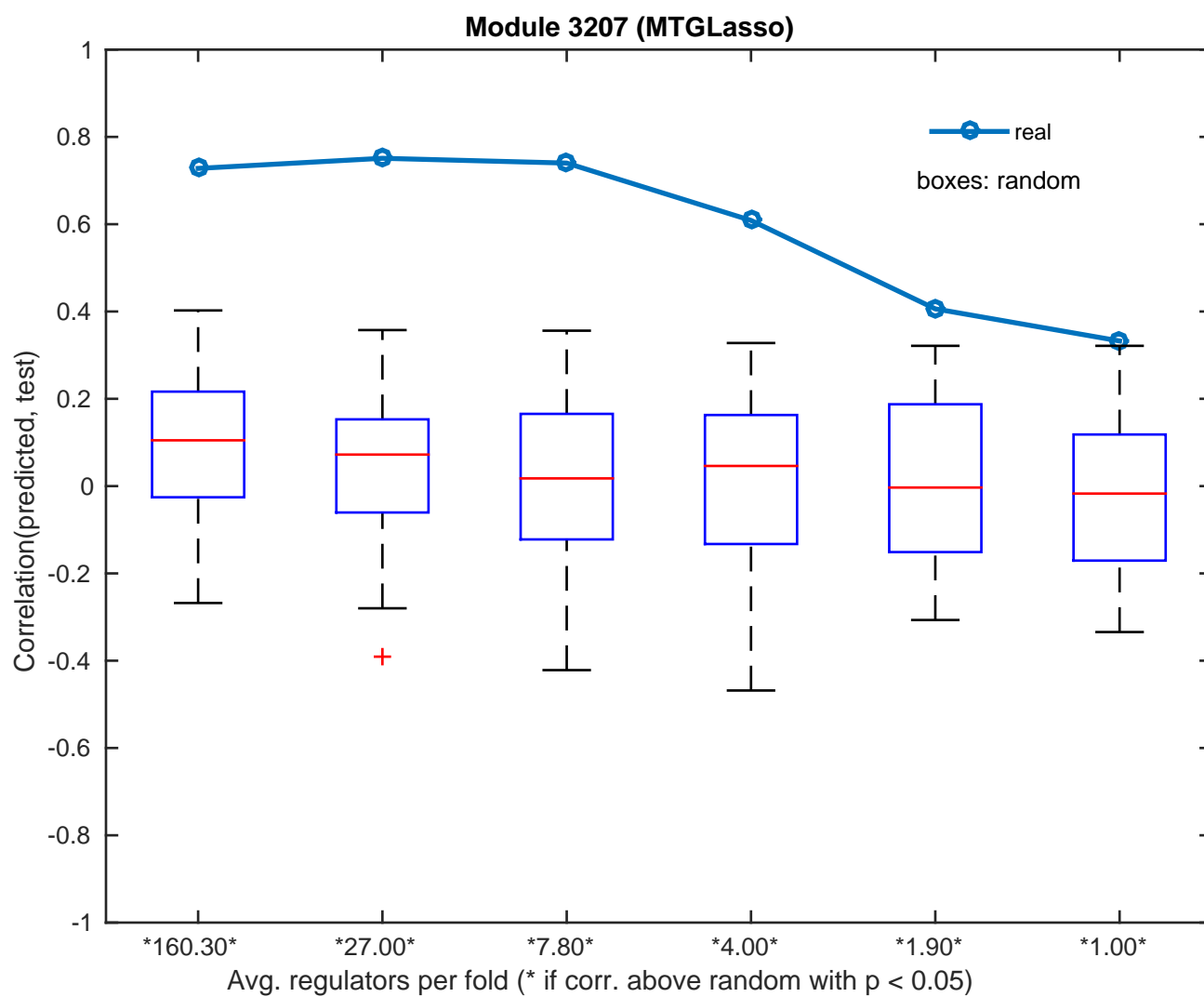

Supplement: S1 Dataset — One plot per module, both species. (GZ) [file pcbi.1005013.s024.gz › mouse_correlation_vs_lambda/mouse_module3207_mtglasso_all_lambdas.pdf]

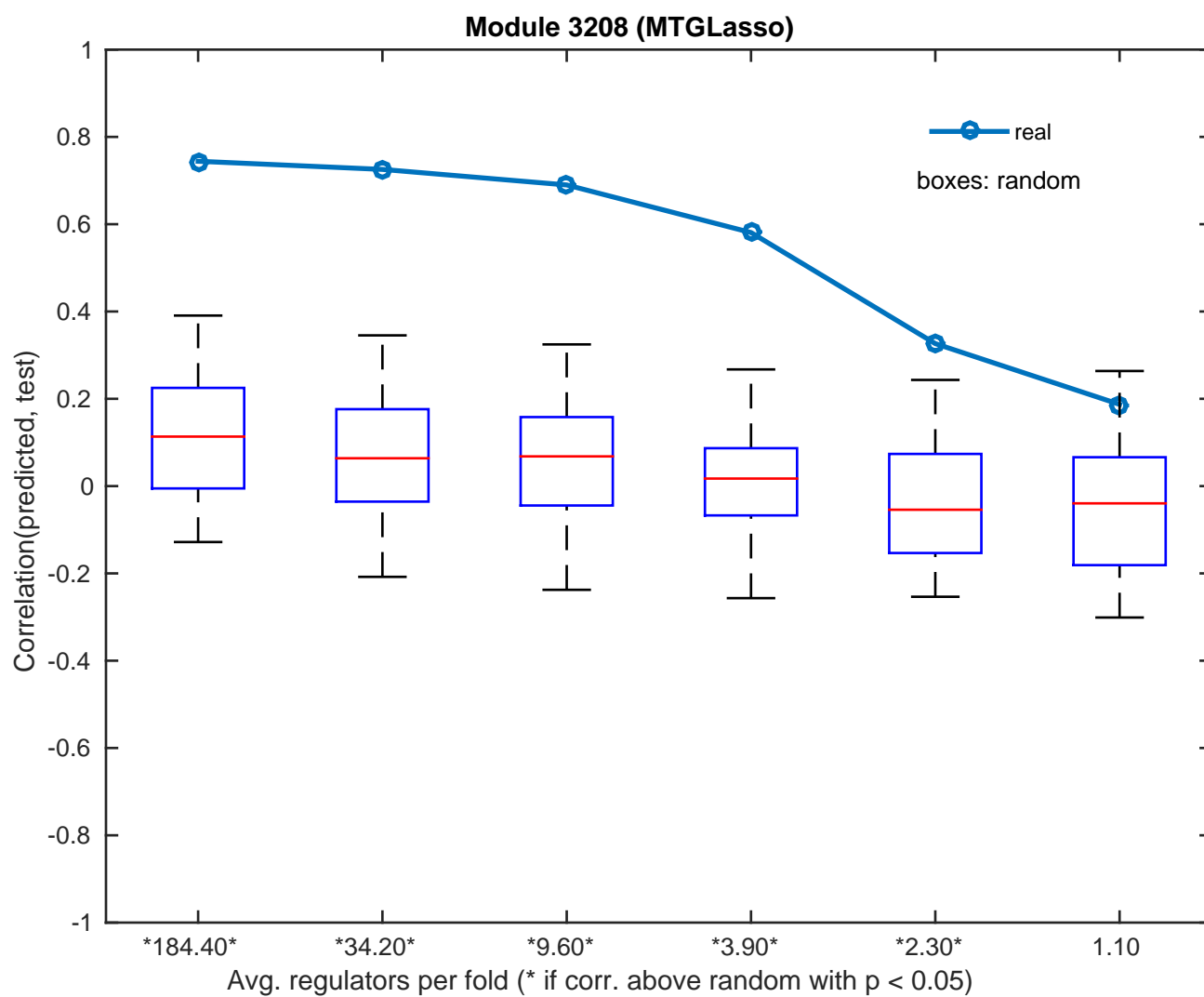

Supplement: S1 Dataset — One plot per module, both species. (GZ) [file pcbi.1005013.s024.gz › mouse_correlation_vs_lambda/mouse_module3208_mtglasso_all_lambdas.pdf]

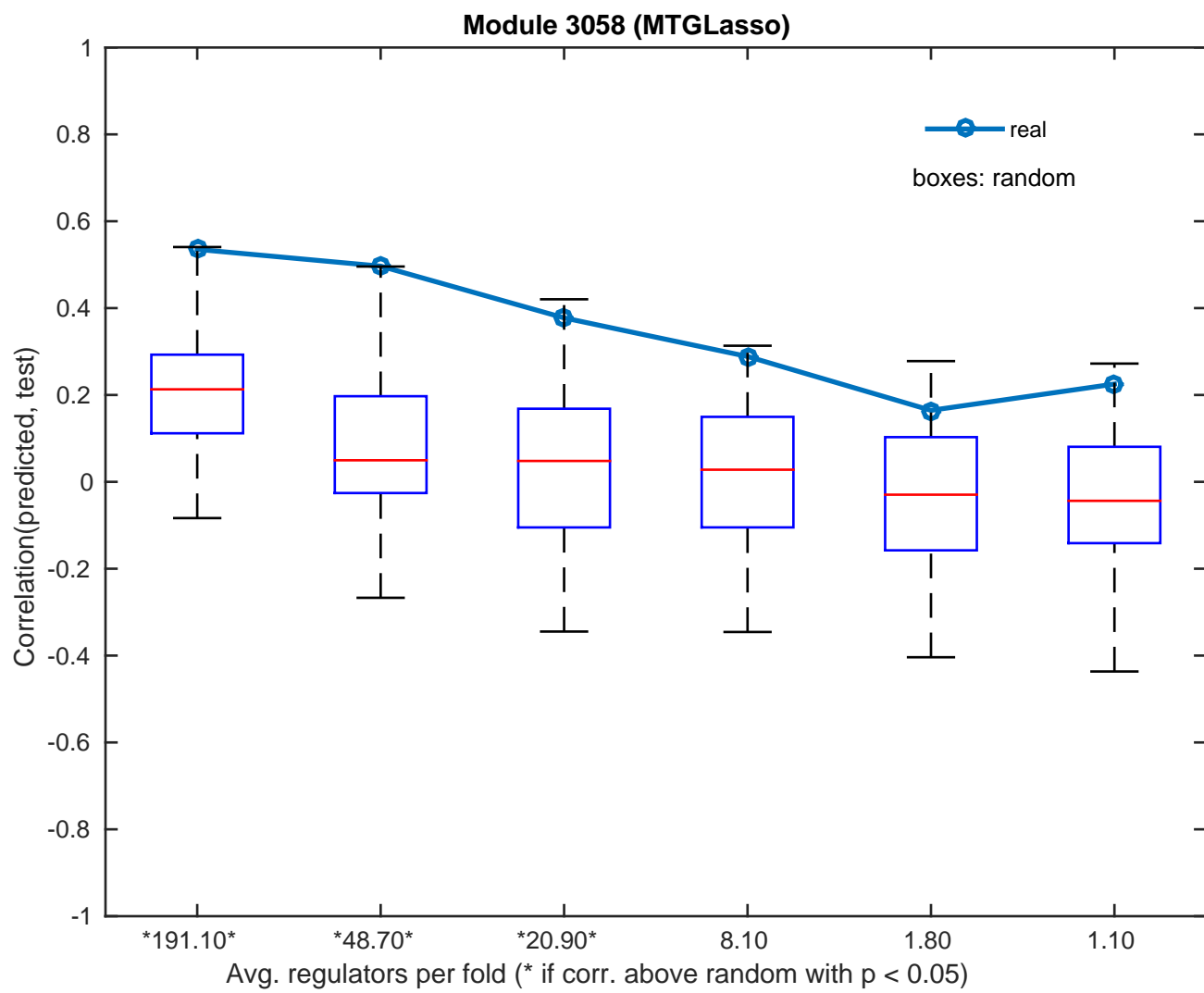

Supplement: S1 Dataset — One plot per module, both species. (GZ) [file pcbi.1005013.s024.gz › mouse_correlation_vs_lambda/mouse_module3058_mtglasso_all_lambdas.pdf]

Module 3062 (MTGLasso)

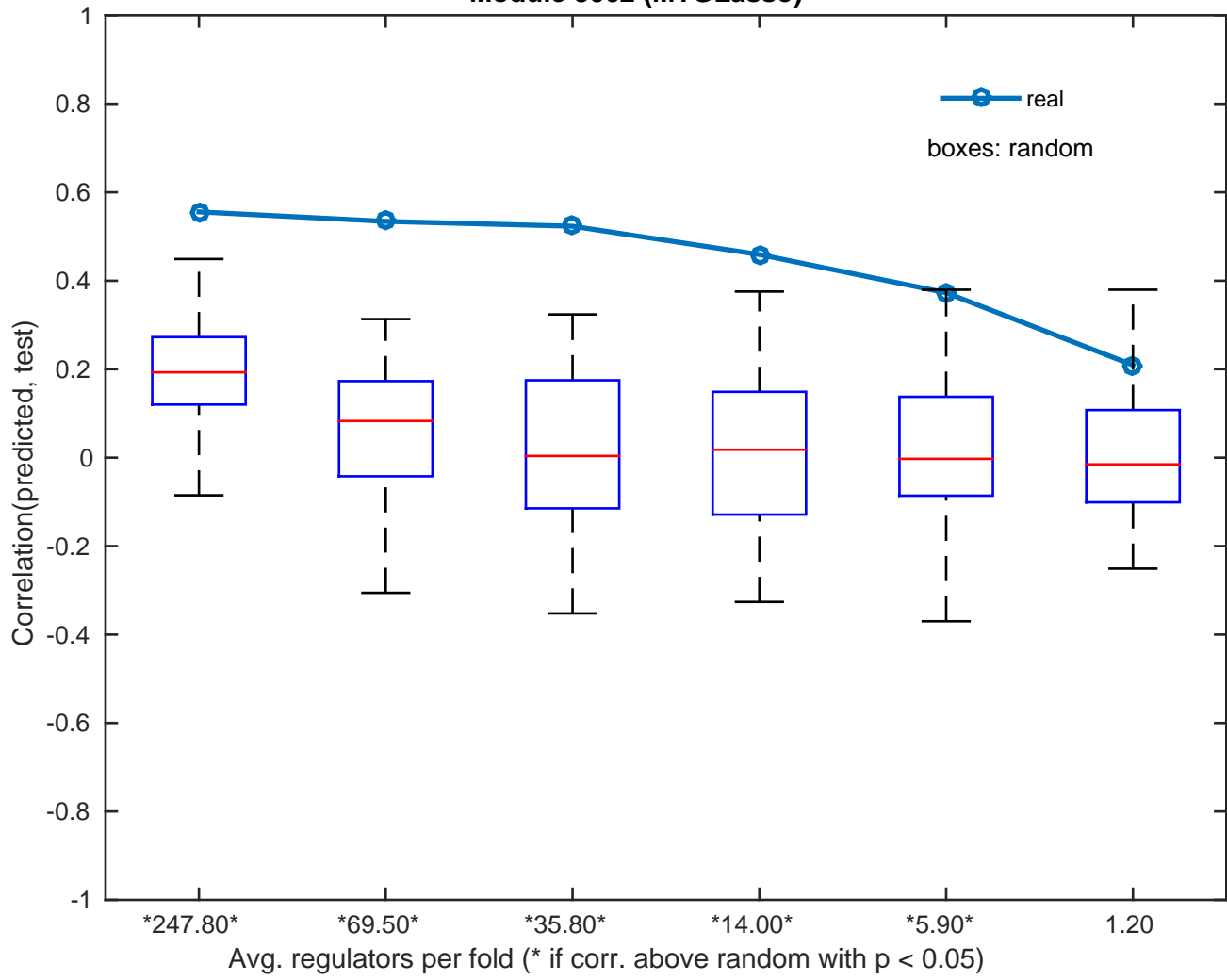

Supplement: S1 Dataset — One plot per module, both species. (GZ) [file pcbi.1005013.s024.gz › mouse_correlation_vs_lambda/mouse_module3062_mtglasso_all_lambdas.pdf]

Module 3134 (MTGLasso)

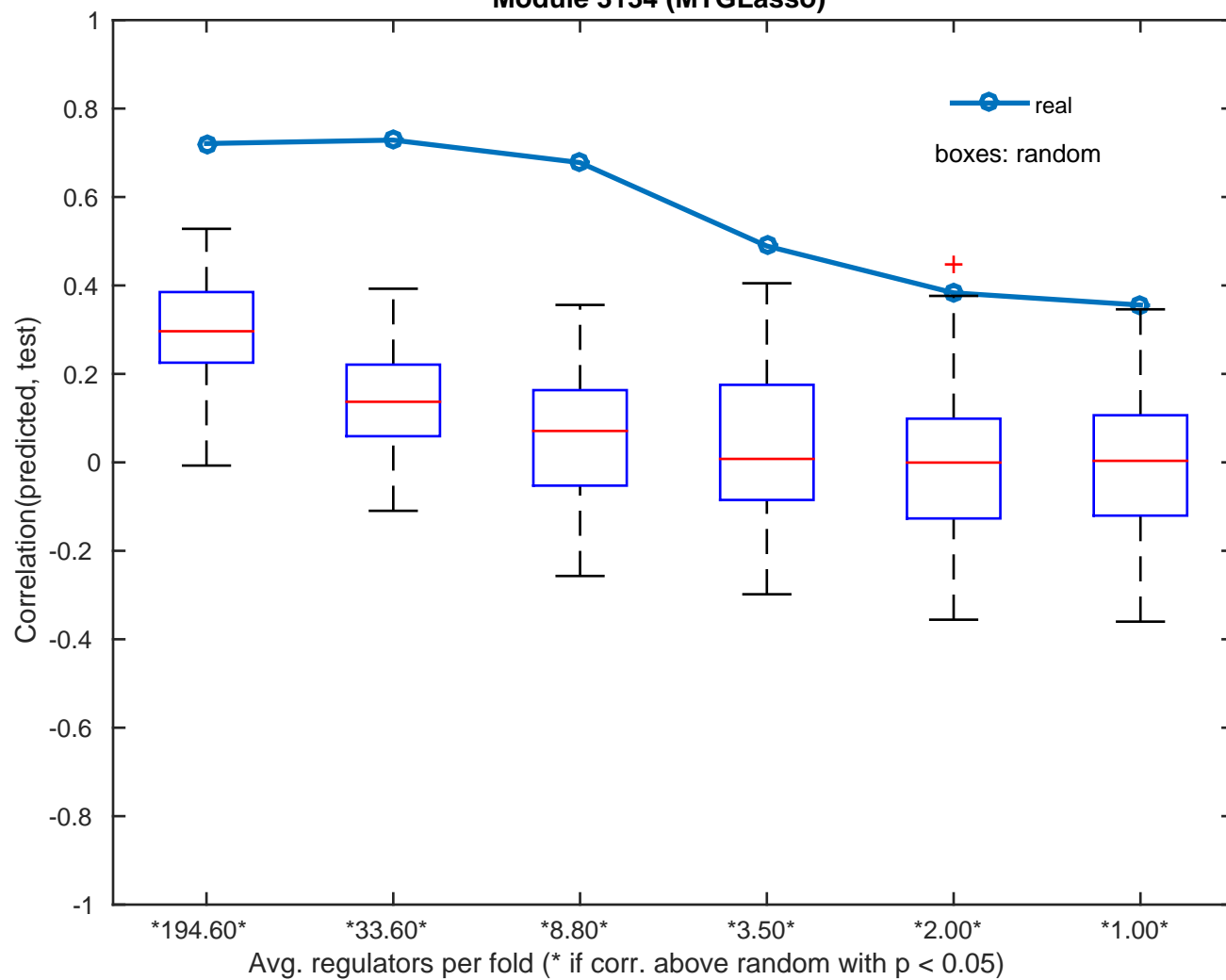

Supplement: S1 Dataset — One plot per module, both species. (GZ) [file pcbi.1005013.s024.gz › mouse_correlation_vs_lambda/mouse_module3134_mtglasso_all_lambdas.pdf]

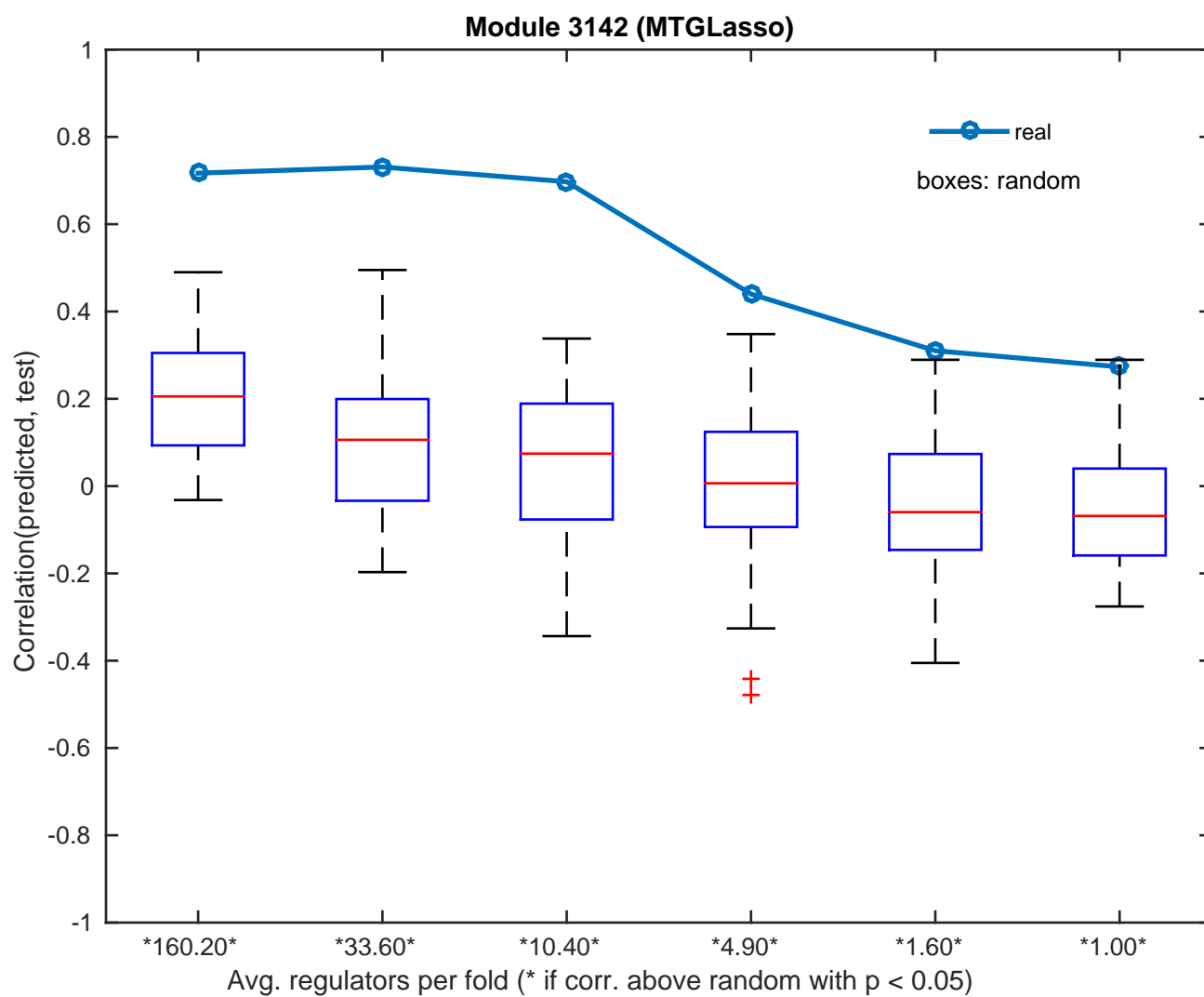

Supplement: S1 Dataset — One plot per module, both species. (GZ) [file pcbi.1005013.s024.gz › mouse_correlation_vs_lambda/mouse_module3142_mtglasso_all_lambdas.pdf]

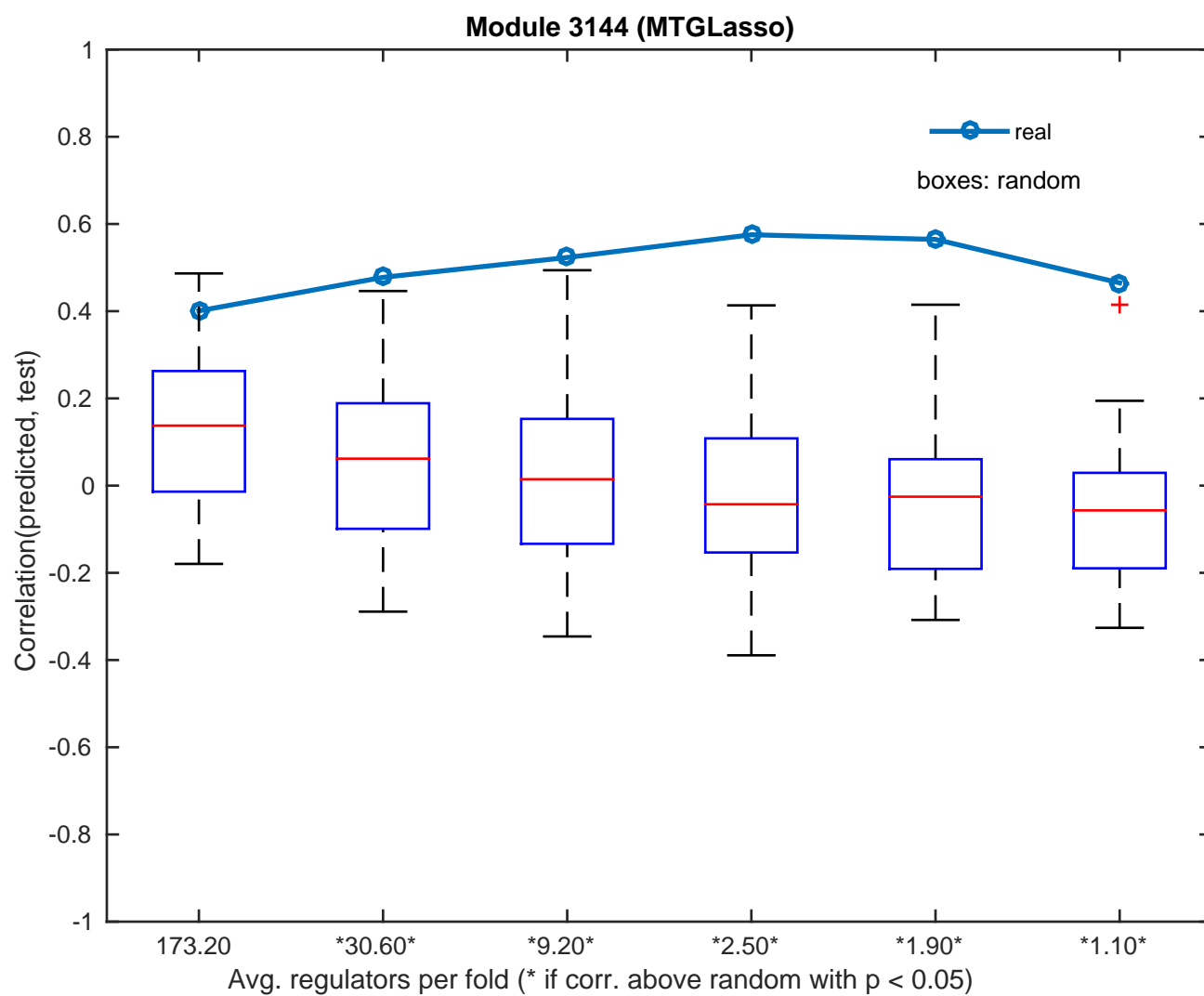

Supplement: S1 Dataset — One plot per module, both species. (GZ) [file pcbi.1005013.s024.gz › mouse_correlation_vs_lambda/mouse_module3144_mtglasso_all_lambdas.pdf]

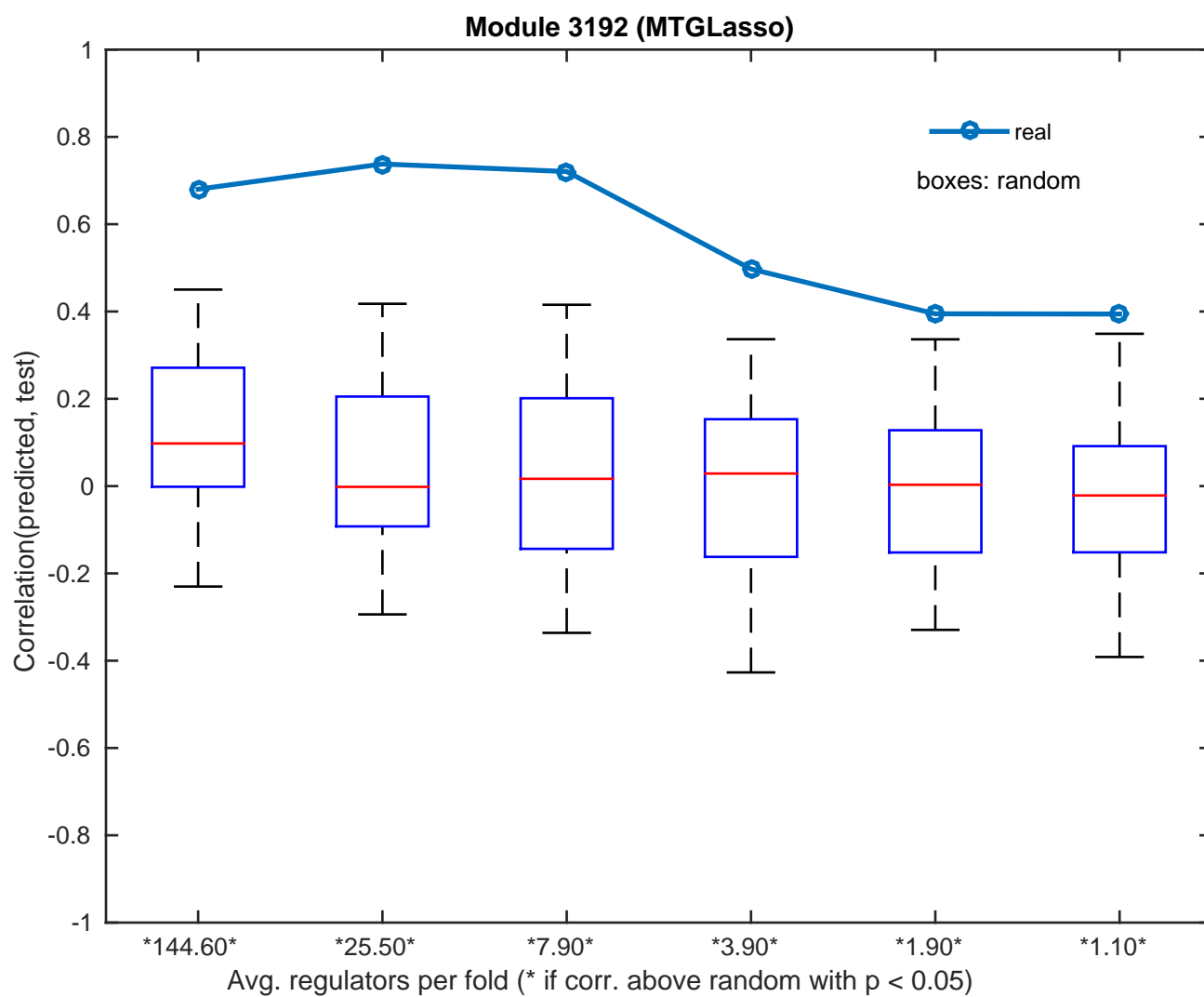

Supplement: S1 Dataset — One plot per module, both species. (GZ) [file pcbi.1005013.s024.gz › mouse_correlation_vs_lambda/mouse_module3192_mtglasso_all_lambdas.pdf]

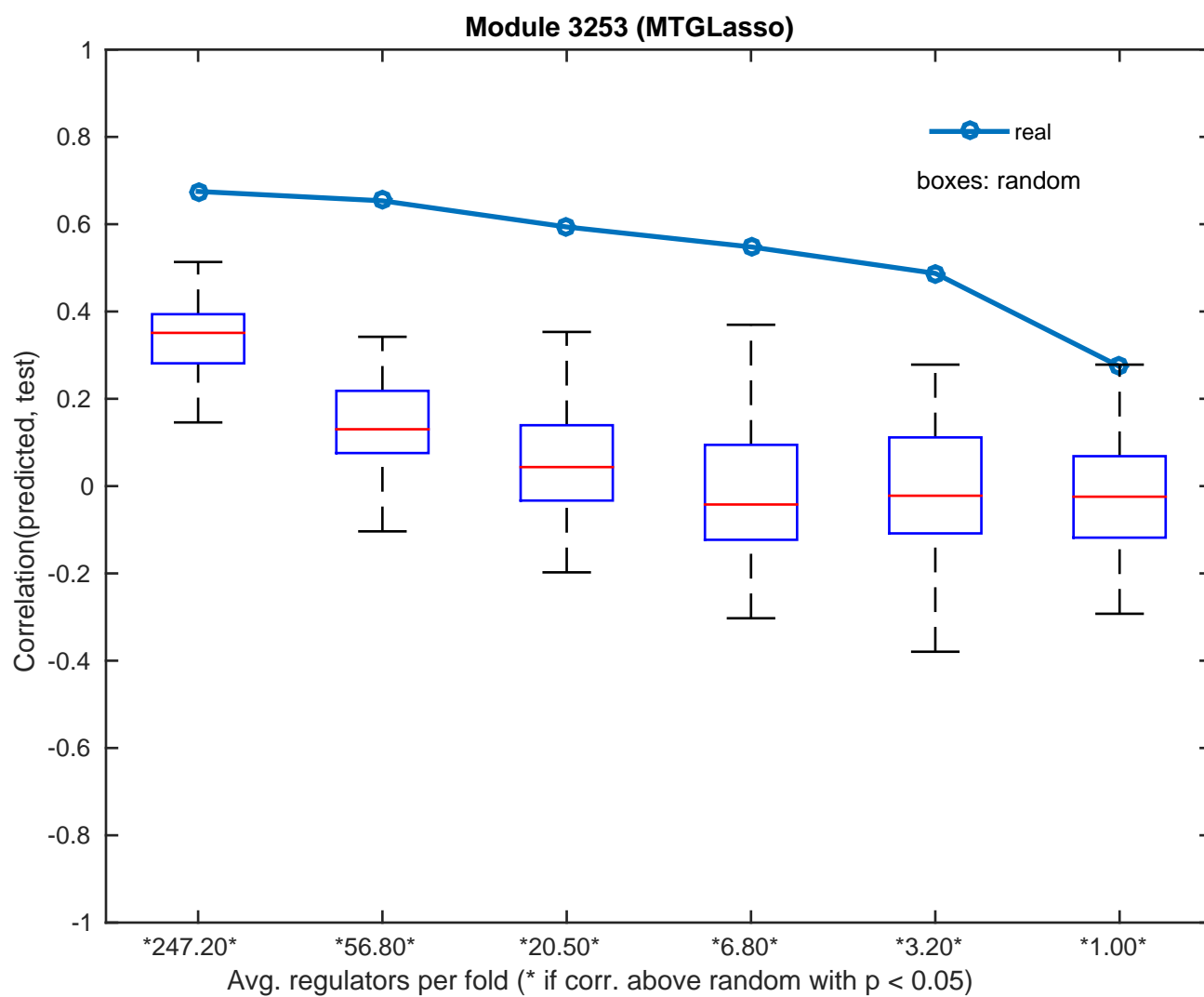

Supplement: S1 Dataset — One plot per module, both species. (GZ) [file pcbi.1005013.s024.gz › mouse_correlation_vs_lambda/mouse_module3253_mtglasso_all_lambdas.pdf]
