# Supplementary material for: Integrating Transcriptomic and Proteomic Data Using Predictive Regulatory Network Models of Host Response to Pathogens
Source: PLoS Comput Biol. 2016 Jul 12;12(7):e1005013. doi: 10.1371/journal.pcbi.1005013 (PMC4942116; doi:10.1371/journal.pcbi.1005013)
Supplement: S3 Fig — Human Calu-3 (A-B), mouse (C-D). Each column is a sample from one of the virus treatment time courses. Rows in each heatmap are genes in the intersection of the complete mRNA and protein data sets for one system after filtering out entries with >50% missing values; before filtering mRNA data set down to differentially expressed genes only. Genes are sorted by hierarchical clustering with average linkage, Manhattan distance followed by optimal leaf ordering of the mRNA data to enhance visualization of patterns. The ordering between mRNA and protein data is the same. Values are scaled between [–1,1] for all heatmaps. (PDF) [file pcbi.1005013.s014.pdf]

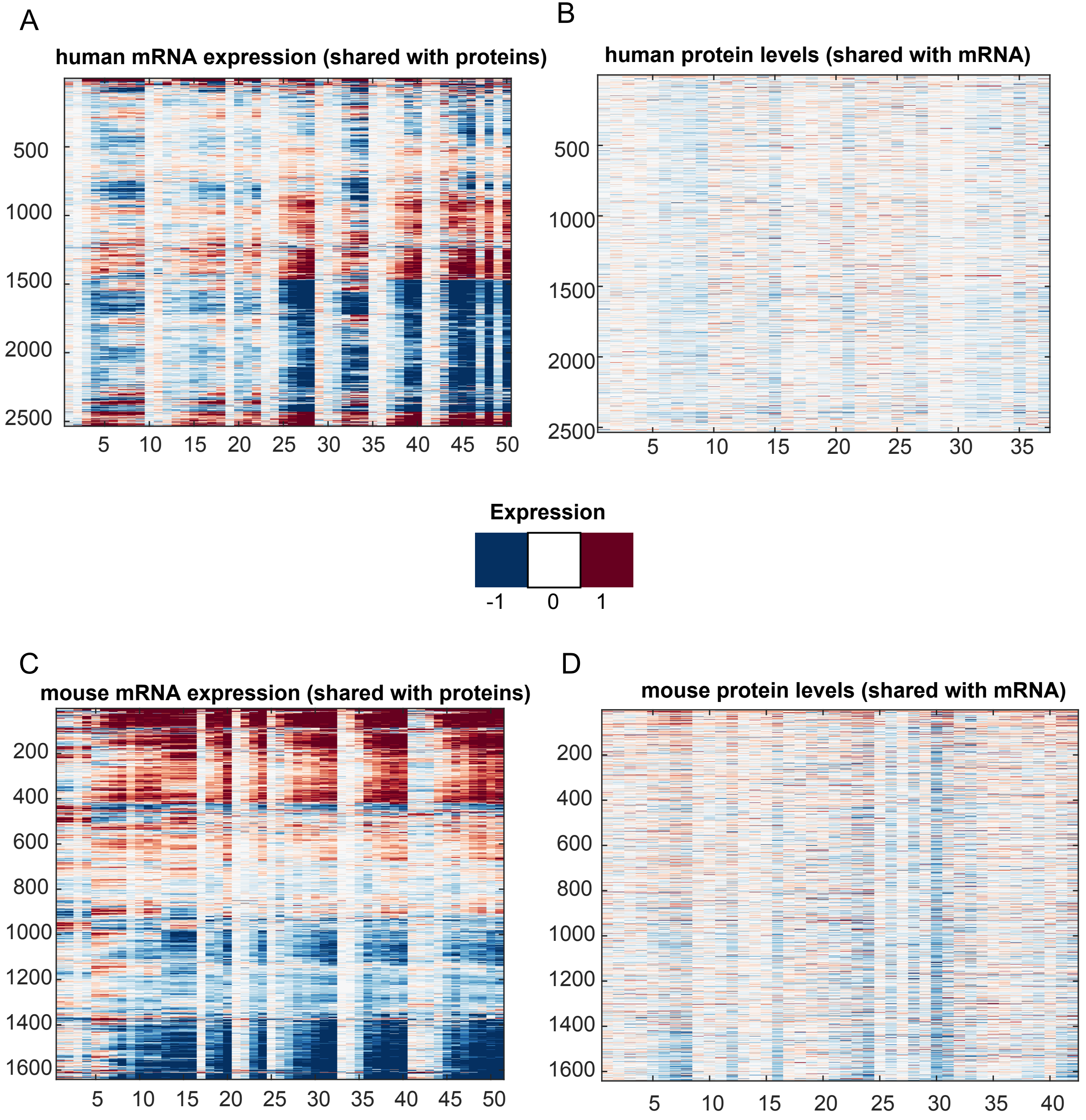
A
human mRNA expression (shared with proteins)
500
1000
1500
2000
2500
5 10 15 20 25 30 35 40 45 50
B
human protein levels (shared with mRNA)
500
1000
1500
2000
2500
5 10 15 20 25 30 35
Expression
-1 0 1
C
mouse mRNA expression (shared with proteins)
200
400
600
800
1000
1200
1400
1600
5 10 15 20 25 30 35 40 45 50
D
mouse protein levels (shared with mRNA)
200
400
600
800
1000
1200
1400
1600
5 10 15 20 25 30 35 40
