# Supplementary material for: Integrating Transcriptomic and Proteomic Data Using Predictive Regulatory Network Models of Host Response to Pathogens
Source: PLoS Comput Biol. 2016 Jul 12;12(7):e1005013. doi: 10.1371/journal.pcbi.1005013 (PMC4942116; doi:10.1371/journal.pcbi.1005013)
Supplement: S4 Fig — Left column human; right column mouse. A. Counts of nonzero regression weights (Y-axis) identified at each level of λ (X-axis) for MTG-LASSO and LASSO for human (left) and mouse (right). The human data is the same as in main manuscript Fig 5B. B. Scatterplots comparing cross-validation Pearson correlation values for all modules, with one plot per value of λ, per species. In each scatterplot, there is one point per module. Inset ρ gives Pearson correlation between MTG-LASSO and LASSO per module Pearson correlations. Diagonal line is shown for comparison. C. Scatterplots comparing cross-validation RMSE values for all modules, with one plot for each value of λ, per species. In each scatterplot, there is one point per module. Inset ρ gives Pearson correlation between MTG-LASSO and LASSO RMSE values. Diagonal line is shown for comparison. D/E. Per-module ranking of MTG-LASSO-selected regulators according to LASSO absolute regression weight for human (D) and mouse (E). One AUROC value is obtained per module. Only modules for which MTG-LASSO predicted the module's expression better than random in at least five of six tested λ settings are shown. (PDF) [file pcbi.1005013.s015.pdf]

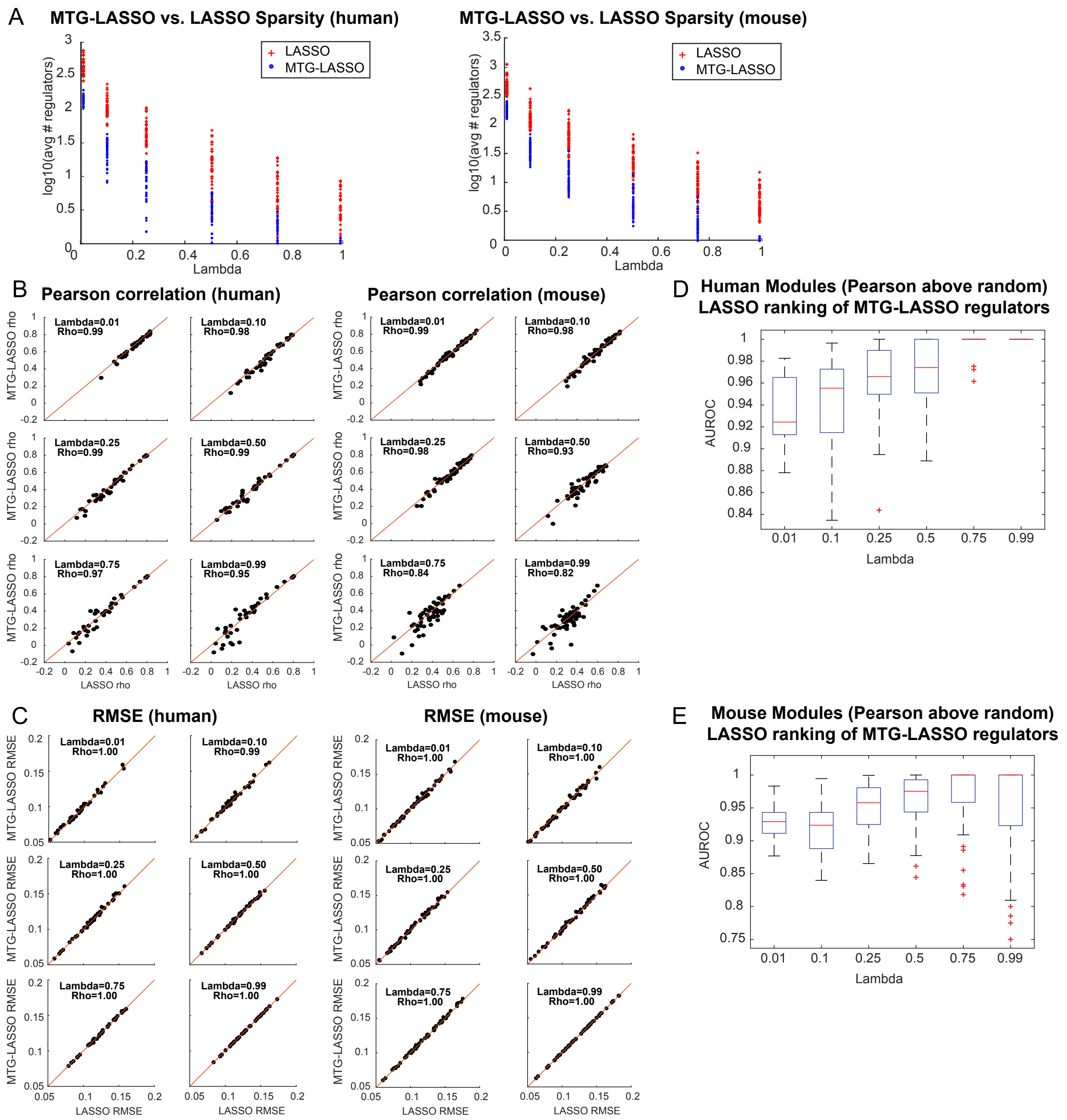
A
MTG-LASSO vs. LASSO Sparsity (human)
MTG-LASSO vs. LASSO Sparsity (mouse)
LASSO
MTG-LASSO
log10(avg # regulators)
Lambda
B
Pearson correlation (human)
Pearson correlation (mouse)
MTG-LASSO rho
LASSO rho
Lambda=0.01 Rho=0.99
Lambda=0.10 Rho=0.98
Lambda=0.25 Rho=0.99
Lambda=0.50 Rho=0.99
Lambda=0.75 Rho=0.97
Lambda=0.99 Rho=0.95
Lambda=0.01 Rho=0.99
Lambda=0.10 Rho=0.98
Lambda=0.25 Rho=0.98
Lambda=0.50 Rho=0.93
Lambda=0.75 Rho=0.84
Lambda=0.99 Rho=0.82
C
RMSE (human)
RMSE (mouse)
MTG-LASSO RMSE
LASSO RMSE
Lambda=0.01 Rho=1.00
Lambda=0.10 Rho=0.99
Lambda=0.25 Rho=1.00
Lambda=0.50 Rho=1.00
Lambda=0.75 Rho=1.00
Lambda=0.99 Rho=1.00
Lambda=0.01 Rho=1.00
Lambda=0.10 Rho=1.00
Lambda=0.25 Rho=1.00
Lambda=0.50 Rho=1.00
Lambda=0.75 Rho=1.00
Lambda=0.99 Rho=1.00
D
Human Modules (Pearson above random)
LASSO ranking of MTG-LASSO regulators
AUROC
Lambda
0.01
0.1
0.25
0.5
0.75
0.99
E
Mouse Modules (Pearson above random)
LASSO ranking of MTG-LASSO regulators
AUROC
Lambda
0.01
0.1
0.25
0.5
0.75
0.99
