# Supplementary material for: Integrating Transcriptomic and Proteomic Data Using Predictive Regulatory Network Models of Host Response to Pathogens
Source: PLoS Comput Biol. 2016 Jul 12;12(7):e1005013. doi: 10.1371/journal.pcbi.1005013 (PMC4942116; doi:10.1371/journal.pcbi.1005013)
Supplement: S6 Fig — Comparison of Calu-3 active regulatory networks between all virus treatments, all time points, based on edges (A), regulators (B), and targets (C). Cells are shaded according to 'precision' relative to the network on the row of the matrix. Precision is defined here as the size of the intersection (edges, regulators or targets) between the two networks divided by the size of the row network (edges, regulators or targets, respectively). (PDF) [file pcbi.1005013.s017.pdf]

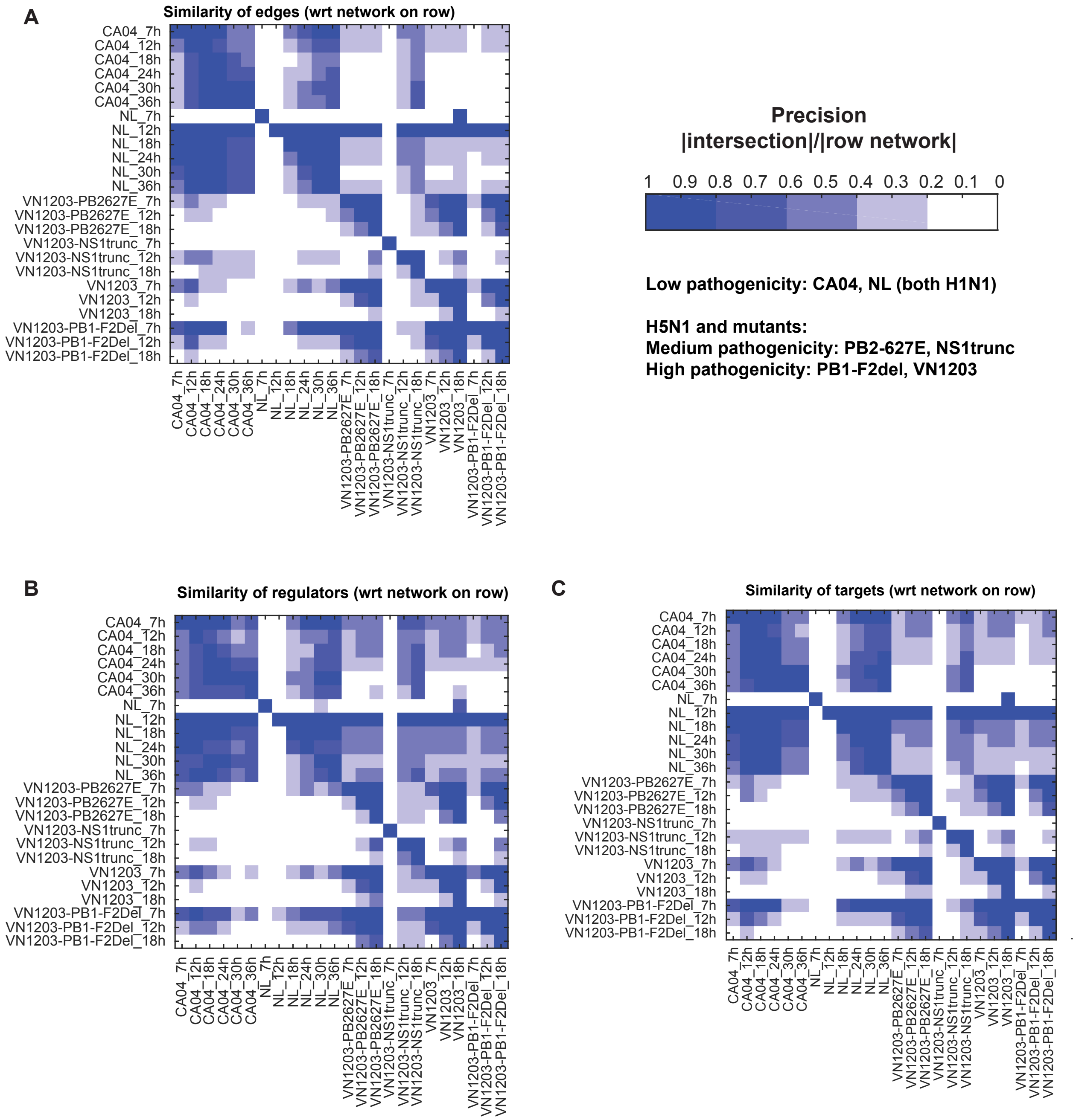

A
Similarity of edges (wrt network on row)
B
Similarity of regulators (wrt network on row)
C
Similarity of targets (wrt network on row)
Precision
|intersection|/|row network|
1 0.9 0.8 0.7 0.6 0.5 0.4 0.3 0.2 0.1 0
Low pathogenicity: CA04, NL (both H1N1)
H5N1 and mutants:
Medium pathogenicity: PB2-627E, NS1trunc
High pathogenicity: PB1-F2del, VN1203
CA04_7h
CA04_12h
CA04_18h
CA04_24h
CA04_30h
CA04_36h
NL_7h
NL_12h
NL_18h
NL_24h
NL_30h
NL_36h
VN1203-PB2627E_7h
VN1203-PB2627E_12h
VN1203-PB2627E_18h
VN1203-NS1trunc_7h
VN1203-NS1trunc_12h
VN1203-NS1trunc_18h
VN1203_7h
VN1203_12h
VN1203_18h
VN1203-PB1-F2Del_7h
VN1203-PB1-F2Del_12h
VN1203-PB1-F2Del_18h
