# Supplementary material for: Integrating Transcriptomic and Proteomic Data Using Predictive Regulatory Network Models of Host Response to Pathogens
Source: PLoS Comput Biol. 2016 Jul 12;12(7):e1005013. doi: 10.1371/journal.pcbi.1005013 (PMC4942116; doi:10.1371/journal.pcbi.1005013)
Supplement: S9 Fig — McDermott et al. identified modules using hierarchical clustering. Edges between modules represent fold-enrichment of the McDermott module's overlap with the MERLIN module relative to all genes in the intersection of the two sets of modules. (PDF) [file pcbi.1005013.s020.pdf]

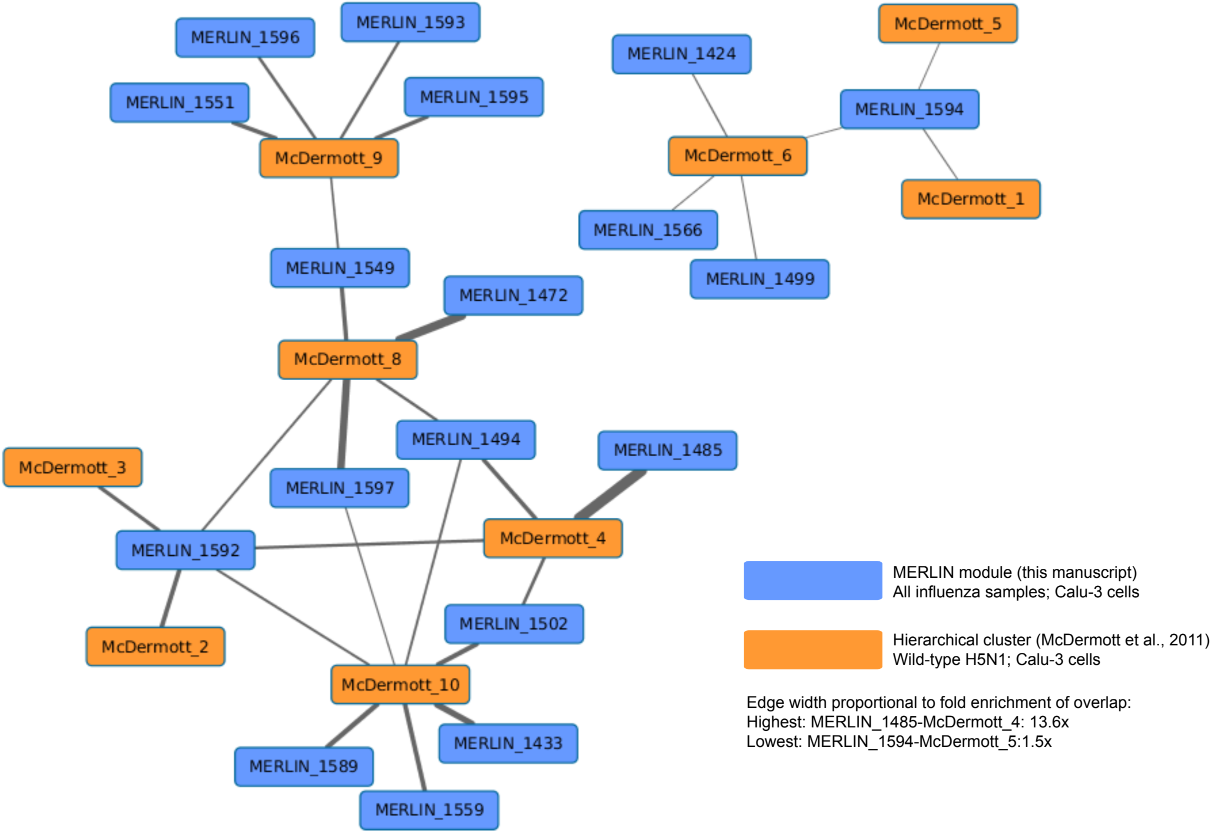
MERLIN_1596
MERLIN_1593
MERLIN_1551
MERLIN_1595
McDermott_9
MERLIN_1424
McDermott_5
MERLIN_1594
McDermott_6
McDermott_1
MERLIN_1566
MERLIN_1499
MERLIN_1549
MERLIN_1472
McDermott_8
MERLIN_1494
MERLIN_1485
McDermott_3
MERLIN_1597
McDermott_4
MERLIN_1592
MERLIN_1502
McDermott_2
McDermott_10
MERLIN_1433
MERLIN_1589
MERLIN_1559
MERLIN module (this manuscript)
All influenza samples; Calu-3 cells
Hierarchical cluster (McDermott et al., 2011)
Wild-type H5N1; Calu-3 cells
Edge width proportional to fold enrichment of overlap:
Highest: MERLIN_1485-McDermott_4: 13.6x
Lowest: MERLIN_1594-McDermott_5:1.5x
