# Supplementary material for: Integrating Transcriptomic and Proteomic Data Using Predictive Regulatory Network Models of Host Response to Pathogens
Source: PLoS Comput Biol. 2016 Jul 12;12(7):e1005013. doi: 10.1371/journal.pcbi.1005013 (PMC4942116; doi:10.1371/journal.pcbi.1005013)
Supplement: S1 Text — (PDF) [file pcbi.1005013.s021.pdf]

S1 Text Assessment of regulator prioritization schemes

We compared the MERLIN-based regulator ranking scheme to other network-based prioritization measure. Each ranking measure scores a regulator using the topology or parameters of the consensus MERLIN network:

- **MERLIN**: increase in the model's error in predicting the regulator's targets' expression when the regulator is removed (see manuscript for details).
- **Regression:** sum of absolute values of outgoing regression weights, obtained by training the consensus MERLIN network on all data
- **Out-degree**: number of outgoing edges (targets)
- **Eigen**: directed version of eigenvector centrality, using the right-hand eigenvector (scores are based on scores of outgoing neighbors)

All pairs of rankings were on the whole similar, with Spearman's correlation values ranging from 0.69-0.88 (human/Calu-3) and 0.70-0.89 (mouse) (Table 1). The MERLIN ranking was most similar to the out-degree ranking.

We also compared the rankings based on their ability to give high ranks to known influenza host factors from independent screening studies (the same as were used to evaluate the MERLIN modules; see main text, **Materials and Methods**). We calculated the precision (fraction of known influenza host factors) in the top n samples for $n = 20, 30, 40, 50, 60$ for all rankings (**S1 Fig**). In both species, the MERLIN ranking achieves the highest precision for $n <= 40$, though the scores appear to be converging as n increases.

Table 1: Comparison of rankings by Spearman's correlation.

Species	Comparison	Spearman's ρ
Human	MERLIN–Eigen	0.69
Human	MERLIN–Out-degree	0.79
Human	MERLIN–Regression	0.68
Human	Eigen–Out-degree	0.88
Human	Eigen–Regression	0.73
Human	Regression–Out-degree	0.84
Mouse	MERLIN–Eigen	0.78
Mouse	MERLIN–Out-degree	0.80
Mouse	MERLIN–Regression	0.70
Mouse	Eigen–Out-degree	0.89
Mouse	Eigen–Regression	0.73
Mouse	Regression–Out-degree	0.82
