# Supplementary material for: Integrating Transcriptomic and Proteomic Data Using Predictive Regulatory Network Models of Host Response to Pathogens
Source: PLoS Comput Biol. 2016 Jul 12;12(7):e1005013. doi: 10.1371/journal.pcbi.1005013 (PMC4942116; doi:10.1371/journal.pcbi.1005013)
Supplement: S2 Text — (PDF) [file pcbi.1005013.s022.pdf]

S2 Text Integer program for identifying high-confidence physical subnetworks

We desired to use physical interaction data to identify potentially mechanistic interactions among our predicted human influenza response regulators from the MERLIN (mRNA) and MTG-LASSO (protein) analyses. We do this on a per-module basis.

We begin by identifying directed, acyclic candidate paths through a physical interaction network. A path is a chain of nodes and edges that begins with one regulator and ends with another that shares predicted targets. We orient these paths such that protein regulators and MERLIN signaling proteins or TFs can be sources at the top, and only MERLIN-identified transcription factors can be sinks at the bottom.

As the subnetwork that results from this entire set of paths is likely large, we wish to identify a smaller, high-confidence physical subnetwork. We accomplish this by solving an integer linear program (ILP). Our formulation is similar to that employed by previous work (47).

We use binary variables to encode the status of each node, edge, and candidate path in an extracted subnetwork, and the direction of each edge. Linear constraints are used to describe how to select paths from the background network. To prune the candidate paths into a high-confidence subnetwork, we apply a series of objective functions that optimize for connecting many regulators with few intermediate nodes, preferring intermediate nodes that are known influenza host factors.

S2.1 Notation

The input to the method is represented as a graph of nodes $\mathcal{N}$, edges $\mathcal{E}$, and candidate paths $\mathcal{P}$. All nodes represent proteins. Each of the set of regulators that we identified from our integrative analysis is represented by a node subset: protein regulators $\mathcal{N}^R$ and MERLIN regulators $\mathcal{N}^M$.We also include interaction host factors identified by Watanabe *et al* (46) as $\mathcal{N}^H$.

The set of edges in the background network is $\mathcal{E} = (\mathcal{E}^D \cup \mathcal{E}^U)$, where $\mathcal{E}^D$ is the set of directed edges and $\mathcal{E}^U$ is the set of undirected edges. We denote an edge e between nodes n_i and n_j as $e = (n_i, n_j)$. $\mathcal{N}(e)$ refers to the nodes connected by a particular edge e, and $\mathcal{E}(n)$ refers to the edges that touch a particular node n.

We find paths to connect the source-sink pairs that are in the set S. We pair up each MERLIN regulator as a source with each MERLIN transcription factor as a sink if they share at least three predicted targets in the module and consensus regulatory network. We then pair up all protein regulators as sources with all MERLIN regulators as sinks. The protein regulators are predicted to influence the regulation of all genes in the module.

To refer to the paths between a specific source regulator s and sink regulator t, we use the notation $\mathcal{P}(s, t)$.

Each path p specifies a direction for each of its undirected edges e, which is denoted as $dir(p, e)$. $\mathcal{E}(p)$ and $\mathcal{N}(p)$ refer to the edges and nodes in a particular path p.

S2.2 Variables

The selection of path p is represented with the variable σ_p, which takes the value 1 if the path is included in the subnetwork, and 0 if it is not. As many as two variables describe each edge. The selection of an edge e is represented with the variable x_e, which takes the value 1 if the edge is in at least one selected path. For undirected edges in the background network, the variable d_e represents the inferred direction of the edge. Each node n has one variable: y_n, representing whether or not the node is present in any included paths. Finally, each source-sink pair (s, t) receives a variable, $c_{s,t}$, representing whether the two are connected by any selected path.

Table 2: Network elements.

Elements	Set	Description
Nodes	$\mathcal{N}$	All nodes
	$\mathcal{N}^R$	Protein regulators
	$\mathcal{N}^M$	MERLIN regulators
	$\mathcal{N}^H$	Interaction host factors
Pairs	$\mathcal{S}$	Source-sink pairs of regulators
Edges	$\mathcal{E}$	All edges
	$\mathcal{E}^D$	Directed edges
	$\mathcal{E}^U$	Undirected edges
	$\mathcal{E}(n)$	Edges that touch node n
Paths	$\mathcal{P}$	All paths
	$\mathcal{P}(s, t)$	Paths between source s and sink t
	$\mathcal{P}(e)$	Paths that include edge e

Table 3: Integer program variables.

Network elements	Variable	Interpretation
Paths p	σ_p	Selected into subnetwork
Edges e	x_e	Selected
	d_e	Direction (back=0, forward=1)
Nodes n	y_n	Selected
Source-sink pairs (s, t)	$c_{s,t}$	Connected by selected path

S2.3 ILP constraints

The following linear constraints define a subnetwork that provides directed paths between sources and sinks.

Include all protein and MERLIN regulators. A subnetwork should include all of the input regulators.

$$\forall n \in \mathcal{N}^R \cup \mathcal{N}^M \qquad y_n = 1$$

How to select paths and edges A selected path p must have all of its edges included in the subnetwork. If only some of the edges are selected (as by other paths), then the path will not be selected. Similarly, for an edge e to be selected (that is, have $x_e = 1$), it must be in at least one selected path p (having $\sigma_p = 1$).

$\mathcal{P}(e)$ refers to the paths that contain e.

$$\forall e \in \mathcal{E} \qquad x_e \leq \sum_{p \in \mathcal{P}(e)} \sigma_p$$

$$\forall p \in \mathcal{P}, e \in \mathcal{E}(p) \qquad \sigma_p \leq x_e$$

Selection of nodes A node n is selected for the subnetwork only if it touches at least one selected edge e. An edge e can only be selected if each of its nodes n is as well.

$$\forall n \in \mathcal{N} \qquad y_n \leq \sum_{e \in \mathcal{E}(n)} x_e$$

$$\forall e \in \mathcal{E}, n \in \mathcal{N}(e) \qquad x_e \leq y_n$$

All edges must be directed so that each path proceeds forward. The required direction for each edge, $dir(p, e)$, is saved when the candidate path is generated.

$$\forall p \in \mathcal{P}, e \in \mathcal{E}(p) \cap \mathcal{E}^U \qquad \sigma_p \leq I(d_e = dir(p, e))$$

A source-sink pair (s, t) is only recorded as being connected if at least one selected path connects them.

$$\forall (s, t) \in \mathcal{S} \qquad c_{s,t} \leq \sum_{p \in \mathcal{P}(s,t)} \sigma_p$$

S2.4 Multiple solutions to the ILP represent an ensemble of subnetworks

We define an optimal subnetwork as one shows how each source may modulate each of its target-sharing sinks, using only a small number of intermediate nodes. An optimal subnetwork prefers to use interaction host factors as intermediates when possible. We identify optimal subnetworks by solving a series of objective functions below.

As multiple optimal choices of subnetworks may be available, we find multiple solutions to the ILP.

Step 1: Identify the maximum number of pairs that can be connected. First, we solve the ILP to identify $\texttt{max\_pairs}$, the maximum number of source-sink pairs that can be connected in the selected subnetwork. This quantity may be lower than the total number of pairs if connecting some pairs would violate our constraint on choosing one direction for each edge.

$$\texttt{max\_pairs} = \max \sum_{(s,t)\in\mathcal{S}} c_{s,t}$$

After identifying this value, we add a new constraint to the ILP so that we require the final selected subnetwork to connect at least this many pairs.

$$\max \sum_{(s,t)\in\mathcal{S}} c_{s,t} = \texttt{max\_pairs}$$

Step 2: Find an ensemble of minimal node solutions. We use this objective function to penalize the selection of intermediate nodes that are not known to be host factors.

$$\min \sum_{n\in\mathcal{N}-\mathcal{N}^R-\mathcal{N}^M-\mathcal{N}^H} y_n$$

We use the CPLEX solver's Solution Pool functionality to accumulate up to 40 solutions (if available) to the ILP for this objective function. Each solution gives a minimal node set, corresponding to a high-confidence subnetwork.

Step 3: Identify all possible paths connecting each minimal node set. Finally, we take each minimal node set and determine which directed paths can be used to connect them. This step does not affect the node choice and merely ensures that all possible directed paths are selected, as choices of paths may otherwise be arbitrary.

For each minimal node set identified in the previous step, we solve the following objective function to select all paths between them.

For each Step 2 solution:

1. Define the selected node set for this subnetwork. Add constraints to fix each value of y_n to its value from the Step 2 solution, $\hat{y_n}$.

$$\forall n \in \mathcal{N} \qquad\qquad y_n = \hat{y_n}$$

2. Solve the IP to maximize the number of selected paths among the nodes.

$$\max \sum_{p \in \mathcal{P}} \sigma_p$$

S2.5 Defining the high-confidence subnetwork

Together the IP solutions define an ensemble of directed subnetworks, with which we assess confidence values on the paths. We define a high-confidence consensus subnetwork by thresholding the path confidence at 0.75. Because some pairs of regulators may have multiple choices of paths through different intermediate nodes, some regulators may not be included in the final consensus subnetwork.
