# Supplementary material for: Integrating Transcriptomic and Proteomic Data Using Predictive Regulatory Network Models of Host Response to Pathogens
Source: PLoS Comput Biol. 2016 Jul 12;12(7):e1005013. doi: 10.1371/journal.pcbi.1005013 (PMC4942116; doi:10.1371/journal.pcbi.1005013)
Supplement: S3 Text — (PDF) [file pcbi.1005013.s023.pdf]

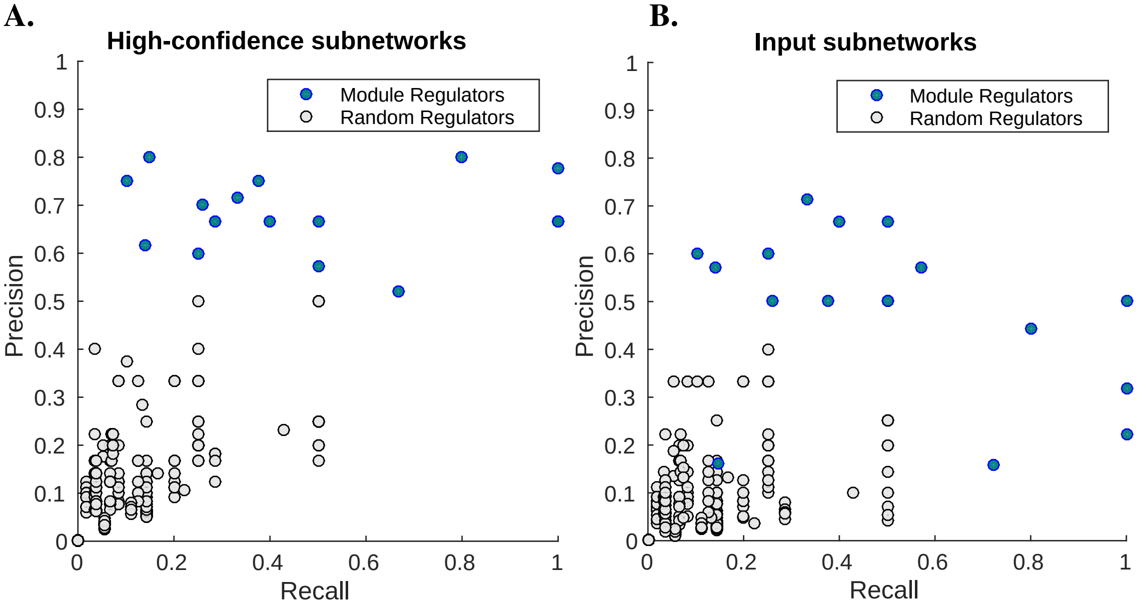

Figure 1: Proximity of real and random regulators in physical subnetworks. Precision is the fraction of subnetwork nodes that were input regulators; recall is the fraction of total module regulators that were captured by the subnetwork. **(A)** High-confidence subnetworks (after optimization). **(B)** Input subnetworks subnetworks (before optimization).

S3 Text Compactness of inferred subnetworks

We wanted to assess whether the number of intermediate nodes in the inferred subnetworks was significantly low, or merely a function of centrality of regulators in the protein interaction network. For each module, we assess the (input) *precision* as the fraction of subnetwork nodes that are input regulators, and the (input) *recall* as the fraction of input nodes that the subnetwork could capture. We calculate these values for the 16 'real' human subnetworks as well as the subnetworks inferred from random regulators (40 per module). Random regulators were selected to maintain the same degree distribution as the real regulators for the corresponding module. We omit interaction host factors from Watanabe *et al* (2014) from both precision and recall, as these were provided as input to both methods. (NB: Although we use the terms precision and recall for convenience, these are assessed on training data and cannot be interpreted as a measure of how well the method will generalize to predicting other relevant genes.)

Plots of the values for all subnetworks (real and random) are given in Figure 1. The high-confidence subnetworks all have higher precision than the random subnetworks (Panel A), suggesting that the real regulators are more tightly connected than expected even controlling for node degree. We also see that even among input paths (Panel B), the subnetworks for real module regulators have either higher precision or recall than random.
